# RNA profiling identifies novel, photoperiod-history dependent markers associated with enhanced saltwater performance in juvenile Atlantic salmon

**DOI:** 10.1371/journal.pone.0227496

**Published:** 2020-04-08

**Authors:** Marianne Iversen, Teshome Mulugeta, Børge Gellein Blikeng, Alexander Christopher West, Even Hjalmar Jørgensen, Simen Rød Sandven, David Hazlerigg

**Affiliations:** 1 Department of Arctic and Marine Biology, UiT -The Arctic University of Norway, Tromsø, Norway; 2 Department of Animal and Aquaculture Sciences, Norwegian University of Life Sciences, Ås, Norway; 3 Centre for Integrative Genetics, Department of Animal and Aquaculture Sciences, Norwegian University of Life Sciences, Ås, Norway; National Cheng Kung University, TAIWAN

## Abstract

Atlantic salmon migrate to sea following completion of a developmental process known as smolting, which establishes a seawater (SW) tolerant phenotype. Smolting is stimulated by exposure to long photoperiod or continuous light (LL) following a period of exposure to short photoperiod (SP), and this leads to major changes in gill ion exchange and osmoregulatory function. Here, we performed an RNAseq experiment to discover novel genes involved in photoperiod-dependent remodeling of the gill. This revealed a novel cohort of genes whose expression rises dramatically in fish transferred to LL following SP exposure, but not in control fish maintained continuously on LL or on SP. A follow-up experiment revealed that the SP-history dependence of LL induction of gene expression varies considerably between genes. Some genes were inducible by LL exposure after only 2 weeks exposure to SP, while others required 8 weeks prior SP exposure for maximum responsiveness to LL. Since subsequent SW growth performance is also markedly improved following 8 weeks SP exposure, these photoperiodic history-dependent genes may be useful predictive markers for full smolt development.

## Introduction

In anadromous salmonids, the transformation of freshwater resident juvenile fish (parr) into a migratory form (a smolt) which will migrate downstream migration and enter the sea is known as smoltification or smolting. Smolting entails a complex combination of physiological and behavioural changes, critical amongst which is the acquisition of the ability to efficiently maintain water and ionic balance upon entering the sea [1, 2].

In natural systems smolting is stimulated by the increasing day length (photoperiod) in spring, causing a cascade of physiological responses mediated by changes in circulating endocrine signals [1, 3–5]. The aquaculture industry depends on this photoperiod-dependence in the production of seawater (SW) tolerant juvenile salmon for transfer to sea cages in which rapid growth can take place. Smolting is artificially achieved by exposing juvenile salmon exceeding a minimum size threshold to short photoperiod (SP) for several weeks and then returning them to continuous light (LL). Based on observations of SW performance, it has been shown that the duration of exposure to SP should be at least six weeks long for LL to induce smolting [6]. The underlying causes of this photoperiodic history-dependence remain unknown, and untangling the role of SP exposure in smolt development is of considerable interest, as SP exposure reduces growth rates and slows aquaculture production.

The gill has a pivotal role in the energy demanding regulation of water and ionic fluxes, and it therefore undergoes extensive differentiation during the smolting process to pre-adapt to the SW environment. Within the gill, mitochondria rich cells (MRCs) are considered the primary drivers of ionic regulation, and smolting includes a pronounced shift in the location and phenotype of MRCs in the gill [7, 8]. During smolting the gill complement of MRCs shifts from an ion-absorbing FW type to an ion-secreting SW type, and the distribution of MRCs shifts from the lamellae to the gill filament itself [9]. Differences between FW and SW MRCs include a redistribution and change in composition of ionic pumps [2, 8, 10], the occurrence of an apical crypt and an extensive tubular network in the SW MRC, and the interdigitation of SW MRCs by accessory cells (ACs) [11–14].

The Na^+^, K^+^- ATPase (NKA) pump in the MRC cells has become an established marker for smolt state due to its marked increase in activity during smolting [1]. Further, it has become evident that its catalytic α-subunit has two protein isoforms, α1a and α1b, which are predominant in freshwater MRCs and in salt-water MRCs, respectively [2, 15–17]. Cystic fibrosis transmembrane conductance regulator I (CFTR I) is another ion channel protein considered a marker for smolt state due to upregulation of its mRNA during smolting [5, 18, 19]. Transcriptomic profiling studies have revealed additional genes associated with smolting in salmonids [20–22], and these offer the potential for increased understanding of the smolting process as well as providing novel markers.

The degree to which remodeling of gill tissue during smolting depends on photoperiodic history remains poorly characterized because extant studies have not sought to resolve history-dependent effects of photoperiod from direct effects of light or developmental age [4, 8, 23]. What is clear is that prior exposure to short photoperiod may enhance the capacity of juvenile salmon to perform well following transfer to SW [6, 24]. To assess the extent to which this priming effect of SP affects gill development, we have performed RNA profiling in smolting Atlantic salmon subjected to a range of different lighting protocols. Our data reveal a novel cohort of genes which expression is dramatically induced by exposure to LL, conditional on prior exposure to SP. Further, we show that history-dependence varies between genes which allows the identification of novel markers whose expression patterns are good predictors of subsequent SW growth performance.

## Materials and methods

### Animal welfare statement

The experiments were conducted as part of the continuously ongoing smolt production at Tromsø Aquaculture Research Station, approved by the Norwegian Animal Research Authority (NARA) for hold of, and experiments on salmonids, fresh- and salt-water fish and marine invertebrates. In accordance with Norwegian and European legislation related to animal research, formal approval of the experimental protocol by NARA is not required when the experimental conditions are practices undertaken routinely during recognized animal husbandry, and no compromised welfare is expected.

### Fish

Atlantic salmon (*Salmo salar*, Linnaeus, 1758, of the Aquagene commercial stain, Trondheim, Norway) were used for both experiments, and were raised from hatching in FW, on continuous light (LL, > 200 lux at water surface) at 10°C (Experiment 1) and 4°C (Experiment 2). Fish were fed continuously with pelleted salmon feed (Skretting, Stavanger, Norway).

### Experimental set-up

During both experiments, all experimental groups were fed pellet salmon feed continuously and in excess with automatic feeders for eight hours a day, corresponding to the light phase under SP.

**Fig 1:**
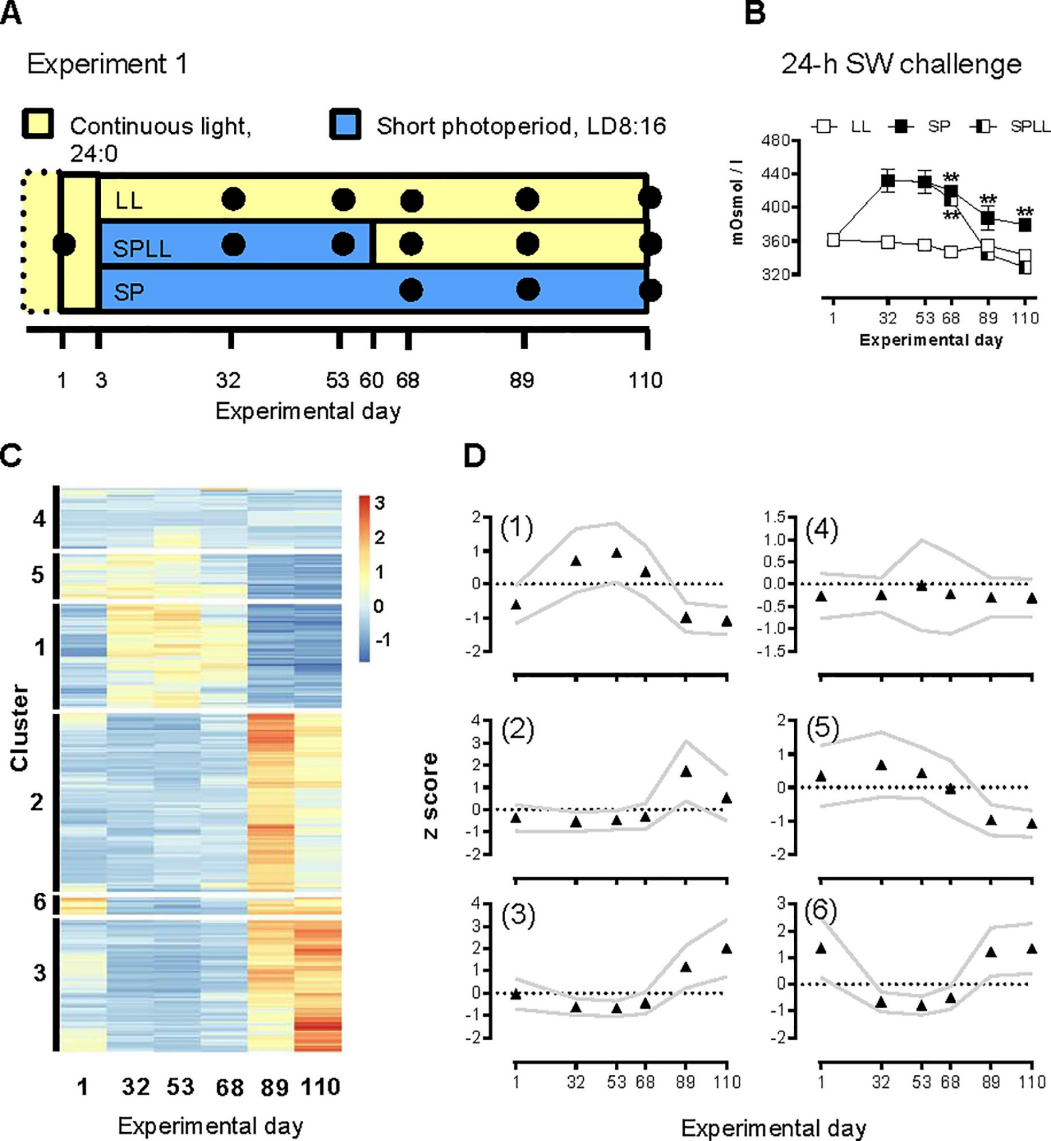
RNA profiling of photoperiodic history-dependent changes in gill gene expression in juvenile Atlantic salmon. A) Schematic presentation of experiment 1. Sampling time-points are indicated by black dots. B) Plasma osmolality (mOsm kg^-1^) following a 24-h SW challenge; data are mean ± SEM of n = 6 fish per sampling point. ** significantly higher osmolality than in LL and SPLL fish at the same sampling point, p<0.01. Where error bars do not appear, errors lie within the symbol. C) Heatmap showing genes that are highly differentially expressed (FDR<0.01, logFC >|2|) between experimental groups over the three latter sampling points of exp.1. Hierarchical clustering has been used to generate six clusters. D) The averaged expression profile (z score) of the six clusters of DEGs, data are mean ± SEM, n = 6.

Experiment 1: This experiment utilized 237 juvenile salmon kept in a 500 L circular tank since start of feeding. The experiment was begun when the salmon juveniles had reached approximately 7 months of age (02.12.2013) and a mean weight of 49.5 g (s.d. ± 7.0 g, n = 6). One initial sampling (set as day 1 of the experiment, and referred to as pre-SP) was done in order to establish a pre-smolt baseline. Two days later (Day 3), 225 parr were taken from the original tank and randomly allocated into two 100 L circular tanks (FW, 8.5°C) in separate rooms. One tank received 75 parr, and was kept on LL for the remainder of the experiment. The other tank received 150 parr, and the photoperiod was gradually decreased over a week from LL to short photoperiod of 8-h light/24-h (SP). Further samplings from both these groups were done on Days 32 and 53 (n = 6). At Day 60 half of the remaining SP group was moved to a new 100L and returned to LL (SPLL). Further sampling of the three groups were done on Days 68, 89, and 110, as shown in Fig 1.

Experiment 2: This experiment utilized 1400 fish at approximately 11 months old (beginning on 05.01.2017), weighing an average of 40.3 g (s.d. ± 9.7 g, n = 10). The juvenile salmon were distributed among eight 300 L circular tanks with FW at 7°C and LL, and left to acclimate for one week. The total number of fish in each tank ranged from 150 to 200, depending on the number of fish to be sampled during the experiment in each tank and the need to avoid density-dependent social stress effects.

After an initial sampling at the last day of acclimation under continuous light (Day 1), fish in all tanks were transferred to SP. One group of fish remained on SP for 16 weeks (SPC group), while the three other groups were kept on SP for two, four or eight weeks (2WSP, 4WSP and 8WSP groups, respectively; collectively termed the SP-LL groups). LL exposure then continued for a further 8 weeks. All treatments were run in duplicate tanks. After the initial sampling, all SP-LL groups were sampled on the last day of SP, and at four and eight weeks post-SP. For the SP-LL groups the two post-SP sampling points corresponded to 196 and 392 degree-days (°d) after re-entering LL. At each of these sampling points, samples were also collected from the SPC group.

#### 24-hours Salt-Water Challenge (SWC)

In both experiments, 24-h prior to each FW sampling point, randomly selected fish (n = 6 for exp.1, and n = 10 for exp.2) were transferred to 100 L tanks supplied with full strength SW (7°C, 34‰ salinity) for 24 hours. No feed was given during this 24-h period. There were no mortalities during the SWCs. After 24 h, the fish were netted out and lethally anesthetized (10 L water container, SW, Benzocaine, 150 ppm), followed by blood sampling, decapitation and tissue dissection as described below.

#### Blood sampling and tissue dissection

Following lethal anesthesia (in 10 L water container, FW or SW as appropriate, Benzocaine, 150 ppm), body masses (± 0.5 g) and fork lengths (± 0.1 cm) were recorded (For FW, n = 6 for exp.1 and n = 10 for exp.2). Blood was collected from the caudal vein into 2 ml Lithium-heparinized vacutainers (BD vacutainers®, Puls Norge, Moss, Norway), and placed on ice until it was centrifuged (6000 x *g*) for 10 min. The plasma fraction was collected and stored at -20°C for later analysis of osmolality and chloride concentration. Fish were then decapitated and dissected. After decapitation, the operculum on the right side of the head (caudal view) was removed and primary gill filaments were collected and placed in RNAlater® (Sigma-Aldrich, St. Louis, Missouri, USA) for transcript and qPCR analyses. Samples were stored at 4°C for 24 h, and then kept frozen at -80°C until further processing.

During Experiment 2, two secondary filaments (2–3 mm) were also collected and placed in 100 μL ice cold SEI buffer (0.15 M sucrose, 0.01 M Na_2_EDTA, 0.05 M Imidazole, in H_2_O, pH 7.3, Sigma-Aldrich, St. Louis, Missouri, USA) and immediately frozen at -80°C until analyses of NKA activity.

#### Prolonged salt-water exposure following smolt induction

Following maintenance on LL for eight weeks, 30 randomly selected fish from each of the SP-LL groups (fifteen from each duplicate tank) were netted out and anaesthetized (Benzocaine, 60 ppm), and fork length and body mass was measured. After recovery fish were placed in 300 L, circular tanks supplied with full strength SW (34 ‰) at 7°C and continuous light. Fish were fed pelleted salmon feed continuously and in excess by automatic feeders. The amount of feed eaten was monitored daily by collection of feed remnants from the tank outlet sieve. After 15 days in SW, fork length and body mass were again recorded as above, before returning the fish to the SW tanks for a further 15 days. On day 30 of SW exposure, all fish were anaesthetized with benzocaine (150 ppm, Sigma-Aldrich, St. Louis, Missouri, USA), after which fork length and body mass were recorded, and the fish decapitated. No fish died during the prolonged SW exposure. Fish from the SPC group were not subjected to extended SW exposure for animal welfare reasons associated with the anticipated lack of SW tolerance.

### Analyses

#### Plasma osmolality and chloride levels

Thawed plasma samples were analysed using a Fiske One-Ten Osmometer (Fiske Associates, Massachusetts, USA, ± 4 mOsm kg^-1^) and a Chloride Analyzer from CIBA Corning Diagnostics (Essex, England, ± 2.2 mmol L^-1^).

#### NKA activity

NKA activity (experiment 2) was measured in gill samples by a method described by McCormick [25] and Schrock et al. [26]. The assay measures the rate of hydrolysis of ATP to ADP and P_i_, which is coupled to the oxidation of NADH to NAD^+^. Briefly, the gill tissue was thawed and homogenized in SEI buffer, and the supernatant assayed for ATP-ADP hydrolysis activity, with and without the NKA activity inhibitor ouabain, by spectrophotometer readings (Spectramax Plus 384, Molecular Devices Corp., California, USA) at 340 nm at 30 second intervals over 10 minutes. Protein was measured using the Pierce BCA Protein Assay kit (Thermo Fisher, Waltham, Massachusetts, USA) utilizing a bicinchoninic acid method [27]. From the measured change in ADP levels and amount of protein, NKA activity is expressed in units of activity per mg protein.

#### RNA extraction

Gill tissue was disrupted using TissueLyser II (QIAGEN, Hilden, Germany). Total RNA from experiment 1 was extracted using the RNeasy Plus Universal Mini Kit (QIAGEN, Hilden, Germany). For experiment 2 total RNA was extracted using a TRIzol-based method (Invitrogen, Thermo Fisher, Waltham, Massachusetts, USA) and following the manufacturer’s protocol. RNA concentrations were measured using a NanoDrop ND2000c spectrophotometer (NanoDrop Technologies, Wilmington, DE, USA). RNA samples were frozen at -80°C until further processing.

#### Transcriptome sequencing

Sequencing libraries were prepared using the TruSeq Stranded mRNA HS kit (Illumina, San Diego, California). Library mean length was determined by a 2100 Bioanalyzer using the DNA 1000 Kit (Agilent Technologies, Santa Clara, California, USA) and library concentration was determined with the Qbit BR Kit (Thermo Scientific, Waltham, Massachusetts, USA). Each sample was barcoded using Illumina unique indexes. Single-end 100bp sequencing of sample libraries was carried out on an Illumina HiSeq 2500 at the Norwegian Sequencing Center (University of Oslo, Oslo, Norway).

Cutadapt [28] was used to remove sequencing adapters, trim low quality bases, and remove short sequencing reads using the parameters -q 20 -O 8—minimum-length 40 (version 1.8.1). Quality control of the reads were performed with FastQC software [29]. Mapping of reads to reference genome was done using STAR software (ver. 2.4.2a) [30]. HTSEQ-count software (version 0.6.1p1) [31] was used to generate read count for annotated genes.

#### Transcriptome analysis

Analysis of differential gene expression in experiment 1 was performed with package edgeR (ver. 3.14.0) using R (ver. 3.4.2) and RStudio (ver. 1.0.153). Prior to higher level analyses, the raw counts were filtered, setting an expression level threshold of a minimum of one count per million reads (cpm) in five or more libraries. The counts were scaled by applying trimmed means of M-values (TMM) scaling. The data was fitted with a quasi-likelihood negative binomial generalized log-linear model. Two tests, (empirical Bayes quasi-likelihood F-tests), contrasting between the SPLD group and the LL or SP group for the three latter sampling points (days 68, 89 and 110), were applied to compare the SPLL light regime with the LL and SP regimes. Both outputs were filtered using a false discovery rate (FDR) < 0.01, and for genes to show a log_2_-fold change > |1|.

The resulting outputs from the SPLL vs. LL and SPLL vs. SP comparisons were combined to form a list of unique genes that showed significant photoperiod-dependent changes in expression. The count data (cpm) for those genes was extracted for the SPLL-group, and row-scaled by calculating z-scores. The R-package pheatmap (ver. 1.0.10) was used to clusters the genes into six clusters applying Euclidian distance measures and complete linkage clustering. The sum of squared error (SSE) and gap statistic were used to evaluate which number of clusters to use. Cluster centroids were calculated, and the correlation between centroid and genes checked for uniformity.

One cluster, appearing to represent light responding, consequently upregulated, genes was chosen for further examination. The expression profile of the genes, their expression levels, magnitude of log_2_-fold change and FDR value was evaluated and a small set of six genes (Table 1) for which primers could be successfully be developed and confirmed was selected for further testing of relevance in experiment 2. Gene functions were briefly investigated using GeneCards [32, 33]. Since the gene symbols of the targeted genes consist of numerical string we opted to use HGNC symbols, based on the gene description, when referring to the genes in the text.

**Table 1:** Primer sequences for target genes.

Target	Gene symbol		Sequence (5’ to 3’)	Annealing temperature°C	Product length (bp)
***EF1A***	LOC100136525	F	AGGCTGCTGAGATGGGTAAG	63	218
** **		R	AGCAACGATAAGCACAGCAC		
***NKA a1b (ii)***	LOC 106575572	F	GGGTGTGGGCATCATTTCTG	66	152
** **		R	CATCCAACTGTTCGGCTGAC		
***CFTR I***	LOC 100136364	F	CCTTCTCCAATATGGTTGAAGAGGCAAG	63	81
		R	GCACTTGGATGAGTCAGCAG		
***CAPN2***	LOC 106589985	F	GTTGAGGAGATCGTGGTGGA	65	118
		R	TGTTCAGAATCCTCCGCAGT		
***TPH1***	LOC 106562311	F	ACTTCCTCAGAGAACGCACA	63	218
		R	CTGGGAGAACTGGGCAAAAC		
***S100A1***	LOC 106570104	F	GGATGACCTGATGACGATGC	65	122
		R	ATCACATACTCCCCACCAGG		
***ST6GALNAC2***	LOC 106589898	F	CTTCGACCGCCAATATCACC	63	149
		R	ATGGCAACCTTGAGTGAGTT		
***FKBP5***	LOC 106565346	F	CTGGGAAAGGGTCAGGTGAT	65	264
		R	GACTGTTGATCCGTCGTTGG		
***SLC5A7***	LOC 106602131	F	AGGTGGGACGTGTTTCAGAT	65	203
		R	CCCGACCAACAAAACCCTTT		

#### Real-time quantitative PCR

RNA samples from experiment 2 were ethanol-precipitated and DNAse-treated according to the manufacturer’s protocol (TURBO DNA-free Kit, Thermo Fisher). cDNA was constructed using the High-Capacity RNA-to-cDNA kit (Thermo Fisher, Waltham, Massachusetts, USA), following the recommended protocol.

Primers (Table 1) were designed to target all splice variants of the target genes, while not picking up ohnologue and paralogue duplicates of the targeted genes. Primer3 [34, 35] and ApE software (v2.0.51) were used for designing primers, and primers were checked against both the National Center for Biotechnology Information (NCBI, Bethesda, Maryland, USA) database using BLAST [36] and the SalmoBase database [37] for non-target hits. Primer specificity was confirmed by melt-curve analysis, and amplicon size verified by agarose gel electrophoresis. In order to establish primer amplification efficiencies a subset of samples were pooled and diluted, and analyzed by qPCR. Amplification efficiencies fell between 90% and 110%.

Real-time quantitative PCR analysis was performed using a BioRad CFX Connect Real-Time instrument (Hercules, California, USA), and SYBR Green detection. Reactions were carried out on 96-well plates, with 20 ng RNA cDNA equivalent, 250 nM forward and reverse primer, and 1x Sso Advanced Universal SYBR Green Supermix (BioRad, Hercules, California, USA), in a total volume of 20 μL. After initial heating (95°C, 30 sec.), amplification was carried out under the following conditions: 95°C for 10 sec., and primer-specific annealing temperature for 1 min. over 40 cycles. A melting curve analysis was completed at the end of each run (0.5°C intervals at 3 sec., from 65°C to 95°C).

#### Data analysis and statistics

Condition factor (CF) was calculated as

$$CF=W\times \left(\frac{100}{L^{3}}\right).$$ (1)

where W is wet body mass (g), and L is fork length (cm).

Specific growth rate (SGR) was calculated as

$$SGR=\left[\frac{\left(ln{\bar{W}}_{T}-ln{\bar{W}}_{t}\right)}{\left(T-t\right)}\right]\times 100.$$ (2)

where W_t_ and W_T_ are mass (g) at the beginning and end of the period of extended SW exposure, respectively. Similarly, the feed conversion ratio (FCR) over the same period was calculated by dividing the total amount of ingested food per tank (g, dry weight) by the increase in total biomass for each tank.

The C_t_ values of target genes were normalized against EF1A [38, 39] using the ΔΔC_t_ method described by Livak [40].

GraphPad Prism (ver. 7.03) was used for statistical computation of one- and two-way ANOVAs for physiological measurements and relative mRNA content for both exp.1 and exp.2. Summary statistics are given as mean ± standard deviation (S.E.M.).

Experiment 1: Effects of photoperiod regime (treatment) and time (i.e. time passed after returning to LL for the SPLL group) over the three latter sampling points were assessed by two-way ANOVA, and Tukey’s test for post hoc pairwise comparisons. A one-way ANOVA was applied to test for significant differences between the initial sampling and any other sampling, applying Dunnett’s test for multiple comparisons. The statistical significance threshold was set to p < 0.05.

Experiment 2: Effects of photoperiod regime (treatment) and time (i.e. time passed after returning to continuous light for SP-LL groups) were assessed by two-way ANOVA, and Tukey’s test for post hoc pairwise comparisons. To avoid pseudo-replication of data the initial sampling point (day 1), which is common for all groups, was excluded from the ANOVA analysis. Data from this sampling point is provided in figures for reference, and a one-way ANOVA was performed to test for any significant differences between the initial sampling point and all other samplings, applying Dunnett’s test for multiple comparisons. The statistical significance threshold was set to p<0.05.

## Results

### Experiment 1

Experiment 1 is summarized in Fig 1A.

#### Hypo-osmoregulatory capacity

The capacity to hypo-osmoregulate in response to an acute (24-h) SW challenge was time- and photoperiod dependent (Fig 1B, p<0.0001 for time, photoperiod regime and the interaction term, two-way ANOVA, Fig 1B, S1 Table). On day 1, prior to SP transfer, plasma osmolality after 24-h in SW was about 360 mOsm kg^-1^. The LL group maintained this capacity for hypo-osmoregulation throughout the experiment. Fish that were transferred to SP lost their osmoregulatory capacity by day 32, but then underwent a partial recovery when exposure to SP conti nued to the end of the study. Fish that were transferred back onto LL on day 60 had fully recovered their hypo-osmoregulatory capacity by day 89 of the experiment. Plasma chloride levels followed the same pattern (S1 Fig). Size is not believed to have influenced osmoregulatory capacity post-SP as there were no significant differences in weight between the three groups on day 110 (LL: 91.3 ± S.D, SP: 78.4 ± SD, SPLL: 81.7 ± SD, n = 6 for all groups, S4 Table).

#### RNA profiling of gill tissue

In order to identify novel photoperiod-dependent changes in gill gene expression we analyzed the transcriptome of the gill, focusing on expression changes across the three last sampling points of experiment 1. This revealed 389 unique transcripts showing photoperiod-dependent changes, after applying filtering criteria. Hierarchical clustering identified six major clusters with distinctive profiles (Fig 1C and 1D, S8 Table) for the SPLL group. In numerical order the clusters consisted of 75, 129, 96, 44, 32 and 12 genes.

Clusters 1 and 6 showed reversible photoperiod-dependent changes in expression over the study as a whole, with the *NKA α1b* gene being placed in cluster 6. Contrastingly, cluster 3 was distinctive in exhibiting a photoperiod-dependent increase in expression over the latter time-points of the study, and lower expression levels at the initial sampling point. This suggested to us that this cluster comprised genes involved in photoperiodic history-dependent smolt transition. Apart from CFTR I, which is closely linked to smolting [5, 18, 19], genes in cluster 3 have not previously been linked to gill differentiation during smolting [20, 21, 41].

**Fig 2:**
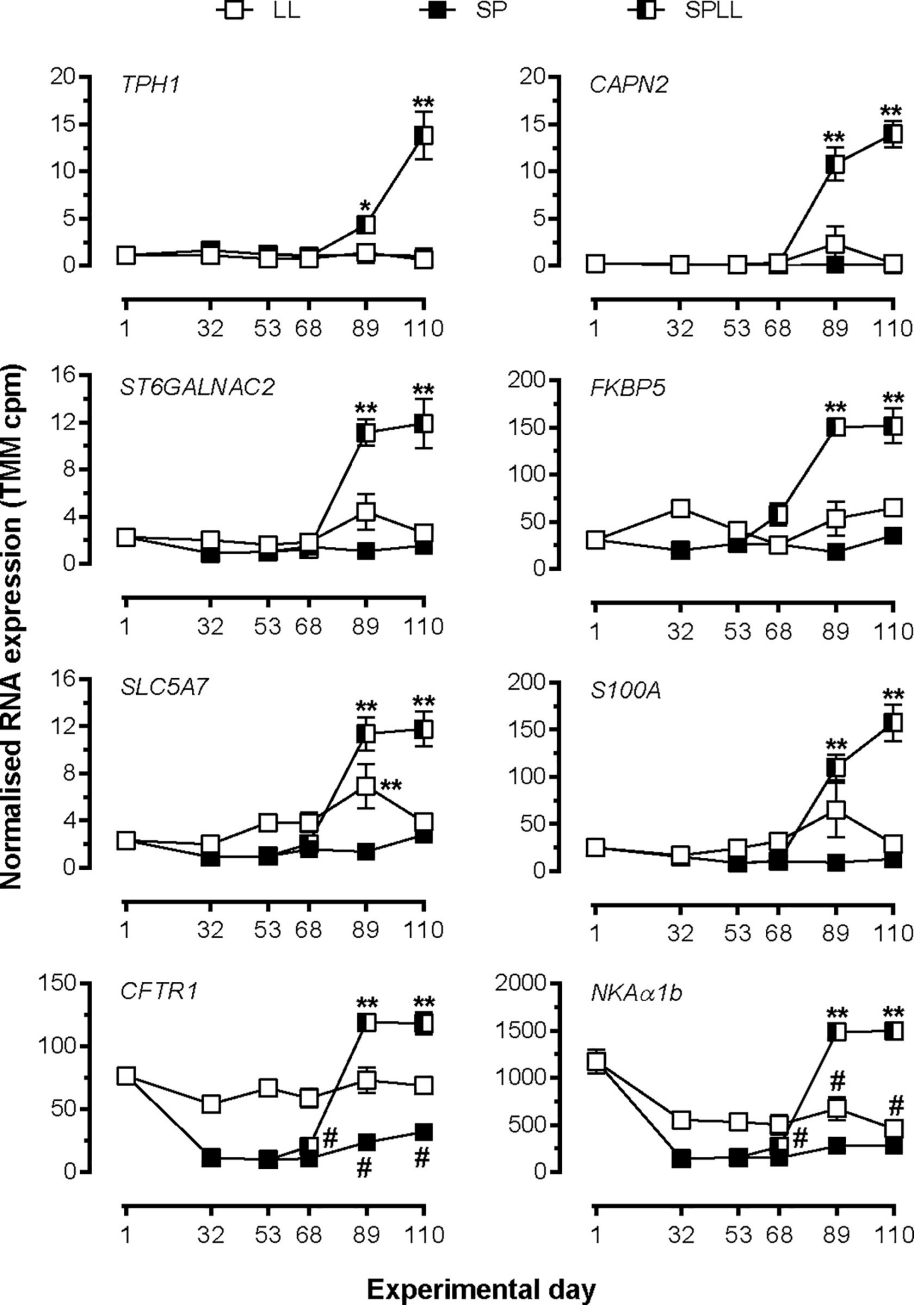
Temporal expression profiling of selected genes from cluster 3 in experiment 1. Data are presented as normalized (TMM) counts and are mean± SEM of n = 6 fish, except for SPLL on day 68 where n = 5. *, ** significantly higher expression than LL and SP values at the corresponding time point, p<0.05, 0.01, respectively; # significantly lower expression than at day 1, p<0.05. Where error bars do not appear, errors lie within the symbol.

Fig 2 shows the TMM normalized RNA expression profiles for 6 novel smolting genes representative of cluster 3, as well as for *CFTR1* and *NKAα1b*. For all 6 novel genes the developmental change in expression over the last 3 sampling points of the study was highly dependent on photoperiodic history (P <0.0001 for treatment x time interaction by 2-way ANOVA, S6 Table). In all cases expression was lowest at day 1, and highest at the end of the study in fish that had been transferred to SP and then returned to LL. In fish maintained throughout on SP or LL, expression levels did not change significantly over time, and final values were markedly lower than in corresponding SPLL fish.

In contrast to these novel genes, the expression of both *CFTR1* and *NKAα1b* was relatively high at day 1 of the experiment and declined markedly with transfer to SP. Return of SP fish to LL led to a return to elevated values, which were 25–50% higher than day 1 values. For both genes maintenance on SP maintained low levels of expression throughout, and for *NKAα1b* continuous exposure to LL caused a progressive decline in expression so that values at the end of the study in LL fish were significantly lower than at the start of study (p<0.0001 by 1-way ANOVA).

### Experiment 2

**Fig 3:**
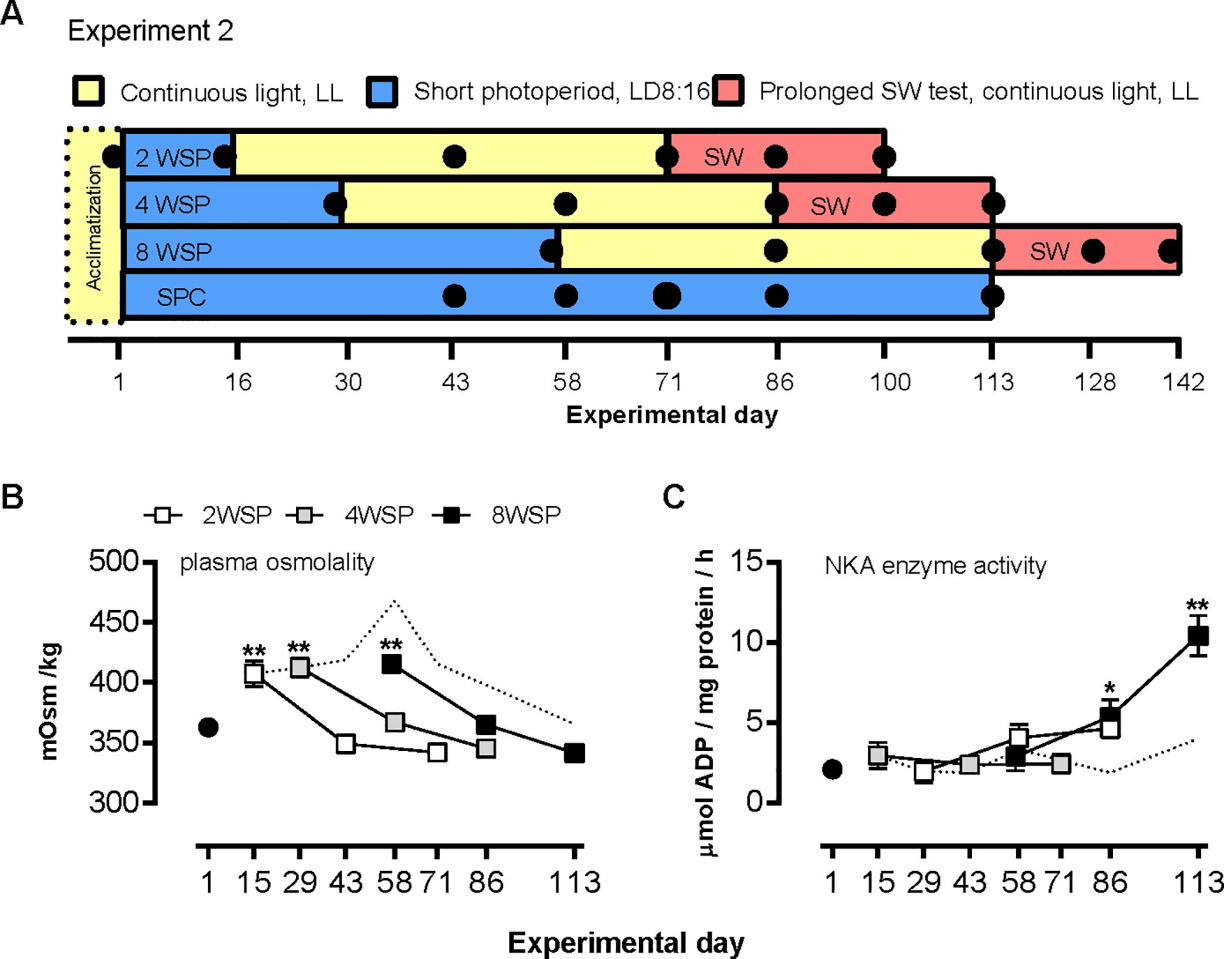
Effect of SP exposure duration on smolting performance parameters. (A) Experimental design for experiment 2. (B) Plasma osmolality after 24-h SW challenge tests at the indicated sampling points. Data are mean ± SEM of n = 9–10 fish per sample point. **, significantly higher values than at day 1 and four and eight weeks after return to LL, p<0.01. (C) Gill Na^+^, K^+^- ATPase activity; data are mean ± SEM of n = 6–10 fish per sampling point. *, **, significantly higher activity than at day 1 of the experiment, p<0.05, 0.01, respectively. Where error bars do not appear, errors lie within the symbol. The dashed line represents the SP control group.

Experiment 2 is summarized in Fig 3A. To further characterize the apparent requirement for exposure to SP for induction of expression of the cluster 3 genes, juvenile salmon were exposed to two, four or eight weeks of SP, before being returned to LL (2WSP, 4WSP, 8WSP, respectively), and their short- and long-term SW-tolerance and gene expression were assessed. Complete information on growth and CF during the FW and SW stays can be found in S5 Table.

#### Hypo-osmoregulatory capacity

The ability to hypo-osmoregulate during a 24-h SW challenge was not dependent upon prior exposure to SP, but rather time spent after re-entering LL (p<0.0001, for main effect of time, by two-way ANOVA, Fig 3B, S2 Table). As in Experiment 1, the fish were able to hypo-osmoregulating efficiently on day 1 of the experiment, and this ability was lost within two weeks of transfer to SP, as evidenced by the increased levels of plasma osmolytes (p<0.0001, one-way ANOVA). The dynamics of re-establishment of hypo-osmoregulatory capacity following return to LL did not differ between the SP-LL groups, which developed smolt-like hypo-osmoregulatory capacity within four weeks of re-entering LL. The SPC group spontaneously regained its ability to osmoregulate towards day 86 after having spent more than 12 weeks under SP. Though slightly higher levels were measured on day 113, plasma osmolality levels of the SPC group were not statistically different from those measured on day 1, nor at the end-points of the SP-LL groups. Chloride plasma levels followed a similar pattern as described for plasma osmolytes.

#### NKA activity

Because gill NKA activity is considered a good indicator of osmoregulatory capacity and smolt status we also examined how this trait was influenced by photoperiodic history (Fig 3C). In contrast to plasma osmolality and chloride levels, gill NKA activity did not change significantly under chronic exposure to SP (SPC group). However, the development of NKA activity following return of fish to LL was highly dependent on photoperiodic history (p<0.001, for time x photoperiod regime, by two-way ANOVA, supplemental material S3 Table). In fish exposed to SP for two weeks no significant rise in gill NKA activity was seen during the subsequent eight weeks of LL exposure, while in the 8WSP group NKA activity rose approximately five-fold over eight weeks of LL exposure (p<0.001, two-way ANOVA). In 4WSP fish, an intermediate response was observed, with NKA activity rising some two-fold over the post-SP phase. Gill NKA activity does not appear do predict performance in 24-h SW challenges.

#### RNA profiling of gill tissue

**Fig 4:**
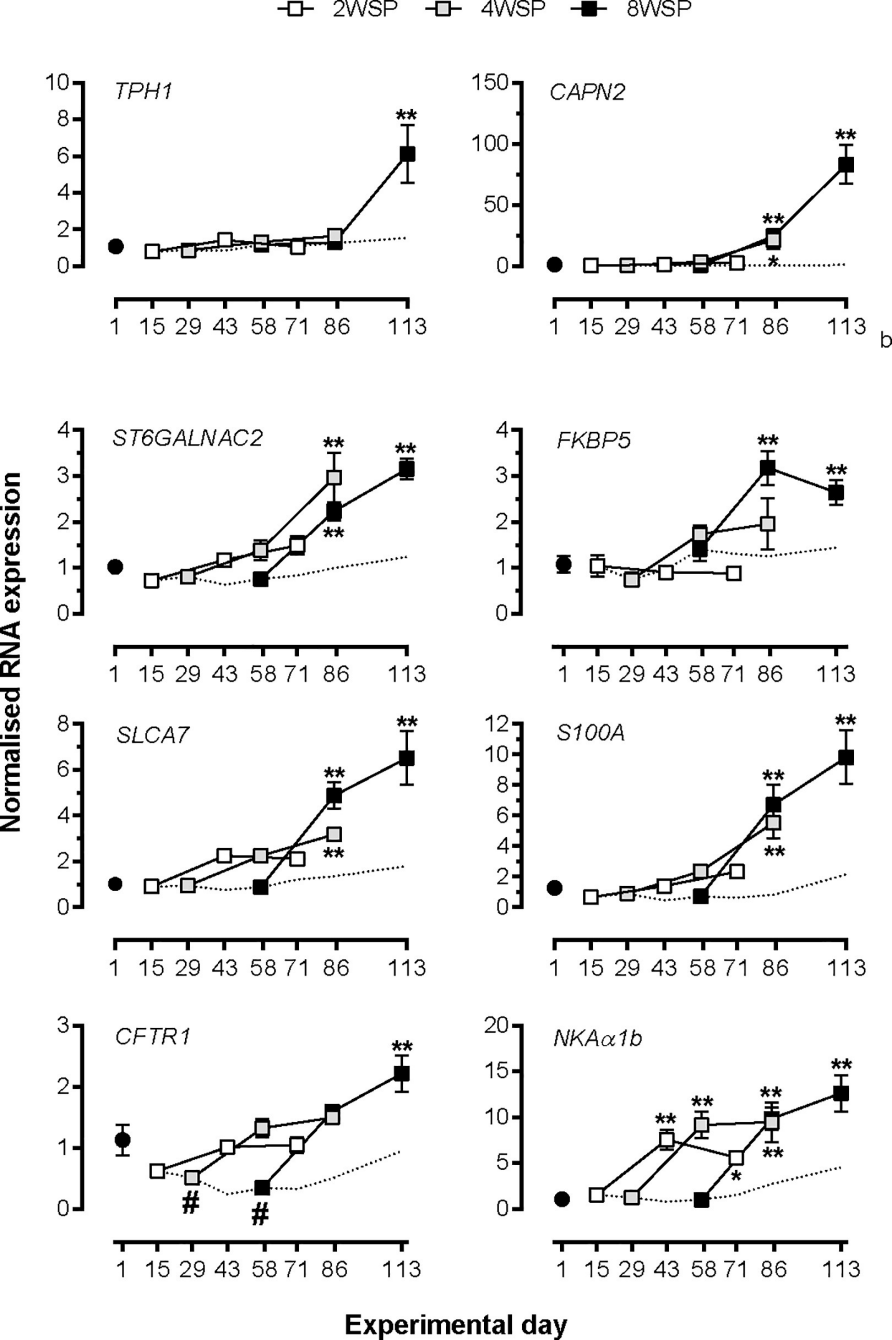
qPCR profiling of effect of SP exposure duration on selected cluster 3 genes. Data are normalized mRNA abundance, mean ± SEM of n = 6 fish per sampling point. *, ** significantly higher expression than LL and SP values at the corresponding time point, p<0.05, 0.01, respectively; # significantly lower expression than at day 1, p<0.05. Where error bars do not appear, errors lie within the symbol. The dashed line represents the SP control group.

We used qPCR to assess the expression of the six novel transcripts selected from Experiment 2, and of *CFTR I* and *NKA α1b* (Fig 4). For each of the novel cluster 3 transcripts induction of expression by exposure to LL was highly dependent on the duration of prior SP exposure (p<0.001, time x photoperiod regime interaction, by two-way ANOVA, S7 Table), with the strongest induction of expression consistently being observed in the 8WSP group (p<0.001, one-way ANOVA), with the exception of *ST6GALNAC2*. No significant increases above day 1 expression levels was seen for the 2WSP group at any point following return to LL, while in the 4WSP group one could observe intermediate increases, with significant differences to pre-SP levels for *S100A1*, *ST6GALNAC2*, *SLC5A7* and *CAPN2* (p<0.05, one-way ANOVA).

A clear dependence on photoperiodic history was also observed for *CFTR I* (p<0.0001, time x photoperiod regime, two-way ANOVA, S7 Table), with insignificant changes in 2WSP and an intermediate response in 4WSP, and a very significant increase in 8WSP (p<0.0001, one-way ANOVA) following the SP-LL transition. Contrastingly, the induction of *NKA α1b* expression by re-entering LL was not dependent on photoperiodic history, with all three SP-LL groups showing elevated (and equal) mRNA levels, compared with pre-SP levels, after four weeks of LL exposure (p>0.001, one-way ANOVA).

#### Growth performance during extended exposure to SW

In order to assess long-term SW performance of the presumed smolts from each of the SP-LL photoperiod regimes fish from each of the SP-LL groups were transferred to SW tanks for 28 days. Initial weights at the point of transfer to SW did not differ significantly between the groups (Table 2), and there were no differences in CF. Contrastingly, subsequent SW growth performance was highly dependent on prior exposure to short photoperiod (p = 0.003 for time x photoperiod regime interaction, two-way ANOVA, S5 Table). During the four weeks in SW, fish transferred from the 2WSP group showed no significant increase in body mass, while over the same period body masse increased in the 4WSP and 8WSP groups by 19.6% and 27.3%, respectively. Moreover, fish in the 8WSP group grew significantly more, and at a higher SGR (Table 2) than fish from the 4WSP group (P<0.01 for final weight comparison). Total dry weight feed intake was 860 g, 944 g, and 1277 g in the 2WSP, 4WSP, and 8WSP treatment groups, respectively, leading to FRCs of 8.27, 2.31 and 2.11 (Table 2).

**Table 2:** Information on age and weight of fish during the prolonged SW stay. Significance as determined by two-way ANOVA. SGR -Specific growth rate, FCR -Feed conversion ratio.

	AGE (weeks)		BIOMASS (g)						
Treatment	SW entry	SW end	SW entry	SW mid-phase	SW end	Increase (%)	p-value	SGR	FCR
2WSP	56	60	67.2±10.9	68.8±11.6	70.6±11.4	5.1	n.s.	0.169	8.27
4WSP	58	62	69.4±8.7	73.8±10.2	83.0±12.5	19.6	< 0.001	0.586	2.31
8WSP	62	66	73.9±11.8	80.8±12.5	94.1±15.1	27. 3	<0.0001	0.839	2.11

In addition to differences in food intake, and weight gain, moderate changes in CF were observed (S5 Table). Upon transfer to SW the fish in the 8WSP group had a significantly lower CF than those in the 2WSP group (p<0.05), while an intermediate value was seen in the 4WSP fish. Only the fish in the 2WSP group showed a significant decrease in CF over the four weeks in SW (p<0.001).

## Discussion

Successful smolting involves the coordination of developmental and physiological processes to produce a SW-ready smolt phenotype. Confirming previous studies, we find that the successful coordination of smolting is dependent upon photoperiodic history [6, 42, 43]. Further, we have identified a cohort of genes, previously unstudied in the context of smolting, whose expression in the gills is highly history-dependent. We show that juvenile salmon exposed to four or fewer weeks of SP, followed by LL, maintain low levels of plasma osmolytes during 24-h SWCs, while experiencing poor growth during extended SW exposure. We suggest that the novel genes presented here could act as markers for SW preparedness in smolts. Further exploration of these genes would improve our understanding of the physiological and endocrine regulation of gill differentiation during smolting, and how it is controlled by photoperiod.

In both experiments, juvenile fish raised on LL were able to maintain osmotic balance during 24-h exposure to SW. Similar hypo-osmoregulatory ability has previously been observed under similar conditions [44, 45], and was attributed to a spontaneous development of salinity tolerance after exceeding a minimum body size threshold. In the present study, SP exposure suppressed salinity tolerance in all groups. Under prolonged exposure to SP, the hypo-osmoregulatory capacity spontaneously recovers, but not to the same extent as in fish that are returned to LL. The physiological reasons for this partial recovery are unknown, but could be due to endogenous processes influencing the MRCs [46], or by an improved capacity to handle osmotic stress due to increased size [47]. Regardless of photoperiodic history, LL is a strong stimulus for recovery of the capacity to hypo-osmoregulate during a 24-h SW challenge. This apparent lack of history-dependence in the response to a short-term SW challenge is consistent with previous reports [6, 42], but gives no indication of how the osmoregulatory mechanisms involved in maintaining ionic balance change during the developmental process of smolting. Based on the impaired SW growth rate of fish exposed to SP for 2 and 4 weeks prior to LL exposure, it appears that smolting entails a development of energetically efficient mechanisms for maintaining ionic balance through a process which is dependent on photoperiodic history. The reacquisition of the ability to hypo-osmoregulate during 24-h SW challenges under prolonged SP exposure could be a result of free-running, endogenous rhythms [6, 46]. A common feature of the 2WSP, 4WSP and SPC groups in Experiment 2 was the unresponsiveness of the NKA activity and in the mRNA expression of five of our six novel genes. A similar decoupling of NKA activity and hypo-osmoregulatory capacity has been observed by Berge, Berg [48] and Handeland and Stefansson [49], indicating that increased NKA activity is not a prerequisite for (short-term) salinity tolerance.

The extended SW exposure in Experiment 2 show reduced growth for the 2WSP and 4WSP, and particularly in the case of the 2WSP, a very high FCR. This indicates that growth and FCR in SW is influenced by photoperiodic history through its control of hypo-osmoregulatory capacity. We suggest that the lack of hypo-osmoregulatory capacity, especially in the 2WSP group, causes a higher energy demand leading to reduced growth. Similar observation were made by Saunders, Henderson [43], whom found that juvenile salmon exposed to LL grew better than juveniles exposed to a natural photoperiod in FW, but in SW these grew substantially less than those exposed to natural photoperiod.

One potential caveat of our experimental design is the age difference arising between treatment groups in experiment 2. However, the juvenile salmon in experiment 1 responded with increased mRNA expression for all the novel genes at four months younger, so we do not believe that age *per se* is an underlying cause for observed differences in long-term SW tolerance. Rather, the time spent on SP prior to LL exposure leads to history-dependent effects on gene expression, NKA activity and long-term SW performance.

Smolting is a hormonally controlled process and photoperiod-dependent changes in the secretion of anterior pituitary trophic hormones (ACTH, GH and TSH), together with cortisol and IGF-1, have been reported [2, 15, 19, 50, 51]. Importantly, the same hormones are not influenced by increased day length in salmon juveniles below a threshold body size for smolting [2, 4, 52, 53]. Thus, several authors have suggested that a central hormonal mechanisms controlling the ‘decision’ to smolt are connected to growth and energetic status [1, 3, 54–57]. Such conditional activation is known to be a key feature of life history transitions where seasonal timing is of paramount importance [58, 59]. Such central hormonal systems may also undergo innately timed changes to control the expression of seasonal responses, even when external stimuli has been inadequate [60–64].

Photoperiodic history-dependence might also be an innate property of autonomous timers in peripheral tissues, expressed as inertia in responses to hormonal signals [65, 66]. An indication of inertia in gill tissue is observed in a paper by McCormick, Björnsson [67], where advancing the phase of the spring increase in photoperiod causes a corresponding advance in pituitary GH secretion, but not in gill NKA activity. Expression of *NKA α1b* has previously been linked with increased plasma GH and NKA activity [2, 23], however, Christensen, Regish [68] emphasize the role of NKA α1b protein rather than mRNA for NKA activity and SW tolerance. The dichotomy in photoperiodic history-dependence between *NKA* α1b mRNA expression and NKA activity in the present study, together with the aforementioned results, point to post-translational mechanisms influencing NKA activity, rather than an effect of increased NKA α1b expression. Other NKA pump components such as the NKA β subunit and FXYD proteins could be significant contributors to the stabilization and function of the NKA pump [23, 69–71]. Further examination of photoperiodic history-dependence of gill gene expression is warranted to resolve between centrally controlled processes and peripheral mechanisms.

The genes in cluster 3 can be linked to cytoskeletal function, G-protein coupled receptor signaling, ion uptake and excretion, epidermal structure and cell adhesion. TPH1 is the rate limiting enzyme for serotonin (5HT) synthesis, serotonin is known to have vasoconstrictory effects in the gill [72], and evidence shows that serotonylation of histones can influence gene transcription [73]. Whereas the latter could be significant in terms of changes to the transcriptome, the vasoconstriction will influence blood pressure in the gill and potentially redistribute the blood flow, influencing the exchange of molecules with the environment [74]. CAPN2’s role in cytoskeletal remodeling and cell motility [75] is also of interest given the extensive tubular network found in SW-ready MRCs [12, 14], and potential migration of developing MRCs from the base of the filament [76, 77]. FKBP5 can be linked to the glucocorticoid receptor regulatory network [78–80] which is known to be involved in SW acclimation [81, 82]. The glucocorticoid receptors have previously been associated with regulation of CFTR I and the NKA α-subunits in salmon [19, 83]. S100A is a calcium binding protein, implicated in the regulation of many cellular processes, including differentiation [84]. After binding with Ca^2+^, the S100A undergoes a conformational change, allowing it to interact with a wide variety of targets, such as cytoskeletal proteins and transcription factors [84]. SLC5A7 is an sodium/substrate symporter known to mediate choline uptake in humans [85]. Cholinergic cells have been described in the gill of zebrafish (*Danio rerio*), and it is proposed that they could be significant in ventilatory control [86]. ST6GALNAC2 is a sialyltransferase, influencing cell-cell and cell-substrate interactions [87]. This breadth of potential actions of cluster 3 genes underlines the extent to which successful smolting relies upon comprehensive re-organisation of gill function, reflecting the pleiotropic role of this tissue in many essential aspects of salmonid physiology. It remains to disclose the true function of these genes in a gill specific context, with further studies of cellular localization and protein function now being required.

In conclusion, commonly used predictors of SW readiness in smolts (osmoregulation, NKA activity) appear to be inadequate when it comes to predicting actual SW performance. A biomolecular approach, simultaneously measuring mRNA levels of several confirmed marker genes potentially offers a better prediction of SW performance. Additionally, it appears that such markers exhibit a strong dependence on photoperiodic history, emphasizing the role of SP for smolt development.

## Supporting information

S1 Checklist The ARRIVE guidelines checklist. (PDF)

S1 Table Experiment 1 Osmolality, 2-way ANOVA. Table showing the 2-way ANOVA and multiple comparison results for the plasma osmolality measurements in experiment 1.
(PDF)

S2 Table Experiment 2 Osmolality, 2-way ANOVA. Table showing the 2-way ANOVA and multiple comparison results for the plasma osmolality measurements in experiment 2.
(PDF)

S3 Table Experiment 2 Na^+^, K^+^- ATPase activity, 2-way ANOVA. Table showing the 2-way ANOVA and multiple comparison results for the gill Na^+^, K^+^- ATPase activity measurements in experiment 2.
(PDF)

S4 Table Experiment 1 weight and condition factor. Table shows the development of weight (g) and conditionu factor of the experimental groups in experiment 1.
(PDF)

S5 Table Experiment 2 weight and condition factor FW. Table shows the development of weight (g) and condition factor of the experimental groups during the FW phase of experiment 2.
(PDF)

S6 Table Experiment 1 Gene expression, 2-way ANOVA. Tables showing the 2-way ANOVA and multiple comparison results for the expression of the genes measured in experiment 1 and shown in Fig 2.
(PDF)

S7 Table Experiment 2 Gene expression, 2-way ANOVA. Tables showing the 2-way ANOVA and multiple comparison results for the expression of the genes measured in experiment 2 and shown in Fig 4.
(PDF)

S8 Table Heatmap data. Data table with z-scores, cluster number and information on each of the genes in the heatmap presented in Fig 1.
(XLSX)

S1 Fig Plasma chloride. Graphs showing the averaged levels (±SEM) of plasma chloride (mmol l^-1^) in the treatments groups of experiment 1 (top, n = 6) and experiment 2 (bottom, n = 10, dotted line represents the SPC group), measured after 24h SWCs.
(PDF)
