# Correction: RNA profiling identifies novel, photoperiod-history dependent markers associated with enhanced saltwater performance in juvenile Atlantic salmon

**DOI:** 10.1371/journal.pone.0237623

**Published:** 2020-08-07

**Authors:** Marianne Iversen, Teshome Mulugeta, Børge Gellein Blikeng, Alexander Christopher West, Even Hjalmar Jørgensen, Simen Rød Sandve, David Hazlerigg

The sixth author's name is spelled incorrectly. The correct name is: Simen Rød Sandve. The correct citation is: Iversen M, Mulugeta T, Gellein Blikeng B, West AC, Jørgensen EH, Rød Sandve S, et al. (2020) RNA profiling identifies novel, photoperiod-history dependent markers associated with enhanced saltwater performance in juvenile Atlantic salmon. PLoS ONE 15(4): e0227496. https://doi.org/10.1371/journal.pone.0227496
