# Supplementary material for: RNA profiling identifies novel, photoperiod-history dependent markers associated with enhanced saltwater performance in juvenile Atlantic salmon
Source: PLoS One. 2020 Apr 8;15(4):e0227496. doi: 10.1371/journal.pone.0227496 (PMC7141700; doi:10.1371/journal.pone.0227496)
Supplement: S1 Table — Table showing the 2-way ANOVA and multiple comparison results for the plasma osmolality measurements in experiment 1. (PDF) [file pone.0227496.s002.pdf]

2way ANOVA
Tabular results

1	Table Analyzed	Osmolality 2013				
2						
3	Two-way ANOVA	Ordinary				
4	Alpha	0.05				
5						
6	Source of Variation	% of total variation	P value	P value summary	Significant?	
7	Interaction	19.58	<0.0001	****	Yes	
8	Time	29.14	<0.0001	****	Yes	
9	Treatment	35.89	<0.0001	****	Yes	
10						
11	ANOVA table	SS (Type III)	DF	MS	F (DFn, DFd)	P value
12	Interaction	11380	4	2845	F (4, 44) = 14.89	P<0.0001
13	Time	16933	2	8466	F (2, 44) = 44.31	P<0.0001
14	Treatment	20858	2	10429	F (2, 44) = 54.58	P<0.0001
15	Residual	8407	44	191.1		
16						
17	Number of missing values	1				

2way ANOVA
Multiple comparisons

1	Within each row, compare columns (simple effects within rows)								
2									
3	Number of families	3							
4	Number of comparisons per family	3							
5	Alpha	0.05							
6									
7	Tukey's multiple comparisons test	Mean Diff.	95.00% CI of diff.	Significant?	Summary	Adjusted P Value			
8									
9	68								
10	LL vs. SP	-73	-92.36 to -53.64	Yes	****	<0.0001			
11	LL vs. SPLL	-62.67	-82.02 to -43.31	Yes	****	<0.0001			
12	SP vs. SPLL	10.33	-9.023 to 29.69	No	ns	0.4056			
13									
14	89								
15	LL vs. SP	-32.9	-53.2 to -12.6	Yes	***	0.0008			
16	LL vs. SPLL	10.5	-8.857 to 29.86	No	ns	0.3942			
17	SP vs. SPLL	43.4	23.1 to 63.7	Yes	****	<0.0001			
18									
19	110								
20	LL vs. SP	-36.33	-55.69 to -16.98	Yes	***	0.0001			
21	LL vs. SPLL	14.5	-4.857 to 33.86	No	ns	0.1759			
22	SP vs. SPLL	50.83	31.48 to 70.19	Yes	****	<0.0001			
23									
24									
25	Test details	Mean 1	Mean 2	Mean Diff.	SE of diff.	N1	N2	q	DF
26									
27	68								
28	LL vs. SP	347	420	-73	7.981	6	6	12.94	44
29	LL vs. SPLL	347	409.7	-62.67	7.981	6	6	11.1	44
30	SP vs. SPLL	420	409.7	10.33	7.981	6	6	1.831	44

**2way ANOVA**<br>Multiple comparisons									
31									
32	89								
33	LL vs. SP	354.5	387.4	-32.9	8.37	6	5	5.559	44
34	LL vs. SPLL	354.5	344	10.5	7.981	6	6	1.861	44
35	SP vs. SPLL	387.4	344	43.4	8.37	5	6	7.333	44
36									
37	110								
38	LL vs. SP	342.8	379.2	-36.33	7.981	6	6	6.439	44
39	LL vs. SPLL	342.8	328.3	14.5	7.981	6	6	2.569	44
40	SP vs. SPLL	379.2	328.3	50.83	7.981	6	6	9.008	44
