# Supplementary material for: RNA profiling identifies novel, photoperiod-history dependent markers associated with enhanced saltwater performance in juvenile Atlantic salmon
Source: PLoS One. 2020 Apr 8;15(4):e0227496. doi: 10.1371/journal.pone.0227496 (PMC7141700; doi:10.1371/journal.pone.0227496)
Supplement: S2 Table — Table showing the 2-way ANOVA and multiple comparison results for the plasma osmolality measurements in experiment 2. (PDF) [file pone.0227496.s003.pdf]

2way ANOVA
Tabular results

1	Table Analyzed	Osmolality 2-ANOVA				
2						
3	Two-way ANOVA	Ordinary				
4	Alpha	0.05				
5						
6	Source of Variation	% of total variation	P value	P value summary	Significant?	
7	Interaction	13.08	<0.0001	****	Yes	
8	Treatment	61.28	<0.0001	****	Yes	
9	Pretreatment	4.933	<0.0001	****	Yes	
10						
11	ANOVA table	SS (Type III)	DF	MS	F (DFn, DFd)	P value
12	Interaction	31215	8	3902	F (8, 131) = 9.62	P<0.0001
13	Treatment	146229	4	36557	F (4, 131) = 90.13	P<0.0001
14	Pretreatment	11772	2	5886	F (2, 131) = 14.51	P<0.0001
15	Residual	53137	131	405.6		
16						
17	Number of missing values	4				

2way ANOVA
Multiple comparisons

1	Compare cell means regardless of rows and columns						
2							
3	Number of families	1					
4	Number of comparisons per family	105					
5	Alpha	0.05					
6							
7	Tukey's multiple comparisons test	Mean Diff.	95.00% CI of diff.	Significant?	Summary	Adjusted P Value	
8							
9	Last day SP:2 week winter vs. Last day SP:4 week winter	-5.25	-36.39 to 25.89	No	ns	>0.9999	
10	Last day SP:2 week winter vs. Last day SP:8 week winter	-8.15	-39.29 to 22.99	No	ns	0.9999	
11	Last day SP:2 week winter vs. 4 weeks post winter:2 week winter	58.03	26.04 to 90.02	Yes	****	<0.0001	
12	Last day SP:2 week winter vs. 4 weeks post winter:4 week winter	40.02	8.878 to 71.16	Yes	**	0.0017	
13	Last day SP:2 week winter vs. 4 weeks post winter:8 week winter	42.1	10.96 to 73.24	Yes	***	0.0007	
14	Last day SP:2 week winter vs. 8 weeks post winter:2 week winter	65.28	33.29 to 97.27	Yes	****	<0.0001	
15	Last day SP:2 week winter vs. 8 weeks post winter:4 week winter	62.18	31.04 to 93.31	Yes	****	<0.0001	
16	Last day SP:2 week winter vs. 8 weeks post winter:8 week winter	65.55	34.41 to 96.69	Yes	****	<0.0001	
17	Last day SP:2 week winter vs. SPC -4 weeks post:2 week winter	-11.64	-43.63 to 20.35	No	ns	0.9950	
18	Last day SP:2 week winter vs. SPC -4 weeks post:4 week winter	-64.42	-96.41 to -32.42	Yes	****	<0.0001	
19	Last day SP:2 week winter vs. SPC -4 weeks post:8 week winter	9.4	-21.74 to 40.54	No	ns	0.9993	
20	Last day SP:2 week winter vs. SPC -8 weeks post:2 week winter	-8.5	-39.64 to 22.64	No	ns	0.9998	
21	Last day SP:2 week winter vs. SPC -8 weeks post:4 week winter	9.4	-21.74 to 40.54	No	ns	0.9993	
22	Last day SP:2 week winter vs. SPC -8 weeks post:8 week winter	41.9	10.76 to 73.04	Yes	***	0.0007	
23	Last day SP:4 week winter vs. Last day SP:8 week winter	-2.9	-34.04 to 28.24	No	ns	>0.9999	
24	Last day SP:4 week winter vs. 4 weeks post winter:2 week winter	63.28	31.29 to 95.27	Yes	****	<0.0001	
25	Last day SP:4 week winter vs. 4 weeks post winter:4 week winter	45.27	14.13 to 76.41	Yes	***	0.0002	
26	Last day SP:4 week winter vs. 4 weeks post winter:8 week winter	47.35	16.21 to 78.49	Yes	****	<0.0001	
27	Last day SP:4 week winter vs. 8 weeks post winter:2 week winter	70.53	38.54 to 102.5	Yes	****	<0.0001	
28	Last day SP:4 week winter vs. 8 weeks post winter:4 week winter	67.43	36.29 to 98.56	Yes	****	<0.0001	
29	Last day SP:4 week winter vs. 8 weeks post winter:8 week winter	70.8	39.66 to 101.9	Yes	****	<0.0001	
30	Last day SP:4 week winter vs. SPC -4 weeks post:2 week winter	-6.389	-38.38 to 25.6	No	ns	>0.9999	

	2way ANOVA<br>Multiple comparisons						
31	Last day SP:4 week winter vs. SPC -4 weeks post:4 week winter	-59.17	-91.16 to -27.17	Yes	****	<0.0001	
32	Last day SP:4 week winter vs. SPC -4 weeks post:8 week winter	14.65	-16.49 to 45.79	No	ns	0.9497	
33	Last day SP:4 week winter vs. SPC -8 weeks post:2 week winter	-3.25	-34.39 to 27.89	No	ns	>0.9999	
34	Last day SP:4 week winter vs. SPC -8 weeks post:4 week winter	14.65	-16.49 to 45.79	No	ns	0.9497	
35	Last day SP:4 week winter vs. SPC -8 weeks post:8 week winter	47.15	16.01 to 78.29	Yes	****	<0.0001	
36	Last day SP:8 week winter vs. 4 weeks post winter:2 week winter	66.18	34.19 to 98.17	Yes	****	<0.0001	
37	Last day SP:8 week winter vs. 4 weeks post winter:4 week winter	48.17	17.03 to 79.31	Yes	****	<0.0001	
38	Last day SP:8 week winter vs. 4 weeks post winter:8 week winter	50.25	19.11 to 81.39	Yes	****	<0.0001	
39	Last day SP:8 week winter vs. 8 weeks post winter:2 week winter	73.43	41.44 to 105.4	Yes	****	<0.0001	
40	Last day SP:8 week winter vs. 8 weeks post winter:4 week winter	70.33	39.19 to 101.5	Yes	****	<0.0001	
41	Last day SP:8 week winter vs. 8 weeks post winter:8 week winter	73.7	42.56 to 104.8	Yes	****	<0.0001	
42	Last day SP:8 week winter vs. SPC -4 weeks post:2 week winter	-3.489	-35.48 to 28.5	No	ns	>0.9999	
43	Last day SP:8 week winter vs. SPC -4 weeks post:4 week winter	-56.27	-88.26 to -24.27	Yes	****	<0.0001	
44	Last day SP:8 week winter vs. SPC -4 weeks post:8 week winter	17.55	-13.59 to 48.69	No	ns	0.8237	
45	Last day SP:8 week winter vs. SPC -8 weeks post:2 week winter	-0.35	-31.49 to 30.79	No	ns	>0.9999	
46	Last day SP:8 week winter vs. SPC -8 weeks post:4 week winter	17.55	-13.59 to 48.69	No	ns	0.8237	
47	Last day SP:8 week winter vs. SPC -8 weeks post:8 week winter	50.05	18.91 to 81.19	Yes	****	<0.0001	
48	4 weeks post winter:2 week winter vs. 4 weeks post winter:4 week winter	-18.01	-50 to 13.98	No	ns	0.8248	
49	4 weeks post winter:2 week winter vs. 4 weeks post winter:8 week winter	-15.93	-47.92 to 16.06	No	ns	0.9227	
50	4 weeks post winter:2 week winter vs. 8 weeks post winter:2 week winter	7.25	-25.57 to 40.07	No	ns	>0.9999	
51	4 weeks post winter:2 week winter vs. 8 weeks post winter:4 week winter	4.147	-27.85 to 36.14	No	ns	>0.9999	
52	4 weeks post winter:2 week winter vs. 8 weeks post winter:8 week winter	7.522	-24.47 to 39.51	No	ns	>0.9999	
53	4 weeks post winter:2 week winter vs. SPC -4 weeks post:2 week winter	-69.67	-102.5 to -36.84	Yes	****	<0.0001	
54	4 weeks post winter:2 week winter vs. SPC -4 weeks post:4 week winter	-122.4	-155.3 to -89.62	Yes	****	<0.0001	
55	4 weeks post winter:2 week winter vs. SPC -4 weeks post:8 week winter	-48.63	-80.62 to -16.64	Yes	****	<0.0001	
56	4 weeks post winter:2 week winter vs. SPC -8 weeks post:2 week winter	-66.53	-98.52 to -34.54	Yes	****	<0.0001	
57	4 weeks post winter:2 week winter vs. SPC -8 weeks post:4 week winter	-48.63	-80.62 to -16.64	Yes	****	<0.0001	
58	4 weeks post winter:2 week winter vs. SPC -8 weeks post:8 week winter	-16.13	-48.12 to 15.86	No	ns	0.9154	
59	4 weeks post winter:4 week winter vs. 4 weeks post winter:8 week winter	2.083	-29.06 to 33.22	No	ns	>0.9999	
60	4 weeks post winter:4 week winter vs. 8 weeks post winter:2 week winter	25.26	-6.731 to 57.25	No	ns	0.2993	

2way ANOVA<br>Multiple comparisons							
61	4 weeks post winter:4 week winter vs. 8 weeks post winter:4 week winter	22.16	-8.981 to 53.3	No	ns	0.4761	
62	4 weeks post winter:4 week winter vs. 8 weeks post winter:8 week winter	25.53	-5.606 to 56.67	No	ns	0.2420	
63	4 weeks post winter:4 week winter vs. SPC -4 weeks post:2 week winter	-51.66	-83.65 to -19.66	Yes	****	<0.0001	
64	4 weeks post winter:4 week winter vs. SPC -4 weeks post:4 week winter	-104.4	-136.4 to -72.44	Yes	****	<0.0001	
65	4 weeks post winter:4 week winter vs. SPC -4 weeks post:8 week winter	-30.62	-61.76 to 0.5224	No	ns	0.0591	
66	4 weeks post winter:4 week winter vs. SPC -8 weeks post:2 week winter	-48.52	-79.66 to -17.38	Yes	****	<0.0001	
67	4 weeks post winter:4 week winter vs. SPC -8 weeks post:4 week winter	-30.62	-61.76 to 0.5224	No	ns	0.0591	
68	4 weeks post winter:4 week winter vs. SPC -8 weeks post:8 week winter	1.883	-29.26 to 33.02	No	ns	>0.9999	
69	4 weeks post winter:8 week winter vs. 8 weeks post winter:2 week winter	23.18	-8.815 to 55.17	No	ns	0.4446	
70	4 weeks post winter:8 week winter vs. 8 weeks post winter:4 week winter	20.08	-11.06 to 51.21	No	ns	0.6438	
71	4 weeks post winter:8 week winter vs. 8 weeks post winter:8 week winter	23.45	-7.689 to 54.59	No	ns	0.3775	
72	4 weeks post winter:8 week winter vs. SPC -4 weeks post:2 week winter	-53.74	-85.73 to -21.75	Yes	****	<0.0001	
73	4 weeks post winter:8 week winter vs. SPC -4 weeks post:4 week winter	-106.5	-138.5 to -74.52	Yes	****	<0.0001	
74	4 weeks post winter:8 week winter vs. SPC -4 weeks post:8 week winter	-32.7	-63.84 to -1.561	Yes	*	0.0296	
75	4 weeks post winter:8 week winter vs. SPC -8 weeks post:2 week winter	-50.6	-81.74 to -19.46	Yes	****	<0.0001	
76	4 weeks post winter:8 week winter vs. SPC -8 weeks post:4 week winter	-32.7	-63.84 to -1.561	Yes	*	0.0296	
77	4 weeks post winter:8 week winter vs. SPC -8 weeks post:8 week winter	-0.2	-31.34 to 30.94	No	ns	>0.9999	
78	8 weeks post winter:2 week winter vs. 8 weeks post winter:4 week winter	-3.103	-35.1 to 28.89	No	ns	>0.9999	
79	8 weeks post winter:2 week winter vs. 8 weeks post winter:8 week winter	0.2722	-31.72 to 32.26	No	ns	>0.9999	
80	8 weeks post winter:2 week winter vs. SPC -4 weeks post:2 week winter	-76.92	-109.7 to -44.09	Yes	****	<0.0001	
81	8 weeks post winter:2 week winter vs. SPC -4 weeks post:4 week winter	-129.7	-162.5 to -96.87	Yes	****	<0.0001	
82	8 weeks post winter:2 week winter vs. SPC -4 weeks post:8 week winter	-55.88	-87.87 to -23.89	Yes	****	<0.0001	
83	8 weeks post winter:2 week winter vs. SPC -8 weeks post:2 week winter	-73.78	-105.8 to -41.79	Yes	****	<0.0001	
84	8 weeks post winter:2 week winter vs. SPC -8 weeks post:4 week winter	-55.88	-87.87 to -23.89	Yes	****	<0.0001	
85	8 weeks post winter:2 week winter vs. SPC -8 weeks post:8 week winter	-23.38	-55.37 to 8.615	No	ns	0.4295	
86	8 weeks post winter:4 week winter vs. 8 weeks post winter:8 week winter	3.375	-27.76 to 34.51	No	ns	>0.9999	
87	8 weeks post winter:4 week winter vs. SPC -4 weeks post:2 week winter	-73.81	-105.8 to -41.82	Yes	****	<0.0001	
88	8 weeks post winter:4 week winter vs. SPC -4 weeks post:4 week winter	-126.6	-158.6 to -94.6	Yes	****	<0.0001	
89	8 weeks post winter:4 week winter vs. SPC -4 weeks post:8 week winter	-52.78	-83.91 to -21.64	Yes	****	<0.0001	
90	8 weeks post winter:4 week winter vs. SPC -8 weeks post:2 week winter	-70.68	-101.8 to -39.54	Yes	****	<0.0001	

	2way ANOVA<br>Multiple comparisons						
91	8 weeks post winter:4 week winter vs. SPC -8 weeks post:4 week winter	-52.78	-83.91 to -21.64	Yes	****	<0.0001	
92	8 weeks post winter:4 week winter vs. SPC -8 weeks post:8 week winter	-20.28	-51.41 to 10.86	No	ns	0.6279	
93	8 weeks post winter:8 week winter vs. SPC -4 weeks post:2 week winter	-77.19	-109.2 to -45.2	Yes	****	<0.0001	
94	8 weeks post winter:8 week winter vs. SPC -4 weeks post:4 week winter	-130	-162 to -97.97	Yes	****	<0.0001	
95	8 weeks post winter:8 week winter vs. SPC -4 weeks post:8 week winter	-56.15	-87.29 to -25.01	Yes	****	<0.0001	
96	8 weeks post winter:8 week winter vs. SPC -8 weeks post:2 week winter	-74.05	-105.2 to -42.91	Yes	****	<0.0001	
97	8 weeks post winter:8 week winter vs. SPC -8 weeks post:4 week winter	-56.15	-87.29 to -25.01	Yes	****	<0.0001	
98	8 weeks post winter:8 week winter vs. SPC -8 weeks post:8 week winter	-23.65	-54.79 to 7.489	No	ns	0.3630	
99	SPC -4 weeks post:2 week winter vs. SPC -4 weeks post:4 week winter	-52.78	-85.6 to -19.95	Yes	****	<0.0001	
100	SPC -4 weeks post:2 week winter vs. SPC -4 weeks post:8 week winter	21.04	-10.95 to 53.03	No	ns	0.6116	
101	SPC -4 weeks post:2 week winter vs. SPC -8 weeks post:2 week winter	3.139	-28.85 to 35.13	No	ns	>0.9999	
102	SPC -4 weeks post:2 week winter vs. SPC -8 weeks post:4 week winter	21.04	-10.95 to 53.03	No	ns	0.6116	
103	SPC -4 weeks post:2 week winter vs. SPC -8 weeks post:8 week winter	53.54	21.55 to 85.53	Yes	****	<0.0001	
104	SPC -4 weeks post:4 week winter vs. SPC -4 weeks post:8 week winter	73.82	41.82 to 105.8	Yes	****	<0.0001	
105	SPC -4 weeks post:4 week winter vs. SPC -8 weeks post:2 week winter	55.92	23.92 to 87.91	Yes	****	<0.0001	
106	SPC -4 weeks post:4 week winter vs. SPC -8 weeks post:4 week winter	73.82	41.82 to 105.8	Yes	****	<0.0001	
107	SPC -4 weeks post:4 week winter vs. SPC -8 weeks post:8 week winter	106.3	74.32 to 138.3	Yes	****	<0.0001	
108	SPC -4 weeks post:8 week winter vs. SPC -8 weeks post:2 week winter	-17.9	-49.04 to 13.24	No	ns	0.8020	
109	SPC -4 weeks post:8 week winter vs. SPC -8 weeks post:4 week winter	0	-31.14 to 31.14	No	ns	>0.9999	
110	SPC -4 weeks post:8 week winter vs. SPC -8 weeks post:8 week winter	32.5	1.361 to 63.64	Yes	*	0.0317	
111	SPC -8 weeks post:2 week winter vs. SPC -8 weeks post:4 week winter	17.9	-13.24 to 49.04	No	ns	0.8020	
112	SPC -8 weeks post:2 week winter vs. SPC -8 weeks post:8 week winter	50.4	19.26 to 81.54	Yes	****	<0.0001	
113	SPC -8 weeks post:4 week winter vs. SPC -8 weeks post:8 week winter	32.5	1.361 to 63.64	Yes	*	0.0317	
114							
115							
116	Test details	Mean 1	Mean 2	Mean Diff.	SE of diff.	N1	N2
117							
118	Last day SP:2 week winter vs. Last day SP:4 week winter	407.3	412.5	-5.25	9.007	10	10
119	Last day SP:2 week winter vs. Last day SP:8 week winter	407.3	415.4	-8.15	9.007	10	10
120	Last day SP:2 week winter vs. 4 weeks post winter:2 week winter	407.3	349.2	58.03	9.254	10	9

**2way ANOVA** Multiple comparisons							
121	Last day SP:2 week winter vs. 4 weeks post winter:4 week winter	407.3	367.2	40.02	9.007	10	10
122	Last day SP:2 week winter vs. 4 weeks post winter:8 week winter	407.3	365.2	42.1	9.007	10	10
123	Last day SP:2 week winter vs. 8 weeks post winter:2 week winter	407.3	342	65.28	9.254	10	9
124	Last day SP:2 week winter vs. 8 weeks post winter:4 week winter	407.3	345.1	62.18	9.007	10	10
125	Last day SP:2 week winter vs. 8 weeks post winter:8 week winter	407.3	341.7	65.55	9.007	10	10
126	Last day SP:2 week winter vs. SPC -4 weeks post:2 week winter	407.3	418.9	-11.64	9.254	10	9
127	Last day SP:2 week winter vs. SPC -4 weeks post:4 week winter	407.3	471.7	-64.42	9.254	10	9
128	Last day SP:2 week winter vs. SPC -4 weeks post:8 week winter	407.3	397.9	9.4	9.007	10	10
129	Last day SP:2 week winter vs. SPC -8 weeks post:2 week winter	407.3	415.8	-8.5	9.007	10	10
130	Last day SP:2 week winter vs. SPC -8 weeks post:4 week winter	407.3	397.9	9.4	9.007	10	10
131	Last day SP:2 week winter vs. SPC -8 weeks post:8 week winter	407.3	365.4	41.9	9.007	10	10
132	Last day SP:4 week winter vs. Last day SP:8 week winter	412.5	415.4	-2.9	9.007	10	10
133	Last day SP:4 week winter vs. 4 weeks post winter:2 week winter	412.5	349.2	63.28	9.254	10	9
134	Last day SP:4 week winter vs. 4 weeks post winter:4 week winter	412.5	367.2	45.27	9.007	10	10
135	Last day SP:4 week winter vs. 4 weeks post winter:8 week winter	412.5	365.2	47.35	9.007	10	10
136	Last day SP:4 week winter vs. 8 weeks post winter:2 week winter	412.5	342	70.53	9.254	10	9
137	Last day SP:4 week winter vs. 8 weeks post winter:4 week winter	412.5	345.1	67.43	9.007	10	10
138	Last day SP:4 week winter vs. 8 weeks post winter:8 week winter	412.5	341.7	70.8	9.007	10	10
139	Last day SP:4 week winter vs. SPC -4 weeks post:2 week winter	412.5	418.9	-6.389	9.254	10	9
140	Last day SP:4 week winter vs. SPC -4 weeks post:4 week winter	412.5	471.7	-59.17	9.254	10	9
141	Last day SP:4 week winter vs. SPC -4 weeks post:8 week winter	412.5	397.9	14.65	9.007	10	10
142	Last day SP:4 week winter vs. SPC -8 weeks post:2 week winter	412.5	415.8	-3.25	9.007	10	10
143	Last day SP:4 week winter vs. SPC -8 weeks post:4 week winter	412.5	397.9	14.65	9.007	10	10
144	Last day SP:4 week winter vs. SPC -8 weeks post:8 week winter	412.5	365.4	47.15	9.007	10	10
145	Last day SP:8 week winter vs. 4 weeks post winter:2 week winter	415.4	349.2	66.18	9.254	10	9
146	Last day SP:8 week winter vs. 4 weeks post winter:4 week winter	415.4	367.2	48.17	9.007	10	10
147	Last day SP:8 week winter vs. 4 weeks post winter:8 week winter	415.4	365.2	50.25	9.007	10	10
148	Last day SP:8 week winter vs. 8 weeks post winter:2 week winter	415.4	342	73.43	9.254	10	9
149	Last day SP:8 week winter vs. 8 weeks post winter:4 week winter	415.4	345.1	70.33	9.007	10	10
150	Last day SP:8 week winter vs. 8 weeks post winter:8 week winter	415.4	341.7	73.7	9.007	10	10

2way ANOVA<br>Multiple comparisons							
151	Last day SP:8 week winter vs. SPC -4 weeks post:2 week winter	415.4	418.9	-3.489	9.254	10	9
152	Last day SP:8 week winter vs. SPC -4 weeks post:4 week winter	415.4	471.7	-56.27	9.254	10	9
153	Last day SP:8 week winter vs. SPC -4 weeks post:8 week winter	415.4	397.9	17.55	9.007	10	10
154	Last day SP:8 week winter vs. SPC -8 weeks post:2 week winter	415.4	415.8	-0.35	9.007	10	10
155	Last day SP:8 week winter vs. SPC -8 weeks post:4 week winter	415.4	397.9	17.55	9.007	10	10
156	Last day SP:8 week winter vs. SPC -8 weeks post:8 week winter	415.4	365.4	50.05	9.007	10	10
157	4 weeks post winter:2 week winter vs. 4 weeks post winter:4 week winter	349.2	367.2	-18.01	9.254	9	10
158	4 weeks post winter:2 week winter vs. 4 weeks post winter:8 week winter	349.2	365.2	-15.93	9.254	9	10
159	4 weeks post winter:2 week winter vs. 8 weeks post winter:2 week winter	349.2	342	7.25	9.494	9	9
160	4 weeks post winter:2 week winter vs. 8 weeks post winter:4 week winter	349.2	345.1	4.147	9.254	9	10
161	4 weeks post winter:2 week winter vs. 8 weeks post winter:8 week winter	349.2	341.7	7.522	9.254	9	10
162	4 weeks post winter:2 week winter vs. SPC -4 weeks post:2 week winter	349.2	418.9	-69.67	9.494	9	9
163	4 weeks post winter:2 week winter vs. SPC -4 weeks post:4 week winter	349.2	471.7	-122.4	9.494	9	9
164	4 weeks post winter:2 week winter vs. SPC -4 weeks post:8 week winter	349.2	397.9	-48.63	9.254	9	10
165	4 weeks post winter:2 week winter vs. SPC -8 weeks post:2 week winter	349.2	415.8	-66.53	9.254	9	10
166	4 weeks post winter:2 week winter vs. SPC -8 weeks post:4 week winter	349.2	397.9	-48.63	9.254	9	10
167	4 weeks post winter:2 week winter vs. SPC -8 weeks post:8 week winter	349.2	365.4	-16.13	9.254	9	10
168	4 weeks post winter:4 week winter vs. 4 weeks post winter:8 week winter	367.2	365.2	2.083	9.007	10	10
169	4 weeks post winter:4 week winter vs. 8 weeks post winter:2 week winter	367.2	342	25.26	9.254	10	9
170	4 weeks post winter:4 week winter vs. 8 weeks post winter:4 week winter	367.2	345.1	22.16	9.007	10	10
171	4 weeks post winter:4 week winter vs. 8 weeks post winter:8 week winter	367.2	341.7	25.53	9.007	10	10
172	4 weeks post winter:4 week winter vs. SPC -4 weeks post:2 week winter	367.2	418.9	-51.66	9.254	10	9
173	4 weeks post winter:4 week winter vs. SPC -4 weeks post:4 week winter	367.2	471.7	-104.4	9.254	10	9
174	4 weeks post winter:4 week winter vs. SPC -4 weeks post:8 week winter	367.2	397.9	-30.62	9.007	10	10
175	4 weeks post winter:4 week winter vs. SPC -8 weeks post:2 week winter	367.2	415.8	-48.52	9.007	10	10
176	4 weeks post winter:4 week winter vs. SPC -8 weeks post:4 week winter	367.2	397.9	-30.62	9.007	10	10
177	4 weeks post winter:4 week winter vs. SPC -8 weeks post:8 week winter	367.2	365.4	1.883	9.007	10	10
178	4 weeks post winter:8 week winter vs. 8 weeks post winter:2 week winter	365.2	342	23.18	9.254	10	9
179	4 weeks post winter:8 week winter vs. 8 weeks post winter:4 week winter	365.2	345.1	20.08	9.007	10	10
180	4 weeks post winter:8 week winter vs. 8 weeks post winter:8 week winter	365.2	341.7	23.45	9.007	10	10

2way ANOVA<br>Multiple comparisons							
181	4 weeks post winter:8 week winter vs. SPC -4 weeks post:2 week winter	365.2	418.9	-53.74	9.254	10	9
182	4 weeks post winter:8 week winter vs. SPC -4 weeks post:4 week winter	365.2	471.7	-106.5	9.254	10	9
183	4 weeks post winter:8 week winter vs. SPC -4 weeks post:8 week winter	365.2	397.9	-32.7	9.007	10	10
184	4 weeks post winter:8 week winter vs. SPC -8 weeks post:2 week winter	365.2	415.8	-50.6	9.007	10	10
185	4 weeks post winter:8 week winter vs. SPC -8 weeks post:4 week winter	365.2	397.9	-32.7	9.007	10	10
186	4 weeks post winter:8 week winter vs. SPC -8 weeks post:8 week winter	365.2	365.4	-0.2	9.007	10	10
187	8 weeks post winter:2 week winter vs. 8 weeks post winter:4 week winter	342	345.1	-3.103	9.254	9	10
188	8 weeks post winter:2 week winter vs. 8 weeks post winter:8 week winter	342	341.7	0.2722	9.254	9	10
189	8 weeks post winter:2 week winter vs. SPC -4 weeks post:2 week winter	342	418.9	-76.92	9.494	9	9
190	8 weeks post winter:2 week winter vs. SPC -4 weeks post:4 week winter	342	471.7	-129.7	9.494	9	9
191	8 weeks post winter:2 week winter vs. SPC -4 weeks post:8 week winter	342	397.9	-55.88	9.254	9	10
192	8 weeks post winter:2 week winter vs. SPC -8 weeks post:2 week winter	342	415.8	-73.78	9.254	9	10
193	8 weeks post winter:2 week winter vs. SPC -8 weeks post:4 week winter	342	397.9	-55.88	9.254	9	10
194	8 weeks post winter:2 week winter vs. SPC -8 weeks post:8 week winter	342	365.4	-23.38	9.254	9	10
195	8 weeks post winter:4 week winter vs. 8 weeks post winter:8 week winter	345.1	341.7	3.375	9.007	10	10
196	8 weeks post winter:4 week winter vs. SPC -4 weeks post:2 week winter	345.1	418.9	-73.81	9.254	10	9
197	8 weeks post winter:4 week winter vs. SPC -4 weeks post:4 week winter	345.1	471.7	-126.6	9.254	10	9
198	8 weeks post winter:4 week winter vs. SPC -4 weeks post:8 week winter	345.1	397.9	-52.78	9.007	10	10
199	8 weeks post winter:4 week winter vs. SPC -8 weeks post:2 week winter	345.1	415.8	-70.68	9.007	10	10
200	8 weeks post winter:4 week winter vs. SPC -8 weeks post:4 week winter	345.1	397.9	-52.78	9.007	10	10
201	8 weeks post winter:4 week winter vs. SPC -8 weeks post:8 week winter	345.1	365.4	-20.28	9.007	10	10
202	8 weeks post winter:8 week winter vs. SPC -4 weeks post:2 week winter	341.7	418.9	-77.19	9.254	10	9
203	8 weeks post winter:8 week winter vs. SPC -4 weeks post:4 week winter	341.7	471.7	-130	9.254	10	9
204	8 weeks post winter:8 week winter vs. SPC -4 weeks post:8 week winter	341.7	397.9	-56.15	9.007	10	10
205	8 weeks post winter:8 week winter vs. SPC -8 weeks post:2 week winter	341.7	415.8	-74.05	9.007	10	10
206	8 weeks post winter:8 week winter vs. SPC -8 weeks post:4 week winter	341.7	397.9	-56.15	9.007	10	10
207	8 weeks post winter:8 week winter vs. SPC -8 weeks post:8 week winter	341.7	365.4	-23.65	9.007	10	10
208	SPC -4 weeks post:2 week winter vs. SPC -4 weeks post:4 week winter	418.9	471.7	-52.78	9.494	9	9
209	SPC -4 weeks post:2 week winter vs. SPC -4 weeks post:8 week winter	418.9	397.9	21.04	9.254	9	10
210	SPC -4 weeks post:2 week winter vs. SPC -8 weeks post:2 week winter	418.9	415.8	3.139	9.254	9	10

2way ANOVA<br>Multiple comparisons							
211	SPC -4 weeks post:2 week winter vs. SPC -8 weeks post:4 week winter	418.9	397.9	21.04	9.254	9	10
212	SPC -4 weeks post:2 week winter vs. SPC -8 weeks post:8 week winter	418.9	365.4	53.54	9.254	9	10
213	SPC -4 weeks post:4 week winter vs. SPC -4 weeks post:8 week winter	471.7	397.9	73.82	9.254	9	10
214	SPC -4 weeks post:4 week winter vs. SPC -8 weeks post:2 week winter	471.7	415.8	55.92	9.254	9	10
215	SPC -4 weeks post:4 week winter vs. SPC -8 weeks post:4 week winter	471.7	397.9	73.82	9.254	9	10
216	SPC -4 weeks post:4 week winter vs. SPC -8 weeks post:8 week winter	471.7	365.4	106.3	9.254	9	10
217	SPC -4 weeks post:8 week winter vs. SPC -8 weeks post:2 week winter	397.9	415.8	-17.9	9.007	10	10
218	SPC -4 weeks post:8 week winter vs. SPC -8 weeks post:4 week winter	397.9	397.9	0	9.007	10	10
219	SPC -4 weeks post:8 week winter vs. SPC -8 weeks post:8 week winter	397.9	365.4	32.5	9.007	10	10
220	SPC -8 weeks post:2 week winter vs. SPC -8 weeks post:4 week winter	415.8	397.9	17.9	9.007	10	10
221	SPC -8 weeks post:2 week winter vs. SPC -8 weeks post:8 week winter	415.8	365.4	50.4	9.007	10	10
222	SPC -8 weeks post:4 week winter vs. SPC -8 weeks post:8 week winter	397.9	365.4	32.5	9.007	10	10

1		
2		
3		
4		
5		
6		
7		
8		
9		
10		
11		
12		
13		
14		
15		
16		
17		
18		
19		
20		
21		
22		
23		
24		
25		
26		
27		
28		
29		
30		

31		
32		
33		
34		
35		
36		
37		
38		
39		
40		
41		
42		
43		
44		
45		
46		
47		
48		
49		
50		
51		
52		
53		
54		
55		
56		
57		
58		
59		
60		

61		
62		
63		
64		
65		
66		
67		
68		
69		
70		
71		
72		
73		
74		
75		
76		
77		
78		
79		
80		
81		
82		
83		
84		
85		
86		
87		
88		
89		
90		

91		
92		
93		
94		
95		
96		
97		
98		
99		
100		
101		
102		
103		
104		
105		
106		
107		
108		
109		
110		
111		
112		
113		
114		
115		
116	q	DF
117		
118	0.8243	131
119	1.28	131
120	8.868	131

121	6.283	131
122	6.61	131
123	9.976	131
124	9.762	131
125	10.29	131
126	1.779	131
127	9.845	131
128	1.476	131
129	1.335	131
130	1.476	131
131	6.579	131
132	0.4553	131
133	9.671	131
134	7.108	131
135	7.435	131
136	10.78	131
137	10.59	131
138	11.12	131
139	0.9764	131
140	9.042	131
141	2.3	131
142	0.5103	131
143	2.3	131
144	7.403	131
145	10.11	131
146	7.563	131
147	7.89	131
148	11.22	131
149	11.04	131
150	11.57	131

151	0.5332	131
152	8.599	131
153	2.756	131
154	0.05495	131
155	2.756	131
156	7.859	131
157	2.753	131
158	2.434	131
159	1.08	131
160	0.6338	131
161	1.15	131
162	10.38	131
163	18.24	131
164	7.432	131
165	10.17	131
166	7.432	131
167	2.465	131
168	0.3271	131
169	3.861	131
170	3.479	131
171	4.009	131
172	7.894	131
173	15.96	131
174	4.807	131
175	7.618	131
176	4.807	131
177	0.2957	131
178	3.542	131
179	3.152	131
180	3.682	131

181	8.213	131
182	16.28	131
183	5.134	131
184	7.945	131
185	5.134	131
186	0.0314	131
187	0.4742	131
188	0.0416	131
189	11.46	131
190	19.32	131
191	8.54	131
192	11.28	131
193	8.54	131
194	3.573	131
195	0.5299	131
196	11.28	131
197	19.35	131
198	8.286	131
199	11.1	131
200	8.286	131
201	3.183	131
202	11.8	131
203	19.86	131
204	8.816	131
205	11.63	131
206	8.816	131
207	3.713	131
208	7.862	131
209	3.215	131
210	0.4797	131

211	3.215	131
212	8.182	131
213	11.28	131
214	8.546	131
215	11.28	131
216	16.25	131
217	2.811	131
218	0	131
219	5.103	131
220	2.811	131
221	7.914	131
222	5.103	131

Data analyzed: Osmolality 2-ANOVA

Source of Variation	Degrees of Freedom	Sum of Squares	Mean square
Pretreatment	2	11772	5886
Treatment	4	146229	36557
Interaction	8	31215	3902
Residual (error)	131	53137	405.6
Total	145		

Does Pretreatment have the same effect at all values of Treatment?

Interaction accounts for approximately 13.08% of the total variance.
F = 9.62. DFn=8 DFd=131
The P value is < 0.0001
If there is no interaction overall, there is a less than 0.01% chance of randomly observing so much interaction in an experiment of this size. The interaction is considered extremely significant.

Since the interaction is statistically significant, the P values that follow for the row and column effects are difficult to interpret.

Does Pretreatment affect the result?

Pretreatment accounts for approximately 4.93% of the total variance.
F = 14.51. DFn=2 DFd=131
The P value is < 0.0001
If Pretreatment has no effect overall, there is a less than 0.01% chance of randomly observing an effect this big (or bigger) in an experiment of this size. The effect is considered extremely significant.

Does Treatment affect the result?

Treatment accounts for approximately 61.28% of the total variance.
F = 90.13. DFn=4 DFd=131
The P value is < 0.0001
If Treatment has no effect overall, there is a less than 0.01% chance of randomly observing an this big (or bigger) in an experiment of this size. The effect is considered extremely significant.
