# Supplementary material for: RNA profiling identifies novel, photoperiod-history dependent markers associated with enhanced saltwater performance in juvenile Atlantic salmon
Source: PLoS One. 2020 Apr 8;15(4):e0227496. doi: 10.1371/journal.pone.0227496 (PMC7141700; doi:10.1371/journal.pone.0227496)
Supplement: S3 Table — Table showing the 2-way ANOVA and multiple comparison results for the gill Na+, K+- ATPase activity measurements in experiment 2. (PDF) [file pone.0227496.s004.pdf]

2way ANOVA
Tabular results

1	Table Analyzed	NKA				
2						
3	Two-way ANOVA	Ordinary				
4	Alpha	0.05				
5						
6	Source of Variation	% of total variation	P value	P value summary	Significant?	
7	Interaction	17.94	0.0001	***	Yes	
8	Treatment	16.43	<0.0001	****	Yes	
9	Pre-treatment	11.33	<0.0001	****	Yes	
10						
11	ANOVA table	SS (Type III)	DF	MS	F (DFn, DFd)	P value
12	Interaction	218.7	8	27.34	F (8, 116) = 4.438	P=0.0001
13	Treatment	200.3	4	50.07	F (4, 116) = 8.126	P<0.0001
14	Pre-treatment	138.2	2	69.09	F (2, 116) = 11.21	P<0.0001
15	Residual	714.7	116	6.162		
16						
17	Number of missing values	19				

2way ANOVA
Multiple comparisons

1	Compare cell means regardless of rows and columns						
2							
3	Number of families	1					
4	Number of comparisons per family	105					
5	Alpha	0.05					
6							
7	Tukey's multiple comparisons test	Mean Diff.	95.00% CI of diff.	Significant?	Summary	Adjusted P Value	
8							
9	Last day SP:2 week winter vs. Last day SP:4 week winter	1.007	-2.841 to 4.854	No	ns	0.9999	
10	Last day SP:2 week winter vs. Last day SP:8 week winter	0.08288	-3.765 to 3.93	No	ns	>0.9999	
11	Last day SP:2 week winter vs. 4 weeks post winter:2 week winter	0.577	-3.27 to 4.424	No	ns	>0.9999	
12	Last day SP:2 week winter vs. 4 weeks post winter:4 week winter	-1.097	-4.944 to 2.751	No	ns	0.9996	
13	Last day SP:2 week winter vs. 4 weeks post winter:8 week winter	-2.436	-6.389 to 1.516	No	ns	0.7080	
14	Last day SP:2 week winter vs. 8 weeks post winter:2 week winter	0.5319	-3.316 to 4.379	No	ns	>0.9999	
15	Last day SP:2 week winter vs. 8 weeks post winter:4 week winter	-1.685	-5.766 to 2.395	No	ns	0.9827	
16	Last day SP:2 week winter vs. 8 weeks post winter:8 week winter	-7.472	-11.71 to -3.232	Yes	****	<0.0001	
17	Last day SP:2 week winter vs. SPC -4 weeks post:2 week winter	1.063	-3.177 to 5.302	No	ns	>0.9999	
18	Last day SP:2 week winter vs. SPC -4 weeks post:4 week winter	-0.3998	-4.842 to 4.043	No	ns	>0.9999	
19	Last day SP:2 week winter vs. SPC -4 weeks post:8 week winter	1.073	-3.008 to 5.154	No	ns	0.9998	
20	Last day SP:2 week winter vs. SPC -8 weeks post:2 week winter	0.2986	-3.782 to 4.379	No	ns	>0.9999	
21	Last day SP:2 week winter vs. SPC -8 weeks post:4 week winter	1.073	-3.008 to 5.154	No	ns	0.9998	
22	Last day SP:2 week winter vs. SPC -8 weeks post:8 week winter	-1.046	-4.893 to 2.801	No	ns	0.9998	
23	Last day SP:4 week winter vs. Last day SP:8 week winter	-0.9238	-4.771 to 2.924	No	ns	>0.9999	
24	Last day SP:4 week winter vs. 4 weeks post winter:2 week winter	-0.4297	-4.277 to 3.418	No	ns	>0.9999	
25	Last day SP:4 week winter vs. 4 weeks post winter:4 week winter	-2.103	-5.951 to 1.744	No	ns	0.8509	
26	Last day SP:4 week winter vs. 4 weeks post winter:8 week winter	-3.443	-7.396 to 0.5097	No	ns	0.1622	
27	Last day SP:4 week winter vs. 8 weeks post winter:2 week winter	-0.4748	-4.322 to 3.373	No	ns	>0.9999	
28	Last day SP:4 week winter vs. 8 weeks post winter:4 week winter	-2.692	-6.773 to 1.389	No	ns	0.6025	
29	Last day SP:4 week winter vs. 8 weeks post winter:8 week winter	-8.479	-12.72 to -4.239	Yes	****	<0.0001	
30	Last day SP:4 week winter vs. SPC -4 weeks post:2 week winter	0.05614	-4.184 to 4.296	No	ns	>0.9999	

2way ANOVA<br>Multiple comparisons							
31	Last day SP:4 week winter vs. SPC -4 weeks post:4 week winter	-1.407	-5.849 to 3.036	No	ns	0.9988	
32	Last day SP:4 week winter vs. SPC -4 weeks post:8 week winter	0.06628	-4.015 to 4.147	No	ns	>0.9999	
33	Last day SP:4 week winter vs. SPC -8 weeks post:2 week winter	-0.7081	-4.789 to 3.373	No	ns	>0.9999	
34	Last day SP:4 week winter vs. SPC -8 weeks post:4 week winter	0.06628	-4.015 to 4.147	No	ns	>0.9999	
35	Last day SP:4 week winter vs. SPC -8 weeks post:8 week winter	-2.053	-5.9 to 1.795	No	ns	0.8724	
36	Last day SP:8 week winter vs. 4 weeks post winter:2 week winter	0.4941	-3.353 to 4.342	No	ns	>0.9999	
37	Last day SP:8 week winter vs. 4 weeks post winter:4 week winter	-1.18	-5.027 to 2.668	No	ns	0.9991	
38	Last day SP:8 week winter vs. 4 weeks post winter:8 week winter	-2.519	-6.472 to 1.434	No	ns	0.6579	
39	Last day SP:8 week winter vs. 8 weeks post winter:2 week winter	0.449	-3.398 to 4.296	No	ns	>0.9999	
40	Last day SP:8 week winter vs. 8 weeks post winter:4 week winter	-1.768	-5.849 to 2.313	No	ns	0.9736	
41	Last day SP:8 week winter vs. 8 weeks post winter:8 week winter	-7.555	-11.79 to -3.315	Yes	****	<0.0001	
42	Last day SP:8 week winter vs. SPC -4 weeks post:2 week winter	0.9799	-3.26 to 5.22	No	ns	>0.9999	
43	Last day SP:8 week winter vs. SPC -4 weeks post:4 week winter	-0.4827	-4.925 to 3.96	No	ns	>0.9999	
44	Last day SP:8 week winter vs. SPC -4 weeks post:8 week winter	0.9901	-3.091 to 5.071	No	ns	>0.9999	
45	Last day SP:8 week winter vs. SPC -8 weeks post:2 week winter	0.2157	-3.865 to 4.297	No	ns	>0.9999	
46	Last day SP:8 week winter vs. SPC -8 weeks post:4 week winter	0.9901	-3.091 to 5.071	No	ns	>0.9999	
47	Last day SP:8 week winter vs. SPC -8 weeks post:8 week winter	-1.129	-4.976 to 2.718	No	ns	0.9995	
48	4 weeks post winter:2 week winter vs. 4 weeks post winter:4 week winter	-1.674	-5.521 to 2.174	No	ns	0.9727	
49	4 weeks post winter:2 week winter vs. 4 weeks post winter:8 week winter	-3.013	-6.966 to 0.9394	No	ns	0.3539	
50	4 weeks post winter:2 week winter vs. 8 weeks post winter:2 week winter	-0.04514	-3.893 to 3.802	No	ns	>0.9999	
51	4 weeks post winter:2 week winter vs. 8 weeks post winter:4 week winter	-2.262	-6.343 to 1.818	No	ns	0.8374	
52	4 weeks post winter:2 week winter vs. 8 weeks post winter:8 week winter	-8.049	-12.29 to -3.809	Yes	****	<0.0001	
53	4 weeks post winter:2 week winter vs. SPC -4 weeks post:2 week winter	0.4858	-3.754 to 4.725	No	ns	>0.9999	
54	4 weeks post winter:2 week winter vs. SPC -4 weeks post:4 week winter	-0.9768	-5.419 to 3.466	No	ns	>0.9999	
55	4 weeks post winter:2 week winter vs. SPC -4 weeks post:8 week winter	0.496	-3.585 to 4.577	No	ns	>0.9999	
56	4 weeks post winter:2 week winter vs. SPC -8 weeks post:2 week winter	-0.2784	-4.359 to 3.802	No	ns	>0.9999	
57	4 weeks post winter:2 week winter vs. SPC -8 weeks post:4 week winter	0.496	-3.585 to 4.577	No	ns	>0.9999	
58	4 weeks post winter:2 week winter vs. SPC -8 weeks post:8 week winter	-1.623	-5.471 to 2.224	No	ns	0.9791	
59	4 weeks post winter:4 week winter vs. 4 weeks post winter:8 week winter	-1.34	-5.293 to 2.613	No	ns	0.9975	
60	4 weeks post winter:4 week winter vs. 8 weeks post winter:2 week winter	1.629	-2.219 to 5.476	No	ns	0.9784	

2way ANOVA<br>Multiple comparisons							
61	4 weeks post winter:4 week winter vs. 8 weeks post winter:4 week winter	-0.5886	-4.669 to 3.492	No	ns	>0.9999	
62	4 weeks post winter:4 week winter vs. 8 weeks post winter:8 week winter	-6.375	-10.61 to -2.135	Yes	****	<0.0001	
63	4 weeks post winter:4 week winter vs. SPC -4 weeks post:2 week winter	2.16	-2.08 to 6.399	No	ns	0.9068	
64	4 weeks post winter:4 week winter vs. SPC -4 weeks post:4 week winter	0.6969	-3.746 to 5.14	No	ns	>0.9999	
65	4 weeks post winter:4 week winter vs. SPC -4 weeks post:8 week winter	2.17	-1.911 to 6.251	No	ns	0.8753	
66	4 weeks post winter:4 week winter vs. SPC -8 weeks post:2 week winter	1.395	-2.685 to 5.476	No	ns	0.9972	
67	4 weeks post winter:4 week winter vs. SPC -8 weeks post:4 week winter	2.17	-1.911 to 6.251	No	ns	0.8753	
68	4 weeks post winter:4 week winter vs. SPC -8 weeks post:8 week winter	0.05069	-3.797 to 3.898	No	ns	>0.9999	
69	4 weeks post winter:8 week winter vs. 8 weeks post winter:2 week winter	2.968	-0.9845 to 6.921	No	ns	0.3793	
70	4 weeks post winter:8 week winter vs. 8 weeks post winter:4 week winter	0.7512	-3.429 to 4.932	No	ns	>0.9999	
71	4 weeks post winter:8 week winter vs. 8 weeks post winter:8 week winter	-5.035	-9.371 to -0.6998	Yes	**	0.0084	
72	4 weeks post winter:8 week winter vs. SPC -4 weeks post:2 week winter	3.499	-0.8363 to 7.835	No	ns	0.2633	
73	4 weeks post winter:8 week winter vs. SPC -4 weeks post:4 week winter	2.037	-2.498 to 6.571	No	ns	0.9643	
74	4 weeks post winter:8 week winter vs. SPC -4 weeks post:8 week winter	3.509	-0.6709 to 7.69	No	ns	0.2078	
75	4 weeks post winter:8 week winter vs. SPC -8 weeks post:2 week winter	2.735	-1.445 to 6.915	No	ns	0.6161	
76	4 weeks post winter:8 week winter vs. SPC -8 weeks post:4 week winter	3.509	-0.6709 to 7.69	No	ns	0.2078	
77	4 weeks post winter:8 week winter vs. SPC -8 weeks post:8 week winter	1.39	-2.562 to 5.343	No	ns	0.9963	
78	8 weeks post winter:2 week winter vs. 8 weeks post winter:4 week winter	-2.217	-6.298 to 1.864	No	ns	0.8566	
79	8 weeks post winter:2 week winter vs. 8 weeks post winter:8 week winter	-8.004	-12.24 to -3.764	Yes	****	<0.0001	
80	8 weeks post winter:2 week winter vs. SPC -4 weeks post:2 week winter	0.531	-3.709 to 4.771	No	ns	>0.9999	
81	8 weeks post winter:2 week winter vs. SPC -4 weeks post:4 week winter	-0.9317	-5.374 to 3.511	No	ns	>0.9999	
82	8 weeks post winter:2 week winter vs. SPC -4 weeks post:8 week winter	0.5411	-3.54 to 4.622	No	ns	>0.9999	
83	8 weeks post winter:2 week winter vs. SPC -8 weeks post:2 week winter	-0.2333	-4.314 to 3.848	No	ns	>0.9999	
84	8 weeks post winter:2 week winter vs. SPC -8 weeks post:4 week winter	0.5411	-3.54 to 4.622	No	ns	>0.9999	
85	8 weeks post winter:2 week winter vs. SPC -8 weeks post:8 week winter	-1.578	-5.425 to 2.269	No	ns	0.9837	
86	8 weeks post winter:4 week winter vs. 8 weeks post winter:8 week winter	-5.787	-10.24 to -1.334	Yes	**	0.0015	
87	8 weeks post winter:4 week winter vs. SPC -4 weeks post:2 week winter	2.748	-1.704 to 7.201	No	ns	0.7061	
88	8 weeks post winter:4 week winter vs. SPC -4 weeks post:4 week winter	1.285	-3.361 to 5.932	No	ns	0.9997	
89	8 weeks post winter:4 week winter vs. SPC -4 weeks post:8 week winter	2.758	-1.543 to 7.06	No	ns	0.6484	
90	8 weeks post winter:4 week winter vs. SPC -8 weeks post:2 week winter	1.984	-2.318 to 6.285	No	ns	0.9558	

2way ANOVA Multiple comparisons							
91	8 weeks post winter:4 week winter vs. SPC -8 weeks post:4 week winter	2.758	-1.543 to 7.06	No	ns	0.6484	
92	8 weeks post winter:4 week winter vs. SPC -8 weeks post:8 week winter	0.6392	-3.442 to 4.72	No	ns	>0.9999	
93	8 weeks post winter:8 week winter vs. SPC -4 weeks post:2 week winter	8.535	3.936 to 13.13	Yes	****	<0.0001	
94	8 weeks post winter:8 week winter vs. SPC -4 weeks post:4 week winter	7.072	2.286 to 11.86	Yes	***	0.0001	
95	8 weeks post winter:8 week winter vs. SPC -4 weeks post:8 week winter	8.545	4.092 to 13	Yes	****	<0.0001	
96	8 weeks post winter:8 week winter vs. SPC -8 weeks post:2 week winter	7.77	3.318 to 12.22	Yes	****	<0.0001	
97	8 weeks post winter:8 week winter vs. SPC -8 weeks post:4 week winter	8.545	4.092 to 13	Yes	****	<0.0001	
98	8 weeks post winter:8 week winter vs. SPC -8 weeks post:8 week winter	6.426	2.186 to 10.67	Yes	****	<0.0001	
99	SPC -4 weeks post:2 week winter vs. SPC -4 weeks post:4 week winter	-1.463	-6.249 to 3.324	No	ns	0.9992	
100	SPC -4 weeks post:2 week winter vs. SPC -4 weeks post:8 week winter	0.01015	-4.442 to 4.463	No	ns	>0.9999	
101	SPC -4 weeks post:2 week winter vs. SPC -8 weeks post:2 week winter	-0.7642	-5.217 to 3.688	No	ns	>0.9999	
102	SPC -4 weeks post:2 week winter vs. SPC -8 weeks post:4 week winter	0.01015	-4.442 to 4.463	No	ns	>0.9999	
103	SPC -4 weeks post:2 week winter vs. SPC -8 weeks post:8 week winter	-2.109	-6.349 to 2.131	No	ns	0.9213	
104	SPC -4 weeks post:4 week winter vs. SPC -4 weeks post:8 week winter	1.473	-3.173 to 6.119	No	ns	0.9987	
105	SPC -4 weeks post:4 week winter vs. SPC -8 weeks post:2 week winter	0.6984	-3.948 to 5.345	No	ns	>0.9999	
106	SPC -4 weeks post:4 week winter vs. SPC -8 weeks post:4 week winter	1.473	-3.173 to 6.119	No	ns	0.9987	
107	SPC -4 weeks post:4 week winter vs. SPC -8 weeks post:8 week winter	-0.6462	-5.089 to 3.796	No	ns	>0.9999	
108	SPC -4 weeks post:8 week winter vs. SPC -8 weeks post:2 week winter	-0.7744	-5.076 to 3.527	No	ns	>0.9999	
109	SPC -4 weeks post:8 week winter vs. SPC -8 weeks post:4 week winter	0	-4.302 to 4.302	No	ns	>0.9999	
110	SPC -4 weeks post:8 week winter vs. SPC -8 weeks post:8 week winter	-2.119	-6.2 to 1.962	No	ns	0.8935	
111	SPC -8 weeks post:2 week winter vs. SPC -8 weeks post:4 week winter	0.7744	-3.527 to 5.076	No	ns	>0.9999	
112	SPC -8 weeks post:2 week winter vs. SPC -8 weeks post:8 week winter	-1.345	-5.425 to 2.736	No	ns	0.9981	
113	SPC -8 weeks post:4 week winter vs. SPC -8 weeks post:8 week winter	-2.119	-6.2 to 1.962	No	ns	0.8935	
114							
115							
116	Test details	Mean 1	Mean 2	Mean Diff.	SE of diff.	N1	N2
117							
118	Last day SP:2 week winter vs. Last day SP:4 week winter	2.945	1.939	1.007	1.11	10	10
119	Last day SP:2 week winter vs. Last day SP:8 week winter	2.945	2.862	0.08288	1.11	10	10
120	Last day SP:2 week winter vs. 4 weeks post winter:2 week winter	2.945	2.368	0.577	1.11	10	10

2way ANOVA<br>Multiple comparisons							
121	Last day SP:2 week winter vs. 4 weeks post winter:4 week winter	2.945	4.042	-1.097	1.11	10	10
122	Last day SP:2 week winter vs. 4 weeks post winter:8 week winter	2.945	5.382	-2.436	1.141	10	9
123	Last day SP:2 week winter vs. 8 weeks post winter:2 week winter	2.945	2.413	0.5319	1.11	10	10
124	Last day SP:2 week winter vs. 8 weeks post winter:4 week winter	2.945	4.631	-1.685	1.177	10	8
125	Last day SP:2 week winter vs. 8 weeks post winter:8 week winter	2.945	10.42	-7.472	1.223	10	7
126	Last day SP:2 week winter vs. SPC -4 weeks post:2 week winter	2.945	1.882	1.063	1.223	10	7
127	Last day SP:2 week winter vs. SPC -4 weeks post:4 week winter	2.945	3.345	-0.3998	1.282	10	6
128	Last day SP:2 week winter vs. SPC -4 weeks post:8 week winter	2.945	1.872	1.073	1.177	10	8
129	Last day SP:2 week winter vs. SPC -8 weeks post:2 week winter	2.945	2.647	0.2986	1.177	10	8
130	Last day SP:2 week winter vs. SPC -8 weeks post:4 week winter	2.945	1.872	1.073	1.177	10	8
131	Last day SP:2 week winter vs. SPC -8 weeks post:8 week winter	2.945	3.991	-1.046	1.11	10	10
132	Last day SP:4 week winter vs. Last day SP:8 week winter	1.939	2.862	-0.9238	1.11	10	10
133	Last day SP:4 week winter vs. 4 weeks post winter:2 week winter	1.939	2.368	-0.4297	1.11	10	10
134	Last day SP:4 week winter vs. 4 weeks post winter:4 week winter	1.939	4.042	-2.103	1.11	10	10
135	Last day SP:4 week winter vs. 4 weeks post winter:8 week winter	1.939	5.382	-3.443	1.141	10	9
136	Last day SP:4 week winter vs. 8 weeks post winter:2 week winter	1.939	2.413	-0.4748	1.11	10	10
137	Last day SP:4 week winter vs. 8 weeks post winter:4 week winter	1.939	4.631	-2.692	1.177	10	8
138	Last day SP:4 week winter vs. 8 weeks post winter:8 week winter	1.939	10.42	-8.479	1.223	10	7
139	Last day SP:4 week winter vs. SPC -4 weeks post:2 week winter	1.939	1.882	0.05614	1.223	10	7
140	Last day SP:4 week winter vs. SPC -4 weeks post:4 week winter	1.939	3.345	-1.407	1.282	10	6
141	Last day SP:4 week winter vs. SPC -4 weeks post:8 week winter	1.939	1.872	0.06628	1.177	10	8
142	Last day SP:4 week winter vs. SPC -8 weeks post:2 week winter	1.939	2.647	-0.7081	1.177	10	8
143	Last day SP:4 week winter vs. SPC -8 weeks post:4 week winter	1.939	1.872	0.06628	1.177	10	8
144	Last day SP:4 week winter vs. SPC -8 weeks post:8 week winter	1.939	3.991	-2.053	1.11	10	10
145	Last day SP:8 week winter vs. 4 weeks post winter:2 week winter	2.862	2.368	0.4941	1.11	10	10
146	Last day SP:8 week winter vs. 4 weeks post winter:4 week winter	2.862	4.042	-1.18	1.11	10	10
147	Last day SP:8 week winter vs. 4 weeks post winter:8 week winter	2.862	5.382	-2.519	1.141	10	9
148	Last day SP:8 week winter vs. 8 weeks post winter:2 week winter	2.862	2.413	0.449	1.11	10	10
149	Last day SP:8 week winter vs. 8 weeks post winter:4 week winter	2.862	4.631	-1.768	1.177	10	8
150	Last day SP:8 week winter vs. 8 weeks post winter:8 week winter	2.862	10.42	-7.555	1.223	10	7

2way ANOVA Multiple comparisons							
151	Last day SP:8 week winter vs. SPC -4 weeks post:2 week winter	2.862	1.882	0.9799	1.223	10	7
152	Last day SP:8 week winter vs. SPC -4 weeks post:4 week winter	2.862	3.345	-0.4827	1.282	10	6
153	Last day SP:8 week winter vs. SPC -4 weeks post:8 week winter	2.862	1.872	0.9901	1.177	10	8
154	Last day SP:8 week winter vs. SPC -8 weeks post:2 week winter	2.862	2.647	0.2157	1.177	10	8
155	Last day SP:8 week winter vs. SPC -8 weeks post:4 week winter	2.862	1.872	0.9901	1.177	10	8
156	Last day SP:8 week winter vs. SPC -8 weeks post:8 week winter	2.862	3.991	-1.129	1.11	10	10
157	4 weeks post winter:2 week winter vs. 4 weeks post winter:4 week winter	2.368	4.042	-1.674	1.11	10	10
158	4 weeks post winter:2 week winter vs. 4 weeks post winter:8 week winter	2.368	5.382	-3.013	1.141	10	9
159	4 weeks post winter:2 week winter vs. 8 weeks post winter:2 week winter	2.368	2.413	-0.04514	1.11	10	10
160	4 weeks post winter:2 week winter vs. 8 weeks post winter:4 week winter	2.368	4.631	-2.262	1.177	10	8
161	4 weeks post winter:2 week winter vs. 8 weeks post winter:8 week winter	2.368	10.42	-8.049	1.223	10	7
162	4 weeks post winter:2 week winter vs. SPC -4 weeks post:2 week winter	2.368	1.882	0.4858	1.223	10	7
163	4 weeks post winter:2 week winter vs. SPC -4 weeks post:4 week winter	2.368	3.345	-0.9768	1.282	10	6
164	4 weeks post winter:2 week winter vs. SPC -4 weeks post:8 week winter	2.368	1.872	0.496	1.177	10	8
165	4 weeks post winter:2 week winter vs. SPC -8 weeks post:2 week winter	2.368	2.647	-0.2784	1.177	10	8
166	4 weeks post winter:2 week winter vs. SPC -8 weeks post:4 week winter	2.368	1.872	0.496	1.177	10	8
167	4 weeks post winter:2 week winter vs. SPC -8 weeks post:8 week winter	2.368	3.991	-1.623	1.11	10	10
168	4 weeks post winter:4 week winter vs. 4 weeks post winter:8 week winter	4.042	5.382	-1.34	1.141	10	9
169	4 weeks post winter:4 week winter vs. 8 weeks post winter:2 week winter	4.042	2.413	1.629	1.11	10	10
170	4 weeks post winter:4 week winter vs. 8 weeks post winter:4 week winter	4.042	4.631	-0.5886	1.177	10	8
171	4 weeks post winter:4 week winter vs. 8 weeks post winter:8 week winter	4.042	10.42	-6.375	1.223	10	7
172	4 weeks post winter:4 week winter vs. SPC -4 weeks post:2 week winter	4.042	1.882	2.16	1.223	10	7
173	4 weeks post winter:4 week winter vs. SPC -4 weeks post:4 week winter	4.042	3.345	0.6969	1.282	10	6
174	4 weeks post winter:4 week winter vs. SPC -4 weeks post:8 week winter	4.042	1.872	2.17	1.177	10	8
175	4 weeks post winter:4 week winter vs. SPC -8 weeks post:2 week winter	4.042	2.647	1.395	1.177	10	8
176	4 weeks post winter:4 week winter vs. SPC -8 weeks post:4 week winter	4.042	1.872	2.17	1.177	10	8
177	4 weeks post winter:4 week winter vs. SPC -8 weeks post:8 week winter	4.042	3.991	0.05069	1.11	10	10
178	4 weeks post winter:8 week winter vs. 8 weeks post winter:2 week winter	5.382	2.413	2.968	1.141	9	10
179	4 weeks post winter:8 week winter vs. 8 weeks post winter:4 week winter	5.382	4.631	0.7512	1.206	9	8
180	4 weeks post winter:8 week winter vs. 8 weeks post winter:8 week winter	5.382	10.42	-5.035	1.251	9	7

2way ANOVA<br>Multiple comparisons							
181	4 weeks post winter:8 week winter vs. SPC -4 weeks post:2 week winter	5.382	1.882	3.499	1.251	9	7
182	4 weeks post winter:8 week winter vs. SPC -4 weeks post:4 week winter	5.382	3.345	2.037	1.308	9	6
183	4 weeks post winter:8 week winter vs. SPC -4 weeks post:8 week winter	5.382	1.872	3.509	1.206	9	8
184	4 weeks post winter:8 week winter vs. SPC -8 weeks post:2 week winter	5.382	2.647	2.735	1.206	9	8
185	4 weeks post winter:8 week winter vs. SPC -8 weeks post:4 week winter	5.382	1.872	3.509	1.206	9	8
186	4 weeks post winter:8 week winter vs. SPC -8 weeks post:8 week winter	5.382	3.991	1.39	1.141	9	10
187	8 weeks post winter:2 week winter vs. 8 weeks post winter:4 week winter	2.413	4.631	-2.217	1.177	10	8
188	8 weeks post winter:2 week winter vs. 8 weeks post winter:8 week winter	2.413	10.42	-8.004	1.223	10	7
189	8 weeks post winter:2 week winter vs. SPC -4 weeks post:2 week winter	2.413	1.882	0.531	1.223	10	7
190	8 weeks post winter:2 week winter vs. SPC -4 weeks post:4 week winter	2.413	3.345	-0.9317	1.282	10	6
191	8 weeks post winter:2 week winter vs. SPC -4 weeks post:8 week winter	2.413	1.872	0.5411	1.177	10	8
192	8 weeks post winter:2 week winter vs. SPC -8 weeks post:2 week winter	2.413	2.647	-0.2333	1.177	10	8
193	8 weeks post winter:2 week winter vs. SPC -8 weeks post:4 week winter	2.413	1.872	0.5411	1.177	10	8
194	8 weeks post winter:2 week winter vs. SPC -8 weeks post:8 week winter	2.413	3.991	-1.578	1.11	10	10
195	8 weeks post winter:4 week winter vs. 8 weeks post winter:8 week winter	4.631	10.42	-5.787	1.285	8	7
196	8 weeks post winter:4 week winter vs. SPC -4 weeks post:2 week winter	4.631	1.882	2.748	1.285	8	7
197	8 weeks post winter:4 week winter vs. SPC -4 weeks post:4 week winter	4.631	3.345	1.285	1.341	8	6
198	8 weeks post winter:4 week winter vs. SPC -4 weeks post:8 week winter	4.631	1.872	2.758	1.241	8	8
199	8 weeks post winter:4 week winter vs. SPC -8 weeks post:2 week winter	4.631	2.647	1.984	1.241	8	8
200	8 weeks post winter:4 week winter vs. SPC -8 weeks post:4 week winter	4.631	1.872	2.758	1.241	8	8
201	8 weeks post winter:4 week winter vs. SPC -8 weeks post:8 week winter	4.631	3.991	0.6392	1.177	8	10
202	8 weeks post winter:8 week winter vs. SPC -4 weeks post:2 week winter	10.42	1.882	8.535	1.327	7	7
203	8 weeks post winter:8 week winter vs. SPC -4 weeks post:4 week winter	10.42	3.345	7.072	1.381	7	6
204	8 weeks post winter:8 week winter vs. SPC -4 weeks post:8 week winter	10.42	1.872	8.545	1.285	7	8
205	8 weeks post winter:8 week winter vs. SPC -8 weeks post:2 week winter	10.42	2.647	7.77	1.285	7	8
206	8 weeks post winter:8 week winter vs. SPC -8 weeks post:4 week winter	10.42	1.872	8.545	1.285	7	8
207	8 weeks post winter:8 week winter vs. SPC -8 weeks post:8 week winter	10.42	3.991	6.426	1.223	7	10
208	SPC -4 weeks post:2 week winter vs. SPC -4 weeks post:4 week winter	1.882	3.345	-1.463	1.381	7	6
209	SPC -4 weeks post:2 week winter vs. SPC -4 weeks post:8 week winter	1.882	1.872	0.01015	1.285	7	8
210	SPC -4 weeks post:2 week winter vs. SPC -8 weeks post:2 week winter	1.882	2.647	-0.7642	1.285	7	8

2way ANOVA<br>Multiple comparisons							
211	SPC -4 weeks post:2 week winter vs. SPC -8 weeks post:4 week winter	1.882	1.872	0.01015	1.285	7	8
212	SPC -4 weeks post:2 week winter vs. SPC -8 weeks post:8 week winter	1.882	3.991	-2.109	1.223	7	10
213	SPC -4 weeks post:4 week winter vs. SPC -4 weeks post:8 week winter	3.345	1.872	1.473	1.341	6	8
214	SPC -4 weeks post:4 week winter vs. SPC -8 weeks post:2 week winter	3.345	2.647	0.6984	1.341	6	8
215	SPC -4 weeks post:4 week winter vs. SPC -8 weeks post:4 week winter	3.345	1.872	1.473	1.341	6	8
216	SPC -4 weeks post:4 week winter vs. SPC -8 weeks post:8 week winter	3.345	3.991	-0.6462	1.282	6	10
217	SPC -4 weeks post:8 week winter vs. SPC -8 weeks post:2 week winter	1.872	2.647	-0.7744	1.241	8	8
218	SPC -4 weeks post:8 week winter vs. SPC -8 weeks post:4 week winter	1.872	1.872	0	1.241	8	8
219	SPC -4 weeks post:8 week winter vs. SPC -8 weeks post:8 week winter	1.872	3.991	-2.119	1.177	8	10
220	SPC -8 weeks post:2 week winter vs. SPC -8 weeks post:4 week winter	2.647	1.872	0.7744	1.241	8	8
221	SPC -8 weeks post:2 week winter vs. SPC -8 weeks post:8 week winter	2.647	3.991	-1.345	1.177	8	10
222	SPC -8 weeks post:4 week winter vs. SPC -8 weeks post:8 week winter	1.872	3.991	-2.119	1.177	8	10

1		
2		
3		
4		
5		
6		
7		
8		
9		
10		
11		
12		
13		
14		
15		
16		
17		
18		
19		
20		
21		
22		
23		
24		
25		
26		
27		
28		
29		
30		

31		
32		
33		
34		
35		
36		
37		
38		
39		
40		
41		
42		
43		
44		
45		
46		
47		
48		
49		
50		
51		
52		
53		
54		
55		
56		
57		
58		
59		
60		

61		
62		
63		
64		
65		
66		
67		
68		
69		
70		
71		
72		
73		
74		
75		
76		
77		
78		
79		
80		
81		
82		
83		
84		
85		
86		
87		
88		
89		
90		

91		
92		
93		
94		
95		
96		
97		
98		
99		
100		
101		
102		
103		
104		
105		
106		
107		
108		
109		
110		
111		
112		
113		
114		
115		
116	q	DF
117		
118	1.282	116
119	0.1056	116
120	0.7351	116

**121**	1.397	116
**122**	3.021	116
**123**	0.6776	116
**124**	2.024	116
**125**	8.638	116
**126**	1.229	116
**127**	0.4411	116
**128**	1.289	116
**129**	0.3586	116
**130**	1.289	116
**131**	1.333	116
**132**	1.177	116
**133**	0.5474	116
**134**	2.68	116
**135**	4.269	116
**136**	0.6049	116
**137**	3.233	116
**138**	9.802	116
**139**	0.0649	116
**140**	1.552	116
**141**	0.07961	116
**142**	0.8505	116
**143**	0.07961	116
**144**	2.615	116
**145**	0.6295	116
**146**	1.503	116
**147**	3.124	116
**148**	0.572	116
**149**	2.124	116
**150**	8.734	116

**151**	1.133	116
**152**	0.5326	116
**153**	1.189	116
**154**	0.2591	116
**155**	1.189	116
**156**	1.438	116
**157**	2.132	116
**158**	3.737	116
**159**	0.0575	116
**160**	2.717	116
**161**	9.305	116
**162**	0.5616	116
**163**	1.078	116
**164**	0.5957	116
**165**	0.3344	116
**166**	0.5957	116
**167**	2.068	116
**168**	1.661	116
**169**	2.075	116
**170**	0.7069	116
**171**	7.37	116
**172**	2.497	116
**173**	0.7689	116
**174**	2.606	116
**175**	1.676	116
**176**	2.606	116
**177**	0.06457	116
**178**	3.681	116
**179**	0.8807	116
**180**	5.693	116

**181**	3.956	116
**182**	2.202	116
**183**	4.115	116
**184**	3.207	116
**185**	4.115	116
**186**	1.724	116
**187**	2.663	116
**188**	9.253	116
**189**	0.6138	116
**190**	1.028	116
**191**	0.6499	116
**192**	0.2802	116
**193**	0.6499	116
**194**	2.01	116
**195**	6.37	116
**196**	3.025	116
**197**	1.356	116
**198**	3.143	116
**199**	2.261	116
**200**	3.143	116
**201**	0.7678	116
**202**	9.097	116
**203**	7.242	116
**204**	9.406	116
**205**	8.554	116
**206**	9.406	116
**207**	7.429	116
**208**	1.498	116
**209**	0.01117	116
**210**	0.8413	116

**211**	0.01117	116
**212**	2.438	116
**213**	1.554	116
**214**	0.7368	116
**215**	1.554	116
**216**	0.713	116
**217**	0.8824	116
**218**	0	116
**219**	2.545	116
**220**	0.8824	116
**221**	1.615	116
**222**	2.545	116

Data analyzed: NKA

Source of Variation	Degrees of Freedom	Sum of Squares	Mean square
Pre-treatment	2	138.2	69.09
Treatment	4	200.3	50.07
Interaction	8	218.7	27.34
Residual (error)	116	714.7	6.162
Total	130		

Does Pre-treatment have the same effect at all values of Treatment?

Interaction accounts for approximately 17.94% of the total variance.
F = 4.44. DFn=8 DFd=116
The P value = 0.0001
If there is no interaction overall, there is a 0.01% chance of randomly observing so much interaction in an experiment of this size. The interaction is considered extremely significant.

Since the interaction is statistically significant, the P values that follow for the row and column effects are difficult to interpret.

Does Pre-treatment affect the result?

Pre-treatment accounts for approximately 11.33% of the total variance.
F = 11.21. DFn=2 DFd=116
The P value is < 0.0001
If Pre-treatment has no effect overall, there is a less than 0.01% chance of randomly observing an effect this big (or bigger) in an experiment of this size. The effect is considered extremely significant.

Does Treatment affect the result?

Treatment accounts for approximately 16.43% of the total variance.
F = 8.13. DFn=4 DFd=116
The P value is < 0.0001
If Treatment has no effect overall, there is a less than 0.01% chance of randomly observing an this big (or bigger) in an experiment of this size. The effect is considered extremely significant.
