# Supplementary material for: RNA profiling identifies novel, photoperiod-history dependent markers associated with enhanced saltwater performance in juvenile Atlantic salmon
Source: PLoS One. 2020 Apr 8;15(4):e0227496. doi: 10.1371/journal.pone.0227496 (PMC7141700; doi:10.1371/journal.pone.0227496)
Supplement: S4 Table — Table shows the development of weight (g) and conditionu factor of the experimental groups in experiment 1. (PDF) [file pone.0227496.s005.pdf]

Experiment 1

Day		Weight (g)			Condition factor		
		LL	SP	SPLL	LL	SP	SPLL
1	Mean	49.48	-	-	1.229	-	-
	N	6	-	-	6	-	-
	SD	7.038	-	-	0.2444	-	-
32	Mean	48.5	55.33	-	1.308	1.322	-
	N	6	6	-	6	6	-
	SD	4.604	8.029	-	0.04799	0.03741	-
53	Mean	73.83	60.17	-	1.255	1.307	-
	N	6	6	-	6	6	-
	SD	11.06	6.601	-	0.03581	0.05045	-
68	Mean	87	63.58	65.58	1.29	1.278	1.395
	N	6	6	6	6	6	6
	SD	8.55	16.56	13.4	0.07972	0.1027	0.0661
89	Mean	91.47	70.23	85.38	1.252	1.259	1.243
	N	6	6	6	6	6	6
	SD	29.3	10.76	21.52	0.02426	0.05877	0.02863
110	Mean	91.33	78.42	81.67	1.252	1.301	1.111
	N	6	6	6	6	6	6
	SD	22.68	18.23	15.91	0.04977	0.05067	0.07973

2way ANOVA
Tabular results

1	Table Analyzed	Weight 2013				
2						
3	Two-way ANOVA	Ordinary				
4	Alpha	0.05				
5						
6	Source of Variation	% of total variation	P value	P value summary	Significant?	
7	Interaction	2.841	0.7834	ns	No	
8	Time	7.107	0.1259	ns	No	
9	Treatment	16.38	0.0109	*	Yes	
10						
11	ANOVA table	SS	DF	MS	F (DFn, DFd)	P value
12	Interaction	590.9	4	147.7	F (4, 45) = 0.4339	P=0.7834
13	Time	1478	2	739.1	F (2, 45) = 2.171	P=0.1259
14	Treatment	3408	2	1704	F (2, 45) = 5.004	P=0.0109
15	Residual	15321	45	340.5		
16						
17	Number of missing values	0				

2way ANOVA
Multiple comparisons

1	Within each row, compare columns (simple effects within rows)								
2									
3	Number of families	3							
4	Number of comparisons per family	3							
5	Alpha	0.05							
6									
7	Tukey's multiple comparisons test	Mean Diff.	95.00% CI of diff.	Significant?	Summary	Adjusted P Value			
8									
9	68								
10	LL vs. SP	23.42	-2.402 to 49.24	No	ns	0.0824			
11	LL vs. SPLL	21.42	-4.402 to 47.24	No	ns	0.1214			
12	SP vs. SPLL	-2	-27.82 to 23.82	No	ns	0.9808			
13									
14	89								
15	LL vs. SP	21.23	-4.586 to 47.05	No	ns	0.1256			
16	LL vs. SPLL	6.083	-19.74 to 31.9	No	ns	0.8362			
17	SP vs. SPLL	-15.15	-40.97 to 10.67	No	ns	0.3383			
18									
19	110								
20	LL vs. SP	12.92	-12.9 to 38.74	No	ns	0.4521			
21	LL vs. SPLL	9.667	-16.15 to 35.49	No	ns	0.6385			
22	SP vs. SPLL	-3.25	-29.07 to 22.57	No	ns	0.9500			
23									
24									
25	Test details	Mean 1	Mean 2	Mean Diff.	SE of diff.	N1	N2	q	DF
26									
27	68								
28	LL vs. SP	87	63.58	23.42	10.65	6	6	3.109	45
29	LL vs. SPLL	87	65.58	21.42	10.65	6	6	2.843	45
30	SP vs. SPLL	63.58	65.58	-2	10.65	6	6	0.2655	45

**2way ANOVA**<br>Multiple comparisons									
31									
32	89								
33	LL vs. SP	91.47	70.23	21.23	10.65	6	6	2.819	45
34	LL vs. SPLL	91.47	85.38	6.083	10.65	6	6	0.8076	45
35	SP vs. SPLL	70.23	85.38	-15.15	10.65	6	6	2.011	45
36									
37	110								
38	LL vs. SP	91.33	78.42	12.92	10.65	6	6	1.715	45
39	LL vs. SPLL	91.33	81.67	9.667	10.65	6	6	1.283	45
40	SP vs. SPLL	78.42	81.67	-3.25	10.65	6	6	0.4314	45

2way ANOVA
Tabular results

1	Table Analyzed	Condition factor 2013				
2						
3	Two-way ANOVA	Ordinary				
4	Alpha	0.05				
5						
6	Source of Variation	% of total variation	P value	P value summary	Significant?	
7	Interaction	35.57	<0.0001	****	Yes	
8	Time	20.95	0.0001	***	Yes	
9	Treatment	1.731	0.4009	ns	No	
10						
11	ANOVA table	SS	DF	MS	F (DFn, DFd)	P value
12	Interaction	0.1598	4	0.03996	F (4, 45) = 9.583	P<0.0001
13	Time	0.09413	2	0.04706	F (2, 45) = 11.29	P=0.0001
14	Treatment	0.00778	2	0.00389	F (2, 45) = 0.933	P=0.4009
15	Residual	0.1876	45	0.00417		
16						
17	Number of missing values	0				

2way ANOVA
Multiple comparisons

1	Within each row, compare columns (simple effects within rows)								
2									
3	Number of families	3							
4	Number of comparisons per family	3							
5	Alpha	0.05							
6									
7	Tukey's multiple comparisons test	Mean Diff.	95.00% CI of diff.	Significant?	Summary	Adjusted P Value			
8									
9	68								
10	LL vs. SP	0.01239	-0.07797 to 0.1027	No	ns	0.9411			
11	LL vs. SPLL	-0.1048	-0.1952 to -0.01447	Yes	*	0.0195			
12	SP vs. SPLL	-0.1172	-0.2076 to -0.02685	Yes	**	0.0081			
13									
14	89								
15	LL vs. SP	-0.006284	-0.09664 to 0.08407	No	ns	0.9845			
16	LL vs. SPLL	0.008936	-0.08142 to 0.09929	No	ns	0.9688			
17	SP vs. SPLL	0.01522	-0.07513 to 0.1056	No	ns	0.9124			
18									
19	110								
20	LL vs. SP	-0.04856	-0.1389 to 0.04179	No	ns	0.4012			
21	LL vs. SPLL	0.1416	0.05126 to 0.232	Yes	**	0.0012			
22	SP vs. SPLL	0.1902	0.09982 to 0.2805	Yes	****	<0.0001			
23									
24									
25	Test details	Mean 1	Mean 2	Mean Diff.	SE of diff.	N1	N2	q	DF
26									
27	68								
28	LL vs. SP	1.29	1.278	0.01239	0.03728	6	6	0.4698	45
29	LL vs. SPLL	1.29	1.395	-0.1048	0.03728	6	6	3.976	45
30	SP vs. SPLL	1.278	1.395	-0.1172	0.03728	6	6	4.446	45

2way ANOVA
Multiple comparisons

31									
32	89								
33	LL vs. SP	1.252	1.259	-0.006284	0.03728	6	6	0.2384	45
34	LL vs. SPLL	1.252	1.243	0.008936	0.03728	6	6	0.339	45
35	SP vs. SPLL	1.259	1.243	0.01522	0.03728	6	6	0.5774	45
36									
37	110								
38	LL vs. SP	1.252	1.301	-0.04856	0.03728	6	6	1.842	45
39	LL vs. SPLL	1.252	1.111	0.1416	0.03728	6	6	5.372	45
40	SP vs. SPLL	1.301	1.111	0.1902	0.03728	6	6	7.214	45
