# Supplementary material for: RNA profiling identifies novel, photoperiod-history dependent markers associated with enhanced saltwater performance in juvenile Atlantic salmon
Source: PLoS One. 2020 Apr 8;15(4):e0227496. doi: 10.1371/journal.pone.0227496 (PMC7141700; doi:10.1371/journal.pone.0227496)
Supplement: S5 Table — Table shows the development of weight (g) and condition factor of the experimental groups during the FW phase of experiment 2. (PDF) [file pone.0227496.s006.pdf]

Experiment 2

FW		Weight (g)				Condition factor			
Day		2WSP	4WSP	8WSP	SPC	2WSP	4WSP	8WSP	SPC
1	Mean	40.33	-	-	-	1.238921	-	-	-
	N	10	-	-	-	10	-	-	-
	SD	9.682751	-	-	-	0.224363	-	-	-
15	Mean	37.7	-	-	39.6	1.173656	-	-	1.230874
	N	10	-	-	10	10	-	-	10
	SD	7.513691	-	-	3.95671	0.044211	-	-	0.064401
29	Mean	-	42.85	-	38.5	-	1.219059	-	1.191846
	N	-	10	-	10	-	10	-	10
	SD	-	5.7399671	-	7.666667	-	0.033448	-	0.050016
43	Mean	40.15	-	-	39.7	1.156269	-	-	1.222724
	N		-	-	10	10	-	-	10
	SD	7.3938788	-	-	5.652925	0.114153	-	-	0.059581
58	Mean	-	55.4	52.35	44.25	-	1.242293	1.194456	1.186525
	N	-	10	10	10	-	10	10	10
	SD	-	6.5226102	6.5066206	4.3092278	-	0.077991	0.052454	0.085887
71	Mean	63.9	-	-	52.45	1.247538	-	-	1.326445
	N		-	-	10	10	-	-	10
	SD	11.117554	-	-	5.40807	0.074836	-	-	0.041075
86	Mean	-	66.6	54.2	52.8	-	1.183827	1.190506	1.247698
	N	-	10	10	10	-	10	10	10
	SD	-	10.107203	7.6055535	10.163114	-	0.038288	0.021213	0.032843
113	Mean	-	-	74.65	65.55	-	-	1.217498	1.2653336
	N	-	-	10	10	-	-	10	10
	SD	-	-	14.431928	11.129166	-	-	0.070139	0.073543

I

Experiment 2

SW		Weight (g)			Condition factor		
**Day**		**2WSP**	**4WSP**	**8WSP**	**2WSP**	**4WSP**	**8WSP**
**71**	**Mean**	67.16667	-	-	1.275637	-	-
	**N**	30	-	-	30	-	-
	**SD**	10.89712	-	-	0.0508	-	-
**86**	**Mean**	68.82759	69.4	-	1.205494	1.25668	-
	**N**	29	30	-	29	30	-
	**SD**	11.55907	8.680537	-	0.150763	0.05041	-
**100**	**Mean**	70.63333	73.8	-	1.186437	1.214933	-
	**N**	30	30	-	30	30	-
	**SD**	11.37142	10.23483	-	0.05927	0.062442	-
**113**	**Mean**	-	83.01667	73.9	-	1.236895	1.216394
	**N**	-	30	30	-	30	30
	**SD**	-	12.50344	11.76157	-	0.056315	0.041986
**128**	**Mean**	-	-	80.75	-	-	1.173613
	**N**	-	-	30	-	-	30
	**SD**	-	-	12.45457	-	-	0.049847
**142**	**Mean**	-	-	94.06667	-	-	1.180716
	**N**	-	-	30	-	-	30
	**SD**	-	-	15.12177	-	-	0.050576

2way ANOVA
Tabular results

1	Table Analyzed	Weigth FW grouped format				
2						
3	Two-way ANOVA	Ordinary				
4	Alpha	0.05				
5						
6	Source of Variation	% of total variation	P value	P value summary	Significant?	
7	Interaction	3.637	0.0835	ns	No	
8	Treatment	48.66	<0.0001	****	Yes	
9	Pretreatment	13.15	<0.0001	****	Yes	
10						
11	ANOVA table	SS	DF	MS	F (DFn, DFd)	P value
12	Interaction	1160	10	116	F (10, 162) = 1.705	P=0.0835
13	Treatment	15515	5	3103	F (5, 162) = 45.63	P<0.0001
14	Pretreatment	4194	2	2097	F (2, 162) = 30.84	P<0.0001
15	Residual	11016	162	68		
16						
17	Number of missing values	0				

2way ANOVA
Multiple comparisons

1	Compare cell means regardless of rows and columns					
2						
3	Number of families	1				
4	Number of comparisons per family	153				
5	Alpha	0.05				
6						
7	Tukey's multiple comparisons test	Mean Diff.	95.00% CI of diff.	Significant?	Summary	Adjusted P Value
8						
9	First long day:2 week winter vs. First long day:4 week winter	-5.15	-18.23 to 7.927	No	ns	0.9952
10	First long day:2 week winter vs. First long day:8 week winter	-14.65	-27.73 to -1.573	Yes	*	0.0124
11	First long day:2 week winter vs. 4 weeks post winter:2 week winter	-2.45	-15.53 to 10.63	No	ns	>0.9999
12	First long day:2 week winter vs. 4 weeks post winter:4 week winter	-17.7	-30.78 to -4.623	Yes	***	0.0005
13	First long day:2 week winter vs. 4 weeks post winter:8 week winter	-16.5	-29.58 to -3.423	Yes	**	0.0019
14	First long day:2 week winter vs. 8 weeks post winter:2 week winter	-26.2	-39.28 to -13.12	Yes	****	<0.0001
15	First long day:2 week winter vs. 8 weeks post winter:4 week winter	-28.9	-41.98 to -15.82	Yes	****	<0.0001
16	First long day:2 week winter vs. 8 weeks post winter:8 week winter	-36.95	-50.03 to -23.87	Yes	****	<0.0001
17	First long day:2 week winter vs. SPC -first long day:2 week winter	-1.9	-14.98 to 11.18	No	ns	>0.9999
18	First long day:2 week winter vs. SPC -first long day:4 week winter	-0.8	-13.88 to 12.28	No	ns	>0.9999
19	First long day:2 week winter vs. SPC -first long day:8 week winter	-6.55	-19.63 to 6.527	No	ns	0.9469
20	First long day:2 week winter vs. SPC -4 weeks post:2 week winter	-2	-15.08 to 11.08	No	ns	>0.9999
21	First long day:2 week winter vs. SPC -4 weeks post:4 week winter	-6.55	-19.63 to 6.527	No	ns	0.9469
22	First long day:2 week winter vs. SPC -4 weeks post:8 week winter	-15.1	-28.18 to -2.023	Yes	**	0.0080
23	First long day:2 week winter vs. SPC -8 weeks post:2 week winter	-14.75	-27.83 to -1.673	Yes	*	0.0113
24	First long day:2 week winter vs. SPC -8 weeks post:4 week winter	-15.1	-28.18 to -2.023	Yes	**	0.0080
25	First long day:2 week winter vs. SPC -8 weeks post:8 week winter	-27.85	-40.93 to -14.77	Yes	****	<0.0001
26	First long day:4 week winter vs. First long day:8 week winter	-9.5	-22.58 to 3.577	No	ns	0.4774
27	First long day:4 week winter vs. 4 weeks post winter:2 week winter	2.7	-10.38 to 15.78	No	ns	>0.9999
28	First long day:4 week winter vs. 4 weeks post winter:4 week winter	-12.55	-25.63 to 0.5275	No	ns	0.0760
29	First long day:4 week winter vs. 4 weeks post winter:8 week winter	-11.35	-24.43 to 1.727	No	ns	0.1779
30	First long day:4 week winter vs. 8 weeks post winter:2 week winter	-21.05	-34.13 to -7.973	Yes	****	<0.0001

2way ANOVA<br>Multiple comparisons							
31	First long day:4 week winter vs. 8 weeks post winter:4 week winter	-23.75	-36.83 to -10.67	Yes	****	<0.0001	
32	First long day:4 week winter vs. 8 weeks post winter:8 week winter	-31.8	-44.88 to -18.72	Yes	****	<0.0001	
33	First long day:4 week winter vs. SPC -first long day:2 week winter	3.25	-9.827 to 16.33	No	ns	>0.9999	
34	First long day:4 week winter vs. SPC -first long day:4 week winter	4.35	-8.727 to 17.43	No	ns	0.9994	
35	First long day:4 week winter vs. SPC -first long day:8 week winter	-1.4	-14.48 to 11.68	No	ns	>0.9999	
36	First long day:4 week winter vs. SPC -4 weeks post:2 week winter	3.15	-9.927 to 16.23	No	ns	>0.9999	
37	First long day:4 week winter vs. SPC -4 weeks post:4 week winter	-1.4	-14.48 to 11.68	No	ns	>0.9999	
38	First long day:4 week winter vs. SPC -4 weeks post:8 week winter	-9.95	-23.03 to 3.127	No	ns	0.3905	
39	First long day:4 week winter vs. SPC -8 weeks post:2 week winter	-9.6	-22.68 to 3.477	No	ns	0.4576	
40	First long day:4 week winter vs. SPC -8 weeks post:4 week winter	-9.95	-23.03 to 3.127	No	ns	0.3905	
41	First long day:4 week winter vs. SPC -8 weeks post:8 week winter	-22.7	-35.78 to -9.623	Yes	****	<0.0001	
42	First long day:8 week winter vs. 4 weeks post winter:2 week winter	12.2	-0.8775 to 25.28	No	ns	0.0989	
43	First long day:8 week winter vs. 4 weeks post winter:4 week winter	-3.05	-16.13 to 10.03	No	ns	>0.9999	
44	First long day:8 week winter vs. 4 weeks post winter:8 week winter	-1.85	-14.93 to 11.23	No	ns	>0.9999	
45	First long day:8 week winter vs. 8 weeks post winter:2 week winter	-11.55	-24.63 to 1.527	No	ns	0.1560	
46	First long day:8 week winter vs. 8 weeks post winter:4 week winter	-14.25	-27.33 to -1.173	Yes	*	0.0180	
47	First long day:8 week winter vs. 8 weeks post winter:8 week winter	-22.3	-35.38 to -9.223	Yes	****	<0.0001	
48	First long day:8 week winter vs. SPC -first long day:2 week winter	12.75	-0.3275 to 25.83	No	ns	0.0651	
49	First long day:8 week winter vs. SPC -first long day:4 week winter	13.85	0.7725 to 26.93	Yes	*	0.0259	
50	First long day:8 week winter vs. SPC -first long day:8 week winter	8.1	-4.977 to 21.18	No	ns	0.7530	
51	First long day:8 week winter vs. SPC -4 weeks post:2 week winter	12.65	-0.4275 to 25.73	No	ns	0.0704	
52	First long day:8 week winter vs. SPC -4 weeks post:4 week winter	8.1	-4.977 to 21.18	No	ns	0.7530	
53	First long day:8 week winter vs. SPC -4 weeks post:8 week winter	-0.45	-13.53 to 12.63	No	ns	>0.9999	
54	First long day:8 week winter vs. SPC -8 weeks post:2 week winter	-0.1	-13.18 to 12.98	No	ns	>0.9999	
55	First long day:8 week winter vs. SPC -8 weeks post:4 week winter	-0.45	-13.53 to 12.63	No	ns	>0.9999	
56	First long day:8 week winter vs. SPC -8 weeks post:8 week winter	-13.2	-26.28 to -0.1225	Yes	*	0.0452	
57	4 weeks post winter:2 week winter vs. 4 weeks post winter:4 week winter	-15.25	-28.33 to -2.173	Yes	**	0.0069	
58	4 weeks post winter:2 week winter vs. 4 weeks post winter:8 week winter	-14.05	-27.13 to -0.9725	Yes	*	0.0216	
59	4 weeks post winter:2 week winter vs. 8 weeks post winter:2 week winter	-23.75	-36.83 to -10.67	Yes	****	<0.0001	
60	4 weeks post winter:2 week winter vs. 8 weeks post winter:4 week winter	-26.45	-39.53 to -13.37	Yes	****	<0.0001	

**2way ANOVA**<br>Multiple comparisons							
61	4 weeks post winter:2 week winter vs. 8 weeks post winter:8 week winter	-34.5	-47.58 to -21.42	Yes	****	<0.0001	
62	4 weeks post winter:2 week winter vs. SPC -first long day:2 week winter	0.55	-12.53 to 13.63	No	ns	>0.9999	
63	4 weeks post winter:2 week winter vs. SPC -first long day:4 week winter	1.65	-11.43 to 14.73	No	ns	>0.9999	
64	4 weeks post winter:2 week winter vs. SPC -first long day:8 week winter	-4.1	-17.18 to 8.977	No	ns	0.9997	
65	4 weeks post winter:2 week winter vs. SPC -4 weeks post:2 week winter	0.45	-12.63 to 13.53	No	ns	>0.9999	
66	4 weeks post winter:2 week winter vs. SPC -4 weeks post:4 week winter	-4.1	-17.18 to 8.977	No	ns	0.9997	
67	4 weeks post winter:2 week winter vs. SPC -4 weeks post:8 week winter	-12.65	-25.73 to 0.4275	No	ns	0.0704	
68	4 weeks post winter:2 week winter vs. SPC -8 weeks post:2 week winter	-12.3	-25.38 to 0.7775	No	ns	0.0919	
69	4 weeks post winter:2 week winter vs. SPC -8 weeks post:4 week winter	-12.65	-25.73 to 0.4275	No	ns	0.0704	
70	4 weeks post winter:2 week winter vs. SPC -8 weeks post:8 week winter	-25.4	-38.48 to -12.32	Yes	****	<0.0001	
71	4 weeks post winter:4 week winter vs. 4 weeks post winter:8 week winter	1.2	-11.88 to 14.28	No	ns	>0.9999	
72	4 weeks post winter:4 week winter vs. 8 weeks post winter:2 week winter	-8.5	-21.58 to 4.577	No	ns	0.6786	
73	4 weeks post winter:4 week winter vs. 8 weeks post winter:4 week winter	-11.2	-24.28 to 1.877	No	ns	0.1957	
74	4 weeks post winter:4 week winter vs. 8 weeks post winter:8 week winter	-19.25	-32.33 to -6.173	Yes	****	<0.0001	
75	4 weeks post winter:4 week winter vs. SPC -first long day:2 week winter	15.8	2.723 to 28.88	Yes	**	0.0040	
76	4 weeks post winter:4 week winter vs. SPC -first long day:4 week winter	16.9	3.823 to 29.98	Yes	**	0.0012	
77	4 weeks post winter:4 week winter vs. SPC -first long day:8 week winter	11.15	-1.927 to 24.23	No	ns	0.2019	
78	4 weeks post winter:4 week winter vs. SPC -4 weeks post:2 week winter	15.7	2.623 to 28.78	Yes	**	0.0044	
79	4 weeks post winter:4 week winter vs. SPC -4 weeks post:4 week winter	11.15	-1.927 to 24.23	No	ns	0.2019	
80	4 weeks post winter:4 week winter vs. SPC -4 weeks post:8 week winter	2.6	-10.48 to 15.68	No	ns	>0.9999	
81	4 weeks post winter:4 week winter vs. SPC -8 weeks post:2 week winter	2.95	-10.13 to 16.03	No	ns	>0.9999	
82	4 weeks post winter:4 week winter vs. SPC -8 weeks post:4 week winter	2.6	-10.48 to 15.68	No	ns	>0.9999	
83	4 weeks post winter:4 week winter vs. SPC -8 weeks post:8 week winter	-10.15	-23.23 to 2.927	No	ns	0.3542	
84	4 weeks post winter:8 week winter vs. 8 weeks post winter:2 week winter	-9.7	-22.78 to 3.377	No	ns	0.4380	
85	4 weeks post winter:8 week winter vs. 8 weeks post winter:4 week winter	-12.4	-25.48 to 0.6775	No	ns	0.0852	
86	4 weeks post winter:8 week winter vs. 8 weeks post winter:8 week winter	-20.45	-33.53 to -7.373	Yes	****	<0.0001	
87	4 weeks post winter:8 week winter vs. SPC -first long day:2 week winter	14.6	1.523 to 27.68	Yes	*	0.0130	
88	4 weeks post winter:8 week winter vs. SPC -first long day:4 week winter	15.7	2.623 to 28.78	Yes	**	0.0044	
89	4 weeks post winter:8 week winter vs. SPC -first long day:8 week winter	9.95	-3.127 to 23.03	No	ns	0.3905	
90	4 weeks post winter:8 week winter vs. SPC -4 weeks post:2 week winter	14.5	1.423 to 27.58	Yes	*	0.0143	

2way ANOVA<br>Multiple comparisons							
91	4 weeks post winter:8 week winter vs. SPC -4 weeks post:4 week winter	9.95	-3.127 to 23.03	No	ns	0.3905	
92	4 weeks post winter:8 week winter vs. SPC -4 weeks post:8 week winter	1.4	-11.68 to 14.48	No	ns	>0.9999	
93	4 weeks post winter:8 week winter vs. SPC -8 weeks post:2 week winter	1.75	-11.33 to 14.83	No	ns	>0.9999	
94	4 weeks post winter:8 week winter vs. SPC -8 weeks post:4 week winter	1.4	-11.68 to 14.48	No	ns	>0.9999	
95	4 weeks post winter:8 week winter vs. SPC -8 weeks post:8 week winter	-11.35	-24.43 to 1.727	No	ns	0.1779	
96	8 weeks post winter:2 week winter vs. 8 weeks post winter:4 week winter	-2.7	-15.78 to 10.38	No	ns	>0.9999	
97	8 weeks post winter:2 week winter vs. 8 weeks post winter:8 week winter	-10.75	-23.83 to 2.327	No	ns	0.2564	
98	8 weeks post winter:2 week winter vs. SPC -first long day:2 week winter	24.3	11.22 to 37.38	Yes	****	<0.0001	
99	8 weeks post winter:2 week winter vs. SPC -first long day:4 week winter	25.4	12.32 to 38.48	Yes	****	<0.0001	
100	8 weeks post winter:2 week winter vs. SPC -first long day:8 week winter	19.65	6.573 to 32.73	Yes	****	<0.0001	
101	8 weeks post winter:2 week winter vs. SPC -4 weeks post:2 week winter	24.2	11.12 to 37.28	Yes	****	<0.0001	
102	8 weeks post winter:2 week winter vs. SPC -4 weeks post:4 week winter	19.65	6.573 to 32.73	Yes	****	<0.0001	
103	8 weeks post winter:2 week winter vs. SPC -4 weeks post:8 week winter	11.1	-1.977 to 24.18	No	ns	0.2082	
104	8 weeks post winter:2 week winter vs. SPC -8 weeks post:2 week winter	11.45	-1.627 to 24.53	No	ns	0.1667	
105	8 weeks post winter:2 week winter vs. SPC -8 weeks post:4 week winter	11.1	-1.977 to 24.18	No	ns	0.2082	
106	8 weeks post winter:2 week winter vs. SPC -8 weeks post:8 week winter	-1.65	-14.73 to 11.43	No	ns	>0.9999	
107	8 weeks post winter:4 week winter vs. 8 weeks post winter:8 week winter	-8.05	-21.13 to 5.027	No	ns	0.7618	
108	8 weeks post winter:4 week winter vs. SPC -first long day:2 week winter	27	13.92 to 40.08	Yes	****	<0.0001	
109	8 weeks post winter:4 week winter vs. SPC -first long day:4 week winter	28.1	15.02 to 41.18	Yes	****	<0.0001	
110	8 weeks post winter:4 week winter vs. SPC -first long day:8 week winter	22.35	9.273 to 35.43	Yes	****	<0.0001	
111	8 weeks post winter:4 week winter vs. SPC -4 weeks post:2 week winter	26.9	13.82 to 39.98	Yes	****	<0.0001	
112	8 weeks post winter:4 week winter vs. SPC -4 weeks post:4 week winter	22.35	9.273 to 35.43	Yes	****	<0.0001	
113	8 weeks post winter:4 week winter vs. SPC -4 weeks post:8 week winter	13.8	0.7225 to 26.88	Yes	*	0.0270	
114	8 weeks post winter:4 week winter vs. SPC -8 weeks post:2 week winter	14.15	1.073 to 27.23	Yes	*	0.0197	
115	8 weeks post winter:4 week winter vs. SPC -8 weeks post:4 week winter	13.8	0.7225 to 26.88	Yes	*	0.0270	
116	8 weeks post winter:4 week winter vs. SPC -8 weeks post:8 week winter	1.05	-12.03 to 14.13	No	ns	>0.9999	
117	8 weeks post winter:8 week winter vs. SPC -first long day:2 week winter	35.05	21.97 to 48.13	Yes	****	<0.0001	
118	8 weeks post winter:8 week winter vs. SPC -first long day:4 week winter	36.15	23.07 to 49.23	Yes	****	<0.0001	
119	8 weeks post winter:8 week winter vs. SPC -first long day:8 week winter	30.4	17.32 to 43.48	Yes	****	<0.0001	
120	8 weeks post winter:8 week winter vs. SPC -4 weeks post:2 week winter	34.95	21.87 to 48.03	Yes	****	<0.0001	

2way ANOVA<br>Multiple comparisons							
121	8 weeks post winter:8 week winter vs. SPC -4 weeks post:4 week winter	30.4	17.32 to 43.48	Yes	****	<0.0001	
122	8 weeks post winter:8 week winter vs. SPC -4 weeks post:8 week winter	21.85	8.773 to 34.93	Yes	****	<0.0001	
123	8 weeks post winter:8 week winter vs. SPC -8 weeks post:2 week winter	22.2	9.123 to 35.28	Yes	****	<0.0001	
124	8 weeks post winter:8 week winter vs. SPC -8 weeks post:4 week winter	21.85	8.773 to 34.93	Yes	****	<0.0001	
125	8 weeks post winter:8 week winter vs. SPC -8 weeks post:8 week winter	9.1	-3.977 to 22.18	No	ns	0.5583	
126	SPC -first long day:2 week winter vs. SPC -first long day:4 week winter	1.1	-11.98 to 14.18	No	ns	>0.9999	
127	SPC -first long day:2 week winter vs. SPC -first long day:8 week winter	-4.65	-17.73 to 8.427	No	ns	0.9986	
128	SPC -first long day:2 week winter vs. SPC -4 weeks post:2 week winter	-0.1	-13.18 to 12.98	No	ns	>0.9999	
129	SPC -first long day:2 week winter vs. SPC -4 weeks post:4 week winter	-4.65	-17.73 to 8.427	No	ns	0.9986	
130	SPC -first long day:2 week winter vs. SPC -4 weeks post:8 week winter	-13.2	-26.28 to -0.1225	Yes	*	0.0452	
131	SPC -first long day:2 week winter vs. SPC -8 weeks post:2 week winter	-12.85	-25.93 to 0.2275	No	ns	0.0601	
132	SPC -first long day:2 week winter vs. SPC -8 weeks post:4 week winter	-13.2	-26.28 to -0.1225	Yes	*	0.0452	
133	SPC -first long day:2 week winter vs. SPC -8 weeks post:8 week winter	-25.95	-39.03 to -12.87	Yes	****	<0.0001	
134	SPC -first long day:4 week winter vs. SPC -first long day:8 week winter	-5.75	-18.83 to 7.327	No	ns	0.9844	
135	SPC -first long day:4 week winter vs. SPC -4 weeks post:2 week winter	-1.2	-14.28 to 11.88	No	ns	>0.9999	
136	SPC -first long day:4 week winter vs. SPC -4 weeks post:4 week winter	-5.75	-18.83 to 7.327	No	ns	0.9844	
137	SPC -first long day:4 week winter vs. SPC -4 weeks post:8 week winter	-14.3	-27.38 to -1.223	Yes	*	0.0172	
138	SPC -first long day:4 week winter vs. SPC -8 weeks post:2 week winter	-13.95	-27.03 to -0.8725	Yes	*	0.0237	
139	SPC -first long day:4 week winter vs. SPC -8 weeks post:4 week winter	-14.3	-27.38 to -1.223	Yes	*	0.0172	
140	SPC -first long day:4 week winter vs. SPC -8 weeks post:8 week winter	-27.05	-40.13 to -13.97	Yes	****	<0.0001	
141	SPC -first long day:8 week winter vs. SPC -4 weeks post:2 week winter	4.55	-8.527 to 17.63	No	ns	0.9989	
142	SPC -first long day:8 week winter vs. SPC -4 weeks post:4 week winter	0	-13.08 to 13.08	No	ns	>0.9999	
143	SPC -first long day:8 week winter vs. SPC -4 weeks post:8 week winter	-8.55	-21.63 to 4.527	No	ns	0.6688	
144	SPC -first long day:8 week winter vs. SPC -8 weeks post:2 week winter	-8.2	-21.28 to 4.877	No	ns	0.7351	
145	SPC -first long day:8 week winter vs. SPC -8 weeks post:4 week winter	-8.55	-21.63 to 4.527	No	ns	0.6688	
146	SPC -first long day:8 week winter vs. SPC -8 weeks post:8 week winter	-21.3	-34.38 to -8.223	Yes	****	<0.0001	
147	SPC -4 weeks post:2 week winter vs. SPC -4 weeks post:4 week winter	-4.55	-17.63 to 8.527	No	ns	0.9989	
148	SPC -4 weeks post:2 week winter vs. SPC -4 weeks post:8 week winter	-13.1	-26.18 to -0.0225	Yes	*	0.0491	
149	SPC -4 weeks post:2 week winter vs. SPC -8 weeks post:2 week winter	-12.75	-25.83 to 0.3275	No	ns	0.0651	
150	SPC -4 weeks post:2 week winter vs. SPC -8 weeks post:4 week winter	-13.1	-26.18 to -0.0225	Yes	*	0.0491	

2way ANOVA<br>Multiple comparisons							
151	SPC -4 weeks post:2 week winter vs. SPC -8 weeks post:8 week winter	-25.85	-38.93 to -12.77	Yes	****	<0.0001	
152	SPC -4 weeks post:4 week winter vs. SPC -4 weeks post:8 week winter	-8.55	-21.63 to 4.527	No	ns	0.6688	
153	SPC -4 weeks post:4 week winter vs. SPC -8 weeks post:2 week winter	-8.2	-21.28 to 4.877	No	ns	0.7351	
154	SPC -4 weeks post:4 week winter vs. SPC -8 weeks post:4 week winter	-8.55	-21.63 to 4.527	No	ns	0.6688	
155	SPC -4 weeks post:4 week winter vs. SPC -8 weeks post:8 week winter	-21.3	-34.38 to -8.223	Yes	****	<0.0001	
156	SPC -4 weeks post:8 week winter vs. SPC -8 weeks post:2 week winter	0.35	-12.73 to 13.43	No	ns	>0.9999	
157	SPC -4 weeks post:8 week winter vs. SPC -8 weeks post:4 week winter	0	-13.08 to 13.08	No	ns	>0.9999	
158	SPC -4 weeks post:8 week winter vs. SPC -8 weeks post:8 week winter	-12.75	-25.83 to 0.3275	No	ns	0.0651	
159	SPC -8 weeks post:2 week winter vs. SPC -8 weeks post:4 week winter	-0.35	-13.43 to 12.73	No	ns	>0.9999	
160	SPC -8 weeks post:2 week winter vs. SPC -8 weeks post:8 week winter	-13.1	-26.18 to -0.0225	Yes	*	0.0491	
161	SPC -8 weeks post:4 week winter vs. SPC -8 weeks post:8 week winter	-12.75	-25.83 to 0.3275	No	ns	0.0651	
162							
163							
164	Test details	Mean 1	Mean 2	Mean Diff.	SE of diff.	N1	N2
165							
166	First long day:2 week winter vs. First long day:4 week winter	37.7	42.85	-5.15	3.688	10	10
167	First long day:2 week winter vs. First long day:8 week winter	37.7	52.35	-14.65	3.688	10	10
168	First long day:2 week winter vs. 4 weeks post winter:2 week winter	37.7	40.15	-2.45	3.688	10	10
169	First long day:2 week winter vs. 4 weeks post winter:4 week winter	37.7	55.4	-17.7	3.688	10	10
170	First long day:2 week winter vs. 4 weeks post winter:8 week winter	37.7	54.2	-16.5	3.688	10	10
171	First long day:2 week winter vs. 8 weeks post winter:2 week winter	37.7	63.9	-26.2	3.688	10	10
172	First long day:2 week winter vs. 8 weeks post winter:4 week winter	37.7	66.6	-28.9	3.688	10	10
173	First long day:2 week winter vs. 8 weeks post winter:8 week winter	37.7	74.65	-36.95	3.688	10	10
174	First long day:2 week winter vs. SPC -first long day:2 week winter	37.7	39.6	-1.9	3.688	10	10
175	First long day:2 week winter vs. SPC -first long day:4 week winter	37.7	38.5	-0.8	3.688	10	10
176	First long day:2 week winter vs. SPC -first long day:8 week winter	37.7	44.25	-6.55	3.688	10	10
177	First long day:2 week winter vs. SPC -4 weeks post:2 week winter	37.7	39.7	-2	3.688	10	10
178	First long day:2 week winter vs. SPC -4 weeks post:4 week winter	37.7	44.25	-6.55	3.688	10	10
179	First long day:2 week winter vs. SPC -4 weeks post:8 week winter	37.7	52.8	-15.1	3.688	10	10
180	First long day:2 week winter vs. SPC -8 weeks post:2 week winter	37.7	52.45	-14.75	3.688	10	10

2way ANOVA<br>Multiple comparisons							
181	First long day:2 week winter vs. SPC -8 weeks post:4 week winter	37.7	52.8	-15.1	3.688	10	10
182	First long day:2 week winter vs. SPC -8 weeks post:8 week winter	37.7	65.55	-27.85	3.688	10	10
183	First long day:4 week winter vs. First long day:8 week winter	42.85	52.35	-9.5	3.688	10	10
184	First long day:4 week winter vs. 4 weeks post winter:2 week winter	42.85	40.15	2.7	3.688	10	10
185	First long day:4 week winter vs. 4 weeks post winter:4 week winter	42.85	55.4	-12.55	3.688	10	10
186	First long day:4 week winter vs. 4 weeks post winter:8 week winter	42.85	54.2	-11.35	3.688	10	10
187	First long day:4 week winter vs. 8 weeks post winter:2 week winter	42.85	63.9	-21.05	3.688	10	10
188	First long day:4 week winter vs. 8 weeks post winter:4 week winter	42.85	66.6	-23.75	3.688	10	10
189	First long day:4 week winter vs. 8 weeks post winter:8 week winter	42.85	74.65	-31.8	3.688	10	10
190	First long day:4 week winter vs. SPC -first long day:2 week winter	42.85	39.6	3.25	3.688	10	10
191	First long day:4 week winter vs. SPC -first long day:4 week winter	42.85	38.5	4.35	3.688	10	10
192	First long day:4 week winter vs. SPC -first long day:8 week winter	42.85	44.25	-1.4	3.688	10	10
193	First long day:4 week winter vs. SPC -4 weeks post:2 week winter	42.85	39.7	3.15	3.688	10	10
194	First long day:4 week winter vs. SPC -4 weeks post:4 week winter	42.85	44.25	-1.4	3.688	10	10
195	First long day:4 week winter vs. SPC -4 weeks post:8 week winter	42.85	52.8	-9.95	3.688	10	10
196	First long day:4 week winter vs. SPC -8 weeks post:2 week winter	42.85	52.45	-9.6	3.688	10	10
197	First long day:4 week winter vs. SPC -8 weeks post:4 week winter	42.85	52.8	-9.95	3.688	10	10
198	First long day:4 week winter vs. SPC -8 weeks post:8 week winter	42.85	65.55	-22.7	3.688	10	10
199	First long day:8 week winter vs. 4 weeks post winter:2 week winter	52.35	40.15	12.2	3.688	10	10
200	First long day:8 week winter vs. 4 weeks post winter:4 week winter	52.35	55.4	-3.05	3.688	10	10
201	First long day:8 week winter vs. 4 weeks post winter:8 week winter	52.35	54.2	-1.85	3.688	10	10
202	First long day:8 week winter vs. 8 weeks post winter:2 week winter	52.35	63.9	-11.55	3.688	10	10
203	First long day:8 week winter vs. 8 weeks post winter:4 week winter	52.35	66.6	-14.25	3.688	10	10
204	First long day:8 week winter vs. 8 weeks post winter:8 week winter	52.35	74.65	-22.3	3.688	10	10
205	First long day:8 week winter vs. SPC -first long day:2 week winter	52.35	39.6	12.75	3.688	10	10
206	First long day:8 week winter vs. SPC -first long day:4 week winter	52.35	38.5	13.85	3.688	10	10
207	First long day:8 week winter vs. SPC -first long day:8 week winter	52.35	44.25	8.1	3.688	10	10
208	First long day:8 week winter vs. SPC -4 weeks post:2 week winter	52.35	39.7	12.65	3.688	10	10
209	First long day:8 week winter vs. SPC -4 weeks post:4 week winter	52.35	44.25	8.1	3.688	10	10
210	First long day:8 week winter vs. SPC -4 weeks post:8 week winter	52.35	52.8	-0.45	3.688	10	10

2way ANOVA<br>Multiple comparisons							
211	First long day:8 week winter vs. SPC -8 weeks post:2 week winter	52.35	52.45	-0.1	3.688	10	10
212	First long day:8 week winter vs. SPC -8 weeks post:4 week winter	52.35	52.8	-0.45	3.688	10	10
213	First long day:8 week winter vs. SPC -8 weeks post:8 week winter	52.35	65.55	-13.2	3.688	10	10
214	4 weeks post winter:2 week winter vs. 4 weeks post winter:4 week winter	40.15	55.4	-15.25	3.688	10	10
215	4 weeks post winter:2 week winter vs. 4 weeks post winter:8 week winter	40.15	54.2	-14.05	3.688	10	10
216	4 weeks post winter:2 week winter vs. 8 weeks post winter:2 week winter	40.15	63.9	-23.75	3.688	10	10
217	4 weeks post winter:2 week winter vs. 8 weeks post winter:4 week winter	40.15	66.6	-26.45	3.688	10	10
218	4 weeks post winter:2 week winter vs. 8 weeks post winter:8 week winter	40.15	74.65	-34.5	3.688	10	10
219	4 weeks post winter:2 week winter vs. SPC -first long day:2 week winter	40.15	39.6	0.55	3.688	10	10
220	4 weeks post winter:2 week winter vs. SPC -first long day:4 week winter	40.15	38.5	1.65	3.688	10	10
221	4 weeks post winter:2 week winter vs. SPC -first long day:8 week winter	40.15	44.25	-4.1	3.688	10	10
222	4 weeks post winter:2 week winter vs. SPC -4 weeks post:2 week winter	40.15	39.7	0.45	3.688	10	10
223	4 weeks post winter:2 week winter vs. SPC -4 weeks post:4 week winter	40.15	44.25	-4.1	3.688	10	10
224	4 weeks post winter:2 week winter vs. SPC -4 weeks post:8 week winter	40.15	52.8	-12.65	3.688	10	10
225	4 weeks post winter:2 week winter vs. SPC -8 weeks post:2 week winter	40.15	52.45	-12.3	3.688	10	10
226	4 weeks post winter:2 week winter vs. SPC -8 weeks post:4 week winter	40.15	52.8	-12.65	3.688	10	10
227	4 weeks post winter:2 week winter vs. SPC -8 weeks post:8 week winter	40.15	65.55	-25.4	3.688	10	10
228	4 weeks post winter:4 week winter vs. 4 weeks post winter:8 week winter	55.4	54.2	1.2	3.688	10	10
229	4 weeks post winter:4 week winter vs. 8 weeks post winter:2 week winter	55.4	63.9	-8.5	3.688	10	10
230	4 weeks post winter:4 week winter vs. 8 weeks post winter:4 week winter	55.4	66.6	-11.2	3.688	10	10
231	4 weeks post winter:4 week winter vs. 8 weeks post winter:8 week winter	55.4	74.65	-19.25	3.688	10	10
232	4 weeks post winter:4 week winter vs. SPC -first long day:2 week winter	55.4	39.6	15.8	3.688	10	10
233	4 weeks post winter:4 week winter vs. SPC -first long day:4 week winter	55.4	38.5	16.9	3.688	10	10
234	4 weeks post winter:4 week winter vs. SPC -first long day:8 week winter	55.4	44.25	11.15	3.688	10	10
235	4 weeks post winter:4 week winter vs. SPC -4 weeks post:2 week winter	55.4	39.7	15.7	3.688	10	10
236	4 weeks post winter:4 week winter vs. SPC -4 weeks post:4 week winter	55.4	44.25	11.15	3.688	10	10
237	4 weeks post winter:4 week winter vs. SPC -4 weeks post:8 week winter	55.4	52.8	2.6	3.688	10	10
238	4 weeks post winter:4 week winter vs. SPC -8 weeks post:2 week winter	55.4	52.45	2.95	3.688	10	10
239	4 weeks post winter:4 week winter vs. SPC -8 weeks post:4 week winter	55.4	52.8	2.6	3.688	10	10
240	4 weeks post winter:4 week winter vs. SPC -8 weeks post:8 week winter	55.4	65.55	-10.15	3.688	10	10

2way ANOVA<br>Multiple comparisons							
241	4 weeks post winter:8 week winter vs. 8 weeks post winter:2 week winter	54.2	63.9	-9.7	3.688	10	10
242	4 weeks post winter:8 week winter vs. 8 weeks post winter:4 week winter	54.2	66.6	-12.4	3.688	10	10
243	4 weeks post winter:8 week winter vs. 8 weeks post winter:8 week winter	54.2	74.65	-20.45	3.688	10	10
244	4 weeks post winter:8 week winter vs. SPC -first long day:2 week winter	54.2	39.6	14.6	3.688	10	10
245	4 weeks post winter:8 week winter vs. SPC -first long day:4 week winter	54.2	38.5	15.7	3.688	10	10
246	4 weeks post winter:8 week winter vs. SPC -first long day:8 week winter	54.2	44.25	9.95	3.688	10	10
247	4 weeks post winter:8 week winter vs. SPC -4 weeks post:2 week winter	54.2	39.7	14.5	3.688	10	10
248	4 weeks post winter:8 week winter vs. SPC -4 weeks post:4 week winter	54.2	44.25	9.95	3.688	10	10
249	4 weeks post winter:8 week winter vs. SPC -4 weeks post:8 week winter	54.2	52.8	1.4	3.688	10	10
250	4 weeks post winter:8 week winter vs. SPC -8 weeks post:2 week winter	54.2	52.45	1.75	3.688	10	10
251	4 weeks post winter:8 week winter vs. SPC -8 weeks post:4 week winter	54.2	52.8	1.4	3.688	10	10
252	4 weeks post winter:8 week winter vs. SPC -8 weeks post:8 week winter	54.2	65.55	-11.35	3.688	10	10
253	8 weeks post winter:2 week winter vs. 8 weeks post winter:4 week winter	63.9	66.6	-2.7	3.688	10	10
254	8 weeks post winter:2 week winter vs. 8 weeks post winter:8 week winter	63.9	74.65	-10.75	3.688	10	10
255	8 weeks post winter:2 week winter vs. SPC -first long day:2 week winter	63.9	39.6	24.3	3.688	10	10
256	8 weeks post winter:2 week winter vs. SPC -first long day:4 week winter	63.9	38.5	25.4	3.688	10	10
257	8 weeks post winter:2 week winter vs. SPC -first long day:8 week winter	63.9	44.25	19.65	3.688	10	10
258	8 weeks post winter:2 week winter vs. SPC -4 weeks post:2 week winter	63.9	39.7	24.2	3.688	10	10
259	8 weeks post winter:2 week winter vs. SPC -4 weeks post:4 week winter	63.9	44.25	19.65	3.688	10	10
260	8 weeks post winter:2 week winter vs. SPC -4 weeks post:8 week winter	63.9	52.8	11.1	3.688	10	10
261	8 weeks post winter:2 week winter vs. SPC -8 weeks post:2 week winter	63.9	52.45	11.45	3.688	10	10
262	8 weeks post winter:2 week winter vs. SPC -8 weeks post:4 week winter	63.9	52.8	11.1	3.688	10	10
263	8 weeks post winter:2 week winter vs. SPC -8 weeks post:8 week winter	63.9	65.55	-1.65	3.688	10	10
264	8 weeks post winter:4 week winter vs. 8 weeks post winter:8 week winter	66.6	74.65	-8.05	3.688	10	10
265	8 weeks post winter:4 week winter vs. SPC -first long day:2 week winter	66.6	39.6	27	3.688	10	10
266	8 weeks post winter:4 week winter vs. SPC -first long day:4 week winter	66.6	38.5	28.1	3.688	10	10
267	8 weeks post winter:4 week winter vs. SPC -first long day:8 week winter	66.6	44.25	22.35	3.688	10	10
268	8 weeks post winter:4 week winter vs. SPC -4 weeks post:2 week winter	66.6	39.7	26.9	3.688	10	10
269	8 weeks post winter:4 week winter vs. SPC -4 weeks post:4 week winter	66.6	44.25	22.35	3.688	10	10
270	8 weeks post winter:4 week winter vs. SPC -4 weeks post:8 week winter	66.6	52.8	13.8	3.688	10	10

2way ANOVA<br>Multiple comparisons							
271	8 weeks post winter:4 week winter vs. SPC -8 weeks post:2 week winter	66.6	52.45	14.15	3.688	10	10
272	8 weeks post winter:4 week winter vs. SPC -8 weeks post:4 week winter	66.6	52.8	13.8	3.688	10	10
273	8 weeks post winter:4 week winter vs. SPC -8 weeks post:8 week winter	66.6	65.55	1.05	3.688	10	10
274	8 weeks post winter:8 week winter vs. SPC -first long day:2 week winter	74.65	39.6	35.05	3.688	10	10
275	8 weeks post winter:8 week winter vs. SPC -first long day:4 week winter	74.65	38.5	36.15	3.688	10	10
276	8 weeks post winter:8 week winter vs. SPC -first long day:8 week winter	74.65	44.25	30.4	3.688	10	10
277	8 weeks post winter:8 week winter vs. SPC -4 weeks post:2 week winter	74.65	39.7	34.95	3.688	10	10
278	8 weeks post winter:8 week winter vs. SPC -4 weeks post:4 week winter	74.65	44.25	30.4	3.688	10	10
279	8 weeks post winter:8 week winter vs. SPC -4 weeks post:8 week winter	74.65	52.8	21.85	3.688	10	10
280	8 weeks post winter:8 week winter vs. SPC -8 weeks post:2 week winter	74.65	52.45	22.2	3.688	10	10
281	8 weeks post winter:8 week winter vs. SPC -8 weeks post:4 week winter	74.65	52.8	21.85	3.688	10	10
282	8 weeks post winter:8 week winter vs. SPC -8 weeks post:8 week winter	74.65	65.55	9.1	3.688	10	10
283	SPC -first long day:2 week winter vs. SPC -first long day:4 week winter	39.6	38.5	1.1	3.688	10	10
284	SPC -first long day:2 week winter vs. SPC -first long day:8 week winter	39.6	44.25	-4.65	3.688	10	10
285	SPC -first long day:2 week winter vs. SPC -4 weeks post:2 week winter	39.6	39.7	-0.1	3.688	10	10
286	SPC -first long day:2 week winter vs. SPC -4 weeks post:4 week winter	39.6	44.25	-4.65	3.688	10	10
287	SPC -first long day:2 week winter vs. SPC -4 weeks post:8 week winter	39.6	52.8	-13.2	3.688	10	10
288	SPC -first long day:2 week winter vs. SPC -8 weeks post:2 week winter	39.6	52.45	-12.85	3.688	10	10
289	SPC -first long day:2 week winter vs. SPC -8 weeks post:4 week winter	39.6	52.8	-13.2	3.688	10	10
290	SPC -first long day:2 week winter vs. SPC -8 weeks post:8 week winter	39.6	65.55	-25.95	3.688	10	10
291	SPC -first long day:4 week winter vs. SPC -first long day:8 week winter	38.5	44.25	-5.75	3.688	10	10
292	SPC -first long day:4 week winter vs. SPC -4 weeks post:2 week winter	38.5	39.7	-1.2	3.688	10	10
293	SPC -first long day:4 week winter vs. SPC -4 weeks post:4 week winter	38.5	44.25	-5.75	3.688	10	10
294	SPC -first long day:4 week winter vs. SPC -4 weeks post:8 week winter	38.5	52.8	-14.3	3.688	10	10
295	SPC -first long day:4 week winter vs. SPC -8 weeks post:2 week winter	38.5	52.45	-13.95	3.688	10	10
296	SPC -first long day:4 week winter vs. SPC -8 weeks post:4 week winter	38.5	52.8	-14.3	3.688	10	10
297	SPC -first long day:4 week winter vs. SPC -8 weeks post:8 week winter	38.5	65.55	-27.05	3.688	10	10
298	SPC -first long day:8 week winter vs. SPC -4 weeks post:2 week winter	44.25	39.7	4.55	3.688	10	10
299	SPC -first long day:8 week winter vs. SPC -4 weeks post:4 week winter	44.25	44.25	0	3.688	10	10
300	SPC -first long day:8 week winter vs. SPC -4 weeks post:8 week winter	44.25	52.8	-8.55	3.688	10	10

2way ANOVA<br>Multiple comparisons							
301	SPC -first long day:8 week winter vs. SPC -8 weeks post:2 week winter	44.25	52.45	-8.2	3.688	10	10
302	SPC -first long day:8 week winter vs. SPC -8 weeks post:4 week winter	44.25	52.8	-8.55	3.688	10	10
303	SPC -first long day:8 week winter vs. SPC -8 weeks post:8 week winter	44.25	65.55	-21.3	3.688	10	10
304	SPC -4 weeks post:2 week winter vs. SPC -4 weeks post:4 week winter	39.7	44.25	-4.55	3.688	10	10
305	SPC -4 weeks post:2 week winter vs. SPC -4 weeks post:8 week winter	39.7	52.8	-13.1	3.688	10	10
306	SPC -4 weeks post:2 week winter vs. SPC -8 weeks post:2 week winter	39.7	52.45	-12.75	3.688	10	10
307	SPC -4 weeks post:2 week winter vs. SPC -8 weeks post:4 week winter	39.7	52.8	-13.1	3.688	10	10
308	SPC -4 weeks post:2 week winter vs. SPC -8 weeks post:8 week winter	39.7	65.55	-25.85	3.688	10	10
309	SPC -4 weeks post:4 week winter vs. SPC -4 weeks post:8 week winter	44.25	52.8	-8.55	3.688	10	10
310	SPC -4 weeks post:4 week winter vs. SPC -8 weeks post:2 week winter	44.25	52.45	-8.2	3.688	10	10
311	SPC -4 weeks post:4 week winter vs. SPC -8 weeks post:4 week winter	44.25	52.8	-8.55	3.688	10	10
312	SPC -4 weeks post:4 week winter vs. SPC -8 weeks post:8 week winter	44.25	65.55	-21.3	3.688	10	10
313	SPC -4 weeks post:8 week winter vs. SPC -8 weeks post:2 week winter	52.8	52.45	0.35	3.688	10	10
314	SPC -4 weeks post:8 week winter vs. SPC -8 weeks post:4 week winter	52.8	52.8	0	3.688	10	10
315	SPC -4 weeks post:8 week winter vs. SPC -8 weeks post:8 week winter	52.8	65.55	-12.75	3.688	10	10
316	SPC -8 weeks post:2 week winter vs. SPC -8 weeks post:4 week winter	52.45	52.8	-0.35	3.688	10	10
317	SPC -8 weeks post:2 week winter vs. SPC -8 weeks post:8 week winter	52.45	65.55	-13.1	3.688	10	10
318	SPC -8 weeks post:4 week winter vs. SPC -8 weeks post:8 week winter	52.8	65.55	-12.75	3.688	10	10

1		
2		
3		
4		
5		
6		
7		
8		
9		
10		
11		
12		
13		
14		
15		
16		
17		
18		
19		
20		
21		
22		
23		
24		
25		
26		
27		
28		
29		
30		

31		
32		
33		
34		
35		
36		
37		
38		
39		
40		
41		
42		
43		
44		
45		
46		
47		
48		
49		
50		
51		
52		
53		
54		
55		
56		
57		
58		
59		
60		

61		
62		
63		
64		
65		
66		
67		
68		
69		
70		
71		
72		
73		
74		
75		
76		
77		
78		
79		
80		
81		
82		
83		
84		
85		
86		
87		
88		
89		
90		

91		
92		
93		
94		
95		
96		
97		
98		
99		
100		
101		
102		
103		
104		
105		
106		
107		
108		
109		
110		
111		
112		
113		
114		
115		
116		
117		
118		
119		
120		

121		
122		
123		
124		
125		
126		
127		
128		
129		
130		
131		
132		
133		
134		
135		
136		
137		
138		
139		
140		
141		
142		
143		
144		
145		
146		
147		
148		
149		
150		

**151**		
**152**		
**153**		
**154**		
**155**		
**156**		
**157**		
**158**		
**159**		
**160**		
**161**		
**162**		
**163**		
**164**	q	DF
**165**		
**166**	1.975	162
**167**	5.618	162
**168**	0.9395	162
**169**	6.788	162
**170**	6.327	162
**171**	10.05	162
**172**	11.08	162
**173**	14.17	162
**174**	0.7286	162
**175**	0.3068	162
**176**	2.512	162
**177**	0.767	162
**178**	2.512	162
**179**	5.79	162
**180**	5.656	162

181	5.79	162
182	10.68	162
183	3.643	162
184	1.035	162
185	4.813	162
186	4.352	162
187	8.072	162
188	9.108	162
189	12.19	162
190	1.246	162
191	1.668	162
192	0.5369	162
193	1.208	162
194	0.5369	162
195	3.816	162
196	3.681	162
197	3.816	162
198	8.705	162
199	4.678	162
200	1.17	162
201	0.7094	162
202	4.429	162
203	5.465	162
204	8.552	162
205	4.889	162
206	5.311	162
207	3.106	162
208	4.851	162
209	3.106	162
210	0.1726	162

**211**	0.03835	162
**212**	0.1726	162
**213**	5.062	162
**214**	5.848	162
**215**	5.388	162
**216**	9.108	162
**217**	10.14	162
**218**	13.23	162
**219**	0.2109	162
**220**	0.6327	162
**221**	1.572	162
**222**	0.1726	162
**223**	1.572	162
**224**	4.851	162
**225**	4.717	162
**226**	4.851	162
**227**	9.74	162
**228**	0.4602	162
**229**	3.26	162
**230**	4.295	162
**231**	7.382	162
**232**	6.059	162
**233**	6.481	162
**234**	4.276	162
**235**	6.021	162
**236**	4.276	162
**237**	0.997	162
**238**	1.131	162
**239**	0.997	162
**240**	3.892	162

**241**	3.72	162
**242**	4.755	162
**243**	7.842	162
**244**	5.599	162
**245**	6.021	162
**246**	3.816	162
**247**	5.56	162
**248**	3.816	162
**249**	0.5369	162
**250**	0.6711	162
**251**	0.5369	162
**252**	4.352	162
**253**	1.035	162
**254**	4.122	162
**255**	9.318	162
**256**	9.74	162
**257**	7.535	162
**258**	9.28	162
**259**	7.535	162
**260**	4.257	162
**261**	4.391	162
**262**	4.257	162
**263**	0.6327	162
**264**	3.087	162
**265**	10.35	162
**266**	10.78	162
**267**	8.571	162
**268**	10.32	162
**269**	8.571	162
**270**	5.292	162

**271**	5.426	162
**272**	5.292	162
**273**	0.4026	162
**274**	13.44	162
**275**	13.86	162
**276**	11.66	162
**277**	13.4	162
**278**	11.66	162
**279**	8.379	162
**280**	8.513	162
**281**	8.379	162
**282**	3.49	162
**283**	0.4218	162
**284**	1.783	162
**285**	0.03835	162
**286**	1.783	162
**287**	5.062	162
**288**	4.928	162
**289**	5.062	162
**290**	9.951	162
**291**	2.205	162
**292**	0.4602	162
**293**	2.205	162
**294**	5.484	162
**295**	5.349	162
**296**	5.484	162
**297**	10.37	162
**298**	1.745	162
**299**	0	162
**300**	3.279	162

**301**	3.144	162
**302**	3.279	162
**303**	8.168	162
**304**	1.745	162
**305**	5.024	162
**306**	4.889	162
**307**	5.024	162
**308**	9.913	162
**309**	3.279	162
**310**	3.144	162
**311**	3.279	162
**312**	8.168	162
**313**	0.1342	162
**314**	0	162
**315**	4.889	162
**316**	0.1342	162
**317**	5.024	162
**318**	4.889	162

Data analyzed: Weigth FW grouped format

Source of Variation	Degrees of Freedom	Sum of Squares	Mean square
Pretreatment	2	4194	2097
Treatment	5	15515	3103
Interaction	10	1160	116
Residual (error)	162	11016	68
Total	179	31885	

Does Pretreatment have the same effect at all values of Treatment?

Interaction accounts for 3.64% of the total variance.
F = 1.71. DFn=10 DFd=162
The P value = 0.0835
If there is no interaction overall, there is a 8.4% chance of randomly observing so much interaction in an experiment of this size. The interaction is considered not quite significant.

Does Pretreatment affect the result?

Pretreatment accounts for 13.15% of the total variance.
F = 30.84. DFn=2 DFd=162
The P value is < 0.0001
If Pretreatment has no effect overall, there is a less than 0.01% chance of randomly observing an effect this big (or bigger) in an experiment of this size. The effect is considered extremely significant.

Does Treatment affect the result?

Treatment accounts for 48.66% of the total variance.
F = 45.63. DFn=5 DFd=162
The P value is < 0.0001
If Treatment has no effect overall, there is a less than 0.01% chance of randomly observing an this big (or bigger) in an experiment of this size. The effect is considered extremely significant.

2way ANOVA
Tabular results

1	Table Analyzed	Weight SW grouped format				
2						
3	Two-way ANOVA	Ordinary				
4	Alpha	0.05				
5						
6	Source of Variation	% of total variation	P value	P value summary	Significant?	
7	Interaction	4.126	0.0034	**	Yes	
8	Treatment	13.24	<0.0001	****	Yes	
9	Pretreatment	16.33	<0.0001	****	Yes	
10						
11	ANOVA table	SS (Type III)	DF	MS	F (DFn, DFd)	P value
12	Interaction	2230	4	557.5	F (4, 260) = 4.045	P=0.0034
13	Treatment	7154	2	3577	F (2, 260) = 25.95	P<0.0001
14	Pretreatment	8823	2	4412	F (2, 260) = 32.01	P<0.0001
15	Residual	35833	260	137.8		
16						
17	Number of missing values	1				

2way ANOVA
Multiple comparisons

1	Within each row, compare columns (simple effects within rows)								
2									
3	Number of families	3							
4	Number of comparisons per family	3							
5	Alpha	0.05							
6									
7	Tukey's multiple comparisons test	Mean Diff.	95.00% CI of diff.	Significant?	Summary	Adjusted P Value			
8									
9	SW_1								
10	2 week winter vs. 4 week winter	-2.233	-9.378 to 4.912	No	ns	0.7418			
11	2 week winter vs. 8 week winter	-6.733	-13.88 to 0.4118	No	ns	0.0695			
12	4 week winter vs. 8 week winter	-4.5	-11.65 to 2.645	No	ns	0.3000			
13									
14	SW_2								
15	2 week winter vs. 4 week winter	-4.972	-12.18 to 2.234	No	ns	0.2363			
16	2 week winter vs. 8 week winter	-11.92	-19.13 to -4.716	Yes	***	0.0004			
17	4 week winter vs. 8 week winter	-6.95	-14.1 to 0.1951	No	ns	0.0586			
18									
19	SW_3								
20	2 week winter vs. 4 week winter	-12.38	-19.53 to -5.238	Yes	***	0.0002			
21	2 week winter vs. 8 week winter	-23.43	-30.58 to -16.29	Yes	****	<0.0001			
22	4 week winter vs. 8 week winter	-11.05	-18.2 to -3.905	Yes	***	0.0009			
23									
24									
25	Test details	Mean 1	Mean 2	Mean Diff.	SE of diff.	N1	N2	q	DF
26									
27	SW_1								
28	2 week winter vs. 4 week winter	67.17	69.4	-2.233	3.031	30	30	1.042	260
29	2 week winter vs. 8 week winter	67.17	73.9	-6.733	3.031	30	30	3.141	260
30	4 week winter vs. 8 week winter	69.4	73.9	-4.5	3.031	30	30	2.1	260

2way ANOVA<br>Multiple comparisons									
31									
32	SW_2								
33	2 week winter vs. 4 week winter	68.83	73.8	-4.972	3.057	29	30	2.3	260
34	2 week winter vs. 8 week winter	68.83	80.75	-11.92	3.057	29	30	5.515	260
35	4 week winter vs. 8 week winter	73.8	80.75	-6.95	3.031	30	30	3.243	260
36									
37	SW_3								
38	2 week winter vs. 4 week winter	70.63	83.02	-12.38	3.031	30	30	5.778	260
39	2 week winter vs. 8 week winter	70.63	94.07	-23.43	3.031	30	30	10.93	260
40	4 week winter vs. 8 week winter	83.02	94.07	-11.05	3.031	30	30	5.155	260

Data analyzed: Weight SW grouped format

Source of Variation	Degrees of Freedom	Sum of Squares	Mean square
Pretreatment	2	8823	4412
Treatment	2	7154	3577
Interaction	4	2230	557.5
Residual (error)	260	35833	137.8
Total	268		

Does Pretreatment have the same effect at all values of Treatment?

Interaction accounts for approximately 4.13% of the total variance.
F = 4.05. DFn=4 DFd=260
The P value = 0.0034
If there is no interaction overall, there is a 0.34% chance of randomly observing so much interaction in an experiment of this size. The interaction is considered very significant.

Since the interaction is statistically significant, the P values that follow for the row and column effects are difficult to interpret.

Does Pretreatment affect the result?

Pretreatment accounts for approximately 16.33% of the total variance.
F = 32.01. DFn=2 DFd=260
The P value is < 0.0001
If Pretreatment has no effect overall, there is a less than 0.01% chance of randomly observing an effect this big (or bigger) in an experiment of this size. The effect is considered extremely significant.

Does Treatment affect the result?

Treatment accounts for approximately 13.24% of the total variance.
F = 25.95. DFn=2 DFd=260
The P value is < 0.0001
If Treatment has no effect overall, there is a less than 0.01% chance of randomly observing an this big (or bigger) in an experiment of this size. The effect is considered extremely significant.

2way ANOVA
Tabular results

1	Table Analyzed	Condition factor FW grouped format		
2				
3	Two-way ANOVA	Ordinary		
4	Alpha	0.05		
5				
6	Source of Variation	% of total variation	P value	P value summary
7	Interaction	13.65	0.0009	***
8	Treatment	16.21	<0.0001	****
9	Pretreatment	0.6868	0.4507	ns
10				
11	ANOVA table	SS	DF	MS
12	Interaction	0.1266	10	0.01266
13	Treatment	0.1504	5	0.03008
14	Pretreatment	0.00637	2	0.003185
15	Residual	0.6442	162	0.003977
16				
17	Number of missing values	0		

1		
2		
3		
4		
5		
6	Significant?	
7	Yes	
8	Yes	
9	No	
10		
11	F (DFn, DFd)	P value
12	F (10, 162) = 3.184	P=0.0009
13	F (5, 162) = 7.563	P<0.0001
14	F (2, 162) = 0.801	P=0.4507
15		
16		
17		

2way ANOVA<br>Multiple comparisons				
1	Compare cell means regardless of rows and columns			
2				
3	Number of families	1		
4	Number of comparisons per family	153		
5	Alpha	0.05		
6				
7	Tukey's multiple comparisons test	Mean Diff.	95.00% CI of diff.	Significant?
8				
9	First long day:2 week winter vs. First long day:4 week winter	-0.0454	-0.1454 to 0.0546	No
10	First long day:2 week winter vs. First long day:8 week winter	-0.0208	-0.1208 to 0.0792	No
11	First long day:2 week winter vs. 4 weeks post winter:2 week winter	0.01739	-0.08262 to 0.1174	No
12	First long day:2 week winter vs. 4 weeks post winter:4 week winter	-0.06864	-0.1686 to 0.03137	No
13	First long day:2 week winter vs. 4 weeks post winter:8 week winter	-0.01685	-0.1169 to 0.08315	No
14	First long day:2 week winter vs. 8 weeks post winter:2 week winter	-0.07388	-0.1739 to 0.02612	No
15	First long day:2 week winter vs. 8 weeks post winter:4 week winter	-0.01017	-0.1102 to 0.08983	No
16	First long day:2 week winter vs. 8 weeks post winter:8 week winter	-0.04384	-0.1438 to 0.05616	No
17	First long day:2 week winter vs. SPC -first long day:2 week winter	-0.05722	-0.1572 to 0.04279	No
18	First long day:2 week winter vs. SPC -first long day:4 week winter	-0.01819	-0.1182 to 0.08181	No
19	First long day:2 week winter vs. SPC -first long day:8 week winter	-0.01287	-0.1129 to 0.08714	No
20	First long day:2 week winter vs. SPC -4 weeks post:2 week winter	-0.04907	-0.1491 to 0.05094	No
21	First long day:2 week winter vs. SPC -4 weeks post:4 week winter	-0.01287	-0.1129 to 0.08714	No
22	First long day:2 week winter vs. SPC -4 weeks post:8 week winter	-0.07404	-0.174 to 0.02596	No
23	First long day:2 week winter vs. SPC -8 weeks post:2 week winter	-0.1528	-0.2528 to -0.05278	Yes
24	First long day:2 week winter vs. SPC -8 weeks post:4 week winter	-0.07404	-0.174 to 0.02596	No
25	First long day:2 week winter vs. SPC -8 weeks post:8 week winter	-0.09168	-0.1917 to 0.008327	No
26	First long day:4 week winter vs. First long day:8 week winter	0.0246	-0.0754 to 0.1246	No
27	First long day:4 week winter vs. 4 weeks post winter:2 week winter	0.06279	-0.03721 to 0.1628	No
28	First long day:4 week winter vs. 4 weeks post winter:4 week winter	-0.02323	-0.1232 to 0.07677	No
29	First long day:4 week winter vs. 4 weeks post winter:8 week winter	0.02855	-0.07145 to 0.1286	No
30	First long day:4 week winter vs. 8 weeks post winter:2 week winter	-0.02848	-0.1285 to 0.07153	No
31	First long day:4 week winter vs. 8 weeks post winter:4 week winter	0.03523	-0.06477 to 0.1352	No
32	First long day:4 week winter vs. 8 weeks post winter:8 week winter	0.001561	-0.09844 to 0.1016	No
33	First long day:4 week winter vs. SPC -first long day:2 week winter	-0.01182	-0.1118 to 0.08819	No
34	First long day:4 week winter vs. SPC -first long day:4 week winter	0.02721	-0.07279 to 0.1272	No
35	First long day:4 week winter vs. SPC -first long day:8 week winter	0.03253	-0.06747 to 0.1325	No
36	First long day:4 week winter vs. SPC -4 weeks post:2 week winter	-0.003666	-0.1037 to 0.09634	No
37	First long day:4 week winter vs. SPC -4 weeks post:4 week winter	0.03253	-0.06747 to 0.1325	No
38	First long day:4 week winter vs. SPC -4 weeks post:8 week winter	-0.02864	-0.1286 to 0.07137	No
39	First long day:4 week winter vs. SPC -8 weeks post:2 week winter	-0.1074	-0.2074 to -0.007382	Yes
40	First long day:4 week winter vs. SPC -8 weeks post:4 week winter	-0.02864	-0.1286 to 0.07137	No
41	First long day:4 week winter vs. SPC -8 weeks post:8 week winter	-0.04627	-0.1463 to 0.05373	No
42	First long day:8 week winter vs. 4 weeks post winter:2 week winter	0.03819	-0.06182 to 0.1382	No
43	First long day:8 week winter vs. 4 weeks post winter:4 week winter	-0.04784	-0.1478 to 0.05217	No
44	First long day:8 week winter vs. 4 weeks post winter:8 week winter	0.00395	-0.09605 to 0.104	No
45	First long day:8 week winter vs. 8 weeks post winter:2 week winter	-0.05308	-0.1531 to 0.04692	No

2way ANOVA<br>Multiple comparisons				
46	First long day:8 week winter vs. 8 weeks post winter:4 week winter	0.01063	-0.08938 to 0.1106	No
47	First long day:8 week winter vs. 8 weeks post winter:8 week winter	-0.02304	-0.123 to 0.07696	No
48	First long day:8 week winter vs. SPC -first long day:2 week winter	-0.03642	-0.1364 to 0.06359	No
49	First long day:8 week winter vs. SPC -first long day:4 week winter	0.00261	-0.09739 to 0.1026	No
50	First long day:8 week winter vs. SPC -first long day:8 week winter	0.007931	-0.09207 to 0.1079	No
51	First long day:8 week winter vs. SPC -4 weeks post:2 week winter	-0.02827	-0.1283 to 0.07174	No
52	First long day:8 week winter vs. SPC -4 weeks post:4 week winter	0.007931	-0.09207 to 0.1079	No
53	First long day:8 week winter vs. SPC -4 weeks post:8 week winter	-0.05324	-0.1532 to 0.04676	No
54	First long day:8 week winter vs. SPC -8 weeks post:2 week winter	-0.132	-0.232 to -0.03198	Yes
55	First long day:8 week winter vs. SPC -8 weeks post:4 week winter	-0.05324	-0.1532 to 0.04676	No
56	First long day:8 week winter vs. SPC -8 weeks post:8 week winter	-0.07088	-0.1709 to 0.02913	No
57	4 weeks post winter:2 week winter vs. 4 weeks post winter:4 week winter	-0.08602	-0.186 to 0.01398	No
58	4 weeks post winter:2 week winter vs. 4 weeks post winter:8 week winter	-0.03424	-0.1342 to 0.06577	No
59	4 weeks post winter:2 week winter vs. 8 weeks post winter:2 week winter	-0.09127	-0.1913 to 0.008735	No
60	4 weeks post winter:2 week winter vs. 8 weeks post winter:4 week winter	-0.02756	-0.1276 to 0.07245	No
61	4 weeks post winter:2 week winter vs. 8 weeks post winter:8 week winter	-0.06123	-0.1612 to 0.03878	No
62	4 weeks post winter:2 week winter vs. SPC -first long day:2 week winter	-0.07461	-0.1746 to 0.0254	No
63	4 weeks post winter:2 week winter vs. SPC -first long day:4 week winter	-0.03558	-0.1356 to 0.06443	No
64	4 weeks post winter:2 week winter vs. SPC -first long day:8 week winter	-0.03026	-0.1303 to 0.06975	No
65	4 weeks post winter:2 week winter vs. SPC -4 weeks post:2 week winter	-0.06646	-0.1665 to 0.03355	No
66	4 weeks post winter:2 week winter vs. SPC -4 weeks post:4 week winter	-0.03026	-0.1303 to 0.06975	No
67	4 weeks post winter:2 week winter vs. SPC -4 weeks post:8 week winter	-0.09143	-0.1914 to 0.008575	No
68	4 weeks post winter:2 week winter vs. SPC -8 weeks post:2 week winter	-0.1702	-0.2702 to -0.07017	Yes
69	4 weeks post winter:2 week winter vs. SPC -8 weeks post:4 week winter	-0.09143	-0.1914 to 0.008575	No
70	4 weeks post winter:2 week winter vs. SPC -8 weeks post:8 week winter	-0.1091	-0.2091 to -0.00906	Yes
71	4 weeks post winter:4 week winter vs. 4 weeks post winter:8 week winter	0.05179	-0.04822 to 0.1518	No
72	4 weeks post winter:4 week winter vs. 8 weeks post winter:2 week winter	-0.005246	-0.1053 to 0.09476	No
73	4 weeks post winter:4 week winter vs. 8 weeks post winter:4 week winter	0.05847	-0.04154 to 0.1585	No
74	4 weeks post winter:4 week winter vs. 8 weeks post winter:8 week winter	0.02479	-0.07521 to 0.1248	No
75	4 weeks post winter:4 week winter vs. SPC -first long day:2 week winter	0.01142	-0.08859 to 0.1114	No
76	4 weeks post winter:4 week winter vs. SPC -first long day:4 week winter	0.05045	-0.04956 to 0.1505	No
77	4 weeks post winter:4 week winter vs. SPC -first long day:8 week winter	0.05577	-0.04424 to 0.1558	No
78	4 weeks post winter:4 week winter vs. SPC -4 weeks post:2 week winter	0.01957	-0.08044 to 0.1196	No
79	4 weeks post winter:4 week winter vs. SPC -4 weeks post:4 week winter	0.05577	-0.04424 to 0.1558	No
80	4 weeks post winter:4 week winter vs. SPC -4 weeks post:8 week winter	-0.005406	-0.1054 to 0.0946	No
81	4 weeks post winter:4 week winter vs. SPC -8 weeks post:2 week winter	-0.08415	-0.1842 to 0.01585	No
82	4 weeks post winter:4 week winter vs. SPC -8 weeks post:4 week winter	-0.005406	-0.1054 to 0.0946	No
83	4 weeks post winter:4 week winter vs. SPC -8 weeks post:8 week winter	-0.02304	-0.123 to 0.07696	No
84	4 weeks post winter:8 week winter vs. 8 weeks post winter:2 week winter	-0.05703	-0.157 to 0.04297	No
85	4 weeks post winter:8 week winter vs. 8 weeks post winter:4 week winter	0.006679	-0.09333 to 0.1067	No
86	4 weeks post winter:8 week winter vs. 8 weeks post winter:8 week winter	-0.02699	-0.127 to 0.07301	No
87	4 weeks post winter:8 week winter vs. SPC -first long day:2 week winter	-0.04037	-0.1404 to 0.05964	No
88	4 weeks post winter:8 week winter vs. SPC -first long day:4 week winter	-0.00134	-0.1013 to 0.09866	No
89	4 weeks post winter:8 week winter vs. SPC -first long day:8 week winter	0.003981	-0.09602 to 0.104	No
90	4 weeks post winter:8 week winter vs. SPC -4 weeks post:2 week winter	-0.03222	-0.1322 to 0.06779	No

2way ANOVA<br>Multiple comparisons				
91	4 weeks post winter:8 week winter vs. SPC -4 weeks post:4 week winter	0.003981	-0.09602 to 0.104	No
92	4 weeks post winter:8 week winter vs. SPC -4 weeks post:8 week winter	-0.05719	-0.1572 to 0.04281	No
93	4 weeks post winter:8 week winter vs. SPC -8 weeks post:2 week winter	-0.1359	-0.2359 to -0.03593	Yes
94	4 weeks post winter:8 week winter vs. SPC -8 weeks post:4 week winter	-0.05719	-0.1572 to 0.04281	No
95	4 weeks post winter:8 week winter vs. SPC -8 weeks post:8 week winter	-0.07483	-0.1748 to 0.02518	No
96	8 weeks post winter:2 week winter vs. 8 weeks post winter:4 week winter	0.06371	-0.03629 to 0.1637	No
97	8 weeks post winter:2 week winter vs. 8 weeks post winter:8 week winter	0.03004	-0.06996 to 0.13	No
98	8 weeks post winter:2 week winter vs. SPC -first long day:2 week winter	0.01666	-0.08334 to 0.1167	No
99	8 weeks post winter:2 week winter vs. SPC -first long day:4 week winter	0.05569	-0.04431 to 0.1557	No
100	8 weeks post winter:2 week winter vs. SPC -first long day:8 week winter	0.06101	-0.03899 to 0.161	No
101	8 weeks post winter:2 week winter vs. SPC -4 weeks post:2 week winter	0.02481	-0.07519 to 0.1248	No
102	8 weeks post winter:2 week winter vs. SPC -4 weeks post:4 week winter	0.06101	-0.03899 to 0.161	No
103	8 weeks post winter:2 week winter vs. SPC -4 weeks post:8 week winter	-0.00016	-0.1002 to 0.09984	No
104	8 weeks post winter:2 week winter vs. SPC -8 weeks post:2 week winter	-0.07891	-0.1789 to 0.0211	No
105	8 weeks post winter:2 week winter vs. SPC -8 weeks post:4 week winter	-0.00016	-0.1002 to 0.09984	No
106	8 weeks post winter:2 week winter vs. SPC -8 weeks post:8 week winter	-0.0178	-0.1178 to 0.08221	No
107	8 weeks post winter:4 week winter vs. 8 weeks post winter:8 week winter	-0.03367	-0.1337 to 0.06633	No
108	8 weeks post winter:4 week winter vs. SPC -first long day:2 week winter	-0.04705	-0.1471 to 0.05296	No
109	8 weeks post winter:4 week winter vs. SPC -first long day:4 week winter	-0.008019	-0.108 to 0.09199	No
110	8 weeks post winter:4 week winter vs. SPC -first long day:8 week winter	-0.002698	-0.1027 to 0.09731	No
111	8 weeks post winter:4 week winter vs. SPC -4 weeks post:2 week winter	-0.0389	-0.1389 to 0.06111	No
112	8 weeks post winter:4 week winter vs. SPC -4 weeks post:4 week winter	-0.002698	-0.1027 to 0.09731	No
113	8 weeks post winter:4 week winter vs. SPC -4 weeks post:8 week winter	-0.06387	-0.1639 to 0.03613	No
114	8 weeks post winter:4 week winter vs. SPC -8 weeks post:2 week winter	-0.1426	-0.2426 to -0.04261	Yes
115	8 weeks post winter:4 week winter vs. SPC -8 weeks post:4 week winter	-0.06387	-0.1639 to 0.03613	No
116	8 weeks post winter:4 week winter vs. SPC -8 weeks post:8 week winter	-0.08151	-0.1815 to 0.0185	No
117	8 weeks post winter:8 week winter vs. SPC -first long day:2 week winter	-0.01338	-0.1134 to 0.08663	No
118	8 weeks post winter:8 week winter vs. SPC -first long day:4 week winter	0.02565	-0.07435 to 0.1257	No
119	8 weeks post winter:8 week winter vs. SPC -first long day:8 week winter	0.03097	-0.06903 to 0.131	No
120	8 weeks post winter:8 week winter vs. SPC -4 weeks post:2 week winter	-0.005226	-0.1052 to 0.09478	No
121	8 weeks post winter:8 week winter vs. SPC -4 weeks post:4 week winter	0.03097	-0.06903 to 0.131	No
122	8 weeks post winter:8 week winter vs. SPC -4 weeks post:8 week winter	-0.0302	-0.1302 to 0.0698	No
123	8 weeks post winter:8 week winter vs. SPC -8 weeks post:2 week winter	-0.1089	-0.209 to -0.008942	Yes
124	8 weeks post winter:8 week winter vs. SPC -8 weeks post:4 week winter	-0.0302	-0.1302 to 0.0698	No
125	8 weeks post winter:8 week winter vs. SPC -8 weeks post:8 week winter	-0.04784	-0.1478 to 0.05217	No
126	SPC -first long day:2 week winter vs. SPC -first long day:4 week winter	0.03903	-0.06098 to 0.139	No
127	SPC -first long day:2 week winter vs. SPC -first long day:8 week winter	0.04435	-0.05566 to 0.1444	No
128	SPC -first long day:2 week winter vs. SPC -4 weeks post:2 week winter	0.00815	-0.09185 to 0.1082	No
129	SPC -first long day:2 week winter vs. SPC -4 weeks post:4 week winter	0.04435	-0.05566 to 0.1444	No
130	SPC -first long day:2 week winter vs. SPC -4 weeks post:8 week winter	-0.01682	-0.1168 to 0.08318	No
131	SPC -first long day:2 week winter vs. SPC -8 weeks post:2 week winter	-0.09557	-0.1956 to 0.004434	No
132	SPC -first long day:2 week winter vs. SPC -8 weeks post:4 week winter	-0.01682	-0.1168 to 0.08318	No
133	SPC -first long day:2 week winter vs. SPC -8 weeks post:8 week winter	-0.03446	-0.1345 to 0.06555	No
134	SPC -first long day:4 week winter vs. SPC -first long day:8 week winter	0.005321	-0.09468 to 0.1053	No
135	SPC -first long day:4 week winter vs. SPC -4 weeks post:2 week winter	-0.03088	-0.1309 to 0.06913	No

2way ANOVA<br>Multiple comparisons				
136	SPC -first long day:4 week winter vs. SPC -4 weeks post:4 week winter	0.005321	-0.09468 to 0.1053	No
137	SPC -first long day:4 week winter vs. SPC -4 weeks post:8 week winter	-0.05585	-0.1559 to 0.04415	No
138	SPC -first long day:4 week winter vs. SPC -8 weeks post:2 week winter	-0.1346	-0.2346 to -0.03459	Yes
139	SPC -first long day:4 week winter vs. SPC -8 weeks post:4 week winter	-0.05585	-0.1559 to 0.04415	No
140	SPC -first long day:4 week winter vs. SPC -8 weeks post:8 week winter	-0.07349	-0.1735 to 0.02652	No
141	SPC -first long day:8 week winter vs. SPC -4 weeks post:2 week winter	-0.0362	-0.1362 to 0.06381	No
142	SPC -first long day:8 week winter vs. SPC -4 weeks post:4 week winter	0	-0.1 to 0.1	No
143	SPC -first long day:8 week winter vs. SPC -4 weeks post:8 week winter	-0.06117	-0.1612 to 0.03883	No
144	SPC -first long day:8 week winter vs. SPC -8 weeks post:2 week winter	-0.1399	-0.2399 to -0.03992	Yes
145	SPC -first long day:8 week winter vs. SPC -8 weeks post:4 week winter	-0.06117	-0.1612 to 0.03883	No
146	SPC -first long day:8 week winter vs. SPC -8 weeks post:8 week winter	-0.07881	-0.1788 to 0.0212	No
147	SPC -4 weeks post:2 week winter vs. SPC -4 weeks post:4 week winter	0.0362	-0.06381 to 0.1362	No
148	SPC -4 weeks post:2 week winter vs. SPC -4 weeks post:8 week winter	-0.02497	-0.125 to 0.07503	No
149	SPC -4 weeks post:2 week winter vs. SPC -8 weeks post:2 week winter	-0.1037	-0.2037 to -0.003716	Yes
150	SPC -4 weeks post:2 week winter vs. SPC -8 weeks post:4 week winter	-0.02497	-0.125 to 0.07503	No
151	SPC -4 weeks post:2 week winter vs. SPC -8 weeks post:8 week winter	-0.04261	-0.1426 to 0.0574	No
152	SPC -4 weeks post:4 week winter vs. SPC -4 weeks post:8 week winter	-0.06117	-0.1612 to 0.03883	No
153	SPC -4 weeks post:4 week winter vs. SPC -8 weeks post:2 week winter	-0.1399	-0.2399 to -0.03992	Yes
154	SPC -4 weeks post:4 week winter vs. SPC -8 weeks post:4 week winter	-0.06117	-0.1612 to 0.03883	No
155	SPC -4 weeks post:4 week winter vs. SPC -8 weeks post:8 week winter	-0.07881	-0.1788 to 0.0212	No
156	SPC -4 weeks post:8 week winter vs. SPC -8 weeks post:2 week winter	-0.07875	-0.1788 to 0.02126	No
157	SPC -4 weeks post:8 week winter vs. SPC -8 weeks post:4 week winter	0	-0.1 to 0.1	No
158	SPC -4 weeks post:8 week winter vs. SPC -8 weeks post:8 week winter	-0.01764	-0.1176 to 0.08237	No
159	SPC -8 weeks post:2 week winter vs. SPC -8 weeks post:4 week winter	0.07875	-0.02126 to 0.1788	No
160	SPC -8 weeks post:2 week winter vs. SPC -8 weeks post:8 week winter	0.06111	-0.03889 to 0.1611	No
161	SPC -8 weeks post:4 week winter vs. SPC -8 weeks post:8 week winter	-0.01764	-0.1176 to 0.08237	No
162				
163				
164	Test details	Mean 1	Mean 2	Mean Diff.
165				
166	First long day:2 week winter vs. First long day:4 week winter	1.174	1.219	-0.0454
167	First long day:2 week winter vs. First long day:8 week winter	1.174	1.194	-0.0208
168	First long day:2 week winter vs. 4 weeks post winter:2 week winter	1.174	1.156	0.01739
169	First long day:2 week winter vs. 4 weeks post winter:4 week winter	1.174	1.242	-0.06864
170	First long day:2 week winter vs. 4 weeks post winter:8 week winter	1.174	1.191	-0.01685
171	First long day:2 week winter vs. 8 weeks post winter:2 week winter	1.174	1.248	-0.07388
172	First long day:2 week winter vs. 8 weeks post winter:4 week winter	1.174	1.184	-0.01017
173	First long day:2 week winter vs. 8 weeks post winter:8 week winter	1.174	1.217	-0.04384
174	First long day:2 week winter vs. SPC -first long day:2 week winter	1.174	1.231	-0.05722
175	First long day:2 week winter vs. SPC -first long day:4 week winter	1.174	1.192	-0.01819
176	First long day:2 week winter vs. SPC -first long day:8 week winter	1.174	1.187	-0.01287
177	First long day:2 week winter vs. SPC -4 weeks post:2 week winter	1.174	1.223	-0.04907
178	First long day:2 week winter vs. SPC -4 weeks post:4 week winter	1.174	1.187	-0.01287
179	First long day:2 week winter vs. SPC -4 weeks post:8 week winter	1.174	1.248	-0.07404
180	First long day:2 week winter vs. SPC -8 weeks post:2 week winter	1.174	1.326	-0.1528

2way ANOVA<br>Multiple comparisons				
181	First long day:2 week winter vs. SPC -8 weeks post:4 week winter	1.174	1.248	-0.07404
182	First long day:2 week winter vs. SPC -8 weeks post:8 week winter	1.174	1.265	-0.09168
183	First long day:4 week winter vs. First long day:8 week winter	1.219	1.194	0.0246
184	First long day:4 week winter vs. 4 weeks post winter:2 week winter	1.219	1.156	0.06279
185	First long day:4 week winter vs. 4 weeks post winter:4 week winter	1.219	1.242	-0.02323
186	First long day:4 week winter vs. 4 weeks post winter:8 week winter	1.219	1.191	0.02855
187	First long day:4 week winter vs. 8 weeks post winter:2 week winter	1.219	1.248	-0.02848
188	First long day:4 week winter vs. 8 weeks post winter:4 week winter	1.219	1.184	0.03523
189	First long day:4 week winter vs. 8 weeks post winter:8 week winter	1.219	1.217	0.001561
190	First long day:4 week winter vs. SPC -first long day:2 week winter	1.219	1.231	-0.01182
191	First long day:4 week winter vs. SPC -first long day:4 week winter	1.219	1.192	0.02721
192	First long day:4 week winter vs. SPC -first long day:8 week winter	1.219	1.187	0.03253
193	First long day:4 week winter vs. SPC -4 weeks post:2 week winter	1.219	1.223	-0.003666
194	First long day:4 week winter vs. SPC -4 weeks post:4 week winter	1.219	1.187	0.03253
195	First long day:4 week winter vs. SPC -4 weeks post:8 week winter	1.219	1.248	-0.02864
196	First long day:4 week winter vs. SPC -8 weeks post:2 week winter	1.219	1.326	-0.1074
197	First long day:4 week winter vs. SPC -8 weeks post:4 week winter	1.219	1.248	-0.02864
198	First long day:4 week winter vs. SPC -8 weeks post:8 week winter	1.219	1.265	-0.04627
199	First long day:8 week winter vs. 4 weeks post winter:2 week winter	1.194	1.156	0.03819
200	First long day:8 week winter vs. 4 weeks post winter:4 week winter	1.194	1.242	-0.04784
201	First long day:8 week winter vs. 4 weeks post winter:8 week winter	1.194	1.191	0.00395
202	First long day:8 week winter vs. 8 weeks post winter:2 week winter	1.194	1.248	-0.05308
203	First long day:8 week winter vs. 8 weeks post winter:4 week winter	1.194	1.184	0.01063
204	First long day:8 week winter vs. 8 weeks post winter:8 week winter	1.194	1.217	-0.02304
205	First long day:8 week winter vs. SPC -first long day:2 week winter	1.194	1.231	-0.03642
206	First long day:8 week winter vs. SPC -first long day:4 week winter	1.194	1.192	0.00261
207	First long day:8 week winter vs. SPC -first long day:8 week winter	1.194	1.187	0.007931
208	First long day:8 week winter vs. SPC -4 weeks post:2 week winter	1.194	1.223	-0.02827
209	First long day:8 week winter vs. SPC -4 weeks post:4 week winter	1.194	1.187	0.007931
210	First long day:8 week winter vs. SPC -4 weeks post:8 week winter	1.194	1.248	-0.05324
211	First long day:8 week winter vs. SPC -8 weeks post:2 week winter	1.194	1.326	-0.132
212	First long day:8 week winter vs. SPC -8 weeks post:4 week winter	1.194	1.248	-0.05324
213	First long day:8 week winter vs. SPC -8 weeks post:8 week winter	1.194	1.265	-0.07088
214	4 weeks post winter:2 week winter vs. 4 weeks post winter:4 week winter	1.156	1.242	-0.08602
215	4 weeks post winter:2 week winter vs. 4 weeks post winter:8 week winter	1.156	1.191	-0.03424
216	4 weeks post winter:2 week winter vs. 8 weeks post winter:2 week winter	1.156	1.248	-0.09127
217	4 weeks post winter:2 week winter vs. 8 weeks post winter:4 week winter	1.156	1.184	-0.02756
218	4 weeks post winter:2 week winter vs. 8 weeks post winter:8 week winter	1.156	1.217	-0.06123
219	4 weeks post winter:2 week winter vs. SPC -first long day:2 week winter	1.156	1.231	-0.07461
220	4 weeks post winter:2 week winter vs. SPC -first long day:4 week winter	1.156	1.192	-0.03558
221	4 weeks post winter:2 week winter vs. SPC -first long day:8 week winter	1.156	1.187	-0.03026
222	4 weeks post winter:2 week winter vs. SPC -4 weeks post:2 week winter	1.156	1.223	-0.06646
223	4 weeks post winter:2 week winter vs. SPC -4 weeks post:4 week winter	1.156	1.187	-0.03026
224	4 weeks post winter:2 week winter vs. SPC -4 weeks post:8 week winter	1.156	1.248	-0.09143
225	4 weeks post winter:2 week winter vs. SPC -8 weeks post:2 week winter	1.156	1.326	-0.1702

2way ANOVA<br>Multiple comparisons				
226	4 weeks post winter:2 week winter vs. SPC -8 weeks post:4 week winter	1.156	1.248	-0.09143
227	4 weeks post winter:2 week winter vs. SPC -8 weeks post:8 week winter	1.156	1.265	-0.1091
228	4 weeks post winter:4 week winter vs. 4 weeks post winter:8 week winter	1.242	1.191	0.05179
229	4 weeks post winter:4 week winter vs. 8 weeks post winter:2 week winter	1.242	1.248	-0.005246
230	4 weeks post winter:4 week winter vs. 8 weeks post winter:4 week winter	1.242	1.184	0.05847
231	4 weeks post winter:4 week winter vs. 8 weeks post winter:8 week winter	1.242	1.217	0.02479
232	4 weeks post winter:4 week winter vs. SPC -first long day:2 week winter	1.242	1.231	0.01142
233	4 weeks post winter:4 week winter vs. SPC -first long day:4 week winter	1.242	1.192	0.05045
234	4 weeks post winter:4 week winter vs. SPC -first long day:8 week winter	1.242	1.187	0.05577
235	4 weeks post winter:4 week winter vs. SPC -4 weeks post:2 week winter	1.242	1.223	0.01957
236	4 weeks post winter:4 week winter vs. SPC -4 weeks post:4 week winter	1.242	1.187	0.05577
237	4 weeks post winter:4 week winter vs. SPC -4 weeks post:8 week winter	1.242	1.248	-0.005406
238	4 weeks post winter:4 week winter vs. SPC -8 weeks post:2 week winter	1.242	1.326	-0.08415
239	4 weeks post winter:4 week winter vs. SPC -8 weeks post:4 week winter	1.242	1.248	-0.005406
240	4 weeks post winter:4 week winter vs. SPC -8 weeks post:8 week winter	1.242	1.265	-0.02304
241	4 weeks post winter:8 week winter vs. 8 weeks post winter:2 week winter	1.191	1.248	-0.05703
242	4 weeks post winter:8 week winter vs. 8 weeks post winter:4 week winter	1.191	1.184	0.006679
243	4 weeks post winter:8 week winter vs. 8 weeks post winter:8 week winter	1.191	1.217	-0.02699
244	4 weeks post winter:8 week winter vs. SPC -first long day:2 week winter	1.191	1.231	-0.04037
245	4 weeks post winter:8 week winter vs. SPC -first long day:4 week winter	1.191	1.192	-0.00134
246	4 weeks post winter:8 week winter vs. SPC -first long day:8 week winter	1.191	1.187	0.003981
247	4 weeks post winter:8 week winter vs. SPC -4 weeks post:2 week winter	1.191	1.223	-0.03222
248	4 weeks post winter:8 week winter vs. SPC -4 weeks post:4 week winter	1.191	1.187	0.003981
249	4 weeks post winter:8 week winter vs. SPC -4 weeks post:8 week winter	1.191	1.248	-0.05719
250	4 weeks post winter:8 week winter vs. SPC -8 weeks post:2 week winter	1.191	1.326	-0.1359
251	4 weeks post winter:8 week winter vs. SPC -8 weeks post:4 week winter	1.191	1.248	-0.05719
252	4 weeks post winter:8 week winter vs. SPC -8 weeks post:8 week winter	1.191	1.265	-0.07483
253	8 weeks post winter:2 week winter vs. 8 weeks post winter:4 week winter	1.248	1.184	0.06371
254	8 weeks post winter:2 week winter vs. 8 weeks post winter:8 week winter	1.248	1.217	0.03004
255	8 weeks post winter:2 week winter vs. SPC -first long day:2 week winter	1.248	1.231	0.01666
256	8 weeks post winter:2 week winter vs. SPC -first long day:4 week winter	1.248	1.192	0.05569
257	8 weeks post winter:2 week winter vs. SPC -first long day:8 week winter	1.248	1.187	0.06101
258	8 weeks post winter:2 week winter vs. SPC -4 weeks post:2 week winter	1.248	1.223	0.02481
259	8 weeks post winter:2 week winter vs. SPC -4 weeks post:4 week winter	1.248	1.187	0.06101
260	8 weeks post winter:2 week winter vs. SPC -4 weeks post:8 week winter	1.248	1.248	-0.00016
261	8 weeks post winter:2 week winter vs. SPC -8 weeks post:2 week winter	1.248	1.326	-0.07891
262	8 weeks post winter:2 week winter vs. SPC -8 weeks post:4 week winter	1.248	1.248	-0.00016
263	8 weeks post winter:2 week winter vs. SPC -8 weeks post:8 week winter	1.248	1.265	-0.0178
264	8 weeks post winter:4 week winter vs. 8 weeks post winter:8 week winter	1.184	1.217	-0.03367
265	8 weeks post winter:4 week winter vs. SPC -first long day:2 week winter	1.184	1.231	-0.04705
266	8 weeks post winter:4 week winter vs. SPC -first long day:4 week winter	1.184	1.192	-0.008019
267	8 weeks post winter:4 week winter vs. SPC -first long day:8 week winter	1.184	1.187	-0.002698
268	8 weeks post winter:4 week winter vs. SPC -4 weeks post:2 week winter	1.184	1.223	-0.0389
269	8 weeks post winter:4 week winter vs. SPC -4 weeks post:4 week winter	1.184	1.187	-0.002698
270	8 weeks post winter:4 week winter vs. SPC -4 weeks post:8 week winter	1.184	1.248	-0.06387

2way ANOVA<br>Multiple comparisons				
271	8 weeks post winter:4 week winter vs. SPC -8 weeks post:2 week winter	1.184	1.326	-0.1426
272	8 weeks post winter:4 week winter vs. SPC -8 weeks post:4 week winter	1.184	1.248	-0.06387
273	8 weeks post winter:4 week winter vs. SPC -8 weeks post:8 week winter	1.184	1.265	-0.08151
274	8 weeks post winter:8 week winter vs. SPC -first long day:2 week winter	1.217	1.231	-0.01338
275	8 weeks post winter:8 week winter vs. SPC -first long day:4 week winter	1.217	1.192	0.02565
276	8 weeks post winter:8 week winter vs. SPC -first long day:8 week winter	1.217	1.187	0.03097
277	8 weeks post winter:8 week winter vs. SPC -4 weeks post:2 week winter	1.217	1.223	-0.005226
278	8 weeks post winter:8 week winter vs. SPC -4 weeks post:4 week winter	1.217	1.187	0.03097
279	8 weeks post winter:8 week winter vs. SPC -4 weeks post:8 week winter	1.217	1.248	-0.0302
280	8 weeks post winter:8 week winter vs. SPC -8 weeks post:2 week winter	1.217	1.326	-0.1089
281	8 weeks post winter:8 week winter vs. SPC -8 weeks post:4 week winter	1.217	1.248	-0.0302
282	8 weeks post winter:8 week winter vs. SPC -8 weeks post:8 week winter	1.217	1.265	-0.04784
283	SPC -first long day:2 week winter vs. SPC -first long day:4 week winter	1.231	1.192	0.03903
284	SPC -first long day:2 week winter vs. SPC -first long day:8 week winter	1.231	1.187	0.04435
285	SPC -first long day:2 week winter vs. SPC -4 weeks post:2 week winter	1.231	1.223	0.00815
286	SPC -first long day:2 week winter vs. SPC -4 weeks post:4 week winter	1.231	1.187	0.04435
287	SPC -first long day:2 week winter vs. SPC -4 weeks post:8 week winter	1.231	1.248	-0.01682
288	SPC -first long day:2 week winter vs. SPC -8 weeks post:2 week winter	1.231	1.326	-0.09557
289	SPC -first long day:2 week winter vs. SPC -8 weeks post:4 week winter	1.231	1.248	-0.01682
290	SPC -first long day:2 week winter vs. SPC -8 weeks post:8 week winter	1.231	1.265	-0.03446
291	SPC -first long day:4 week winter vs. SPC -first long day:8 week winter	1.192	1.187	0.005321
292	SPC -first long day:4 week winter vs. SPC -4 weeks post:2 week winter	1.192	1.223	-0.03088
293	SPC -first long day:4 week winter vs. SPC -4 weeks post:4 week winter	1.192	1.187	0.005321
294	SPC -first long day:4 week winter vs. SPC -4 weeks post:8 week winter	1.192	1.248	-0.05585
295	SPC -first long day:4 week winter vs. SPC -8 weeks post:2 week winter	1.192	1.326	-0.1346
296	SPC -first long day:4 week winter vs. SPC -8 weeks post:4 week winter	1.192	1.248	-0.05585
297	SPC -first long day:4 week winter vs. SPC -8 weeks post:8 week winter	1.192	1.265	-0.07349
298	SPC -first long day:8 week winter vs. SPC -4 weeks post:2 week winter	1.187	1.223	-0.0362
299	SPC -first long day:8 week winter vs. SPC -4 weeks post:4 week winter	1.187	1.187	0
300	SPC -first long day:8 week winter vs. SPC -4 weeks post:8 week winter	1.187	1.248	-0.06117
301	SPC -first long day:8 week winter vs. SPC -8 weeks post:2 week winter	1.187	1.326	-0.1399
302	SPC -first long day:8 week winter vs. SPC -8 weeks post:4 week winter	1.187	1.248	-0.06117
303	SPC -first long day:8 week winter vs. SPC -8 weeks post:8 week winter	1.187	1.265	-0.07881
304	SPC -4 weeks post:2 week winter vs. SPC -4 weeks post:4 week winter	1.223	1.187	0.0362
305	SPC -4 weeks post:2 week winter vs. SPC -4 weeks post:8 week winter	1.223	1.248	-0.02497
306	SPC -4 weeks post:2 week winter vs. SPC -8 weeks post:2 week winter	1.223	1.326	-0.1037
307	SPC -4 weeks post:2 week winter vs. SPC -8 weeks post:4 week winter	1.223	1.248	-0.02497
308	SPC -4 weeks post:2 week winter vs. SPC -8 weeks post:8 week winter	1.223	1.265	-0.04261
309	SPC -4 weeks post:4 week winter vs. SPC -4 weeks post:8 week winter	1.187	1.248	-0.06117
310	SPC -4 weeks post:4 week winter vs. SPC -8 weeks post:2 week winter	1.187	1.326	-0.1399
311	SPC -4 weeks post:4 week winter vs. SPC -8 weeks post:4 week winter	1.187	1.248	-0.06117
312	SPC -4 weeks post:4 week winter vs. SPC -8 weeks post:8 week winter	1.187	1.265	-0.07881
313	SPC -4 weeks post:8 week winter vs. SPC -8 weeks post:2 week winter	1.248	1.326	-0.07875
314	SPC -4 weeks post:8 week winter vs. SPC -8 weeks post:4 week winter	1.248	1.248	0
315	SPC -4 weeks post:8 week winter vs. SPC -8 weeks post:8 week winter	1.248	1.265	-0.01764

**2way ANOVA**<br>Multiple comparisons				
316	SPC -8 weeks post:2 week winter vs. SPC -8 weeks post:4 week winter	1.326	1.248	0.07875
317	SPC -8 weeks post:2 week winter vs. SPC -8 weeks post:8 week winter	1.326	1.265	0.06111
318	SPC -8 weeks post:4 week winter vs. SPC -8 weeks post:8 week winter	1.248	1.265	-0.01764

1					
2					
3					
4					
5					
6					
7	Summary	Adjusted P Value			
8					
9	ns	0.9785			
10	ns	>0.9999			
11	ns	>0.9999			
12	ns	0.5836			
13	ns	>0.9999			
14	ns	0.4455			
15	ns	>0.9999			
16	ns	0.9849			
17	ns	0.8507			
18	ns	>0.9999			
19	ns	>0.9999			
20	ns	0.9556			
21	ns	>0.9999			
22	ns	0.4415			
23	****	<0.0001			
24	ns	0.4415			
25	ns	0.1153			
26	ns	>0.9999			
27	ns	0.7331			
28	ns	>0.9999			
29	ns	>0.9999			
30	ns	>0.9999			
31	ns	0.9987			
32	ns	>0.9999			
33	ns	>0.9999			
34	ns	>0.9999			
35	ns	0.9995			
36	ns	>0.9999			
37	ns	0.9995			
38	ns	>0.9999			
39	*	0.0218			
40	ns	>0.9999			
41	ns	0.9742			
42	ns	0.9966			
43	ns	0.9648			
44	ns	>0.9999			
45	ns	0.9142			

**46**	ns	>0.9999			
**47**	ns	>0.9999			
**48**	ns	0.9981			
**49**	ns	>0.9999			
**50**	ns	>0.9999			
**51**	ns	>0.9999			
**52**	ns	>0.9999			
**53**	ns	0.9122			
**54**	***	0.0008			
**55**	ns	0.9122			
**56**	ns	0.5240			
**57**	ns	0.1897			
**58**	ns	0.9991			
**59**	ns	0.1198			
**60**	ns	>0.9999			
**61**	ns	0.7692			
**62**	ns	0.4272			
**63**	ns	0.9985			
**64**	ns	0.9998			
**65**	ns	0.6411			
**66**	ns	0.9998			
**67**	ns	0.1180			
**68**	****	<0.0001			
**69**	ns	0.1180			
**70**	*	0.0178			
**71**	ns	0.9297			
**72**	ns	>0.9999			
**73**	ns	0.8273			
**74**	ns	>0.9999			
**75**	ns	>0.9999			
**76**	ns	0.9436			
**77**	ns	0.8754			
**78**	ns	>0.9999			
**79**	ns	0.8754			
**80**	ns	>0.9999			
**81**	ns	0.2207			
**82**	ns	>0.9999			
**83**	ns	>0.9999			
**84**	ns	0.8540			
**85**	ns	>0.9999			
**86**	ns	>0.9999			
**87**	ns	0.9937			
**88**	ns	>0.9999			
**89**	ns	>0.9999			
**90**	ns	0.9996			

**91**	ns	>0.9999			
**92**	ns	0.8512			
**93**	***	0.0005			
**94**	ns	0.8512			
**95**	ns	0.4216			
**96**	ns	0.7108			
**97**	ns	0.9998			
**98**	ns	>0.9999			
**99**	ns	0.8767			
**100**	ns	0.7741			
**101**	ns	>0.9999			
**102**	ns	0.7741			
**103**	ns	>0.9999			
**104**	ns	0.3249			
**105**	ns	>0.9999			
**106**	ns	>0.9999			
**107**	ns	0.9993			
**108**	ns	0.9698			
**109**	ns	>0.9999			
**110**	ns	>0.9999			
**111**	ns	0.9958			
**112**	ns	>0.9999			
**113**	ns	0.7068			
**114**	***	0.0002			
**115**	ns	0.7068			
**116**	ns	0.2701			
**117**	ns	>0.9999			
**118**	ns	>0.9999			
**119**	ns	0.9998			
**120**	ns	>0.9999			
**121**	ns	0.9998			
**122**	ns	0.9998			
**123**	*	0.0181			
**124**	ns	0.9998			
**125**	ns	0.9648			
**126**	ns	0.9957			
**127**	ns	0.9830			
**128**	ns	>0.9999			
**129**	ns	0.9830			
**130**	ns	>0.9999			
**131**	ns	0.0791			
**132**	ns	>0.9999			
**133**	ns	0.9990			
**134**	ns	>0.9999			
**135**	ns	0.9998			

136	ns	>0.9999			
137	ns	0.8741			
138	***	0.0006			
139	ns	0.8741			
140	ns	0.4557			
141	ns	0.9982			
142	ns	>0.9999			
143	ns	0.7705			
144	***	0.0002			
145	ns	0.7705			
146	ns	0.3271			
147	ns	0.9982			
148	ns	>0.9999			
149	*	0.0332			
150	ns	>0.9999			
151	ns	0.9887			
152	ns	0.7705			
153	***	0.0002			
154	ns	0.7705			
155	ns	0.3271			
156	ns	0.3285			
157	ns	>0.9999			
158	ns	>0.9999			
159	ns	0.3285			
160	ns	0.7719			
161	ns	>0.9999			
162					
163					
164	SE of diff.	N1	N2	q	DF
165					
166	0.0282	10	10	2.277	162
167	0.0282	10	10	1.043	162
168	0.0282	10	10	0.8719	162
169	0.0282	10	10	3.442	162
170	0.0282	10	10	0.845	162
171	0.0282	10	10	3.705	162
172	0.0282	10	10	0.5101	162
173	0.0282	10	10	2.199	162
174	0.0282	10	10	2.869	162
175	0.0282	10	10	0.9122	162
176	0.0282	10	10	0.6454	162
177	0.0282	10	10	2.461	162
178	0.0282	10	10	0.6454	162
179	0.0282	10	10	3.713	162
180	0.0282	10	10	7.662	162

181	0.0282	10	10	3.713	162
182	0.0282	10	10	4.597	162
183	0.0282	10	10	1.234	162
184	0.0282	10	10	3.149	162
185	0.0282	10	10	1.165	162
186	0.0282	10	10	1.432	162
187	0.0282	10	10	1.428	162
188	0.0282	10	10	1.767	162
189	0.0282	10	10	0.07826	162
190	0.0282	10	10	0.5925	162
191	0.0282	10	10	1.365	162
192	0.0282	10	10	1.631	162
193	0.0282	10	10	0.1838	162
194	0.0282	10	10	1.631	162
195	0.0282	10	10	1.436	162
196	0.0282	10	10	5.385	162
197	0.0282	10	10	1.436	162
198	0.0282	10	10	2.321	162
199	0.0282	10	10	1.915	162
200	0.0282	10	10	2.399	162
201	0.0282	10	10	0.1981	162
202	0.0282	10	10	2.662	162
203	0.0282	10	10	0.533	162
204	0.0282	10	10	1.155	162
205	0.0282	10	10	1.826	162
206	0.0282	10	10	0.1309	162
207	0.0282	10	10	0.3977	162
208	0.0282	10	10	1.418	162
209	0.0282	10	10	0.3977	162
210	0.0282	10	10	2.67	162
211	0.0282	10	10	6.619	162
212	0.0282	10	10	2.67	162
213	0.0282	10	10	3.554	162
214	0.0282	10	10	4.314	162
215	0.0282	10	10	1.717	162
216	0.0282	10	10	4.577	162
217	0.0282	10	10	1.382	162
218	0.0282	10	10	3.07	162
219	0.0282	10	10	3.741	162
220	0.0282	10	10	1.784	162
221	0.0282	10	10	1.517	162
222	0.0282	10	10	3.333	162
223	0.0282	10	10	1.517	162
224	0.0282	10	10	4.585	162
225	0.0282	10	10	8.534	162

226	0.0282	10	10	4.585	162
227	0.0282	10	10	5.469	162
228	0.0282	10	10	2.597	162
229	0.0282	10	10	0.2631	162
230	0.0282	10	10	2.932	162
231	0.0282	10	10	1.243	162
232	0.0282	10	10	0.5726	162
233	0.0282	10	10	2.53	162
234	0.0282	10	10	2.797	162
235	0.0282	10	10	0.9813	162
236	0.0282	10	10	2.797	162
237	0.0282	10	10	0.2711	162
238	0.0282	10	10	4.22	162
239	0.0282	10	10	0.2711	162
240	0.0282	10	10	1.155	162
241	0.0282	10	10	2.86	162
242	0.0282	10	10	0.335	162
243	0.0282	10	10	1.354	162
244	0.0282	10	10	2.024	162
245	0.0282	10	10	0.06718	162
246	0.0282	10	10	0.1997	162
247	0.0282	10	10	1.616	162
248	0.0282	10	10	0.1997	162
249	0.0282	10	10	2.868	162
250	0.0282	10	10	6.817	162
251	0.0282	10	10	2.868	162
252	0.0282	10	10	3.752	162
253	0.0282	10	10	3.195	162
254	0.0282	10	10	1.506	162
255	0.0282	10	10	0.8357	162
256	0.0282	10	10	2.793	162
257	0.0282	10	10	3.06	162
258	0.0282	10	10	1.244	162
259	0.0282	10	10	3.06	162
260	0.0282	10	10	0.008023	162
261	0.0282	10	10	3.957	162
262	0.0282	10	10	0.008023	162
263	0.0282	10	10	0.8924	162
264	0.0282	10	10	1.689	162
265	0.0282	10	10	2.359	162
266	0.0282	10	10	0.4021	162
267	0.0282	10	10	0.1353	162
268	0.0282	10	10	1.951	162
269	0.0282	10	10	0.1353	162
270	0.0282	10	10	3.203	162

271	0.0282	10	10	7.152	162
272	0.0282	10	10	3.203	162
273	0.0282	10	10	4.087	162
274	0.0282	10	10	0.6708	162
275	0.0282	10	10	1.286	162
276	0.0282	10	10	1.553	162
277	0.0282	10	10	0.2621	162
278	0.0282	10	10	1.553	162
279	0.0282	10	10	1.514	162
280	0.0282	10	10	5.463	162
281	0.0282	10	10	1.514	162
282	0.0282	10	10	2.399	162
283	0.0282	10	10	1.957	162
284	0.0282	10	10	2.224	162
285	0.0282	10	10	0.4087	162
286	0.0282	10	10	2.224	162
287	0.0282	10	10	0.8437	162
288	0.0282	10	10	4.793	162
289	0.0282	10	10	0.8437	162
290	0.0282	10	10	1.728	162
291	0.0282	10	10	0.2668	162
292	0.0282	10	10	1.548	162
293	0.0282	10	10	0.2668	162
294	0.0282	10	10	2.801	162
295	0.0282	10	10	6.75	162
296	0.0282	10	10	2.801	162
297	0.0282	10	10	3.685	162
298	0.0282	10	10	1.815	162
299	0.0282	10	10	0	162
300	0.0282	10	10	3.068	162
301	0.0282	10	10	7.017	162
302	0.0282	10	10	3.068	162
303	0.0282	10	10	3.952	162
304	0.0282	10	10	1.815	162
305	0.0282	10	10	1.252	162
306	0.0282	10	10	5.201	162
307	0.0282	10	10	1.252	162
308	0.0282	10	10	2.137	162
309	0.0282	10	10	3.068	162
310	0.0282	10	10	7.017	162
311	0.0282	10	10	3.068	162
312	0.0282	10	10	3.952	162
313	0.0282	10	10	3.949	162
314	0.0282	10	10	0	162
315	0.0282	10	10	0.8843	162

**316**	0.0282	10	10	3.949	162
**317**	0.0282	10	10	3.065	162
**318**	0.0282	10	10	0.8843	162

Data analyzed: Condition factor FW grouped format

Source of Variation	Degrees of Freedom	Sum of Squares	Mean square
Pretreatment	2	0.00637	0.003185
Treatment	5	0.1504	0.03008
Interaction	10	0.1266	0.01266
Residual (error)	162	0.6442	0.003977
Total	179	0.9276	

Does Pretreatment have the same effect at all values of Treatment?

Interaction accounts for 13.65% of the total variance.
F = 3.18. DFn=10 DFd=162
The P value = 0.0009
If there is no interaction overall, there is a 0.093% chance of randomly observing so much interaction in an experiment of this size. The interaction is considered extremely significant.

Since the interaction is statistically significant, the P values that follow for the row and column effects are difficult to interpret.

Does Pretreatment affect the result?

Pretreatment accounts for 0.69% of the total variance.
F = 0.80. DFn=2 DFd=162
The P value = 0.4507
If Pretreatment has no effect overall, there is a 45% chance of randomly observing an effect this big (or bigger) in an experiment of this size. The effect is considered not significant.

Does Treatment affect the result?

Treatment accounts for 16.21% of the total variance.
F = 7.56. DFn=5 DFd=162
The P value is < 0.0001
If Treatment has no effect overall, there is a less than 0.01% chance of randomly observing an this big (or bigger) in an experiment of this size. The effect is considered extremely significant.

**2way ANOVA**<br>Tabular results				
1	Table Analyzed	Condition factor SW grouped format		
2				
3	Two-way ANOVA	Ordinary		
4	Alpha	0.05		
5				
6	Source of Variation	% of total variation	P value	P value summary
7	Interaction	2.549	0.0906	ns
8	Treatment	9.465	<0.0001	****
9	Pretreatment	6.338	<0.0001	****
10				
11	ANOVA table	SS (Type III)	DF	MS
12	Interaction	0.04026	4	0.01007
13	Treatment	0.1495	2	0.07475
14	Pretreatment	0.1001	2	0.05005
15	Residual	1.289	260	0.004959
16				
17	Number of missing values	1		

1		
2		
3		
4		
5		
6	Significant?	
7	No	
8	Yes	
9	Yes	
10		
11	F (DFn, DFd)	P value
12	F (4, 260) = 2.03	P=0.0906
13	F (2, 260) = 15.07	P<0.0001
14	F (2, 260) = 10.09	P<0.0001
15		
16		
17		

2way ANOVA
Multiple comparisons

1	Compare cell means regardless of rows and columns				
2					
3	Number of families	1			
4	Number of comparisons per family	36			
5	Alpha	0.05			
6					
7	Tukey's multiple comparisons test	Mean Diff.	95.00% CI of diff.	Significant?	Summary
8					
9	SW_1:2 week winter vs. SW_1:4 week winter	0.01896	-0.03792 to 0.07583	No	ns
10	SW_1:2 week winter vs. SW_1:8 week winter	0.05924	0.002369 to 0.1161	Yes	*
11	SW_1:2 week winter vs. SW_2:2 week winter	0.07014	0.01278 to 0.1275	Yes	**
12	SW_1:2 week winter vs. SW_2:4 week winter	0.0607	0.003829 to 0.1176	Yes	*
13	SW_1:2 week winter vs. SW_2:8 week winter	0.102	0.04515 to 0.1589	Yes	****
14	SW_1:2 week winter vs. SW_3:2 week winter	0.0892	0.03232 to 0.1461	Yes	****
15	SW_1:2 week winter vs. SW_3:4 week winter	0.03874	-0.01813 to 0.09562	No	ns
16	SW_1:2 week winter vs. SW_3:8 week winter	0.09492	0.03805 to 0.1518	Yes	****
17	SW_1:4 week winter vs. SW_1:8 week winter	0.04029	-0.01659 to 0.09716	No	ns
18	SW_1:4 week winter vs. SW_2:2 week winter	0.05119	-0.006177 to 0.1085	No	ns
19	SW_1:4 week winter vs. SW_2:4 week winter	0.04175	-0.01513 to 0.09862	No	ns
20	SW_1:4 week winter vs. SW_2:8 week winter	0.08307	0.02619 to 0.1399	Yes	***
21	SW_1:4 week winter vs. SW_3:2 week winter	0.07024	0.01337 to 0.1271	Yes	**
22	SW_1:4 week winter vs. SW_3:4 week winter	0.01978	-0.03709 to 0.07666	No	ns
23	SW_1:4 week winter vs. SW_3:8 week winter	0.07596	0.01909 to 0.1328	Yes	**
24	SW_1:8 week winter vs. SW_2:2 week winter	0.0109	-0.04646 to 0.06826	No	ns
25	SW_1:8 week winter vs. SW_2:4 week winter	0.001461	-0.05541 to 0.05834	No	ns
26	SW_1:8 week winter vs. SW_2:8 week winter	0.04278	-0.01409 to 0.09966	No	ns
27	SW_1:8 week winter vs. SW_3:2 week winter	0.02996	-0.02692 to 0.08683	No	ns
28	SW_1:8 week winter vs. SW_3:4 week winter	-0.0205	-0.07738 to 0.03637	No	ns
29	SW_1:8 week winter vs. SW_3:8 week winter	0.03568	-0.0212 to 0.09255	No	ns
30	SW_2:2 week winter vs. SW_2:4 week winter	-0.009439	-0.0668 to 0.04792	No	ns
31	SW_2:2 week winter vs. SW_2:8 week winter	0.03188	-0.02548 to 0.08924	No	ns
32	SW_2:2 week winter vs. SW_3:2 week winter	0.01906	-0.03831 to 0.07642	No	ns
33	SW_2:2 week winter vs. SW_3:4 week winter	-0.0314	-0.08876 to 0.02596	No	ns
34	SW_2:2 week winter vs. SW_3:8 week winter	0.02478	-0.03259 to 0.08214	No	ns
35	SW_2:4 week winter vs. SW_2:8 week winter	0.04132	-0.01555 to 0.09819	No	ns
36	SW_2:4 week winter vs. SW_3:2 week winter	0.0285	-0.02838 to 0.08537	No	ns
37	SW_2:4 week winter vs. SW_3:4 week winter	-0.02196	-0.07884 to 0.03491	No	ns
38	SW_2:4 week winter vs. SW_3:8 week winter	0.03422	-0.02266 to 0.09109	No	ns
39	SW_2:8 week winter vs. SW_3:2 week winter	-0.01282	-0.0697 to 0.04405	No	ns
40	SW_2:8 week winter vs. SW_3:4 week winter	-0.06328	-0.1202 to -0.006408	Yes	*
41	SW_2:8 week winter vs. SW_3:8 week winter	-0.007104	-0.06398 to 0.04977	No	ns
42	SW_3:2 week winter vs. SW_3:4 week winter	-0.05046	-0.1073 to 0.006417	No	ns
43	SW_3:2 week winter vs. SW_3:8 week winter	0.005721	-0.05115 to 0.0626	No	ns
44	SW_3:4 week winter vs. SW_3:8 week winter	0.05618	-0.0006958 to 0.1131	No	ns
45					

2way ANOVA<br>Multiple comparisons					
46					
47	Test details	Mean 1	Mean 2	Mean Diff.	SE of diff.
48					
49	SW_1:2 week winter vs. SW_1:4 week winter	1.276	1.257	0.01896	0.01818
50	SW_1:2 week winter vs. SW_1:8 week winter	1.276	1.216	0.05924	0.01818
51	SW_1:2 week winter vs. SW_2:2 week winter	1.276	1.205	0.07014	0.01834
52	SW_1:2 week winter vs. SW_2:4 week winter	1.276	1.215	0.0607	0.01818
53	SW_1:2 week winter vs. SW_2:8 week winter	1.276	1.174	0.102	0.01818
54	SW_1:2 week winter vs. SW_3:2 week winter	1.276	1.186	0.0892	0.01818
55	SW_1:2 week winter vs. SW_3:4 week winter	1.276	1.237	0.03874	0.01818
56	SW_1:2 week winter vs. SW_3:8 week winter	1.276	1.181	0.09492	0.01818
57	SW_1:4 week winter vs. SW_1:8 week winter	1.257	1.216	0.04029	0.01818
58	SW_1:4 week winter vs. SW_2:2 week winter	1.257	1.205	0.05119	0.01834
59	SW_1:4 week winter vs. SW_2:4 week winter	1.257	1.215	0.04175	0.01818
60	SW_1:4 week winter vs. SW_2:8 week winter	1.257	1.174	0.08307	0.01818
61	SW_1:4 week winter vs. SW_3:2 week winter	1.257	1.186	0.07024	0.01818
62	SW_1:4 week winter vs. SW_3:4 week winter	1.257	1.237	0.01978	0.01818
63	SW_1:4 week winter vs. SW_3:8 week winter	1.257	1.181	0.07596	0.01818
64	SW_1:8 week winter vs. SW_2:2 week winter	1.216	1.205	0.0109	0.01834
65	SW_1:8 week winter vs. SW_2:4 week winter	1.216	1.215	0.001461	0.01818
66	SW_1:8 week winter vs. SW_2:8 week winter	1.216	1.174	0.04278	0.01818
67	SW_1:8 week winter vs. SW_3:2 week winter	1.216	1.186	0.02996	0.01818
68	SW_1:8 week winter vs. SW_3:4 week winter	1.216	1.237	-0.0205	0.01818
69	SW_1:8 week winter vs. SW_3:8 week winter	1.216	1.181	0.03568	0.01818
70	SW_2:2 week winter vs. SW_2:4 week winter	1.205	1.215	-0.009439	0.01834
71	SW_2:2 week winter vs. SW_2:8 week winter	1.205	1.174	0.03188	0.01834
72	SW_2:2 week winter vs. SW_3:2 week winter	1.205	1.186	0.01906	0.01834
73	SW_2:2 week winter vs. SW_3:4 week winter	1.205	1.237	-0.0314	0.01834
74	SW_2:2 week winter vs. SW_3:8 week winter	1.205	1.181	0.02478	0.01834
75	SW_2:4 week winter vs. SW_2:8 week winter	1.215	1.174	0.04132	0.01818
76	SW_2:4 week winter vs. SW_3:2 week winter	1.215	1.186	0.0285	0.01818
77	SW_2:4 week winter vs. SW_3:4 week winter	1.215	1.237	-0.02196	0.01818
78	SW_2:4 week winter vs. SW_3:8 week winter	1.215	1.181	0.03422	0.01818
79	SW_2:8 week winter vs. SW_3:2 week winter	1.174	1.186	-0.01282	0.01818
80	SW_2:8 week winter vs. SW_3:4 week winter	1.174	1.237	-0.06328	0.01818
81	SW_2:8 week winter vs. SW_3:8 week winter	1.174	1.181	-0.007104	0.01818
82	SW_3:2 week winter vs. SW_3:4 week winter	1.186	1.237	-0.05046	0.01818
83	SW_3:2 week winter vs. SW_3:8 week winter	1.186	1.181	0.005721	0.01818
84	SW_3:4 week winter vs. SW_3:8 week winter	1.237	1.181	0.05618	0.01818

1				
2				
3				
4				
5				
6				
7	Adjusted P Value			
8				
9	0.9813			
10	0.0340			
11	0.0051			
12	0.0265			
13	<0.0001			
14	<0.0001			
15	0.4547			
16	<0.0001			
17	0.3986			
18	0.1230			
19	0.3483			
20	0.0003			
21	0.0044			
22	0.9755			
23	0.0013			
24	0.9996			
25	>0.9999			
26	0.3148			
27	0.7774			
28	0.9696			
29	0.5711			
30	0.9999			
31	0.7221			
32	0.9817			
33	0.7385			
34	0.9146			
35	0.3627			
36	0.8216			
37	0.9542			
38	0.6268			
39	0.9987			
40	0.0168			
41	>0.9999			
42	0.1279			
43	>0.9999			
44	0.0558			
45				

46				
47	N1	N2	q	DF
48				
49	30	30	1.474	260
50	30	30	4.608	260
51	30	29	5.409	260
52	30	30	4.722	260
53	30	30	7.936	260
54	30	30	6.938	260
55	30	30	3.013	260
56	30	30	7.383	260
57	30	30	3.134	260
58	30	29	3.948	260
59	30	30	3.247	260
60	30	30	6.461	260
61	30	30	5.464	260
62	30	30	1.539	260
63	30	30	5.909	260
64	30	29	0.8406	260
65	30	30	0.1136	260
66	30	30	3.328	260
67	30	30	2.33	260
68	30	30	1.595	260
69	30	30	2.775	260
70	29	30	0.7279	260
71	29	30	2.459	260
72	29	30	1.47	260
73	29	30	2.422	260
74	29	30	1.911	260
75	30	30	3.214	260
76	30	30	2.216	260
77	30	30	1.708	260
78	30	30	2.661	260
79	30	30	0.9975	260
80	30	30	4.922	260
81	30	30	0.5525	260
82	30	30	3.925	260
83	30	30	0.445	260
84	30	30	4.37	260

Data analyzed: Condition factor SW grouped format

Source of Variation	Degrees of Freedom	Sum of Squares	Mean square
Pretreatment	2	0.1001	0.05005
Treatment	2	0.1495	0.07475
Interaction	4	0.04026	0.01007
Residual (error)	260	1.289	0.004959
Total	268		

Does Pretreatment have the same effect at all values of Treatment?

Interaction accounts for approximately 2.55% of the total variance.
F = 2.03. DFn=4 DFd=260
The P value = 0.0906
If there is no interaction overall, there is a 9.1% chance of randomly observing so much interaction in an experiment of this size. The interaction is considered not quite significant.

Does Pretreatment affect the result?

Pretreatment accounts for approximately 6.34% of the total variance.
F = 10.09. DFn=2 DFd=260
The P value is < 0.0001
If Pretreatment has no effect overall, there is a less than 0.01% chance of randomly observing an effect this big (or bigger) in an experiment of this size. The effect is considered extremely significant.

Does Treatment affect the result?

Treatment accounts for approximately 9.47% of the total variance.
F = 15.07. DFn=2 DFd=260
The P value is < 0.0001
If Treatment has no effect overall, there is a less than 0.01% chance of randomly observing an this big (or bigger) in an experiment of this size. The effect is considered extremely significant.
