# Supplementary material for: RNA profiling identifies novel, photoperiod-history dependent markers associated with enhanced saltwater performance in juvenile Atlantic salmon
Source: PLoS One. 2020 Apr 8;15(4):e0227496. doi: 10.1371/journal.pone.0227496 (PMC7141700; doi:10.1371/journal.pone.0227496)
Supplement: S6 Table — Tables showing the 2-way ANOVA and multiple comparison results for the expression of the genes measured in experiment 1 and shown in Fig 2. (PDF) [file pone.0227496.s007.pdf]

2way ANOVA
Tabular results

1	Table Analyzed	Cand.genes 2013 NKA a1b				
2						
3	Two-way ANOVA	Ordinary				
4	Alpha	0.05				
5						
6	Source of Variation	% of total variation	P value	P value summary	Significant?	
7	Interaction	22.93	<0.0001	****	Yes	
8	Time	18.46	<0.0001	****	Yes	
9	Treatment	45.88	<0.0001	****	Yes	
10						
11	ANOVA table	SS (Type III)	DF	MS	F (DFn, DFd)	P value
12	Interaction	3200212	4	800053	F (4, 44) = 27.78	P<0.0001
13	Time	2575743	2	1287872	F (2, 44) = 44.72	P<0.0001
14	Treatment	6402464	2	3201232	F (2, 44) = 111.2	P<0.0001
15	Residual	1267089	44	28797		
16						
17	Number of missing values	1				

2way ANOVA<br>Multiple comparisons					
1	Compare cell means regardless of rows and columns				
2					
3	Number of families	1			
4	Number of comparisons per family	36			
5	Alpha	0.05			
6					
7	Tukey's multiple comparisons test	Mean Diff.	95.00% CI of diff.	Significant?	Summary
8					
9	68:LL vs. 68:SP	351.4	31.9 to 670.8	Yes	*
10	68:LL vs. 68:SPLL	230.6	-104.5 to 565.6	No	ns
11	68:LL vs. 89:LL	-170.2	-489.7 to 149.3	No	ns
12	68:LL vs. 89:SP	225.1	-94.34 to 544.6	No	ns
13	68:LL vs. 89:SPLL	-984.4	-1304 to -665	Yes	****
14	68:LL vs. 110:LL	44.45	-275 to 363.9	No	ns
15	68:LL vs. 110:SP	224.1	-95.36 to 543.6	No	ns
16	68:LL vs. 110:SPLL	-992.7	-1312 to -673.2	Yes	****
17	68:SP vs. 68:SPLL	-120.8	-455.9 to 214.3	No	ns
18	68:SP vs. 89:LL	-521.6	-841.1 to -202.1	Yes	***
19	68:SP vs. 89:SP	-126.2	-445.7 to 193.2	No	ns
20	68:SP vs. 89:SPLL	-1336	-1655 to -1016	Yes	****
21	68:SP vs. 110:LL	-306.9	-626.4 to 12.56	No	ns
22	68:SP vs. 110:SP	-127.3	-446.7 to 192.2	No	ns
23	68:SP vs. 110:SPLL	-1344	-1664 to -1025	Yes	****
24	68:SPLL vs. 89:LL	-400.8	-735.9 to -65.73	Yes	**
25	68:SPLL vs. 89:SP	-5.439	-340.5 to 329.6	No	ns
26	68:SPLL vs. 89:SPLL	-1215	-1550 to -879.9	Yes	****
27	68:SPLL vs. 110:LL	-186.1	-521.2 to 148.9	No	ns
28	68:SPLL vs. 110:SP	-6.455	-341.5 to 328.6	No	ns
29	68:SPLL vs. 110:SPLL	-1223	-1558 to -888.2	Yes	****
30	89:LL vs. 89:SP	395.4	75.88 to 714.8	Yes	**
31	89:LL vs. 89:SPLL	-814.2	-1134 to -494.7	Yes	****
32	89:LL vs. 110:LL	214.7	-104.8 to 534.1	No	ns
33	89:LL vs. 110:SP	394.3	74.86 to 713.8	Yes	**
34	89:LL vs. 110:SPLL	-822.5	-1142 to -503	Yes	****
35	89:SP vs. 89:SPLL	-1210	-1529 to -890.1	Yes	****
36	89:SP vs. 110:LL	-180.7	-500.2 to 138.8	No	ns
37	89:SP vs. 110:SP	-1.016	-320.5 to 318.5	No	ns
38	89:SP vs. 110:SPLL	-1218	-1537 to -898.4	Yes	****
39	89:SPLL vs. 110:LL	1029	709.4 to 1348	Yes	****
40	89:SPLL vs. 110:SP	1209	889.1 to 1528	Yes	****
41	89:SPLL vs. 110:SPLL	-8.289	-327.8 to 311.2	No	ns
42	110:LL vs. 110:SP	179.7	-139.8 to 499.1	No	ns
43	110:LL vs. 110:SPLL	-1037	-1357 to -717.7	Yes	****
44	110:SP vs. 110:SPLL	-1217	-1536 to -897.4	Yes	****
45					

2way ANOVA
Multiple comparisons

46					
47	Test details	Mean 1	Mean 2	Mean Diff.	SE of diff.
48					
49	68:LL vs. 68:SP	503.5	152.2	351.4	97.98
50	68:LL vs. 68:SPLL	503.5	273	230.6	102.8
51	68:LL vs. 89:LL	503.5	673.8	-170.2	97.98
52	68:LL vs. 89:SP	503.5	278.4	225.1	97.98
53	68:LL vs. 89:SPLL	503.5	1488	-984.4	97.98
54	68:LL vs. 110:LL	503.5	459.1	44.45	97.98
55	68:LL vs. 110:SP	503.5	279.4	224.1	97.98
56	68:LL vs. 110:SPLL	503.5	1496	-992.7	97.98
57	68:SP vs. 68:SPLL	152.2	273	-120.8	102.8
58	68:SP vs. 89:LL	152.2	673.8	-521.6	97.98
59	68:SP vs. 89:SP	152.2	278.4	-126.2	97.98
60	68:SP vs. 89:SPLL	152.2	1488	-1336	97.98
61	68:SP vs. 110:LL	152.2	459.1	-306.9	97.98
62	68:SP vs. 110:SP	152.2	279.4	-127.3	97.98
63	68:SP vs. 110:SPLL	152.2	1496	-1344	97.98
64	68:SPLL vs. 89:LL	273	673.8	-400.8	102.8
65	68:SPLL vs. 89:SP	273	278.4	-5.439	102.8
66	68:SPLL vs. 89:SPLL	273	1488	-1215	102.8
67	68:SPLL vs. 110:LL	273	459.1	-186.1	102.8
68	68:SPLL vs. 110:SP	273	279.4	-6.455	102.8
69	68:SPLL vs. 110:SPLL	273	1496	-1223	102.8
70	89:LL vs. 89:SP	673.8	278.4	395.4	97.98
71	89:LL vs. 89:SPLL	673.8	1488	-814.2	97.98
72	89:LL vs. 110:LL	673.8	459.1	214.7	97.98
73	89:LL vs. 110:SP	673.8	279.4	394.3	97.98
74	89:LL vs. 110:SPLL	673.8	1496	-822.5	97.98
75	89:SP vs. 89:SPLL	278.4	1488	-1210	97.98
76	89:SP vs. 110:LL	278.4	459.1	-180.7	97.98
77	89:SP vs. 110:SP	278.4	279.4	-1.016	97.98
78	89:SP vs. 110:SPLL	278.4	1496	-1218	97.98
79	89:SPLL vs. 110:LL	1488	459.1	1029	97.98
80	89:SPLL vs. 110:SP	1488	279.4	1209	97.98
81	89:SPLL vs. 110:SPLL	1488	1496	-8.289	97.98
82	110:LL vs. 110:SP	459.1	279.4	179.7	97.98
83	110:LL vs. 110:SPLL	459.1	1496	-1037	97.98
84	110:SP vs. 110:SPLL	279.4	1496	-1217	97.98

1				
2				
3				
4				
5				
6				
7	Adjusted P Value			
8				
9	0.0215			
10	0.3970			
11	0.7208			
12	0.3654			
13	<0.0001			
14	>0.9999			
15	0.3713			
16	<0.0001			
17	0.9575			
18	0.0001			
19	0.9295			
20	<0.0001			
21	0.0683			
22	0.9264			
23	<0.0001			
24	0.0090			
25	>0.9999			
26	<0.0001			
27	0.6747			
28	>0.9999			
29	<0.0001			
30	0.0061			
31	<0.0001			
32	0.4289			
33	0.0063			
34	<0.0001			
35	<0.0001			
36	0.6536			
37	>0.9999			
38	<0.0001			
39	<0.0001			
40	<0.0001			
41	>0.9999			
42	0.6603			
43	<0.0001			
44	<0.0001			
45				

46				
47	N1	N2	q	DF
48				
49	6	6	5.072	44
50	6	5	3.173	44
51	6	6	2.457	44
52	6	6	3.25	44
53	6	6	14.21	44
54	6	6	0.6417	44
55	6	6	3.235	44
56	6	6	14.33	44
57	6	5	1.663	44
58	6	6	7.529	44
59	6	6	1.822	44
60	6	6	19.28	44
61	6	6	4.43	44
62	6	6	1.837	44
63	6	6	19.4	44
64	5	6	5.516	44
65	5	6	0.07486	44
66	5	6	16.72	44
67	5	6	2.561	44
68	5	6	0.08884	44
69	5	6	16.84	44
70	6	6	5.707	44
71	6	6	11.75	44
72	6	6	3.099	44
73	6	6	5.692	44
74	6	6	11.87	44
75	6	6	17.46	44
76	6	6	2.608	44
77	6	6	0.01466	44
78	6	6	17.58	44
79	6	6	14.85	44
80	6	6	17.44	44
81	6	6	0.1196	44
82	6	6	2.593	44
83	6	6	14.97	44
84	6	6	17.56	44

2way ANOVA
Tabular results

1	Table Analyzed	Cand.genes 2013 CFTR I				
2						
3	Two-way ANOVA	Ordinary				
4	Alpha	0.05				
5						
6	Source of Variation	% of total variation	P value	P value summary	Significant?	
7	Interaction	20.15	<0.0001	****	Yes	
8	Time	23.96	<0.0001	****	Yes	
9	Treatment	43.5	<0.0001	****	Yes	
10						
11	ANOVA table	SS (Type III)	DF	MS	F (DFn, DFd)	P value
12	Interaction	17475	4	4369	F (4, 44) = 21.62	P<0.0001
13	Time	20779	2	10390	F (2, 44) = 51.41	P<0.0001
14	Treatment	37729	2	18865	F (2, 44) = 93.34	P<0.0001
15	Residual	8893	44	202.1		
16						
17	Number of missing values	1				

2way ANOVA<br>Multiple comparisons					
1	Compare cell means regardless of rows and columns				
2					
3	Number of families	1			
4	Number of comparisons per family	36			
5	Alpha	0.05			
6					
7	Tukey's multiple comparisons test	Mean Diff.	95.00% CI of diff.	Significant?	Summary
8					
9	68:LL vs. 68:SP	47.93	21.17 to 74.69	Yes	****
10	68:LL vs. 68:SPLL	38.37	10.3 to 66.44	Yes	**
11	68:LL vs. 89:LL	-14.34	-41.1 to 12.43	No	ns
12	68:LL vs. 89:SP	35.01	8.249 to 61.78	Yes	**
13	68:LL vs. 89:SPLL	-60.36	-87.13 to -33.6	Yes	****
14	68:LL vs. 110:LL	-10.06	-36.82 to 16.71	No	ns
15	68:LL vs. 110:SP	26.85	0.08768 to 53.61	Yes	*
16	68:LL vs. 110:SPLL	-59.61	-86.37 to -32.84	Yes	****
17	68:SP vs. 68:SPLL	-9.556	-37.63 to 18.51	No	ns
18	68:SP vs. 89:LL	-62.27	-89.03 to -35.5	Yes	****
19	68:SP vs. 89:SP	-12.92	-39.68 to 13.85	No	ns
20	68:SP vs. 89:SPLL	-108.3	-135.1 to -81.53	Yes	****
21	68:SP vs. 110:LL	-57.98	-84.75 to -31.22	Yes	****
22	68:SP vs. 110:SP	-21.08	-47.84 to 5.686	No	ns
23	68:SP vs. 110:SPLL	-107.5	-134.3 to -80.77	Yes	****
24	68:SPLL vs. 89:LL	-52.71	-80.78 to -24.64	Yes	****
25	68:SPLL vs. 89:SP	-3.36	-31.43 to 24.71	No	ns
26	68:SPLL vs. 89:SPLL	-98.74	-126.8 to -70.67	Yes	****
27	68:SPLL vs. 110:LL	-48.43	-76.5 to -20.36	Yes	****
28	68:SPLL vs. 110:SP	-11.52	-39.59 to 16.55	No	ns
29	68:SPLL vs. 110:SPLL	-97.98	-126.1 to -69.91	Yes	****
30	89:LL vs. 89:SP	49.35	22.59 to 76.11	Yes	****
31	89:LL vs. 89:SPLL	-46.03	-72.79 to -19.26	Yes	****
32	89:LL vs. 110:LL	4.281	-22.48 to 31.04	No	ns
33	89:LL vs. 110:SP	41.19	14.42 to 67.95	Yes	***
34	89:LL vs. 110:SPLL	-45.27	-72.03 to -18.51	Yes	****
35	89:SP vs. 89:SPLL	-95.38	-122.1 to -68.61	Yes	****
36	89:SP vs. 110:LL	-45.07	-71.83 to -18.31	Yes	****
37	89:SP vs. 110:SP	-8.162	-34.93 to 18.6	No	ns
38	89:SP vs. 110:SPLL	-94.62	-121.4 to -67.86	Yes	****
39	89:SPLL vs. 110:LL	50.31	23.54 to 77.07	Yes	****
40	89:SPLL vs. 110:SP	87.22	60.45 to 114	Yes	****
41	89:SPLL vs. 110:SPLL	0.7564	-26.01 to 27.52	No	ns
42	110:LL vs. 110:SP	36.91	10.14 to 63.67	Yes	**
43	110:LL vs. 110:SPLL	-49.55	-76.32 to -22.79	Yes	****
44	110:SP vs. 110:SPLL	-86.46	-113.2 to -59.7	Yes	****
45					

2way ANOVA<br>Multiple comparisons					
46					
47	Test details	Mean 1	Mean 2	Mean Diff.	SE of diff.
48					
49	68:LL vs. 68:SP	58.84	10.92	47.93	8.208
50	68:LL vs. 68:SPLL	58.84	20.47	38.37	8.608
51	68:LL vs. 89:LL	58.84	73.18	-14.34	8.208
52	68:LL vs. 89:SP	58.84	23.83	35.01	8.208
53	68:LL vs. 89:SPLL	58.84	119.2	-60.36	8.208
54	68:LL vs. 110:LL	58.84	68.9	-10.06	8.208
55	68:LL vs. 110:SP	58.84	31.99	26.85	8.208
56	68:LL vs. 110:SPLL	58.84	118.5	-59.61	8.208
57	68:SP vs. 68:SPLL	10.92	20.47	-9.556	8.608
58	68:SP vs. 89:LL	10.92	73.18	-62.27	8.208
59	68:SP vs. 89:SP	10.92	23.83	-12.92	8.208
60	68:SP vs. 89:SPLL	10.92	119.2	-108.3	8.208
61	68:SP vs. 110:LL	10.92	68.9	-57.98	8.208
62	68:SP vs. 110:SP	10.92	31.99	-21.08	8.208
63	68:SP vs. 110:SPLL	10.92	118.5	-107.5	8.208
64	68:SPLL vs. 89:LL	20.47	73.18	-52.71	8.608
65	68:SPLL vs. 89:SP	20.47	23.83	-3.36	8.608
66	68:SPLL vs. 89:SPLL	20.47	119.2	-98.74	8.608
67	68:SPLL vs. 110:LL	20.47	68.9	-48.43	8.608
68	68:SPLL vs. 110:SP	20.47	31.99	-11.52	8.608
69	68:SPLL vs. 110:SPLL	20.47	118.5	-97.98	8.608
70	89:LL vs. 89:SP	73.18	23.83	49.35	8.208
71	89:LL vs. 89:SPLL	73.18	119.2	-46.03	8.208
72	89:LL vs. 110:LL	73.18	68.9	4.281	8.208
73	89:LL vs. 110:SP	73.18	31.99	41.19	8.208
74	89:LL vs. 110:SPLL	73.18	118.5	-45.27	8.208
75	89:SP vs. 89:SPLL	23.83	119.2	-95.38	8.208
76	89:SP vs. 110:LL	23.83	68.9	-45.07	8.208
77	89:SP vs. 110:SP	23.83	31.99	-8.162	8.208
78	89:SP vs. 110:SPLL	23.83	118.5	-94.62	8.208
79	89:SPLL vs. 110:LL	119.2	68.9	50.31	8.208
80	89:SPLL vs. 110:SP	119.2	31.99	87.22	8.208
81	89:SPLL vs. 110:SPLL	119.2	118.5	0.7564	8.208
82	110:LL vs. 110:SP	68.9	31.99	36.91	8.208
83	110:LL vs. 110:SPLL	68.9	118.5	-49.55	8.208
84	110:SP vs. 110:SPLL	31.99	118.5	-86.46	8.208

1				
2				
3				
4				
5				
6				
7	Adjusted P Value			
8				
9	<0.0001			
10	0.0017			
11	0.7151			
12	0.0031			
13	<0.0001			
14	0.9464			
15	0.0487			
16	<0.0001			
17	0.9696			
18	<0.0001			
19	0.8135			
20	<0.0001			
21	<0.0001			
22	0.2294			
23	<0.0001			
24	<0.0001			
25	>0.9999			
26	<0.0001			
27	<0.0001			
28	0.9139			
29	<0.0001			
30	<0.0001			
31	<0.0001			
32	0.9998			
33	0.0003			
34	<0.0001			
35	<0.0001			
36	<0.0001			
37	0.9845			
38	<0.0001			
39	<0.0001			
40	<0.0001			
41	>0.9999			
42	0.0015			
43	<0.0001			
44	<0.0001			
45				

46				
47	N1	N2	q	DF
48				
49	6	6	8.258	44
50	6	5	6.304	44
51	6	6	2.47	44
52	6	6	6.033	44
53	6	6	10.4	44
54	6	6	1.733	44
55	6	6	4.627	44
56	6	6	10.27	44
57	6	5	1.57	44
58	6	6	10.73	44
59	6	6	2.225	44
60	6	6	18.66	44
61	6	6	9.991	44
62	6	6	3.632	44
63	6	6	18.53	44
64	5	6	8.659	44
65	5	6	0.5519	44
66	5	6	16.22	44
67	5	6	7.956	44
68	5	6	1.893	44
69	5	6	16.1	44
70	6	6	8.503	44
71	6	6	7.931	44
72	6	6	0.7376	44
73	6	6	7.097	44
74	6	6	7.8	44
75	6	6	16.43	44
76	6	6	7.765	44
77	6	6	1.406	44
78	6	6	16.3	44
79	6	6	8.668	44
80	6	6	15.03	44
81	6	6	0.1303	44
82	6	6	6.359	44
83	6	6	8.538	44
84	6	6	14.9	44

2way ANOVA
Tabular results

1	Table Analyzed	Cand.genes 2013 CAPN2				
2						
3	Two-way ANOVA	Ordinary				
4	Alpha	0.05				
5						
6	Source of Variation	% of total variation	P value	P value summary	Significant?	
7	Interaction	23.37	<0.0001	****	Yes	
8	Time	13.91	<0.0001	****	Yes	
9	Treatment	42.87	<0.0001	****	Yes	
10						
11	ANOVA table	SS (Type III)	DF	MS	F (DFn, DFd)	P value
12	Interaction	382.7	4	95.67	F (4, 44) = 16	P<0.0001
13	Time	227.8	2	113.9	F (2, 44) = 19.05	P<0.0001
14	Treatment	701.9	2	351	F (2, 44) = 58.69	P<0.0001
15	Residual	263.1	44	5.979		
16						
17	Number of missing values	1				

2way ANOVA
Multiple comparisons

1	Compare cell means regardless of rows and columns								
2									
3	Number of families	1							
4	Number of comparisons per family	36							
5	Alpha	0.05							
6									
7	Tukey's multiple comparisons test	Mean Diff.	95.00% CI of diff.	Significant?	Summary	Adjusted P Value			
8									
9	68:LL vs. 68:SP	0.2601	-4.343 to 4.864	No	ns	>0.9999			
10	68:LL vs. 68:SPLL	0.1916	-4.637 to 5.02	No	ns	>0.9999			
11	68:LL vs. 89:LL	-2.026	-6.63 to 2.577	No	ns	0.8778			
12	68:LL vs. 89:SP	0.1689	-4.435 to 4.772	No	ns	>0.9999			
13	68:LL vs. 89:SPLL	-10.5	-15.1 to -5.896	Yes	****	<0.0001			
14	68:LL vs. 110:LL	0.05966	-4.544 to 4.663	No	ns	>0.9999			
15	68:LL vs. 110:SP	0.226	-4.377 to 4.829	No	ns	>0.9999			
16	68:LL vs. 110:SPLL	-13.68	-18.28 to -9.078	Yes	****	<0.0001			
17	68:SP vs. 68:SPLL	-0.06845	-4.897 to 4.76	No	ns	>0.9999			
18	68:SP vs. 89:LL	-2.286	-6.89 to 2.317	No	ns	0.7891			
19	68:SP vs. 89:SP	-0.0912	-4.695 to 4.512	No	ns	>0.9999			
20	68:SP vs. 89:SPLL	-10.76	-15.36 to -6.156	Yes	****	<0.0001			
21	68:SP vs. 110:LL	-0.2004	-4.804 to 4.403	No	ns	>0.9999			
22	68:SP vs. 110:SP	-0.03406	-4.638 to 4.569	No	ns	>0.9999			
23	68:SP vs. 110:SPLL	-13.94	-18.54 to -9.338	Yes	****	<0.0001			
24	68:SPLL vs. 89:LL	-2.218	-7.046 to 2.61	No	ns	0.8505			
25	68:SPLL vs. 89:SP	-0.02275	-4.851 to 4.805	No	ns	>0.9999			
26	68:SPLL vs. 89:SPLL	-10.69	-15.52 to -5.863	Yes	****	<0.0001			
27	68:SPLL vs. 110:LL	-0.1319	-4.96 to 4.696	No	ns	>0.9999			
28	68:SPLL vs. 110:SP	0.03439	-4.794 to 4.863	No	ns	>0.9999			
29	68:SPLL vs. 110:SPLL	-13.87	-18.7 to -9.045	Yes	****	<0.0001			
30	89:LL vs. 89:SP	2.195	-2.408 to 6.799	No	ns	0.8230			

2way ANOVA
Multiple comparisons

31	89:LL vs. 89:SPLL	-8.473	-13.08 to -3.87	Yes	****	<0.0001			
32	89:LL vs. 110:LL	2.086	-2.518 to 6.689	No	ns	0.8597			
33	89:LL vs. 110:SP	2.252	-2.351 to 6.856	No	ns	0.8021			
34	89:LL vs. 110:SPLL	-11.66	-16.26 to -7.052	Yes	****	<0.0001			
35	89:SP vs. 89:SPLL	-10.67	-15.27 to -6.065	Yes	****	<0.0001			
36	89:SP vs. 110:LL	-0.1092	-4.713 to 4.494	No	ns	>0.9999			
37	89:SP vs. 110:SP	0.05714	-4.546 to 4.661	No	ns	>0.9999			
38	89:SP vs. 110:SPLL	-13.85	-18.45 to -9.247	Yes	****	<0.0001			
39	89:SPLL vs. 110:LL	10.56	5.956 to 15.16	Yes	****	<0.0001			
40	89:SPLL vs. 110:SP	10.73	6.122 to 15.33	Yes	****	<0.0001			
41	89:SPLL vs. 110:SPLL	-3.182	-7.785 to 1.421	No	ns	0.3910			
42	110:LL vs. 110:SP	0.1663	-4.437 to 4.77	No	ns	>0.9999			
43	110:LL vs. 110:SPLL	-13.74	-18.34 to -9.138	Yes	****	<0.0001			
44	110:SP vs. 110:SPLL	-13.91	-18.51 to -9.304	Yes	****	<0.0001			
45									
46									
47	Test details	Mean 1	Mean 2	Mean Diff.	SE of diff.	N1	N2	q	DF
48									
49	68:LL vs. 68:SP	0.3038	0.04374	0.2601	1.412	6	6	0.2605	44
50	68:LL vs. 68:SPLL	0.3038	0.1122	0.1916	1.481	6	5	0.183	44
51	68:LL vs. 89:LL	0.3038	2.33	-2.026	1.412	6	6	2.03	44
52	68:LL vs. 89:SP	0.3038	0.1349	0.1689	1.412	6	6	0.1691	44
53	68:LL vs. 89:SPLL	0.3038	10.8	-10.5	1.412	6	6	10.52	44
54	68:LL vs. 110:LL	0.3038	0.2441	0.05966	1.412	6	6	0.05976	44
55	68:LL vs. 110:SP	0.3038	0.0778	0.226	1.412	6	6	0.2264	44
56	68:LL vs. 110:SPLL	0.3038	13.99	-13.68	1.412	6	6	13.71	44
57	68:SP vs. 68:SPLL	0.04374	0.1122	-0.06845	1.481	6	5	0.06538	44
58	68:SP vs. 89:LL	0.04374	2.33	-2.286	1.412	6	6	2.29	44
59	68:SP vs. 89:SP	0.04374	0.1349	-0.0912	1.412	6	6	0.09135	44
60	68:SP vs. 89:SPLL	0.04374	10.8	-10.76	1.412	6	6	10.78	44

2way ANOVA<br>Multiple comparisons									
61	68:SP vs. 110:LL	0.04374	0.2441	-0.2004	1.412	6	6	0.2007	44
62	68:SP vs. 110:SP	0.04374	0.0778	-0.03406	1.412	6	6	0.03412	44
63	68:SP vs. 110:SPLL	0.04374	13.99	-13.94	1.412	6	6	13.97	44
64	68:SPLL vs. 89:LL	0.1122	2.33	-2.218	1.481	5	6	2.118	44
65	68:SPLL vs. 89:SP	0.1122	0.1349	-0.02275	1.481	5	6	0.02173	44
66	68:SPLL vs. 89:SPLL	0.1122	10.8	-10.69	1.481	5	6	10.21	44
67	68:SPLL vs. 110:LL	0.1122	0.2441	-0.1319	1.481	5	6	0.126	44
68	68:SPLL vs. 110:SP	0.1122	0.0778	0.03439	1.481	5	6	0.03285	44
69	68:SPLL vs. 110:SPLL	0.1122	13.99	-13.87	1.481	5	6	13.25	44
70	89:LL vs. 89:SP	2.33	0.1349	2.195	1.412	6	6	2.199	44
71	89:LL vs. 89:SPLL	2.33	10.8	-8.473	1.412	6	6	8.488	44
72	89:LL vs. 110:LL	2.33	0.2441	2.086	1.412	6	6	2.09	44
73	89:LL vs. 110:SP	2.33	0.0778	2.252	1.412	6	6	2.256	44
74	89:LL vs. 110:SPLL	2.33	13.99	-11.66	1.412	6	6	11.68	44
75	89:SP vs. 89:SPLL	0.1349	10.8	-10.67	1.412	6	6	10.69	44
76	89:SP vs. 110:LL	0.1349	0.2441	-0.1092	1.412	6	6	0.1094	44
77	89:SP vs. 110:SP	0.1349	0.0778	0.05714	1.412	6	6	0.05724	44
78	89:SP vs. 110:SPLL	0.1349	13.99	-13.85	1.412	6	6	13.87	44
79	89:SPLL vs. 110:LL	10.8	0.2441	10.56	1.412	6	6	10.58	44
80	89:SPLL vs. 110:SP	10.8	0.0778	10.73	1.412	6	6	10.74	44
81	89:SPLL vs. 110:SPLL	10.8	13.99	-3.182	1.412	6	6	3.187	44
82	110:LL vs. 110:SP	0.2441	0.0778	0.1663	1.412	6	6	0.1666	44
83	110:LL vs. 110:SPLL	0.2441	13.99	-13.74	1.412	6	6	13.76	44
84	110:SP vs. 110:SPLL	0.0778	13.99	-13.91	1.412	6	6	13.93	44

2way ANOVA
Tabular results

1	Table Analyzed	Cand.genes 2013 FKBP5				
2						
3	Two-way ANOVA	Ordinary				
4	Alpha	0.05				
5						
6	Source of Variation	% of total variation	P value	P value summary	Significant?	
7	Interaction	10.9	0.0003	***	Yes	
8	Time	14.02	<0.0001	****	Yes	
9	Treatment	53.42	<0.0001	****	Yes	
10						
11	ANOVA table	SS (Type III)	DF	MS	F (DFn, DFd)	P value
12	Interaction	16881	4	4220	F (4, 44) = 6.653	P=0.0003
13	Time	21723	2	10862	F (2, 44) = 17.12	P<0.0001
14	Treatment	82735	2	41368	F (2, 44) = 65.21	P<0.0001
15	Residual	27913	44	634.4		
16						
17	Number of missing values	1				

2way ANOVA
Multiple comparisons

1	Compare cell means regardless of rows and columns								
2									
3	Number of families	1							
4	Number of comparisons per family	36							
5	Alpha	0.05							
6									
7	Tukey's multiple comparisons test	Mean Diff.	95.00% CI of diff.	Significant?	Summary	Adjusted P Value			
8									
9	68:LL vs. 68:SP	-0.3438	-47.76 to 47.07	No	ns	>0.9999			
10	68:LL vs. 68:SPLL	-31.85	-81.58 to 17.88	No	ns	0.4938			
11	68:LL vs. 89:LL	-27.57	-74.99 to 19.84	No	ns	0.6198			
12	68:LL vs. 89:SP	7.866	-39.55 to 55.28	No	ns	0.9998			
13	68:LL vs. 89:SPLL	-124.6	-172 to -77.15	Yes	****	<0.0001			
14	68:LL vs. 110:LL	-39.18	-86.6 to 8.237	No	ns	0.1796			
15	68:LL vs. 110:SP	-9.818	-57.23 to 37.6	No	ns	0.9989			
16	68:LL vs. 110:SPLL	-126.2	-173.6 to -78.79	Yes	****	<0.0001			
17	68:SP vs. 68:SPLL	-31.51	-81.24 to 18.22	No	ns	0.5084			
18	68:SP vs. 89:LL	-27.23	-74.65 to 20.19	No	ns	0.6352			
19	68:SP vs. 89:SP	8.209	-39.21 to 55.63	No	ns	0.9997			
20	68:SP vs. 89:SPLL	-124.2	-171.6 to -76.81	Yes	****	<0.0001			
21	68:SP vs. 110:LL	-38.84	-86.25 to 8.581	No	ns	0.1882			
22	68:SP vs. 110:SP	-9.474	-56.89 to 37.94	No	ns	0.9991			
23	68:SP vs. 110:SPLL	-125.9	-173.3 to -78.44	Yes	****	<0.0001			
24	68:SPLL vs. 89:LL	4.279	-45.45 to 54.01	No	ns	>0.9999			
25	68:SPLL vs. 89:SP	39.72	-10.01 to 89.45	No	ns	0.2142			
26	68:SPLL vs. 89:SPLL	-92.72	-142.4 to -42.99	Yes	****	<0.0001			
27	68:SPLL vs. 110:LL	-7.327	-57.06 to 42.4	No	ns	>0.9999			
28	68:SPLL vs. 110:SP	22.04	-27.7 to 71.77	No	ns	0.8739			
29	68:SPLL vs. 110:SPLL	-94.35	-144.1 to -44.62	Yes	****	<0.0001			
30	89:LL vs. 89:SP	35.44	-11.98 to 82.86	No	ns	0.2904			

2way ANOVA<br>Multiple comparisons									
31	89:LL vs. 89:SPLL	-96.99	-144.4 to -49.58	Yes	****	<0.0001			
32	89:LL vs. 110:LL	-11.61	-59.02 to 35.81	No	ns	0.9964			
33	89:LL vs. 110:SP	17.76	-29.66 to 65.17	No	ns	0.9474			
34	89:LL vs. 110:SPLL	-98.63	-146 to -51.21	Yes	****	<0.0001			
35	89:SP vs. 89:SPLL	-132.4	-179.9 to -85.02	Yes	****	<0.0001			
36	89:SP vs. 110:LL	-47.05	-94.46 to 0.3711	No	ns	0.0533			
37	89:SP vs. 110:SP	-17.68	-65.1 to 29.73	No	ns	0.9486			
38	89:SP vs. 110:SPLL	-134.1	-181.5 to -86.65	Yes	****	<0.0001			
39	89:SPLL vs. 110:LL	85.39	37.97 to 132.8	Yes	****	<0.0001			
40	89:SPLL vs. 110:SP	114.8	67.33 to 162.2	Yes	****	<0.0001			
41	89:SPLL vs. 110:SPLL	-1.636	-49.05 to 45.78	No	ns	>0.9999			
42	110:LL vs. 110:SP	29.36	-18.05 to 76.78	No	ns	0.5389			
43	110:LL vs. 110:SPLL	-87.03	-134.4 to -39.61	Yes	****	<0.0001			
44	110:SP vs. 110:SPLL	-116.4	-163.8 to -68.97	Yes	****	<0.0001			
45									
46									
47	Test details	Mean 1	Mean 2	Mean Diff.	SE of diff.	N1	N2	q	DF
48									
49	68:LL vs. 68:SP	25.62	25.96	-0.3438	14.54	6	6	0.03344	44
50	68:LL vs. 68:SPLL	25.62	57.47	-31.85	15.25	6	5	2.954	44
51	68:LL vs. 89:LL	25.62	53.19	-27.57	14.54	6	6	2.682	44
52	68:LL vs. 89:SP	25.62	17.75	7.866	14.54	6	6	0.7649	44
53	68:LL vs. 89:SPLL	25.62	150.2	-124.6	14.54	6	6	12.11	44
54	68:LL vs. 110:LL	25.62	64.8	-39.18	14.54	6	6	3.81	44
55	68:LL vs. 110:SP	25.62	35.44	-9.818	14.54	6	6	0.9548	44
56	68:LL vs. 110:SPLL	25.62	151.8	-126.2	14.54	6	6	12.27	44
57	68:SP vs. 68:SPLL	25.96	57.47	-31.51	15.25	6	5	2.922	44
58	68:SP vs. 89:LL	25.96	53.19	-27.23	14.54	6	6	2.648	44
59	68:SP vs. 89:SP	25.96	17.75	8.209	14.54	6	6	0.7984	44
60	68:SP vs. 89:SPLL	25.96	150.2	-124.2	14.54	6	6	12.08	44

2way ANOVA<br>Multiple comparisons									
61	68:SP vs. 110:LL	25.96	64.8	-38.84	14.54	6	6	3.777	44
62	68:SP vs. 110:SP	25.96	35.44	-9.474	14.54	6	6	0.9214	44
63	68:SP vs. 110:SPLL	25.96	151.8	-125.9	14.54	6	6	12.24	44
64	68:SPLL vs. 89:LL	57.47	53.19	4.279	15.25	5	6	0.3967	44
65	68:SPLL vs. 89:SP	57.47	17.75	39.72	15.25	5	6	3.683	44
66	68:SPLL vs. 89:SPLL	57.47	150.2	-92.72	15.25	5	6	8.597	44
67	68:SPLL vs. 110:LL	57.47	64.8	-7.327	15.25	5	6	0.6794	44
68	68:SPLL vs. 110:SP	57.47	35.44	22.04	15.25	5	6	2.043	44
69	68:SPLL vs. 110:SPLL	57.47	151.8	-94.35	15.25	5	6	8.749	44
70	89:LL vs. 89:SP	53.19	17.75	35.44	14.54	6	6	3.447	44
71	89:LL vs. 89:SPLL	53.19	150.2	-96.99	14.54	6	6	9.433	44
72	89:LL vs. 110:LL	53.19	64.8	-11.61	14.54	6	6	1.129	44
73	89:LL vs. 110:SP	53.19	35.44	17.76	14.54	6	6	1.727	44
74	89:LL vs. 110:SPLL	53.19	151.8	-98.63	14.54	6	6	9.592	44
75	89:SP vs. 89:SPLL	17.75	150.2	-132.4	14.54	6	6	12.88	44
76	89:SP vs. 110:LL	17.75	64.8	-47.05	14.54	6	6	4.575	44
77	89:SP vs. 110:SP	17.75	35.44	-17.68	14.54	6	6	1.72	44
78	89:SP vs. 110:SPLL	17.75	151.8	-134.1	14.54	6	6	13.04	44
79	89:SPLL vs. 110:LL	150.2	64.8	85.39	14.54	6	6	8.304	44
80	89:SPLL vs. 110:SP	150.2	35.44	114.8	14.54	6	6	11.16	44
81	89:SPLL vs. 110:SPLL	150.2	151.8	-1.636	14.54	6	6	0.1591	44
82	110:LL vs. 110:SP	64.8	35.44	29.36	14.54	6	6	2.856	44
83	110:LL vs. 110:SPLL	64.8	151.8	-87.03	14.54	6	6	8.463	44
84	110:SP vs. 110:SPLL	35.44	151.8	-116.4	14.54	6	6	11.32	44

2way ANOVA
Tabular results

1	Table Analyzed	Cand.genes 2013 TPH1				
2						
3	Two-way ANOVA	Ordinary				
4	Alpha	0.05				
5						
6	Source of Variation	% of total variation	P value	P value summary	Significant?	
7	Interaction	31.94	<0.0001	****	Yes	
8	Time	14.85	<0.0001	****	Yes	
9	Treatment	30	<0.0001	****	Yes	
10						
11	ANOVA table	SS (Type III)	DF	MS	F (DFn, DFd)	P value
12	Interaction	352.6	4	88.14	F (4, 44) = 17.07	P<0.0001
13	Time	163.9	2	81.97	F (2, 44) = 15.88	P<0.0001
14	Treatment	331.1	2	165.6	F (2, 44) = 32.07	P<0.0001
15	Residual	227.1	44	5.162		
16						
17	Number of missing values	1				

2way ANOVA
Multiple comparisons

1	Compare cell means regardless of rows and columns								
2									
3	Number of families	1							
4	Number of comparisons per family	36							
5	Alpha	0.05							
6									
7	Tukey's multiple comparisons test	Mean Diff.	95.00% CI of diff.	Significant?	Summary	Adjusted P Value			
8									
9	68:LL vs. 68:SP	-0.341	-4.618 to 3.936	No	ns	>0.9999			
10	68:LL vs. 68:SPLL	-0.2108	-4.697 to 4.275	No	ns	>0.9999			
11	68:LL vs. 89:LL	-0.6203	-4.898 to 3.657	No	ns	>0.9999			
12	68:LL vs. 89:SP	-0.3285	-4.606 to 3.949	No	ns	>0.9999			
13	68:LL vs. 89:SPLL	-3.563	-7.84 to 0.7143	No	ns	0.1718			
14	68:LL vs. 110:LL	0.141	-4.136 to 4.418	No	ns	>0.9999			
15	68:LL vs. 110:SP	-0.304	-4.581 to 3.973	No	ns	>0.9999			
16	68:LL vs. 110:SPLL	-13.05	-17.33 to -8.775	Yes	****	<0.0001			
17	68:SP vs. 68:SPLL	0.1302	-4.356 to 4.616	No	ns	>0.9999			
18	68:SP vs. 89:LL	-0.2793	-4.557 to 3.998	No	ns	>0.9999			
19	68:SP vs. 89:SP	0.01248	-4.265 to 4.29	No	ns	>0.9999			
20	68:SP vs. 89:SPLL	-3.222	-7.499 to 1.055	No	ns	0.2809			
21	68:SP vs. 110:LL	0.482	-3.795 to 4.759	No	ns	>0.9999			
22	68:SP vs. 110:SP	0.037	-4.24 to 4.314	No	ns	>0.9999			
23	68:SP vs. 110:SPLL	-12.71	-16.99 to -8.434	Yes	****	<0.0001			
24	68:SPLL vs. 89:LL	-0.4095	-4.896 to 4.077	No	ns	>0.9999			
25	68:SPLL vs. 89:SP	-0.1177	-4.604 to 4.368	No	ns	>0.9999			
26	68:SPLL vs. 89:SPLL	-3.352	-7.838 to 1.134	No	ns	0.2907			
27	68:SPLL vs. 110:LL	0.3518	-4.134 to 4.838	No	ns	>0.9999			
28	68:SPLL vs. 110:SP	-0.09318	-4.579 to 4.393	No	ns	>0.9999			
29	68:SPLL vs. 110:SPLL	-12.84	-17.33 to -8.355	Yes	****	<0.0001			
30	89:LL vs. 89:SP	0.2918	-3.986 to 4.569	No	ns	>0.9999			

**2way ANOVA** Multiple comparisons									
31	89:LL vs. 89:SPLL	-2.943	-7.22 to 1.335	No	ns	0.3973			
32	89:LL vs. 110:LL	0.7613	-3.516 to 5.039	No	ns	0.9996			
33	89:LL vs. 110:SP	0.3163	-3.961 to 4.594	No	ns	>0.9999			
34	89:LL vs. 110:SPLL	-12.43	-16.71 to -8.154	Yes	****	<0.0001			
35	89:SP vs. 89:SPLL	-3.235	-7.512 to 1.043	No	ns	0.2762			
36	89:SP vs. 110:LL	0.4695	-3.808 to 4.747	No	ns	>0.9999			
37	89:SP vs. 110:SP	0.02452	-4.253 to 4.302	No	ns	>0.9999			
38	89:SP vs. 110:SPLL	-12.72	-17 to -8.446	Yes	****	<0.0001			
39	89:SPLL vs. 110:LL	3.704	-0.5733 to 7.981	No	ns	0.1375			
40	89:SPLL vs. 110:SP	3.259	-1.018 to 7.536	No	ns	0.2672			
41	89:SPLL vs. 110:SPLL	-9.489	-13.77 to -5.212	Yes	****	<0.0001			
42	110:LL vs. 110:SP	-0.445	-4.722 to 3.832	No	ns	>0.9999			
43	110:LL vs. 110:SPLL	-13.19	-17.47 to -8.916	Yes	****	<0.0001			
44	110:SP vs. 110:SPLL	-12.75	-17.03 to -8.471	Yes	****	<0.0001			
45									
46									
47	Test details	Mean 1	Mean 2	Mean Diff.	SE of diff.	N1	N2	q	DF
48									
49	68:LL vs. 68:SP	0.7731	1.114	-0.341	1.312	6	6	0.3676	44
50	68:LL vs. 68:SPLL	0.7731	0.9839	-0.2108	1.376	6	5	0.2167	44
51	68:LL vs. 89:LL	0.7731	1.393	-0.6203	1.312	6	6	0.6688	44
52	68:LL vs. 89:SP	0.7731	1.102	-0.3285	1.312	6	6	0.3542	44
53	68:LL vs. 89:SPLL	0.7731	4.336	-3.563	1.312	6	6	3.841	44
54	68:LL vs. 110:LL	0.7731	0.6321	0.141	1.312	6	6	0.152	44
55	68:LL vs. 110:SP	0.7731	1.077	-0.304	1.312	6	6	0.3277	44
56	68:LL vs. 110:SPLL	0.7731	13.83	-13.05	1.312	6	6	14.07	44
57	68:SP vs. 68:SPLL	1.114	0.9839	0.1302	1.376	6	5	0.1338	44
58	68:SP vs. 89:LL	1.114	1.393	-0.2793	1.312	6	6	0.3011	44
59	68:SP vs. 89:SP	1.114	1.102	0.01248	1.312	6	6	0.01346	44
60	68:SP vs. 89:SPLL	1.114	4.336	-3.222	1.312	6	6	3.474	44

2way ANOVA<br>Multiple comparisons									
61	68:SP vs. 110:LL	1.114	0.6321	0.482	1.312	6	6	0.5196	44
62	68:SP vs. 110:SP	1.114	1.077	0.037	1.312	6	6	0.03989	44
63	68:SP vs. 110:SPLL	1.114	13.83	-12.71	1.312	6	6	13.7	44
64	68:SPLL vs. 89:LL	0.9839	1.393	-0.4095	1.376	5	6	0.4209	44
65	68:SPLL vs. 89:SP	0.9839	1.102	-0.1177	1.376	5	6	0.121	44
66	68:SPLL vs. 89:SPLL	0.9839	4.336	-3.352	1.376	5	6	3.446	44
67	68:SPLL vs. 110:LL	0.9839	0.6321	0.3518	1.376	5	6	0.3616	44
68	68:SPLL vs. 110:SP	0.9839	1.077	-0.09318	1.376	5	6	0.09578	44
69	68:SPLL vs. 110:SPLL	0.9839	13.83	-12.84	1.376	5	6	13.2	44
70	89:LL vs. 89:SP	1.393	1.102	0.2918	1.312	6	6	0.3146	44
71	89:LL vs. 89:SPLL	1.393	4.336	-2.943	1.312	6	6	3.173	44
72	89:LL vs. 110:LL	1.393	0.6321	0.7613	1.312	6	6	0.8208	44
73	89:LL vs. 110:SP	1.393	1.077	0.3163	1.312	6	6	0.341	44
74	89:LL vs. 110:SPLL	1.393	13.83	-12.43	1.312	6	6	13.4	44
75	89:SP vs. 89:SPLL	1.102	4.336	-3.235	1.312	6	6	3.487	44
76	89:SP vs. 110:LL	1.102	0.6321	0.4695	1.312	6	6	0.5062	44
77	89:SP vs. 110:SP	1.102	1.077	0.02452	1.312	6	6	0.02643	44
78	89:SP vs. 110:SPLL	1.102	13.83	-12.72	1.312	6	6	13.72	44
79	89:SPLL vs. 110:LL	4.336	0.6321	3.704	1.312	6	6	3.993	44
80	89:SPLL vs. 110:SP	4.336	1.077	3.259	1.312	6	6	3.514	44
81	89:SPLL vs. 110:SPLL	4.336	13.83	-9.489	1.312	6	6	10.23	44
82	110:LL vs. 110:SP	0.6321	1.077	-0.445	1.312	6	6	0.4797	44
83	110:LL vs. 110:SPLL	0.6321	13.83	-13.19	1.312	6	6	14.22	44
84	110:SP vs. 110:SPLL	1.077	13.83	-12.75	1.312	6	6	13.74	44

2way ANOVA
Tabular results

1	Table Analyzed	Cand.genes 2013 ST6GALNAC				
2						
3	Two-way ANOVA	Ordinary				
4	Alpha	0.05				
5						
6	Source of Variation	% of total variation	P value	P value summary	Significant?	
7	Interaction	21.24	<0.0001	****	Yes	
8	Time	15.81	<0.0001	****	Yes	
9	Treatment	37.79	<0.0001	****	Yes	
10						
11	ANOVA table	SS (Type III)	DF	MS	F (DFn, DFd)	P value
12	Interaction	240.3	4	60.06	F (4, 44) = 10.67	P<0.0001
13	Time	178.9	2	89.44	F (2, 44) = 15.89	P<0.0001
14	Treatment	427.4	2	213.7	F (2, 44) = 37.98	P<0.0001
15	Residual	247.6	44	5.628		
16						
17	Number of missing values	1				

2way ANOVA
Multiple comparisons

1	Compare cell means regardless of rows and columns								
2									
3	Number of families	1							
4	Number of comparisons per family	36							
5	Alpha	0.05							
6									
7	Tukey's multiple comparisons test	Mean Diff.	95.00% CI of diff.	Significant?	Summary	Adjusted P Value			
8									
9	68:LL vs. 68:SP	0.3867	-4.079 to 4.853	No	ns	>0.9999			
10	68:LL vs. 68:SPLL	0.6079	-4.076 to 5.292	No	ns	>0.9999			
11	68:LL vs. 89:LL	-2.575	-7.041 to 1.891	No	ns	0.6304			
12	68:LL vs. 89:SP	0.7663	-3.7 to 5.232	No	ns	0.9997			
13	68:LL vs. 89:SPLL	-9.304	-13.77 to -4.838	Yes	****	<0.0001			
14	68:LL vs. 110:LL	-0.7876	-5.254 to 3.679	No	ns	0.9997			
15	68:LL vs. 110:SP	0.2937	-4.172 to 4.76	No	ns	>0.9999			
16	68:LL vs. 110:SPLL	-10.06	-14.52 to -5.592	Yes	****	<0.0001			
17	68:SP vs. 68:SPLL	0.2212	-4.463 to 4.905	No	ns	>0.9999			
18	68:SP vs. 89:LL	-2.962	-7.428 to 1.505	No	ns	0.4469			
19	68:SP vs. 89:SP	0.3796	-4.087 to 4.846	No	ns	>0.9999			
20	68:SP vs. 89:SPLL	-9.691	-14.16 to -5.225	Yes	****	<0.0001			
21	68:SP vs. 110:LL	-1.174	-5.64 to 3.292	No	ns	0.9941			
22	68:SP vs. 110:SP	-0.09297	-4.559 to 4.373	No	ns	>0.9999			
23	68:SP vs. 110:SPLL	-10.44	-14.91 to -5.979	Yes	****	<0.0001			
24	68:SPLL vs. 89:LL	-3.183	-7.867 to 1.501	No	ns	0.4140			
25	68:SPLL vs. 89:SP	0.1584	-4.526 to 4.843	No	ns	>0.9999			
26	68:SPLL vs. 89:SPLL	-9.912	-14.6 to -5.228	Yes	****	<0.0001			
27	68:SPLL vs. 110:LL	-1.395	-6.08 to 3.289	No	ns	0.9866			
28	68:SPLL vs. 110:SP	-0.3141	-4.998 to 4.37	No	ns	>0.9999			
29	68:SPLL vs. 110:SPLL	-10.67	-15.35 to -5.982	Yes	****	<0.0001			
30	89:LL vs. 89:SP	3.341	-1.125 to 7.807	No	ns	0.2893			

**2way ANOVA** Multiple comparisons									
31	89:LL vs. 89:SPLL	-6.729	-11.2 to -2.263	Yes	***	0.0004			
32	89:LL vs. 110:LL	1.787	-2.679 to 6.253	No	ns	0.9246			
33	89:LL vs. 110:SP	2.869	-1.598 to 7.335	No	ns	0.4900			
34	89:LL vs. 110:SPLL	-7.483	-11.95 to -3.017	Yes	****	<0.0001			
35	89:SP vs. 89:SPLL	-10.07	-14.54 to -5.604	Yes	****	<0.0001			
36	89:SP vs. 110:LL	-1.554	-6.02 to 2.912	No	ns	0.9654			
37	89:SP vs. 110:SP	-0.4726	-4.939 to 3.994	No	ns	>0.9999			
38	89:SP vs. 110:SPLL	-10.82	-15.29 to -6.358	Yes	****	<0.0001			
39	89:SPLL vs. 110:LL	8.517	4.05 to 12.98	Yes	****	<0.0001			
40	89:SPLL vs. 110:SP	9.598	5.132 to 14.06	Yes	****	<0.0001			
41	89:SPLL vs. 110:SPLL	-0.7538	-5.22 to 3.712	No	ns	0.9997			
42	110:LL vs. 110:SP	1.081	-3.385 to 5.548	No	ns	0.9966			
43	110:LL vs. 110:SPLL	-9.27	-13.74 to -4.804	Yes	****	<0.0001			
44	110:SP vs. 110:SPLL	-10.35	-14.82 to -5.886	Yes	****	<0.0001			
45									
46									
47	Test details	Mean 1	Mean 2	Mean Diff.	SE of diff.	N1	N2	q	DF
48									
49	68:LL vs. 68:SP	1.835	1.448	0.3867	1.37	6	6	0.3993	44
50	68:LL vs. 68:SPLL	1.835	1.227	0.6079	1.437	6	5	0.5984	44
51	68:LL vs. 89:LL	1.835	4.41	-2.575	1.37	6	6	2.659	44
52	68:LL vs. 89:SP	1.835	1.069	0.7663	1.37	6	6	0.7912	44
53	68:LL vs. 89:SPLL	1.835	11.14	-9.304	1.37	6	6	9.607	44
54	68:LL vs. 110:LL	1.835	2.623	-0.7876	1.37	6	6	0.8132	44
55	68:LL vs. 110:SP	1.835	1.541	0.2937	1.37	6	6	0.3033	44
56	68:LL vs. 110:SPLL	1.835	11.89	-10.06	1.37	6	6	10.39	44
57	68:SP vs. 68:SPLL	1.448	1.227	0.2212	1.437	6	5	0.2177	44
58	68:SP vs. 89:LL	1.448	4.41	-2.962	1.37	6	6	3.058	44
59	68:SP vs. 89:SP	1.448	1.069	0.3796	1.37	6	6	0.3919	44
60	68:SP vs. 89:SPLL	1.448	11.14	-9.691	1.37	6	6	10.01	44

2way ANOVA<br>Multiple comparisons									
61	68:SP vs. 110:LL	1.448	2.623	-1.174	1.37	6	6	1.213	44
62	68:SP vs. 110:SP	1.448	1.541	-0.09297	1.37	6	6	0.09599	44
63	68:SP vs. 110:SPLL	1.448	11.89	-10.44	1.37	6	6	10.78	44
64	68:SPLL vs. 89:LL	1.227	4.41	-3.183	1.437	5	6	3.133	44
65	68:SPLL vs. 89:SP	1.227	1.069	0.1584	1.437	5	6	0.156	44
66	68:SPLL vs. 89:SPLL	1.227	11.14	-9.912	1.437	5	6	9.758	44
67	68:SPLL vs. 110:LL	1.227	2.623	-1.395	1.437	5	6	1.374	44
68	68:SPLL vs. 110:SP	1.227	1.541	-0.3141	1.437	5	6	0.3092	44
69	68:SPLL vs. 110:SPLL	1.227	11.89	-10.67	1.437	5	6	10.5	44
70	89:LL vs. 89:SP	4.41	1.069	3.341	1.37	6	6	3.45	44
71	89:LL vs. 89:SPLL	4.41	11.14	-6.729	1.37	6	6	6.948	44
72	89:LL vs. 110:LL	4.41	2.623	1.787	1.37	6	6	1.845	44
73	89:LL vs. 110:SP	4.41	1.541	2.869	1.37	6	6	2.962	44
74	89:LL vs. 110:SPLL	4.41	11.89	-7.483	1.37	6	6	7.727	44
75	89:SP vs. 89:SPLL	1.069	11.14	-10.07	1.37	6	6	10.4	44
76	89:SP vs. 110:LL	1.069	2.623	-1.554	1.37	6	6	1.604	44
77	89:SP vs. 110:SP	1.069	1.541	-0.4726	1.37	6	6	0.4879	44
78	89:SP vs. 110:SPLL	1.069	11.89	-10.82	1.37	6	6	11.18	44
79	89:SPLL vs. 110:LL	11.14	2.623	8.517	1.37	6	6	8.794	44
80	89:SPLL vs. 110:SP	11.14	1.541	9.598	1.37	6	6	9.91	44
81	89:SPLL vs. 110:SPLL	11.14	11.89	-0.7538	1.37	6	6	0.7783	44
82	110:LL vs. 110:SP	2.623	1.541	1.081	1.37	6	6	1.117	44
83	110:LL vs. 110:SPLL	2.623	11.89	-9.27	1.37	6	6	9.572	44
84	110:SP vs. 110:SPLL	1.541	11.89	-10.35	1.37	6	6	10.69	44

2way ANOVA
Tabular results

1	Table Analyzed	Cand.genes 2013 SLC5A7				
2						
3	Two-way ANOVA	Ordinary				
4	Alpha	0.05				
5						
6	Source of Variation	% of total variation	P value	P value summary	Significant?	
7	Interaction	20.11	<0.0001	****	Yes	
8	Time	16.62	<0.0001	****	Yes	
9	Treatment	34.76	<0.0001	****	Yes	
10						
11	ANOVA table	SS (Type III)	DF	MS	F (DFn, DFd)	P value
12	Interaction	211.9	4	52.98	F (4, 44) = 8.518	P<0.0001
13	Time	175.1	2	87.55	F (2, 44) = 14.07	P<0.0001
14	Treatment	366.3	2	183.2	F (2, 44) = 29.45	P<0.0001
15	Residual	273.7	44	6.22		
16						
17	Number of missing values	1				

2way ANOVA
Multiple comparisons

1	Compare cell means regardless of rows and columns								
2									
3	Number of families	1							
4	Number of comparisons per family	36							
5	Alpha	0.05							
6									
7	Tukey's multiple comparisons test	Mean Diff.	95.00% CI of diff.	Significant?	Summary	Adjusted P Value			
8									
9	68:LL vs. 68:SP	2.297	-2.398 to 6.992	No	ns	0.8022			
10	68:LL vs. 68:SPLL	1.824	-3.101 to 6.748	No	ns	0.9506			
11	68:LL vs. 89:LL	-3.105	-7.8 to 1.591	No	ns	0.4508			
12	68:LL vs. 89:SP	2.47	-2.225 to 7.165	No	ns	0.7341			
13	68:LL vs. 89:SPLL	-7.528	-12.22 to -2.833	Yes	***	0.0001			
14	68:LL vs. 110:LL	-0.04379	-4.739 to 4.651	No	ns	>0.9999			
15	68:LL vs. 110:SP	1.054	-3.642 to 5.749	No	ns	0.9980			
16	68:LL vs. 110:SPLL	-7.925	-12.62 to -3.229	Yes	****	<0.0001			
17	68:SP vs. 68:SPLL	-0.4733	-5.398 to 4.451	No	ns	>0.9999			
18	68:SP vs. 89:LL	-5.401	-10.1 to -0.7062	Yes	*	0.0137			
19	68:SP vs. 89:SP	0.1729	-4.522 to 4.868	No	ns	>0.9999			
20	68:SP vs. 89:SPLL	-9.825	-14.52 to -5.13	Yes	****	<0.0001			
21	68:SP vs. 110:LL	-2.341	-7.036 to 2.355	No	ns	0.7858			
22	68:SP vs. 110:SP	-1.243	-5.939 to 3.452	No	ns	0.9938			
23	68:SP vs. 110:SPLL	-10.22	-14.92 to -5.526	Yes	****	<0.0001			
24	68:SPLL vs. 89:LL	-4.928	-9.853 to -0.003688	Yes	*	0.0497			
25	68:SPLL vs. 89:SP	0.6463	-4.278 to 5.571	No	ns	>0.9999			
26	68:SPLL vs. 89:SPLL	-9.352	-14.28 to -4.427	Yes	****	<0.0001			
27	68:SPLL vs. 110:LL	-1.867	-6.792 to 3.057	No	ns	0.9436			
28	68:SPLL vs. 110:SP	-0.77	-5.694 to 4.154	No	ns	0.9999			
29	68:SPLL vs. 110:SPLL	-9.748	-14.67 to -4.824	Yes	****	<0.0001			
30	89:LL vs. 89:SP	5.574	0.8791 to 10.27	Yes	**	0.0097			

**2way ANOVA** Multiple comparisons									
31	89:LL vs. 89:SPLL	-4.423	-9.119 to 0.2717	No	ns	0.0789			
32	89:LL vs. 110:LL	3.061	-1.635 to 7.756	No	ns	0.4700			
33	89:LL vs. 110:SP	4.158	-0.5372 to 8.853	No	ns	0.1197			
34	89:LL vs. 110:SPLL	-4.82	-9.515 to -0.1248	Yes	*	0.0402			
35	89:SP vs. 89:SPLL	-9.998	-14.69 to -5.303	Yes	****	<0.0001			
36	89:SP vs. 110:LL	-2.514	-7.209 to 2.182	No	ns	0.7157			
37	89:SP vs. 110:SP	-1.416	-6.112 to 3.279	No	ns	0.9855			
38	89:SP vs. 110:SPLL	-10.39	-15.09 to -5.699	Yes	****	<0.0001			
39	89:SPLL vs. 110:LL	7.484	2.789 to 12.18	Yes	***	0.0002			
40	89:SPLL vs. 110:SP	8.582	3.886 to 13.28	Yes	****	<0.0001			
41	89:SPLL vs. 110:SPLL	-0.3965	-5.092 to 4.299	No	ns	>0.9999			
42	110:LL vs. 110:SP	1.097	-3.598 to 5.793	No	ns	0.9974			
43	110:LL vs. 110:SPLL	-7.881	-12.58 to -3.186	Yes	****	<0.0001			
44	110:SP vs. 110:SPLL	-8.978	-13.67 to -4.283	Yes	****	<0.0001			
45									
46									
47	Test details	Mean 1	Mean 2	Mean Diff.	SE of diff.	N1	N2	q	DF
48									
49	68:LL vs. 68:SP	3.835	1.538	2.297	1.44	6	6	2.256	44
50	68:LL vs. 68:SPLL	3.835	2.012	1.824	1.51	6	5	1.708	44
51	68:LL vs. 89:LL	3.835	6.94	-3.105	1.44	6	6	3.049	44
52	68:LL vs. 89:SP	3.835	1.365	2.47	1.44	6	6	2.426	44
53	68:LL vs. 89:SPLL	3.835	11.36	-7.528	1.44	6	6	7.394	44
54	68:LL vs. 110:LL	3.835	3.879	-0.04379	1.44	6	6	0.043	44
55	68:LL vs. 110:SP	3.835	2.782	1.054	1.44	6	6	1.035	44
56	68:LL vs. 110:SPLL	3.835	11.76	-7.925	1.44	6	6	7.783	44
57	68:SP vs. 68:SPLL	1.538	2.012	-0.4733	1.51	6	5	0.4433	44
58	68:SP vs. 89:LL	1.538	6.94	-5.401	1.44	6	6	5.305	44
59	68:SP vs. 89:SP	1.538	1.365	0.1729	1.44	6	6	0.1698	44
60	68:SP vs. 89:SPLL	1.538	11.36	-9.825	1.44	6	6	9.649	44

2way ANOVA<br>Multiple comparisons									
61	68:SP vs. 110:LL	1.538	3.879	-2.341	1.44	6	6	2.299	44
62	68:SP vs. 110:SP	1.538	2.782	-1.243	1.44	6	6	1.221	44
63	68:SP vs. 110:SPLL	1.538	11.76	-10.22	1.44	6	6	10.04	44
64	68:SPLL vs. 89:LL	2.012	6.94	-4.928	1.51	5	6	4.615	44
65	68:SPLL vs. 89:SP	2.012	1.365	0.6463	1.51	5	6	0.6052	44
66	68:SPLL vs. 89:SPLL	2.012	11.36	-9.352	1.51	5	6	8.757	44
67	68:SPLL vs. 110:LL	2.012	3.879	-1.867	1.51	5	6	1.749	44
68	68:SPLL vs. 110:SP	2.012	2.782	-0.77	1.51	5	6	0.7211	44
69	68:SPLL vs. 110:SPLL	2.012	11.76	-9.748	1.51	5	6	9.128	44
70	89:LL vs. 89:SP	6.94	1.365	5.574	1.44	6	6	5.475	44
71	89:LL vs. 89:SPLL	6.94	11.36	-4.423	1.44	6	6	4.345	44
72	89:LL vs. 110:LL	6.94	3.879	3.061	1.44	6	6	3.006	44
73	89:LL vs. 110:SP	6.94	2.782	4.158	1.44	6	6	4.084	44
74	89:LL vs. 110:SPLL	6.94	11.76	-4.82	1.44	6	6	4.734	44
75	89:SP vs. 89:SPLL	1.365	11.36	-9.998	1.44	6	6	9.819	44
76	89:SP vs. 110:LL	1.365	3.879	-2.514	1.44	6	6	2.469	44
77	89:SP vs. 110:SP	1.365	2.782	-1.416	1.44	6	6	1.391	44
78	89:SP vs. 110:SPLL	1.365	11.76	-10.39	1.44	6	6	10.21	44
79	89:SPLL vs. 110:LL	11.36	3.879	7.484	1.44	6	6	7.351	44
80	89:SPLL vs. 110:SP	11.36	2.782	8.582	1.44	6	6	8.428	44
81	89:SPLL vs. 110:SPLL	11.36	11.76	-0.3965	1.44	6	6	0.3894	44
82	110:LL vs. 110:SP	3.879	2.782	1.097	1.44	6	6	1.078	44
83	110:LL vs. 110:SPLL	3.879	11.76	-7.881	1.44	6	6	7.74	44
84	110:SP vs. 110:SPLL	2.782	11.76	-8.978	1.44	6	6	8.818	44

2way ANOVA
Tabular results

1	Table Analyzed	Cand.genes 2013 S100A				
2						
3	Two-way ANOVA	Ordinary				
4	Alpha	0.05				
5						
6	Source of Variation	% of total variation	P value	P value summary	Significant?	
7	Interaction	24.27	<0.0001	****	Yes	
8	Time	13.88	<0.0001	****	Yes	
9	Treatment	33.5	<0.0001	****	Yes	
10						
11	ANOVA table	SS (Type III)	DF	MS	F (DFn, DFd)	P value
12	Interaction	43130	4	10783	F (4, 44) = 10.41	P<0.0001
13	Time	24674	2	12337	F (2, 44) = 11.91	P<0.0001
14	Treatment	59545	2	29773	F (2, 44) = 28.74	P<0.0001
15	Residual	45582	44	1036		
16						
17	Number of missing values	1				

2way ANOVA
Multiple comparisons

1	Compare cell means regardless of rows and columns					
2						
3	Number of families	1				
4	Number of comparisons per family	36				
5	Alpha	0.05				
6						
7	Tukey's multiple comparisons test	Mean Diff.	95.00% CI of diff.	Significant?	Summary	Adjusted P Value
8						
9	68:LL vs. 68:SP	21.47	-39.12 to 82.06	No	ns	0.9615
10	68:LL vs. 68:SPLL	20.8	-42.75 to 84.35	No	ns	0.9760
11	68:LL vs. 89:LL	-32.9	-93.5 to 27.69	No	ns	0.7003
12	68:LL vs. 89:SP	22.53	-38.07 to 83.12	No	ns	0.9494
13	68:LL vs. 89:SPLL	-78.36	-139 to -17.77	Yes	**	0.0035
14	68:LL vs. 110:LL	2.995	-57.6 to 63.59	No	ns	>0.9999
15	68:LL vs. 110:SP	19.03	-41.56 to 79.63	No	ns	0.9813
16	68:LL vs. 110:SPLL	-125.6	-186.2 to -64.98	Yes	****	<0.0001
17	68:SP vs. 68:SPLL	-0.6735	-64.22 to 62.88	No	ns	>0.9999
18	68:SP vs. 89:LL	-54.38	-115 to 6.219	No	ns	0.1100
19	68:SP vs. 89:SP	1.057	-59.54 to 61.65	No	ns	>0.9999
20	68:SP vs. 89:SPLL	-99.83	-160.4 to -39.24	Yes	****	<0.0001
21	68:SP vs. 110:LL	-18.48	-79.07 to 42.12	No	ns	0.9845
22	68:SP vs. 110:SP	-2.438	-63.03 to 58.16	No	ns	>0.9999
23	68:SP vs. 110:SPLL	-147	-207.6 to -86.45	Yes	****	<0.0001
24	68:SPLL vs. 89:LL	-53.7	-117.3 to 9.849	No	ns	0.1586
25	68:SPLL vs. 89:SP	1.73	-61.82 to 65.28	No	ns	>0.9999
26	68:SPLL vs. 89:SPLL	-99.16	-162.7 to -35.61	Yes	***	0.0002
27	68:SPLL vs. 110:LL	-17.8	-81.35 to 45.75	No	ns	0.9910
28	68:SPLL vs. 110:SP	-1.765	-65.32 to 61.79	No	ns	>0.9999
29	68:SPLL vs. 110:SPLL	-146.4	-209.9 to -82.82	Yes	****	<0.0001
30	89:LL vs. 89:SP	55.43	-5.162 to 116	No	ns	0.0968

2way ANOVA
Multiple comparisons

31	89:LL vs. 89:SPLL	-45.46	-106 to 15.14	No	ns	0.2859			
32	89:LL vs. 110:LL	35.9	-24.7 to 96.49	No	ns	0.5964			
33	89:LL vs. 110:SP	51.94	-8.657 to 112.5	No	ns	0.1461			
34	89:LL vs. 110:SPLL	-92.67	-153.3 to -32.07	Yes	***	0.0003			
35	89:SP vs. 89:SPLL	-100.9	-161.5 to -40.29	Yes	****	<0.0001			
36	89:SP vs. 110:LL	-19.53	-80.13 to 41.06	No	ns	0.9781			
37	89:SP vs. 110:SP	-3.495	-64.09 to 57.1	No	ns	>0.9999			
38	89:SP vs. 110:SPLL	-148.1	-208.7 to -87.5	Yes	****	<0.0001			
39	89:SPLL vs. 110:LL	81.35	20.76 to 141.9	Yes	**	0.0022			
40	89:SPLL vs. 110:SP	97.39	36.8 to 158	Yes	***	0.0001			
41	89:SPLL vs. 110:SPLL	-47.21	-107.8 to 13.38	No	ns	0.2414			
42	110:LL vs. 110:SP	16.04	-44.55 to 76.63	No	ns	0.9938			
43	110:LL vs. 110:SPLL	-128.6	-189.2 to -67.97	Yes	****	<0.0001			
44	110:SP vs. 110:SPLL	-144.6	-205.2 to -84.01	Yes	****	<0.0001			
45									
46									
47	Test details	Mean 1	Mean 2	Mean Diff.	SE of diff.	N1	N2	q	DF
48									
49	68:LL vs. 68:SP	31.75	10.28	21.47	18.58	6	6	1.634	44
50	68:LL vs. 68:SPLL	31.75	10.95	20.8	19.49	6	5	1.509	44
51	68:LL vs. 89:LL	31.75	64.66	-32.9	18.58	6	6	2.504	44
52	68:LL vs. 89:SP	31.75	9.224	22.53	18.58	6	6	1.714	44
53	68:LL vs. 89:SPLL	31.75	110.1	-78.36	18.58	6	6	5.963	44
54	68:LL vs. 110:LL	31.75	28.76	2.995	18.58	6	6	0.2279	44
55	68:LL vs. 110:SP	31.75	12.72	19.03	18.58	6	6	1.449	44
56	68:LL vs. 110:SPLL	31.75	157.3	-125.6	18.58	6	6	9.556	44
57	68:SP vs. 68:SPLL	10.28	10.95	-0.6735	19.49	6	5	0.04887	44
58	68:SP vs. 89:LL	10.28	64.66	-54.38	18.58	6	6	4.138	44
59	68:SP vs. 89:SP	10.28	9.224	1.057	18.58	6	6	0.08043	44
60	68:SP vs. 89:SPLL	10.28	110.1	-99.83	18.58	6	6	7.598	44

**2way ANOVA**<br>Multiple comparisons									
**61**	68:SP vs. 110:LL	10.28	28.76	-18.48	18.58	6	6	1.406	44
**62**	68:SP vs. 110:SP	10.28	12.72	-2.438	18.58	6	6	0.1855	44
**63**	68:SP vs. 110:SPLL	10.28	157.3	-147	18.58	6	6	11.19	44
**64**	68:SPLL vs. 89:LL	10.95	64.66	-53.7	19.49	5	6	3.897	44
**65**	68:SPLL vs. 89:SP	10.95	9.224	1.73	19.49	5	6	0.1256	44
**66**	68:SPLL vs. 89:SPLL	10.95	110.1	-99.16	19.49	5	6	7.195	44
**67**	68:SPLL vs. 110:LL	10.95	28.76	-17.8	19.49	5	6	1.292	44
**68**	68:SPLL vs. 110:SP	10.95	12.72	-1.765	19.49	5	6	0.128	44
**69**	68:SPLL vs. 110:SPLL	10.95	157.3	-146.4	19.49	5	6	10.62	44
**70**	89:LL vs. 89:SP	64.66	9.224	55.43	18.58	6	6	4.219	44
**71**	89:LL vs. 89:SPLL	64.66	110.1	-45.46	18.58	6	6	3.459	44
**72**	89:LL vs. 110:LL	64.66	28.76	35.9	18.58	6	6	2.732	44
**73**	89:LL vs. 110:SP	64.66	12.72	51.94	18.58	6	6	3.953	44
**74**	89:LL vs. 110:SPLL	64.66	157.3	-92.67	18.58	6	6	7.052	44
**75**	89:SP vs. 89:SPLL	9.224	110.1	-100.9	18.58	6	6	7.678	44
**76**	89:SP vs. 110:LL	9.224	28.76	-19.53	18.58	6	6	1.487	44
**77**	89:SP vs. 110:SP	9.224	12.72	-3.495	18.58	6	6	0.266	44
**78**	89:SP vs. 110:SPLL	9.224	157.3	-148.1	18.58	6	6	11.27	44
**79**	89:SPLL vs. 110:LL	110.1	28.76	81.35	18.58	6	6	6.191	44
**80**	89:SPLL vs. 110:SP	110.1	12.72	97.39	18.58	6	6	7.412	44
**81**	89:SPLL vs. 110:SPLL	110.1	157.3	-47.21	18.58	6	6	3.593	44
**82**	110:LL vs. 110:SP	28.76	12.72	16.04	18.58	6	6	1.221	44
**83**	110:LL vs. 110:SPLL	28.76	157.3	-128.6	18.58	6	6	9.784	44
**84**	110:SP vs. 110:SPLL	12.72	157.3	-144.6	18.58	6	6	11	44
