# Supplementary material for: RNA profiling identifies novel, photoperiod-history dependent markers associated with enhanced saltwater performance in juvenile Atlantic salmon
Source: PLoS One. 2020 Apr 8;15(4):e0227496. doi: 10.1371/journal.pone.0227496 (PMC7141700; doi:10.1371/journal.pone.0227496)
Supplement: S7 Table — Tables showing the 2-way ANOVA and multiple comparison results for the expression of the genes measured in experiment 2 and shown in Fig 4. (PDF) [file pone.0227496.s008.pdf]

2way ANOVA
Tabular results

1	Table Analyzed	CFTR I 2-ANOVA				
2						
3	Two-way ANOVA	Ordinary				
4	Alpha	0.05				
5						
6	Source of Variation	% of total variation	P value	P value summary	Significant?	
7	Interaction	10.17	<0.0001	****	Yes	
8	Time	62.43	<0.0001	****	Yes	
9	Treatment	9.897	<0.0001	****	Yes	
10						
11	ANOVA table	SS	DF	MS	F (DFn, DFd)	P value
12	Interaction	3.461	8	0.4326	F (8, 75) = 5.446	P<0.0001
13	Time	21.25	4	5.312	F (4, 75) = 66.87	P<0.0001
14	Treatment	3.368	2	1.684	F (2, 75) = 21.2	P<0.0001
15	Residual	5.958	75	0.07944		
16						
17	Number of missing values	0				

2way ANOVA
Multiple comparisons

1	Compare cell means regardless of rows and columns						
2							
3	Number of families	1					
4	Number of comparisons per family	105					
5	Alpha	0.05					
6							
7	Tukey's multiple comparisons test	Mean Diff.	95.00% CI of diff.	Significant?	Summary	Adjusted P Value	
8							
9	End of winter:2 week winter vs. End of winter:4 week winter	0.1092	-0.4615 to 0.6799	No	ns	>0.9999	
10	End of winter:2 week winter vs. End of winter:8 week winter	0.2742	-0.2965 to 0.8449	No	ns	0.9312	
11	End of winter:2 week winter vs. 4 weeks post winter:2 week winter	-0.3932	-0.9639 to 0.1774	No	ns	0.5110	
12	End of winter:2 week winter vs. 4 weeks post winter:4 week winter	-0.7041	-1.275 to -0.1334	Yes	**	0.0039	
13	End of winter:2 week winter vs. 4 weeks post winter:8 week winter	-0.9697	-1.54 to -0.399	Yes	****	<0.0001	
14	End of winter:2 week winter vs. 8 weeks post winter:2 week winter	-0.4283	-0.999 to 0.1423	No	ns	0.3664	
15	End of winter:2 week winter vs. 8 weeks post winter:4 week winter	-0.8716	-1.442 to -0.3009	Yes	****	<0.0001	
16	End of winter:2 week winter vs. 8 weeks post winter:8 week winter	-1.595	-2.166 to -1.024	Yes	****	<0.0001	
17	End of winter:2 week winter vs. SPC -4 weeks post winter:2 week winter	0.3781	-0.1926 to 0.9488	No	ns	0.5769	
18	End of winter:2 week winter vs. SPC -4 weeks post winter:4 week winter	0.2742	-0.2965 to 0.8449	No	ns	0.9312	
19	End of winter:2 week winter vs. SPC -4 weeks post winter:8 week winter	0.1176	-0.4531 to 0.6882	No	ns	>0.9999	
20	End of winter:2 week winter vs. SPC -8 weeks post winter:2 week winter	0.2942	-0.2765 to 0.8649	No	ns	0.8875	
21	End of winter:2 week winter vs. SPC -8 weeks post winter:4 week winter	0.1176	-0.4531 to 0.6882	No	ns	>0.9999	
22	End of winter:2 week winter vs. SPC -8 weeks post winter:8 week winter	-0.328	-0.8987 to 0.2426	No	ns	0.7825	
23	End of winter:4 week winter vs. End of winter:8 week winter	0.165	-0.4057 to 0.7357	No	ns	0.9994	
24	End of winter:4 week winter vs. 4 weeks post winter:2 week winter	-0.5024	-1.073 to 0.06826	No	ns	0.1459	
25	End of winter:4 week winter vs. 4 weeks post winter:4 week winter	-0.8133	-1.384 to -0.2426	Yes	***	0.0003	
26	End of winter:4 week winter vs. 4 weeks post winter:8 week winter	-1.079	-1.65 to -0.5082	Yes	****	<0.0001	
27	End of winter:4 week winter vs. 8 weeks post winter:2 week winter	-0.5375	-1.108 to 0.03317	No	ns	0.0862	
28	End of winter:4 week winter vs. 8 weeks post winter:4 week winter	-0.9808	-1.551 to -0.4101	Yes	****	<0.0001	
29	End of winter:4 week winter vs. 8 weeks post winter:8 week winter	-1.704	-2.275 to -1.133	Yes	****	<0.0001	
30	End of winter:4 week winter vs. SPC -4 weeks post winter:2 week winter	0.2689	-0.3018 to 0.8396	No	ns	0.9403	

2way ANOVA<br>Multiple comparisons							
31	End of winter:4 week winter vs. SPC -4 weeks post winter:4 week winter	0.165	-0.4057 to 0.7357	No	ns	0.9994	
32	End of winter:4 week winter vs. SPC -4 weeks post winter:8 week winter	0.008374	-0.5623 to 0.5791	No	ns	>0.9999	
33	End of winter:4 week winter vs. SPC -8 weeks post winter:2 week winter	0.185	-0.3857 to 0.7557	No	ns	0.9980	
34	End of winter:4 week winter vs. SPC -8 weeks post winter:4 week winter	0.008374	-0.5623 to 0.5791	No	ns	>0.9999	
35	End of winter:4 week winter vs. SPC -8 weeks post winter:8 week winter	-0.4372	-1.008 to 0.1335	No	ns	0.3331	
36	End of winter:8 week winter vs. 4 weeks post winter:2 week winter	-0.6674	-1.238 to -0.09674	Yes	**	0.0082	
37	End of winter:8 week winter vs. 4 weeks post winter:4 week winter	-0.9783	-1.549 to -0.4076	Yes	****	<0.0001	
38	End of winter:8 week winter vs. 4 weeks post winter:8 week winter	-1.244	-1.815 to -0.6732	Yes	****	<0.0001	
39	End of winter:8 week winter vs. 8 weeks post winter:2 week winter	-0.7025	-1.273 to -0.1318	Yes	**	0.0040	
40	End of winter:8 week winter vs. 8 weeks post winter:4 week winter	-1.146	-1.716 to -0.5751	Yes	****	<0.0001	
41	End of winter:8 week winter vs. 8 weeks post winter:8 week winter	-1.869	-2.44 to -1.298	Yes	****	<0.0001	
42	End of winter:8 week winter vs. SPC -4 weeks post winter:2 week winter	0.1039	-0.4667 to 0.6746	No	ns	>0.9999	
43	End of winter:8 week winter vs. SPC -4 weeks post winter:4 week winter	0	-0.5707 to 0.5707	No	ns	>0.9999	
44	End of winter:8 week winter vs. SPC -4 weeks post winter:8 week winter	-0.1566	-0.7273 to 0.4141	No	ns	0.9997	
45	End of winter:8 week winter vs. SPC -8 weeks post winter:2 week winter	0.02	-0.5507 to 0.5907	No	ns	>0.9999	
46	End of winter:8 week winter vs. SPC -8 weeks post winter:4 week winter	-0.1566	-0.7273 to 0.4141	No	ns	0.9997	
47	End of winter:8 week winter vs. SPC -8 weeks post winter:8 week winter	-0.6022	-1.173 to -0.03153	Yes	*	0.0287	
48	4 weeks post winter:2 week winter vs. 4 weeks post winter:4 week winter	-0.3108	-0.8815 to 0.2599	No	ns	0.8405	
49	4 weeks post winter:2 week winter vs. 4 weeks post winter:8 week winter	-0.5765	-1.147 to -0.005775	Yes	*	0.0453	
50	4 weeks post winter:2 week winter vs. 8 weeks post winter:2 week winter	-0.03509	-0.6058 to 0.5356	No	ns	>0.9999	
51	4 weeks post winter:2 week winter vs. 8 weeks post winter:4 week winter	-0.4784	-1.049 to 0.09231	No	ns	0.2028	
52	4 weeks post winter:2 week winter vs. 8 weeks post winter:8 week winter	-1.202	-1.772 to -0.6311	Yes	****	<0.0001	
53	4 weeks post winter:2 week winter vs. SPC -4 weeks post winter:2 week winter	0.7714	0.2007 to 1.342	Yes	***	0.0009	
54	4 weeks post winter:2 week winter vs. SPC -4 weeks post winter:4 week winter	0.6674	0.09674 to 1.238	Yes	**	0.0082	
55	4 weeks post winter:2 week winter vs. SPC -4 weeks post winter:8 week winter	0.5108	-0.05988 to 1.081	No	ns	0.1293	
56	4 weeks post winter:2 week winter vs. SPC -8 weeks post winter:2 week winter	0.6874	0.1167 to 1.258	Yes	**	0.0055	
57	4 weeks post winter:2 week winter vs. SPC -8 weeks post winter:4 week winter	0.5108	-0.05988 to 1.081	No	ns	0.1293	
58	4 weeks post winter:2 week winter vs. SPC -8 weeks post winter:8 week winter	0.0652	-0.5055 to 0.6359	No	ns	>0.9999	
59	4 weeks post winter:4 week winter vs. 4 weeks post winter:8 week winter	-0.2656	-0.8363 to 0.3051	No	ns	0.9457	
60	4 weeks post winter:4 week winter vs. 8 weeks post winter:2 week winter	0.2757	-0.2949 to 0.8464	No	ns	0.9282	

2way ANOVA<br>Multiple comparisons							
61	4 weeks post winter:4 week winter vs. 8 weeks post winter:4 week winter	-0.1675	-0.7382 to 0.4031	No	ns	0.9993	
62	4 weeks post winter:4 week winter vs. 8 weeks post winter:8 week winter	-0.8909	-1.462 to -0.3202	Yes	****	<0.0001	
63	4 weeks post winter:4 week winter vs. SPC -4 weeks post winter:2 week winter	1.082	0.5115 to 1.653	Yes	****	<0.0001	
64	4 weeks post winter:4 week winter vs. SPC -4 weeks post winter:4 week winter	0.9783	0.4076 to 1.549	Yes	****	<0.0001	
65	4 weeks post winter:4 week winter vs. SPC -4 weeks post winter:8 week winter	0.8216	0.2509 to 1.392	Yes	***	0.0003	
66	4 weeks post winter:4 week winter vs. SPC -8 weeks post winter:2 week winter	0.9983	0.4276 to 1.569	Yes	****	<0.0001	
67	4 weeks post winter:4 week winter vs. SPC -8 weeks post winter:4 week winter	0.8216	0.2509 to 1.392	Yes	***	0.0003	
68	4 weeks post winter:4 week winter vs. SPC -8 weeks post winter:8 week winter	0.376	-0.1947 to 0.9467	No	ns	0.5860	
69	4 weeks post winter:8 week winter vs. 8 weeks post winter:2 week winter	0.5414	-0.02931 to 1.112	No	ns	0.0811	
70	4 weeks post winter:8 week winter vs. 8 weeks post winter:4 week winter	0.09809	-0.4726 to 0.6688	No	ns	>0.9999	
71	4 weeks post winter:8 week winter vs. 8 weeks post winter:8 week winter	-0.6253	-1.196 to -0.0546	Yes	*	0.0187	
72	4 weeks post winter:8 week winter vs. SPC -4 weeks post winter:2 week winter	1.348	0.7771 to 1.919	Yes	****	<0.0001	
73	4 weeks post winter:8 week winter vs. SPC -4 weeks post winter:4 week winter	1.244	0.6732 to 1.815	Yes	****	<0.0001	
74	4 weeks post winter:8 week winter vs. SPC -4 weeks post winter:8 week winter	1.087	0.5166 to 1.658	Yes	****	<0.0001	
75	4 weeks post winter:8 week winter vs. SPC -8 weeks post winter:2 week winter	1.264	0.6932 to 1.835	Yes	****	<0.0001	
76	4 weeks post winter:8 week winter vs. SPC -8 weeks post winter:4 week winter	1.087	0.5166 to 1.658	Yes	****	<0.0001	
77	4 weeks post winter:8 week winter vs. SPC -8 weeks post winter:8 week winter	0.6417	0.07098 to 1.212	Yes	*	0.0137	
78	8 weeks post winter:2 week winter vs. 8 weeks post winter:4 week winter	-0.4433	-1.014 to 0.1274	No	ns	0.3113	
79	8 weeks post winter:2 week winter vs. 8 weeks post winter:8 week winter	-1.167	-1.737 to -0.596	Yes	****	<0.0001	
80	8 weeks post winter:2 week winter vs. SPC -4 weeks post winter:2 week winter	0.8065	0.2358 to 1.377	Yes	***	0.0004	
81	8 weeks post winter:2 week winter vs. SPC -4 weeks post winter:4 week winter	0.7025	0.1318 to 1.273	Yes	**	0.0040	
82	8 weeks post winter:2 week winter vs. SPC -4 weeks post winter:8 week winter	0.5459	-0.02479 to 1.117	No	ns	0.0754	
83	8 weeks post winter:2 week winter vs. SPC -8 weeks post winter:2 week winter	0.7225	0.1518 to 1.293	Yes	**	0.0026	
84	8 weeks post winter:2 week winter vs. SPC -8 weeks post winter:4 week winter	0.5459	-0.02479 to 1.117	No	ns	0.0754	
85	8 weeks post winter:2 week winter vs. SPC -8 weeks post winter:8 week winter	0.1003	-0.4704 to 0.671	No	ns	>0.9999	
86	8 weeks post winter:4 week winter vs. 8 weeks post winter:8 week winter	-0.7234	-1.294 to -0.1527	Yes	**	0.0026	
87	8 weeks post winter:4 week winter vs. SPC -4 weeks post winter:2 week winter	1.25	0.679 to 1.82	Yes	****	<0.0001	
88	8 weeks post winter:4 week winter vs. SPC -4 weeks post winter:4 week winter	1.146	0.5751 to 1.716	Yes	****	<0.0001	
89	8 weeks post winter:4 week winter vs. SPC -4 weeks post winter:8 week winter	0.9892	0.4185 to 1.56	Yes	****	<0.0001	
90	8 weeks post winter:4 week winter vs. SPC -8 weeks post winter:2 week winter	1.166	0.5951 to 1.736	Yes	****	<0.0001	

	**2way ANOVA**<br>Multiple comparisons						
91	8 weeks post winter:4 week winter vs. SPC -8 weeks post winter:4 week winter	0.9892	0.4185 to 1.56	Yes	****	<0.0001	
92	8 weeks post winter:4 week winter vs. SPC -8 weeks post winter:8 week winter	0.5436	-0.02711 to 1.114	No	ns	0.0783	
93	8 weeks post winter:8 week winter vs. SPC -4 weeks post winter:2 week winter	1.973	1.402 to 2.544	Yes	****	<0.0001	
94	8 weeks post winter:8 week winter vs. SPC -4 weeks post winter:4 week winter	1.869	1.298 to 2.44	Yes	****	<0.0001	
95	8 weeks post winter:8 week winter vs. SPC -4 weeks post winter:8 week winter	1.713	1.142 to 2.283	Yes	****	<0.0001	
96	8 weeks post winter:8 week winter vs. SPC -8 weeks post winter:2 week winter	1.889	1.318 to 2.46	Yes	****	<0.0001	
97	8 weeks post winter:8 week winter vs. SPC -8 weeks post winter:4 week winter	1.713	1.142 to 2.283	Yes	****	<0.0001	
98	8 weeks post winter:8 week winter vs. SPC -8 weeks post winter:8 week winter	1.267	0.6963 to 1.838	Yes	****	<0.0001	
99	SPC -4 weeks post winter:2 week winter vs. SPC -4 weeks post winter:4 week winter	-0.1039	-0.6746 to 0.4667	No	ns	>0.9999	
100	SPC -4 weeks post winter:2 week winter vs. SPC -4 weeks post winter:8 week winter	-0.2606	-0.8312 to 0.3101	No	ns	0.9532	
101	SPC -4 weeks post winter:2 week winter vs. SPC -8 weeks post winter:2 week winter	-0.08393	-0.6546 to 0.4868	No	ns	>0.9999	
102	SPC -4 weeks post winter:2 week winter vs. SPC -8 weeks post winter:4 week winter	-0.2606	-0.8312 to 0.3101	No	ns	0.9532	
103	SPC -4 weeks post winter:2 week winter vs. SPC -8 weeks post winter:8 week winter	-0.7062	-1.277 to -0.1355	Yes	**	0.0037	
104	SPC -4 weeks post winter:4 week winter vs. SPC -4 weeks post winter:8 week winter	-0.1566	-0.7273 to 0.4141	No	ns	0.9997	
105	SPC -4 weeks post winter:4 week winter vs. SPC -8 weeks post winter:2 week winter	0.02	-0.5507 to 0.5907	No	ns	>0.9999	
106	SPC -4 weeks post winter:4 week winter vs. SPC -8 weeks post winter:4 week winter	-0.1566	-0.7273 to 0.4141	No	ns	0.9997	
107	SPC -4 weeks post winter:4 week winter vs. SPC -8 weeks post winter:8 week winter	-0.6022	-1.173 to -0.03153	Yes	*	0.0287	
108	SPC -4 weeks post winter:8 week winter vs. SPC -8 weeks post winter:2 week winter	0.1766	-0.3941 to 0.7473	No	ns	0.9988	
109	SPC -4 weeks post winter:8 week winter vs. SPC -8 weeks post winter:4 week winter	0	-0.5707 to 0.5707	No	ns	>0.9999	
110	SPC -4 weeks post winter:8 week winter vs. SPC -8 weeks post winter:8 week winter	-0.4456	-1.016 to 0.1251	No	ns	0.3033	
111	SPC -8 weeks post winter:2 week winter vs. SPC -8 weeks post winter:4 week winter	-0.1766	-0.7473 to 0.3941	No	ns	0.9988	
112	SPC -8 weeks post winter:2 week winter vs. SPC -8 weeks post winter:8 week winter	-0.6222	-1.193 to -0.05154	Yes	*	0.0198	
113	SPC -8 weeks post winter:4 week winter vs. SPC -8 weeks post winter:8 week winter	-0.4456	-1.016 to 0.1251	No	ns	0.3033	
114							
115							
116	Test details	Mean 1	Mean 2	Mean Diff.	SE of diff.	N1	N2
117							
118	End of winter:2 week winter vs. End of winter:4 week winter	0.6194	0.5102	0.1092	0.1627	6	6
119	End of winter:2 week winter vs. End of winter:8 week winter	0.6194	0.3452	0.2742	0.1627	6	6
120	End of winter:2 week winter vs. 4 weeks post winter:2 week winter	0.6194	1.013	-0.3932	0.1627	6	6

2way ANOVA<br>Multiple comparisons							
121	End of winter:2 week winter vs. 4 weeks post winter:4 week winter	0.6194	1.323	-0.7041	0.1627	6	6
122	End of winter:2 week winter vs. 4 weeks post winter:8 week winter	0.6194	1.589	-0.9697	0.1627	6	6
123	End of winter:2 week winter vs. 8 weeks post winter:2 week winter	0.6194	1.048	-0.4283	0.1627	6	6
124	End of winter:2 week winter vs. 8 weeks post winter:4 week winter	0.6194	1.491	-0.8716	0.1627	6	6
125	End of winter:2 week winter vs. 8 weeks post winter:8 week winter	0.6194	2.214	-1.595	0.1627	6	6
126	End of winter:2 week winter vs. SPC -4 weeks post winter:2 week winter	0.6194	0.2413	0.3781	0.1627	6	6
127	End of winter:2 week winter vs. SPC -4 weeks post winter:4 week winter	0.6194	0.3452	0.2742	0.1627	6	6
128	End of winter:2 week winter vs. SPC -4 weeks post winter:8 week winter	0.6194	0.5018	0.1176	0.1627	6	6
129	End of winter:2 week winter vs. SPC -8 weeks post winter:2 week winter	0.6194	0.3252	0.2942	0.1627	6	6
130	End of winter:2 week winter vs. SPC -8 weeks post winter:4 week winter	0.6194	0.5018	0.1176	0.1627	6	6
131	End of winter:2 week winter vs. SPC -8 weeks post winter:8 week winter	0.6194	0.9474	-0.328	0.1627	6	6
132	End of winter:4 week winter vs. End of winter:8 week winter	0.5102	0.3452	0.165	0.1627	6	6
133	End of winter:4 week winter vs. 4 weeks post winter:2 week winter	0.5102	1.013	-0.5024	0.1627	6	6
134	End of winter:4 week winter vs. 4 weeks post winter:4 week winter	0.5102	1.323	-0.8133	0.1627	6	6
135	End of winter:4 week winter vs. 4 weeks post winter:8 week winter	0.5102	1.589	-1.079	0.1627	6	6
136	End of winter:4 week winter vs. 8 weeks post winter:2 week winter	0.5102	1.048	-0.5375	0.1627	6	6
137	End of winter:4 week winter vs. 8 weeks post winter:4 week winter	0.5102	1.491	-0.9808	0.1627	6	6
138	End of winter:4 week winter vs. 8 weeks post winter:8 week winter	0.5102	2.214	-1.704	0.1627	6	6
139	End of winter:4 week winter vs. SPC -4 weeks post winter:2 week winter	0.5102	0.2413	0.2689	0.1627	6	6
140	End of winter:4 week winter vs. SPC -4 weeks post winter:4 week winter	0.5102	0.3452	0.165	0.1627	6	6
141	End of winter:4 week winter vs. SPC -4 weeks post winter:8 week winter	0.5102	0.5018	0.008374	0.1627	6	6
142	End of winter:4 week winter vs. SPC -8 weeks post winter:2 week winter	0.5102	0.3252	0.185	0.1627	6	6
143	End of winter:4 week winter vs. SPC -8 weeks post winter:4 week winter	0.5102	0.5018	0.008374	0.1627	6	6
144	End of winter:4 week winter vs. SPC -8 weeks post winter:8 week winter	0.5102	0.9474	-0.4372	0.1627	6	6
145	End of winter:8 week winter vs. 4 weeks post winter:2 week winter	0.3452	1.013	-0.6674	0.1627	6	6
146	End of winter:8 week winter vs. 4 weeks post winter:4 week winter	0.3452	1.323	-0.9783	0.1627	6	6
147	End of winter:8 week winter vs. 4 weeks post winter:8 week winter	0.3452	1.589	-1.244	0.1627	6	6
148	End of winter:8 week winter vs. 8 weeks post winter:2 week winter	0.3452	1.048	-0.7025	0.1627	6	6
149	End of winter:8 week winter vs. 8 weeks post winter:4 week winter	0.3452	1.491	-1.146	0.1627	6	6
150	End of winter:8 week winter vs. 8 weeks post winter:8 week winter	0.3452	2.214	-1.869	0.1627	6	6

2way ANOVA<br>Multiple comparisons							
151	End of winter:8 week winter vs. SPC -4 weeks post winter:2 week winter	0.3452	0.2413	0.1039	0.1627	6	6
152	End of winter:8 week winter vs. SPC -4 weeks post winter:4 week winter	0.3452	0.3452	0	0.1627	6	6
153	End of winter:8 week winter vs. SPC -4 weeks post winter:8 week winter	0.3452	0.5018	-0.1566	0.1627	6	6
154	End of winter:8 week winter vs. SPC -8 weeks post winter:2 week winter	0.3452	0.3252	0.02	0.1627	6	6
155	End of winter:8 week winter vs. SPC -8 weeks post winter:4 week winter	0.3452	0.5018	-0.1566	0.1627	6	6
156	End of winter:8 week winter vs. SPC -8 weeks post winter:8 week winter	0.3452	0.9474	-0.6022	0.1627	6	6
157	4 weeks post winter:2 week winter vs. 4 weeks post winter:4 week winter	1.013	1.323	-0.3108	0.1627	6	6
158	4 weeks post winter:2 week winter vs. 4 weeks post winter:8 week winter	1.013	1.589	-0.5765	0.1627	6	6
159	4 weeks post winter:2 week winter vs. 8 weeks post winter:2 week winter	1.013	1.048	-0.03509	0.1627	6	6
160	4 weeks post winter:2 week winter vs. 8 weeks post winter:4 week winter	1.013	1.491	-0.4784	0.1627	6	6
161	4 weeks post winter:2 week winter vs. 8 weeks post winter:8 week winter	1.013	2.214	-1.202	0.1627	6	6
162	4 weeks post winter:2 week winter vs. SPC -4 weeks post winter:2 week winter	1.013	0.2413	0.7714	0.1627	6	6
163	4 weeks post winter:2 week winter vs. SPC -4 weeks post winter:4 week winter	1.013	0.3452	0.6674	0.1627	6	6
164	4 weeks post winter:2 week winter vs. SPC -4 weeks post winter:8 week winter	1.013	0.5018	0.5108	0.1627	6	6
165	4 weeks post winter:2 week winter vs. SPC -8 weeks post winter:2 week winter	1.013	0.3252	0.6874	0.1627	6	6
166	4 weeks post winter:2 week winter vs. SPC -8 weeks post winter:4 week winter	1.013	0.5018	0.5108	0.1627	6	6
167	4 weeks post winter:2 week winter vs. SPC -8 weeks post winter:8 week winter	1.013	0.9474	0.0652	0.1627	6	6
168	4 weeks post winter:4 week winter vs. 4 weeks post winter:8 week winter	1.323	1.589	-0.2656	0.1627	6	6
169	4 weeks post winter:4 week winter vs. 8 weeks post winter:2 week winter	1.323	1.048	0.2757	0.1627	6	6
170	4 weeks post winter:4 week winter vs. 8 weeks post winter:4 week winter	1.323	1.491	-0.1675	0.1627	6	6
171	4 weeks post winter:4 week winter vs. 8 weeks post winter:8 week winter	1.323	2.214	-0.8909	0.1627	6	6
172	4 weeks post winter:4 week winter vs. SPC -4 weeks post winter:2 week winter	1.323	0.2413	1.082	0.1627	6	6
173	4 weeks post winter:4 week winter vs. SPC -4 weeks post winter:4 week winter	1.323	0.3452	0.9783	0.1627	6	6
174	4 weeks post winter:4 week winter vs. SPC -4 weeks post winter:8 week winter	1.323	0.5018	0.8216	0.1627	6	6
175	4 weeks post winter:4 week winter vs. SPC -8 weeks post winter:2 week winter	1.323	0.3252	0.9983	0.1627	6	6
176	4 weeks post winter:4 week winter vs. SPC -8 weeks post winter:4 week winter	1.323	0.5018	0.8216	0.1627	6	6
177	4 weeks post winter:4 week winter vs. SPC -8 weeks post winter:8 week winter	1.323	0.9474	0.376	0.1627	6	6
178	4 weeks post winter:8 week winter vs. 8 weeks post winter:2 week winter	1.589	1.048	0.5414	0.1627	6	6
179	4 weeks post winter:8 week winter vs. 8 weeks post winter:4 week winter	1.589	1.491	0.09809	0.1627	6	6
180	4 weeks post winter:8 week winter vs. 8 weeks post winter:8 week winter	1.589	2.214	-0.6253	0.1627	6	6

2way ANOVA<br>Multiple comparisons							
181	4 weeks post winter:8 week winter vs. SPC -4 weeks post winter:2 week winter	1.589	0.2413	1.348	0.1627	6	6
182	4 weeks post winter:8 week winter vs. SPC -4 weeks post winter:4 week winter	1.589	0.3452	1.244	0.1627	6	6
183	4 weeks post winter:8 week winter vs. SPC -4 weeks post winter:8 week winter	1.589	0.5018	1.087	0.1627	6	6
184	4 weeks post winter:8 week winter vs. SPC -8 weeks post winter:2 week winter	1.589	0.3252	1.264	0.1627	6	6
185	4 weeks post winter:8 week winter vs. SPC -8 weeks post winter:4 week winter	1.589	0.5018	1.087	0.1627	6	6
186	4 weeks post winter:8 week winter vs. SPC -8 weeks post winter:8 week winter	1.589	0.9474	0.6417	0.1627	6	6
187	8 weeks post winter:2 week winter vs. 8 weeks post winter:4 week winter	1.048	1.491	-0.4433	0.1627	6	6
188	8 weeks post winter:2 week winter vs. 8 weeks post winter:8 week winter	1.048	2.214	-1.167	0.1627	6	6
189	8 weeks post winter:2 week winter vs. SPC -4 weeks post winter:2 week winter	1.048	0.2413	0.8065	0.1627	6	6
190	8 weeks post winter:2 week winter vs. SPC -4 weeks post winter:4 week winter	1.048	0.3452	0.7025	0.1627	6	6
191	8 weeks post winter:2 week winter vs. SPC -4 weeks post winter:8 week winter	1.048	0.5018	0.5459	0.1627	6	6
192	8 weeks post winter:2 week winter vs. SPC -8 weeks post winter:2 week winter	1.048	0.3252	0.7225	0.1627	6	6
193	8 weeks post winter:2 week winter vs. SPC -8 weeks post winter:4 week winter	1.048	0.5018	0.5459	0.1627	6	6
194	8 weeks post winter:2 week winter vs. SPC -8 weeks post winter:8 week winter	1.048	0.9474	0.1003	0.1627	6	6
195	8 weeks post winter:4 week winter vs. 8 weeks post winter:8 week winter	1.491	2.214	-0.7234	0.1627	6	6
196	8 weeks post winter:4 week winter vs. SPC -4 weeks post winter:2 week winter	1.491	0.2413	1.25	0.1627	6	6
197	8 weeks post winter:4 week winter vs. SPC -4 weeks post winter:4 week winter	1.491	0.3452	1.146	0.1627	6	6
198	8 weeks post winter:4 week winter vs. SPC -4 weeks post winter:8 week winter	1.491	0.5018	0.9892	0.1627	6	6
199	8 weeks post winter:4 week winter vs. SPC -8 weeks post winter:2 week winter	1.491	0.3252	1.166	0.1627	6	6
200	8 weeks post winter:4 week winter vs. SPC -8 weeks post winter:4 week winter	1.491	0.5018	0.9892	0.1627	6	6
201	8 weeks post winter:4 week winter vs. SPC -8 weeks post winter:8 week winter	1.491	0.9474	0.5436	0.1627	6	6
202	8 weeks post winter:8 week winter vs. SPC -4 weeks post winter:2 week winter	2.214	0.2413	1.973	0.1627	6	6
203	8 weeks post winter:8 week winter vs. SPC -4 weeks post winter:4 week winter	2.214	0.3452	1.869	0.1627	6	6
204	8 weeks post winter:8 week winter vs. SPC -4 weeks post winter:8 week winter	2.214	0.5018	1.713	0.1627	6	6
205	8 weeks post winter:8 week winter vs. SPC -8 weeks post winter:2 week winter	2.214	0.3252	1.889	0.1627	6	6
206	8 weeks post winter:8 week winter vs. SPC -8 weeks post winter:4 week winter	2.214	0.5018	1.713	0.1627	6	6
207	8 weeks post winter:8 week winter vs. SPC -8 weeks post winter:8 week winter	2.214	0.9474	1.267	0.1627	6	6
208	SPC -4 weeks post winter:2 week winter vs. SPC -4 weeks post winter:4 week winter	0.2413	0.3452	-0.1039	0.1627	6	6
209	SPC -4 weeks post winter:2 week winter vs. SPC -4 weeks post winter:8 week winter	0.2413	0.5018	-0.2606	0.1627	6	6
210	SPC -4 weeks post winter:2 week winter vs. SPC -8 weeks post winter:2 week winter	0.2413	0.3252	-0.08393	0.1627	6	6

**2way ANOVA**<br>Multiple comparisons							
211	SPC -4 weeks post winter:2 week winter vs. SPC -8 weeks post winter:4 week winter	0.2413	0.5018	-0.2606	0.1627	6	6
212	SPC -4 weeks post winter:2 week winter vs. SPC -8 weeks post winter:8 week winter	0.2413	0.9474	-0.7062	0.1627	6	6
213	SPC -4 weeks post winter:4 week winter vs. SPC -4 weeks post winter:8 week winter	0.3452	0.5018	-0.1566	0.1627	6	6
214	SPC -4 weeks post winter:4 week winter vs. SPC -8 weeks post winter:2 week winter	0.3452	0.3252	0.02	0.1627	6	6
215	SPC -4 weeks post winter:4 week winter vs. SPC -8 weeks post winter:4 week winter	0.3452	0.5018	-0.1566	0.1627	6	6
216	SPC -4 weeks post winter:4 week winter vs. SPC -8 weeks post winter:8 week winter	0.3452	0.9474	-0.6022	0.1627	6	6
217	SPC -4 weeks post winter:8 week winter vs. SPC -8 weeks post winter:2 week winter	0.5018	0.3252	0.1766	0.1627	6	6
218	SPC -4 weeks post winter:8 week winter vs. SPC -8 weeks post winter:4 week winter	0.5018	0.5018	0	0.1627	6	6
219	SPC -4 weeks post winter:8 week winter vs. SPC -8 weeks post winter:8 week winter	0.5018	0.9474	-0.4456	0.1627	6	6
220	SPC -8 weeks post winter:2 week winter vs. SPC -8 weeks post winter:4 week winter	0.3252	0.5018	-0.1766	0.1627	6	6
221	SPC -8 weeks post winter:2 week winter vs. SPC -8 weeks post winter:8 week winter	0.3252	0.9474	-0.6222	0.1627	6	6
222	SPC -8 weeks post winter:4 week winter vs. SPC -8 weeks post winter:8 week winter	0.5018	0.9474	-0.4456	0.1627	6	6

1		
2		
3		
4		
5		
6		
7		
8		
9		
10		
11		
12		
13		
14		
15		
16		
17		
18		
19		
20		
21		
22		
23		
24		
25		
26		
27		
28		
29		
30		

31		
32		
33		
34		
35		
36		
37		
38		
39		
40		
41		
42		
43		
44		
45		
46		
47		
48		
49		
50		
51		
52		
53		
54		
55		
56		
57		
58		
59		
60		

61		
62		
63		
64		
65		
66		
67		
68		
69		
70		
71		
72		
73		
74		
75		
76		
77		
78		
79		
80		
81		
82		
83		
84		
85		
86		
87		
88		
89		
90		

91		
92		
93		
94		
95		
96		
97		
98		
99		
100		
101		
102		
103		
104		
105		
106		
107		
108		
109		
110		
111		
112		
113		
114		
115		
116	q	DF
117		
118	0.9489	75
119	2.383	75
120	3.418	75

121	6.119	75
122	8.427	75
123	3.723	75
124	7.575	75
125	13.86	75
126	3.286	75
127	2.383	75
128	1.022	75
129	2.557	75
130	1.022	75
131	2.851	75
132	1.434	75
133	4.366	75
134	7.068	75
135	9.376	75
136	4.671	75
137	8.524	75
138	14.81	75
139	2.337	75
140	1.434	75
141	0.07277	75
142	1.608	75
143	0.07277	75
144	3.8	75
145	5.8	75
146	8.502	75
147	10.81	75
148	6.105	75
149	9.958	75
150	16.24	75

151	0.9033	75
152	0	75
153	1.361	75
154	0.1738	75
155	1.361	75
156	5.234	75
157	2.701	75
158	5.01	75
159	0.305	75
160	4.157	75
161	10.44	75
162	6.704	75
163	5.8	75
164	4.439	75
165	5.974	75
166	4.439	75
167	0.5667	75
168	2.309	75
169	2.396	75
170	1.456	75
171	7.743	75
172	9.405	75
173	8.502	75
174	7.141	75
175	8.676	75
176	7.141	75
177	3.268	75
178	4.705	75
179	0.8524	75
180	5.434	75

181	11.71	75
182	10.81	75
183	9.449	75
184	10.98	75
185	9.449	75
186	5.577	75
187	3.852	75
188	10.14	75
189	7.009	75
190	6.105	75
191	4.744	75
192	6.279	75
193	4.744	75
194	0.8716	75
195	6.287	75
196	10.86	75
197	9.958	75
198	8.597	75
199	10.13	75
200	8.597	75
201	4.724	75
202	17.15	75
203	16.24	75
204	14.88	75
205	16.42	75
206	14.88	75
207	11.01	75
208	0.9033	75
209	2.264	75
210	0.7294	75

211	2.264	75
212	6.137	75
213	1.361	75
214	0.1738	75
215	1.361	75
216	5.234	75
217	1.535	75
218	0	75
219	3.873	75
220	1.535	75
221	5.408	75
222	3.873	75

2way ANOVA
Tabular results

1	Table Analyzed	NKA 2-ANOVA				
2						
3	Two-way ANOVA	Ordinary				
4	Alpha	0.05				
5						
6	Source of Variation	% of total variation	P value	P value summary	Significant?	
7	Interaction	5.186	0.0836	ns	No	
8	Time	62.11	<0.0001	****	Yes	
9	Treatment	6.208	0.0004	***	Yes	
10						
11	ANOVA table	SS	DF	MS	F (DFn, DFd)	P value
12	Interaction	94.62	8	11.83	F (8, 75) = 1.835	P=0.0836
13	Time	1133	4	283.3	F (4, 75) = 43.96	P<0.0001
14	Treatment	113.3	2	56.63	F (2, 75) = 8.787	P=0.0004
15	Residual	483.3	75	6.444		
16						
17	Number of missing values	0				

2way ANOVA
Multiple comparisons

1	Compare cell means regardless of rows and columns						
2							
3	Number of families	1					
4	Number of comparisons per family	105					
5	Alpha	0.05					
6							
7	Tukey's multiple comparisons test	Mean Diff.	95.00% CI of diff.	Significant?	Summary	Adjusted P Value	
8							
9	End of winter:2 week winter vs. End of winter:4 week winter	0.2993	-4.841 to 5.439	No	ns	>0.9999	
10	End of winter:2 week winter vs. End of winter:8 week winter	0.5384	-4.602 to 5.678	No	ns	>0.9999	
11	End of winter:2 week winter vs. 4 weeks post winter:2 week winter	-6.003	-11.14 to -0.8625	Yes	**	0.0084	
12	End of winter:2 week winter vs. 4 weeks post winter:4 week winter	-7.602	-12.74 to -2.462	Yes	***	0.0002	
13	End of winter:2 week winter vs. 4 weeks post winter:8 week winter	-8.31	-13.45 to -3.17	Yes	****	<0.0001	
14	End of winter:2 week winter vs. 8 weeks post winter:2 week winter	-4.064	-9.204 to 1.076	No	ns	0.2843	
15	End of winter:2 week winter vs. 8 weeks post winter:4 week winter	-7.953	-13.09 to -2.813	Yes	****	<0.0001	
16	End of winter:2 week winter vs. 8 weeks post winter:8 week winter	-11.11	-16.25 to -5.965	Yes	****	<0.0001	
17	End of winter:2 week winter vs. SPC -4 weeks post winter:2 week winter	0.7296	-4.41 to 5.87	No	ns	>0.9999	
18	End of winter:2 week winter vs. SPC -4 weeks post winter:4 week winter	0.5384	-4.602 to 5.678	No	ns	>0.9999	
19	End of winter:2 week winter vs. SPC -4 weeks post winter:8 week winter	-1.18	-6.32 to 3.96	No	ns	>0.9999	
20	End of winter:2 week winter vs. SPC -8 weeks post winter:2 week winter	0.006419	-5.134 to 5.146	No	ns	>0.9999	
21	End of winter:2 week winter vs. SPC -8 weeks post winter:4 week winter	-1.18	-6.32 to 3.96	No	ns	>0.9999	
22	End of winter:2 week winter vs. SPC -8 weeks post winter:8 week winter	-3.008	-8.148 to 2.132	No	ns	0.7605	
23	End of winter:4 week winter vs. End of winter:8 week winter	0.2391	-4.901 to 5.379	No	ns	>0.9999	
24	End of winter:4 week winter vs. 4 weeks post winter:2 week winter	-6.302	-11.44 to -1.162	Yes	**	0.0042	
25	End of winter:4 week winter vs. 4 weeks post winter:4 week winter	-7.901	-13.04 to -2.761	Yes	****	<0.0001	
26	End of winter:4 week winter vs. 4 weeks post winter:8 week winter	-8.609	-13.75 to -3.469	Yes	****	<0.0001	
27	End of winter:4 week winter vs. 8 weeks post winter:2 week winter	-4.363	-9.503 to 0.7771	No	ns	0.1872	
28	End of winter:4 week winter vs. 8 weeks post winter:4 week winter	-8.252	-13.39 to -3.112	Yes	****	<0.0001	
29	End of winter:4 week winter vs. 8 weeks post winter:8 week winter	-11.4	-16.54 to -6.264	Yes	****	<0.0001	
30	End of winter:4 week winter vs. SPC -4 weeks post winter:2 week winter	0.4303	-4.71 to 5.57	No	ns	>0.9999	

2way ANOVA
Multiple comparisons

31	End of winter:4 week winter vs. SPC -4 weeks post winter:4 week winter	0.2391	-4.901 to 5.379	No	ns	>0.9999	
32	End of winter:4 week winter vs. SPC -4 weeks post winter:8 week winter	-1.479	-6.619 to 3.661	No	ns	0.9995	
33	End of winter:4 week winter vs. SPC -8 weeks post winter:2 week winter	-0.2929	-5.433 to 4.847	No	ns	>0.9999	
34	End of winter:4 week winter vs. SPC -8 weeks post winter:4 week winter	-1.479	-6.619 to 3.661	No	ns	0.9995	
35	End of winter:4 week winter vs. SPC -8 weeks post winter:8 week winter	-3.307	-8.448 to 1.833	No	ns	0.6243	
36	End of winter:8 week winter vs. 4 weeks post winter:2 week winter	-6.541	-11.68 to -1.401	Yes	**	0.0024	
37	End of winter:8 week winter vs. 4 weeks post winter:4 week winter	-8.14	-13.28 to -3	Yes	****	<0.0001	
38	End of winter:8 week winter vs. 4 weeks post winter:8 week winter	-8.848	-13.99 to -3.708	Yes	****	<0.0001	
39	End of winter:8 week winter vs. 8 weeks post winter:2 week winter	-4.602	-9.742 to 0.538	No	ns	0.1291	
40	End of winter:8 week winter vs. 8 weeks post winter:4 week winter	-8.491	-13.63 to -3.351	Yes	****	<0.0001	
41	End of winter:8 week winter vs. 8 weeks post winter:8 week winter	-11.64	-16.78 to -6.503	Yes	****	<0.0001	
42	End of winter:8 week winter vs. SPC -4 weeks post winter:2 week winter	0.1912	-4.949 to 5.331	No	ns	>0.9999	
43	End of winter:8 week winter vs. SPC -4 weeks post winter:4 week winter	0	-5.14 to 5.14	No	ns	>0.9999	
44	End of winter:8 week winter vs. SPC -4 weeks post winter:8 week winter	-1.718	-6.858 to 3.422	No	ns	0.9973	
45	End of winter:8 week winter vs. SPC -8 weeks post winter:2 week winter	-0.532	-5.672 to 4.608	No	ns	>0.9999	
46	End of winter:8 week winter vs. SPC -8 weeks post winter:4 week winter	-1.718	-6.858 to 3.422	No	ns	0.9973	
47	End of winter:8 week winter vs. SPC -8 weeks post winter:8 week winter	-3.547	-8.687 to 1.593	No	ns	0.5088	
48	4 weeks post winter:2 week winter vs. 4 weeks post winter:4 week winter	-1.599	-6.739 to 3.541	No	ns	0.9987	
49	4 weeks post winter:2 week winter vs. 4 weeks post winter:8 week winter	-2.307	-7.447 to 2.833	No	ns	0.9591	
50	4 weeks post winter:2 week winter vs. 8 weeks post winter:2 week winter	1.939	-3.201 to 7.079	No	ns	0.9910	
51	4 weeks post winter:2 week winter vs. 8 weeks post winter:4 week winter	-1.95	-7.09 to 3.19	No	ns	0.9905	
52	4 weeks post winter:2 week winter vs. 8 weeks post winter:8 week winter	-5.103	-10.24 to 0.03741	No	ns	0.0537	
53	4 weeks post winter:2 week winter vs. SPC -4 weeks post winter:2 week winter	6.732	1.592 to 11.87	Yes	**	0.0015	
54	4 weeks post winter:2 week winter vs. SPC -4 weeks post winter:4 week winter	6.541	1.401 to 11.68	Yes	**	0.0024	
55	4 weeks post winter:2 week winter vs. SPC -4 weeks post winter:8 week winter	4.823	-0.3173 to 9.963	No	ns	0.0890	
56	4 weeks post winter:2 week winter vs. SPC -8 weeks post winter:2 week winter	6.009	0.8689 to 11.15	Yes	**	0.0083	
57	4 weeks post winter:2 week winter vs. SPC -8 weeks post winter:4 week winter	4.823	-0.3173 to 9.963	No	ns	0.0890	
58	4 weeks post winter:2 week winter vs. SPC -8 weeks post winter:8 week winter	2.994	-2.146 to 8.134	No	ns	0.7662	
59	4 weeks post winter:4 week winter vs. 4 weeks post winter:8 week winter	-0.708	-5.848 to 4.432	No	ns	>0.9999	
60	4 weeks post winter:4 week winter vs. 8 weeks post winter:2 week winter	3.538	-1.602 to 8.678	No	ns	0.5128	

2way ANOVA<br>Multiple comparisons							
61	4 weeks post winter:4 week winter vs. 8 weeks post winter:4 week winter	-0.3511	-5.491 to 4.789	No	ns	>0.9999	
62	4 weeks post winter:4 week winter vs. 8 weeks post winter:8 week winter	-3.503	-8.643 to 1.637	No	ns	0.5295	
63	4 weeks post winter:4 week winter vs. SPC -4 weeks post winter:2 week winter	8.331	3.191 to 13.47	Yes	****	<0.0001	
64	4 weeks post winter:4 week winter vs. SPC -4 weeks post winter:4 week winter	8.14	3 to 13.28	Yes	****	<0.0001	
65	4 weeks post winter:4 week winter vs. SPC -4 weeks post winter:8 week winter	6.422	1.282 to 11.56	Yes	**	0.0032	
66	4 weeks post winter:4 week winter vs. SPC -8 weeks post winter:2 week winter	7.608	2.468 to 12.75	Yes	***	0.0002	
67	4 weeks post winter:4 week winter vs. SPC -8 weeks post winter:4 week winter	6.422	1.282 to 11.56	Yes	**	0.0032	
68	4 weeks post winter:4 week winter vs. SPC -8 weeks post winter:8 week winter	4.593	-0.5465 to 9.734	No	ns	0.1308	
69	4 weeks post winter:8 week winter vs. 8 weeks post winter:2 week winter	4.246	-0.8939 to 9.386	No	ns	0.2219	
70	4 weeks post winter:8 week winter vs. 8 weeks post winter:4 week winter	0.3569	-4.783 to 5.497	No	ns	>0.9999	
71	4 weeks post winter:8 week winter vs. 8 weeks post winter:8 week winter	-2.795	-7.935 to 2.345	No	ns	0.8419	
72	4 weeks post winter:8 week winter vs. SPC -4 weeks post winter:2 week winter	9.039	3.899 to 14.18	Yes	****	<0.0001	
73	4 weeks post winter:8 week winter vs. SPC -4 weeks post winter:4 week winter	8.848	3.708 to 13.99	Yes	****	<0.0001	
74	4 weeks post winter:8 week winter vs. SPC -4 weeks post winter:8 week winter	7.13	1.99 to 12.27	Yes	***	0.0006	
75	4 weeks post winter:8 week winter vs. SPC -8 weeks post winter:2 week winter	8.316	3.176 to 13.46	Yes	****	<0.0001	
76	4 weeks post winter:8 week winter vs. SPC -8 weeks post winter:4 week winter	7.13	1.99 to 12.27	Yes	***	0.0006	
77	4 weeks post winter:8 week winter vs. SPC -8 weeks post winter:8 week winter	5.302	0.1615 to 10.44	Yes	*	0.0366	
78	8 weeks post winter:2 week winter vs. 8 weeks post winter:4 week winter	-3.889	-9.029 to 1.251	No	ns	0.3532	
79	8 weeks post winter:2 week winter vs. 8 weeks post winter:8 week winter	-7.042	-12.18 to -1.902	Yes	***	0.0007	
80	8 weeks post winter:2 week winter vs. SPC -4 weeks post winter:2 week winter	4.793	-0.3468 to 9.933	No	ns	0.0937	
81	8 weeks post winter:2 week winter vs. SPC -4 weeks post winter:4 week winter	4.602	-0.538 to 9.742	No	ns	0.1291	
82	8 weeks post winter:2 week winter vs. SPC -4 weeks post winter:8 week winter	2.884	-2.256 to 8.024	No	ns	0.8101	
83	8 weeks post winter:2 week winter vs. SPC -8 weeks post winter:2 week winter	4.07	-1.07 to 9.21	No	ns	0.2819	
84	8 weeks post winter:2 week winter vs. SPC -8 weeks post winter:4 week winter	2.884	-2.256 to 8.024	No	ns	0.8101	
85	8 weeks post winter:2 week winter vs. SPC -8 weeks post winter:8 week winter	1.055	-4.085 to 6.195	No	ns	>0.9999	
86	8 weeks post winter:4 week winter vs. 8 weeks post winter:8 week winter	-3.152	-8.292 to 1.988	No	ns	0.6971	
87	8 weeks post winter:4 week winter vs. SPC -4 weeks post winter:2 week winter	8.682	3.542 to 13.82	Yes	****	<0.0001	
88	8 weeks post winter:4 week winter vs. SPC -4 weeks post winter:4 week winter	8.491	3.351 to 13.63	Yes	****	<0.0001	
89	8 weeks post winter:4 week winter vs. SPC -4 weeks post winter:8 week winter	6.773	1.633 to 11.91	Yes	**	0.0014	
90	8 weeks post winter:4 week winter vs. SPC -8 weeks post winter:2 week winter	7.959	2.819 to 13.1	Yes	****	<0.0001	

2way ANOVA<br>Multiple comparisons							
91	8 weeks post winter:4 week winter vs. SPC -8 weeks post winter:4 week winter	6.773	1.633 to 11.91	Yes	**	0.0014	
92	8 weeks post winter:4 week winter vs. SPC -8 weeks post winter:8 week winter	4.945	-0.1955 to 10.08	No	ns	0.0717	
93	8 weeks post winter:8 week winter vs. SPC -4 weeks post winter:2 week winter	11.83	6.695 to 16.97	Yes	****	<0.0001	
94	8 weeks post winter:8 week winter vs. SPC -4 weeks post winter:4 week winter	11.64	6.503 to 16.78	Yes	****	<0.0001	
95	8 weeks post winter:8 week winter vs. SPC -4 weeks post winter:8 week winter	9.925	4.785 to 15.07	Yes	****	<0.0001	
96	8 weeks post winter:8 week winter vs. SPC -8 weeks post winter:2 week winter	11.11	5.972 to 16.25	Yes	****	<0.0001	
97	8 weeks post winter:8 week winter vs. SPC -8 weeks post winter:4 week winter	9.925	4.785 to 15.07	Yes	****	<0.0001	
98	8 weeks post winter:8 week winter vs. SPC -8 weeks post winter:8 week winter	8.097	2.957 to 13.24	Yes	****	<0.0001	
99	SPC -4 weeks post winter:2 week winter vs. SPC -4 weeks post winter:4 week winter	-0.1912	-5.331 to 4.949	No	ns	>0.9999	
100	SPC -4 weeks post winter:2 week winter vs. SPC -4 weeks post winter:8 week winter	-1.909	-7.049 to 3.231	No	ns	0.9922	
101	SPC -4 weeks post winter:2 week winter vs. SPC -8 weeks post winter:2 week winter	-0.7232	-5.863 to 4.417	No	ns	>0.9999	
102	SPC -4 weeks post winter:2 week winter vs. SPC -8 weeks post winter:4 week winter	-1.909	-7.049 to 3.231	No	ns	0.9922	
103	SPC -4 weeks post winter:2 week winter vs. SPC -8 weeks post winter:8 week winter	-3.738	-8.878 to 1.402	No	ns	0.4192	
104	SPC -4 weeks post winter:4 week winter vs. SPC -4 weeks post winter:8 week winter	-1.718	-6.858 to 3.422	No	ns	0.9973	
105	SPC -4 weeks post winter:4 week winter vs. SPC -8 weeks post winter:2 week winter	-0.532	-5.672 to 4.608	No	ns	>0.9999	
106	SPC -4 weeks post winter:4 week winter vs. SPC -8 weeks post winter:4 week winter	-1.718	-6.858 to 3.422	No	ns	0.9973	
107	SPC -4 weeks post winter:4 week winter vs. SPC -8 weeks post winter:8 week winter	-3.547	-8.687 to 1.593	No	ns	0.5088	
108	SPC -4 weeks post winter:8 week winter vs. SPC -8 weeks post winter:2 week winter	1.186	-3.954 to 6.326	No	ns	>0.9999	
109	SPC -4 weeks post winter:8 week winter vs. SPC -8 weeks post winter:4 week winter	0	-5.14 to 5.14	No	ns	>0.9999	
110	SPC -4 weeks post winter:8 week winter vs. SPC -8 weeks post winter:8 week winter	-1.828	-6.968 to 3.312	No	ns	0.9949	
111	SPC -8 weeks post winter:2 week winter vs. SPC -8 weeks post winter:4 week winter	-1.186	-6.326 to 3.954	No	ns	>0.9999	
112	SPC -8 weeks post winter:2 week winter vs. SPC -8 weeks post winter:8 week winter	-3.015	-8.155 to 2.125	No	ns	0.7578	
113	SPC -8 weeks post winter:4 week winter vs. SPC -8 weeks post winter:8 week winter	-1.828	-6.968 to 3.312	No	ns	0.9949	
114							
115							
116	Test details	Mean 1	Mean 2	Mean Diff.	SE of diff.	N1	N2
117							
118	End of winter:2 week winter vs. End of winter:4 week winter	1.537	1.237	0.2993	1.466	6	6
119	End of winter:2 week winter vs. End of winter:8 week winter	1.537	0.9983	0.5384	1.466	6	6
120	End of winter:2 week winter vs. 4 weeks post winter:2 week winter	1.537	7.539	-6.003	1.466	6	6

2way ANOVA<br>Multiple comparisons							
121	End of winter:2 week winter vs. 4 weeks post winter:4 week winter	1.537	9.138	-7.602	1.466	6	6
122	End of winter:2 week winter vs. 4 weeks post winter:8 week winter	1.537	9.846	-8.31	1.466	6	6
123	End of winter:2 week winter vs. 8 weeks post winter:2 week winter	1.537	5.6	-4.064	1.466	6	6
124	End of winter:2 week winter vs. 8 weeks post winter:4 week winter	1.537	9.489	-7.953	1.466	6	6
125	End of winter:2 week winter vs. 8 weeks post winter:8 week winter	1.537	12.64	-11.11	1.466	6	6
126	End of winter:2 week winter vs. SPC -4 weeks post winter:2 week winter	1.537	0.807	0.7296	1.466	6	6
127	End of winter:2 week winter vs. SPC -4 weeks post winter:4 week winter	1.537	0.9983	0.5384	1.466	6	6
128	End of winter:2 week winter vs. SPC -4 weeks post winter:8 week winter	1.537	2.716	-1.18	1.466	6	6
129	End of winter:2 week winter vs. SPC -8 weeks post winter:2 week winter	1.537	1.53	0.006419	1.466	6	6
130	End of winter:2 week winter vs. SPC -8 weeks post winter:4 week winter	1.537	2.716	-1.18	1.466	6	6
131	End of winter:2 week winter vs. SPC -8 weeks post winter:8 week winter	1.537	4.545	-3.008	1.466	6	6
132	End of winter:4 week winter vs. End of winter:8 week winter	1.237	0.9983	0.2391	1.466	6	6
133	End of winter:4 week winter vs. 4 weeks post winter:2 week winter	1.237	7.539	-6.302	1.466	6	6
134	End of winter:4 week winter vs. 4 weeks post winter:4 week winter	1.237	9.138	-7.901	1.466	6	6
135	End of winter:4 week winter vs. 4 weeks post winter:8 week winter	1.237	9.846	-8.609	1.466	6	6
136	End of winter:4 week winter vs. 8 weeks post winter:2 week winter	1.237	5.6	-4.363	1.466	6	6
137	End of winter:4 week winter vs. 8 weeks post winter:4 week winter	1.237	9.489	-8.252	1.466	6	6
138	End of winter:4 week winter vs. 8 weeks post winter:8 week winter	1.237	12.64	-11.4	1.466	6	6
139	End of winter:4 week winter vs. SPC -4 weeks post winter:2 week winter	1.237	0.807	0.4303	1.466	6	6
140	End of winter:4 week winter vs. SPC -4 weeks post winter:4 week winter	1.237	0.9983	0.2391	1.466	6	6
141	End of winter:4 week winter vs. SPC -4 weeks post winter:8 week winter	1.237	2.716	-1.479	1.466	6	6
142	End of winter:4 week winter vs. SPC -8 weeks post winter:2 week winter	1.237	1.53	-0.2929	1.466	6	6
143	End of winter:4 week winter vs. SPC -8 weeks post winter:4 week winter	1.237	2.716	-1.479	1.466	6	6
144	End of winter:4 week winter vs. SPC -8 weeks post winter:8 week winter	1.237	4.545	-3.307	1.466	6	6
145	End of winter:8 week winter vs. 4 weeks post winter:2 week winter	0.9983	7.539	-6.541	1.466	6	6
146	End of winter:8 week winter vs. 4 weeks post winter:4 week winter	0.9983	9.138	-8.14	1.466	6	6
147	End of winter:8 week winter vs. 4 weeks post winter:8 week winter	0.9983	9.846	-8.848	1.466	6	6
148	End of winter:8 week winter vs. 8 weeks post winter:2 week winter	0.9983	5.6	-4.602	1.466	6	6
149	End of winter:8 week winter vs. 8 weeks post winter:4 week winter	0.9983	9.489	-8.491	1.466	6	6
150	End of winter:8 week winter vs. 8 weeks post winter:8 week winter	0.9983	12.64	-11.64	1.466	6	6

2way ANOVA<br>Multiple comparisons							
151	End of winter:8 week winter vs. SPC -4 weeks post winter:2 week winter	0.9983	0.807	0.1912	1.466	6	6
152	End of winter:8 week winter vs. SPC -4 weeks post winter:4 week winter	0.9983	0.9983	0	1.466	6	6
153	End of winter:8 week winter vs. SPC -4 weeks post winter:8 week winter	0.9983	2.716	-1.718	1.466	6	6
154	End of winter:8 week winter vs. SPC -8 weeks post winter:2 week winter	0.9983	1.53	-0.532	1.466	6	6
155	End of winter:8 week winter vs. SPC -8 weeks post winter:4 week winter	0.9983	2.716	-1.718	1.466	6	6
156	End of winter:8 week winter vs. SPC -8 weeks post winter:8 week winter	0.9983	4.545	-3.547	1.466	6	6
157	4 weeks post winter:2 week winter vs. 4 weeks post winter:4 week winter	7.539	9.138	-1.599	1.466	6	6
158	4 weeks post winter:2 week winter vs. 4 weeks post winter:8 week winter	7.539	9.846	-2.307	1.466	6	6
159	4 weeks post winter:2 week winter vs. 8 weeks post winter:2 week winter	7.539	5.6	1.939	1.466	6	6
160	4 weeks post winter:2 week winter vs. 8 weeks post winter:4 week winter	7.539	9.489	-1.95	1.466	6	6
161	4 weeks post winter:2 week winter vs. 8 weeks post winter:8 week winter	7.539	12.64	-5.103	1.466	6	6
162	4 weeks post winter:2 week winter vs. SPC -4 weeks post winter:2 week winter	7.539	0.807	6.732	1.466	6	6
163	4 weeks post winter:2 week winter vs. SPC -4 weeks post winter:4 week winter	7.539	0.9983	6.541	1.466	6	6
164	4 weeks post winter:2 week winter vs. SPC -4 weeks post winter:8 week winter	7.539	2.716	4.823	1.466	6	6
165	4 weeks post winter:2 week winter vs. SPC -8 weeks post winter:2 week winter	7.539	1.53	6.009	1.466	6	6
166	4 weeks post winter:2 week winter vs. SPC -8 weeks post winter:4 week winter	7.539	2.716	4.823	1.466	6	6
167	4 weeks post winter:2 week winter vs. SPC -8 weeks post winter:8 week winter	7.539	4.545	2.994	1.466	6	6
168	4 weeks post winter:4 week winter vs. 4 weeks post winter:8 week winter	9.138	9.846	-0.708	1.466	6	6
169	4 weeks post winter:4 week winter vs. 8 weeks post winter:2 week winter	9.138	5.6	3.538	1.466	6	6
170	4 weeks post winter:4 week winter vs. 8 weeks post winter:4 week winter	9.138	9.489	-0.3511	1.466	6	6
171	4 weeks post winter:4 week winter vs. 8 weeks post winter:8 week winter	9.138	12.64	-3.503	1.466	6	6
172	4 weeks post winter:4 week winter vs. SPC -4 weeks post winter:2 week winter	9.138	0.807	8.331	1.466	6	6
173	4 weeks post winter:4 week winter vs. SPC -4 weeks post winter:4 week winter	9.138	0.9983	8.14	1.466	6	6
174	4 weeks post winter:4 week winter vs. SPC -4 weeks post winter:8 week winter	9.138	2.716	6.422	1.466	6	6
175	4 weeks post winter:4 week winter vs. SPC -8 weeks post winter:2 week winter	9.138	1.53	7.608	1.466	6	6
176	4 weeks post winter:4 week winter vs. SPC -8 weeks post winter:4 week winter	9.138	2.716	6.422	1.466	6	6
177	4 weeks post winter:4 week winter vs. SPC -8 weeks post winter:8 week winter	9.138	4.545	4.593	1.466	6	6
178	4 weeks post winter:8 week winter vs. 8 weeks post winter:2 week winter	9.846	5.6	4.246	1.466	6	6
179	4 weeks post winter:8 week winter vs. 8 weeks post winter:4 week winter	9.846	9.489	0.3569	1.466	6	6
180	4 weeks post winter:8 week winter vs. 8 weeks post winter:8 week winter	9.846	12.64	-2.795	1.466	6	6

2way ANOVA
Multiple comparisons

181	4 weeks post winter:8 week winter vs. SPC -4 weeks post winter:2 week winter	9.846	0.807	9.039	1.466	6	6
182	4 weeks post winter:8 week winter vs. SPC -4 weeks post winter:4 week winter	9.846	0.9983	8.848	1.466	6	6
183	4 weeks post winter:8 week winter vs. SPC -4 weeks post winter:8 week winter	9.846	2.716	7.13	1.466	6	6
184	4 weeks post winter:8 week winter vs. SPC -8 weeks post winter:2 week winter	9.846	1.53	8.316	1.466	6	6
185	4 weeks post winter:8 week winter vs. SPC -8 weeks post winter:4 week winter	9.846	2.716	7.13	1.466	6	6
186	4 weeks post winter:8 week winter vs. SPC -8 weeks post winter:8 week winter	9.846	4.545	5.302	1.466	6	6
187	8 weeks post winter:2 week winter vs. 8 weeks post winter:4 week winter	5.6	9.489	-3.889	1.466	6	6
188	8 weeks post winter:2 week winter vs. 8 weeks post winter:8 week winter	5.6	12.64	-7.042	1.466	6	6
189	8 weeks post winter:2 week winter vs. SPC -4 weeks post winter:2 week winter	5.6	0.807	4.793	1.466	6	6
190	8 weeks post winter:2 week winter vs. SPC -4 weeks post winter:4 week winter	5.6	0.9983	4.602	1.466	6	6
191	8 weeks post winter:2 week winter vs. SPC -4 weeks post winter:8 week winter	5.6	2.716	2.884	1.466	6	6
192	8 weeks post winter:2 week winter vs. SPC -8 weeks post winter:2 week winter	5.6	1.53	4.07	1.466	6	6
193	8 weeks post winter:2 week winter vs. SPC -8 weeks post winter:4 week winter	5.6	2.716	2.884	1.466	6	6
194	8 weeks post winter:2 week winter vs. SPC -8 weeks post winter:8 week winter	5.6	4.545	1.055	1.466	6	6
195	8 weeks post winter:4 week winter vs. 8 weeks post winter:8 week winter	9.489	12.64	-3.152	1.466	6	6
196	8 weeks post winter:4 week winter vs. SPC -4 weeks post winter:2 week winter	9.489	0.807	8.682	1.466	6	6
197	8 weeks post winter:4 week winter vs. SPC -4 weeks post winter:4 week winter	9.489	0.9983	8.491	1.466	6	6
198	8 weeks post winter:4 week winter vs. SPC -4 weeks post winter:8 week winter	9.489	2.716	6.773	1.466	6	6
199	8 weeks post winter:4 week winter vs. SPC -8 weeks post winter:2 week winter	9.489	1.53	7.959	1.466	6	6
200	8 weeks post winter:4 week winter vs. SPC -8 weeks post winter:4 week winter	9.489	2.716	6.773	1.466	6	6
201	8 weeks post winter:4 week winter vs. SPC -8 weeks post winter:8 week winter	9.489	4.545	4.945	1.466	6	6
202	8 weeks post winter:8 week winter vs. SPC -4 weeks post winter:2 week winter	12.64	0.807	11.83	1.466	6	6
203	8 weeks post winter:8 week winter vs. SPC -4 weeks post winter:4 week winter	12.64	0.9983	11.64	1.466	6	6
204	8 weeks post winter:8 week winter vs. SPC -4 weeks post winter:8 week winter	12.64	2.716	9.925	1.466	6	6
205	8 weeks post winter:8 week winter vs. SPC -8 weeks post winter:2 week winter	12.64	1.53	11.11	1.466	6	6
206	8 weeks post winter:8 week winter vs. SPC -8 weeks post winter:4 week winter	12.64	2.716	9.925	1.466	6	6
207	8 weeks post winter:8 week winter vs. SPC -8 weeks post winter:8 week winter	12.64	4.545	8.097	1.466	6	6
208	SPC -4 weeks post winter:2 week winter vs. SPC -4 weeks post winter:4 week winter	0.807	0.9983	-0.1912	1.466	6	6
209	SPC -4 weeks post winter:2 week winter vs. SPC -4 weeks post winter:8 week winter	0.807	2.716	-1.909	1.466	6	6
210	SPC -4 weeks post winter:2 week winter vs. SPC -8 weeks post winter:2 week winter	0.807	1.53	-0.7232	1.466	6	6

2way ANOVA
Multiple comparisons

211	SPC -4 weeks post winter:2 week winter vs. SPC -8 weeks post winter:4 week winter	0.807	2.716	-1.909	1.466	6	6
212	SPC -4 weeks post winter:2 week winter vs. SPC -8 weeks post winter:8 week winter	0.807	4.545	-3.738	1.466	6	6
213	SPC -4 weeks post winter:4 week winter vs. SPC -4 weeks post winter:8 week winter	0.9983	2.716	-1.718	1.466	6	6
214	SPC -4 weeks post winter:4 week winter vs. SPC -8 weeks post winter:2 week winter	0.9983	1.53	-0.532	1.466	6	6
215	SPC -4 weeks post winter:4 week winter vs. SPC -8 weeks post winter:4 week winter	0.9983	2.716	-1.718	1.466	6	6
216	SPC -4 weeks post winter:4 week winter vs. SPC -8 weeks post winter:8 week winter	0.9983	4.545	-3.547	1.466	6	6
217	SPC -4 weeks post winter:8 week winter vs. SPC -8 weeks post winter:2 week winter	2.716	1.53	1.186	1.466	6	6
218	SPC -4 weeks post winter:8 week winter vs. SPC -8 weeks post winter:4 week winter	2.716	2.716	0	1.466	6	6
219	SPC -4 weeks post winter:8 week winter vs. SPC -8 weeks post winter:8 week winter	2.716	4.545	-1.828	1.466	6	6
220	SPC -8 weeks post winter:2 week winter vs. SPC -8 weeks post winter:4 week winter	1.53	2.716	-1.186	1.466	6	6
221	SPC -8 weeks post winter:2 week winter vs. SPC -8 weeks post winter:8 week winter	1.53	4.545	-3.015	1.466	6	6
222	SPC -8 weeks post winter:4 week winter vs. SPC -8 weeks post winter:8 week winter	2.716	4.545	-1.828	1.466	6	6

1		
2		
3		
4		
5		
6		
7		
8		
9		
10		
11		
12		
13		
14		
15		
16		
17		
18		
19		
20		
21		
22		
23		
24		
25		
26		
27		
28		
29		
30		

31		
32		
33		
34		
35		
36		
37		
38		
39		
40		
41		
42		
43		
44		
45		
46		
47		
48		
49		
50		
51		
52		
53		
54		
55		
56		
57		
58		
59		
60		

61		
62		
63		
64		
65		
66		
67		
68		
69		
70		
71		
72		
73		
74		
75		
76		
77		
78		
79		
80		
81		
82		
83		
84		
85		
86		
87		
88		
89		
90		

91		
92		
93		
94		
95		
96		
97		
98		
99		
100		
101		
102		
103		
104		
105		
106		
107		
108		
109		
110		
111		
112		
113		
114		
115		
116	q	DF
117		
118	0.2888	75
119	0.5195	75
120	5.792	75

121	7.335	75
122	8.018	75
123	3.921	75
124	7.674	75
125	10.72	75
126	0.704	75
127	0.5195	75
128	1.138	75
129	0.006194	75
130	1.138	75
131	2.903	75
132	0.2307	75
133	6.081	75
134	7.624	75
135	8.307	75
136	4.21	75
137	7.962	75
138	11	75
139	0.4152	75
140	0.2307	75
141	1.427	75
142	0.2826	75
143	1.427	75
144	3.191	75
145	6.311	75
146	7.854	75
147	8.538	75
148	4.44	75
149	8.193	75
150	11.23	75

151	0.1845	75
152	0	75
153	1.658	75
154	0.5133	75
155	1.658	75
156	3.422	75
157	1.543	75
158	2.226	75
159	1.871	75
160	1.882	75
161	4.924	75
162	6.496	75
163	6.311	75
164	4.654	75
165	5.798	75
166	4.654	75
167	2.889	75
168	0.6832	75
169	3.414	75
170	0.3388	75
171	3.381	75
172	8.039	75
173	7.854	75
174	6.197	75
175	7.341	75
176	6.197	75
177	4.432	75
178	4.097	75
179	0.3444	75
180	2.697	75

181	8.722	75
182	8.538	75
183	6.88	75
184	8.024	75
185	6.88	75
186	5.115	75
187	3.753	75
188	6.794	75
189	4.625	75
190	4.44	75
191	2.783	75
192	3.927	75
193	2.783	75
194	1.018	75
195	3.042	75
196	8.378	75
197	8.193	75
198	6.535	75
199	7.68	75
200	6.535	75
201	4.771	75
202	11.42	75
203	11.23	75
204	9.577	75
205	10.72	75
206	9.577	75
207	7.813	75
208	0.1845	75
209	1.842	75
210	0.6978	75

**211**	1.842	75
**212**	3.607	75
**213**	1.658	75
**214**	0.5133	75
**215**	1.658	75
**216**	3.422	75
**217**	1.145	75
**218**	0	75
**219**	1.764	75
**220**	1.145	75
**221**	2.909	75
**222**	1.764	75

2way ANOVA
Tabular results

1	Table Analyzed	ST6GALNAC2				
2						
3	Two-way ANOVA	Ordinary				
4	Alpha	0.05				
5						
6	Source of Variation	% of total variation	P value	P value summary	Significant?	
7	Interaction	9.997	0.0006	***	Yes	
8	Time post-winter	56.05	<0.0001	****	Yes	
9	Treatment	10.36	<0.0001	****	Yes	
10						
11	ANOVA table	SS	DF	MS	F (DFn, DFd)	P value
12	Interaction	7.233	8	0.9041	F (8, 75) = 3.972	P=0.0006
13	Time post-winter	40.55	4	10.14	F (4, 75) = 44.54	P<0.0001
14	Treatment	7.494	2	3.747	F (2, 75) = 16.46	P<0.0001
15	Residual	17.07	75	0.2276		
16						
17	Number of missing values	0				

2way ANOVA
Multiple comparisons

1	Compare cell means regardless of rows and columns					
2						
3	Number of families	1				
4	Number of comparisons per family	105				
5	Alpha	0.05				
6						
7	Tukey's multiple comparisons test	Mean Diff.	95.00% CI of diff.	Significant?	Summary	Adjusted P Value
8						
9	End of winter:2 weeks of winter vs. End of winter:4 weeks of winter	-0.08302	-1.049 to 0.883	No	ns	>0.9999
10	End of winter:2 weeks of winter vs. End of winter:8 weeks of winter	-0.03097	-0.997 to 0.935	No	ns	>0.9999
11	End of winter:2 weeks of winter vs. 4 weeks post winter:2 weeks of winter	-0.4552	-1.421 to 0.5108	No	ns	0.9403
12	End of winter:2 weeks of winter vs. 4 weeks post winter:4 weeks of winter	-0.6661	-1.632 to 0.2999	No	ns	0.5098
13	End of winter:2 weeks of winter vs. 4 weeks post winter:8 weeks of winter	-1.513	-2.479 to -0.5471	Yes	****	<0.0001
14	End of winter:2 weeks of winter vs. 8 weeks post winter:2 weeks of winter	-0.7676	-1.734 to 0.1984	No	ns	0.2767
15	End of winter:2 weeks of winter vs. 8 weeks post winter:4 weeks of winter	-2.253	-3.219 to -1.287	Yes	****	<0.0001
16	End of winter:2 weeks of winter vs. 8 weeks post winter:8 weeks of winter	-2.438	-3.404 to -1.472	Yes	****	<0.0001
17	End of winter:2 weeks of winter vs. SPC -4 weeks post winter:2 weeks of winter	0.09402	-0.872 to 1.06	No	ns	>0.9999
18	End of winter:2 weeks of winter vs. SPC -4 weeks post winter:4 weeks of winter	-0.03097	-0.997 to 0.935	No	ns	>0.9999
19	End of winter:2 weeks of winter vs. SPC -4 weeks post winter:8 weeks of winter	-0.2667	-1.233 to 0.6993	No	ns	0.9997
20	End of winter:2 weeks of winter vs. SPC -8 weeks post winter:2 weeks of winter	-0.1138	-1.08 to 0.8522	No	ns	>0.9999
21	End of winter:2 weeks of winter vs. SPC -8 weeks post winter:4 weeks of winter	-0.2667	-1.233 to 0.6993	No	ns	0.9997
22	End of winter:2 weeks of winter vs. SPC -8 weeks post winter:8 weeks of winter	-0.5112	-1.477 to 0.4548	No	ns	0.8665
23	End of winter:4 weeks of winter vs. End of winter:8 weeks of winter	0.05205	-0.9139 to 1.018	No	ns	>0.9999
24	End of winter:4 weeks of winter vs. 4 weeks post winter:2 weeks of winter	-0.3722	-1.338 to 0.5938	No	ns	0.9891
25	End of winter:4 weeks of winter vs. 4 weeks post winter:4 weeks of winter	-0.5831	-1.549 to 0.3829	No	ns	0.7195
26	End of winter:4 weeks of winter vs. 4 weeks post winter:8 weeks of winter	-1.43	-2.396 to -0.4641	Yes	***	0.0002
27	End of winter:4 weeks of winter vs. 8 weeks post winter:2 weeks of winter	-0.6846	-1.651 to 0.2814	No	ns	0.4632
28	End of winter:4 weeks of winter vs. 8 weeks post winter:4 weeks of winter	-2.17	-3.136 to -1.204	Yes	****	<0.0001
29	End of winter:4 weeks of winter vs. 8 weeks post winter:8 weeks of winter	-2.355	-3.321 to -1.389	Yes	****	<0.0001
30	End of winter:4 weeks of winter vs. SPC -4 weeks post winter:2 weeks of winter	0.177	-0.789 to 1.143	No	ns	>0.9999

**2way ANOVA**<br>Multiple comparisons						
31	End of winter:4 weeks of winter vs. SPC -4 weeks post winter:4 weeks of winter	0.05205	-0.9139 to 1.018	No	ns	>0.9999
32	End of winter:4 weeks of winter vs. SPC -4 weeks post winter:8 weeks of winter	-0.1837	-1.15 to 0.7823	No	ns	>0.9999
33	End of winter:4 weeks of winter vs. SPC -8 weeks post winter:2 weeks of winter	-0.03078	-0.9968 to 0.9352	No	ns	>0.9999
34	End of winter:4 weeks of winter vs. SPC -8 weeks post winter:4 weeks of winter	-0.1837	-1.15 to 0.7823	No	ns	>0.9999
35	End of winter:4 weeks of winter vs. SPC -8 weeks post winter:8 weeks of winter	-0.4282	-1.394 to 0.5378	No	ns	0.9630
36	End of winter:8 weeks of winter vs. 4 weeks post winter:2 weeks of winter	-0.4243	-1.39 to 0.5417	No	ns	0.9657
37	End of winter:8 weeks of winter vs. 4 weeks post winter:4 weeks of winter	-0.6352	-1.601 to 0.3308	No	ns	0.5895
38	End of winter:8 weeks of winter vs. 4 weeks post winter:8 weeks of winter	-1.482	-2.448 to -0.5161	Yes	****	<0.0001
39	End of winter:8 weeks of winter vs. 8 weeks post winter:2 weeks of winter	-0.7366	-1.703 to 0.2294	No	ns	0.3406
40	End of winter:8 weeks of winter vs. 8 weeks post winter:4 weeks of winter	-2.222	-3.188 to -1.256	Yes	****	<0.0001
41	End of winter:8 weeks of winter vs. 8 weeks post winter:8 weeks of winter	-2.407	-3.373 to -1.441	Yes	****	<0.0001
42	End of winter:8 weeks of winter vs. SPC -4 weeks post winter:2 weeks of winter	0.125	-0.841 to 1.091	No	ns	>0.9999
43	End of winter:8 weeks of winter vs. SPC -4 weeks post winter:4 weeks of winter	0	-0.966 to 0.966	No	ns	>0.9999
44	End of winter:8 weeks of winter vs. SPC -4 weeks post winter:8 weeks of winter	-0.2357	-1.202 to 0.7303	No	ns	>0.9999
45	End of winter:8 weeks of winter vs. SPC -8 weeks post winter:2 weeks of winter	-0.08283	-1.049 to 0.8832	No	ns	>0.9999
46	End of winter:8 weeks of winter vs. SPC -8 weeks post winter:4 weeks of winter	-0.2357	-1.202 to 0.7303	No	ns	>0.9999
47	End of winter:8 weeks of winter vs. SPC -8 weeks post winter:8 weeks of winter	-0.4803	-1.446 to 0.4857	No	ns	0.9120
48	4 weeks post winter:2 weeks of winter vs. 4 weeks post winter:4 weeks of winter	-0.2109	-1.177 to 0.7551	No	ns	>0.9999
49	4 weeks post winter:2 weeks of winter vs. 4 weeks post winter:8 weeks of winter	-1.058	-2.024 to -0.09189	Yes	*	0.0188
50	4 weeks post winter:2 weeks of winter vs. 8 weeks post winter:2 weeks of winter	-0.3124	-1.278 to 0.6536	No	ns	0.9981
51	4 weeks post winter:2 weeks of winter vs. 8 weeks post winter:4 weeks of winter	-1.797	-2.763 to -0.8314	Yes	****	<0.0001
52	4 weeks post winter:2 weeks of winter vs. 8 weeks post winter:8 weeks of winter	-1.983	-2.949 to -1.017	Yes	****	<0.0001
53	4 weeks post winter:2 weeks of winter vs. SPC -4 weeks post winter:2 weeks of winter	0.5492	-0.4167 to 1.515	No	ns	0.7952
54	4 weeks post winter:2 weeks of winter vs. SPC -4 weeks post winter:4 weeks of winter	0.4243	-0.5417 to 1.39	No	ns	0.9657
55	4 weeks post winter:2 weeks of winter vs. SPC -4 weeks post winter:8 weeks of winter	0.1885	-0.7775 to 1.155	No	ns	>0.9999
56	4 weeks post winter:2 weeks of winter vs. SPC -8 weeks post winter:2 weeks of winter	0.3414	-0.6246 to 1.307	No	ns	0.9952
57	4 weeks post winter:2 weeks of winter vs. SPC -8 weeks post winter:4 weeks of winter	0.1885	-0.7775 to 1.155	No	ns	>0.9999
58	4 weeks post winter:2 weeks of winter vs. SPC -8 weeks post winter:8 weeks of winter	-0.05601	-1.022 to 0.91	No	ns	>0.9999
59	4 weeks post winter:4 weeks of winter vs. 4 weeks post winter:8 weeks of winter	-0.847	-1.813 to 0.119	No	ns	0.1503
60	4 weeks post winter:4 weeks of winter vs. 8 weeks post winter:2 weeks of winter	-0.1014	-1.067 to 0.8645	No	ns	>0.9999

2way ANOVA<br>Multiple comparisons						
61	4 weeks post winter:4 weeks of winter vs. 8 weeks post winter:4 weeks of winter	-1.586	-2.552 to -0.6205	Yes	****	<0.0001
62	4 weeks post winter:4 weeks of winter vs. 8 weeks post winter:8 weeks of winter	-1.772	-2.738 to -0.8058	Yes	****	<0.0001
63	4 weeks post winter:4 weeks of winter vs. SPC -4 weeks post winter:2 weeks of winter	0.7601	-0.2058 to 1.726	No	ns	0.2913
64	4 weeks post winter:4 weeks of winter vs. SPC -4 weeks post winter:4 weeks of winter	0.6352	-0.3308 to 1.601	No	ns	0.5895
65	4 weeks post winter:4 weeks of winter vs. SPC -4 weeks post winter:8 weeks of winter	0.3994	-0.5666 to 1.365	No	ns	0.9794
66	4 weeks post winter:4 weeks of winter vs. SPC -8 weeks post winter:2 weeks of winter	0.5523	-0.4137 to 1.518	No	ns	0.7887
67	4 weeks post winter:4 weeks of winter vs. SPC -8 weeks post winter:4 weeks of winter	0.3994	-0.5666 to 1.365	No	ns	0.9794
68	4 weeks post winter:4 weeks of winter vs. SPC -8 weeks post winter:8 weeks of winter	0.1549	-0.8111 to 1.121	No	ns	>0.9999
69	4 weeks post winter:8 weeks of winter vs. 8 weeks post winter:2 weeks of winter	0.7455	-0.2205 to 1.712	No	ns	0.3214
70	4 weeks post winter:8 weeks of winter vs. 8 weeks post winter:4 weeks of winter	-0.7395	-1.705 to 0.2265	No	ns	0.3344
71	4 weeks post winter:8 weeks of winter vs. 8 weeks post winter:8 weeks of winter	-0.9248	-1.891 to 0.04117	No	ns	0.0748
72	4 weeks post winter:8 weeks of winter vs. SPC -4 weeks post winter:2 weeks of winter	1.607	0.6411 to 2.573	Yes	****	<0.0001
73	4 weeks post winter:8 weeks of winter vs. SPC -4 weeks post winter:4 weeks of winter	1.482	0.5161 to 2.448	Yes	****	<0.0001
74	4 weeks post winter:8 weeks of winter vs. SPC -4 weeks post winter:8 weeks of winter	1.246	0.2804 to 2.212	Yes	**	0.0019
75	4 weeks post winter:8 weeks of winter vs. SPC -8 weeks post winter:2 weeks of winter	1.399	0.4333 to 2.365	Yes	***	0.0003
76	4 weeks post winter:8 weeks of winter vs. SPC -8 weeks post winter:4 weeks of winter	1.246	0.2804 to 2.212	Yes	**	0.0019
77	4 weeks post winter:8 weeks of winter vs. SPC -8 weeks post winter:8 weeks of winter	1.002	0.03588 to 1.968	Yes	*	0.0346
78	8 weeks post winter:2 weeks of winter vs. 8 weeks post winter:4 weeks of winter	-1.485	-2.451 to -0.519	Yes	****	<0.0001
79	8 weeks post winter:2 weeks of winter vs. 8 weeks post winter:8 weeks of winter	-1.67	-2.636 to -0.7044	Yes	****	<0.0001
80	8 weeks post winter:2 weeks of winter vs. SPC -4 weeks post winter:2 weeks of winter	0.8616	-0.1044 to 1.828	No	ns	0.1328
81	8 weeks post winter:2 weeks of winter vs. SPC -4 weeks post winter:4 weeks of winter	0.7366	-0.2294 to 1.703	No	ns	0.3406
82	8 weeks post winter:2 weeks of winter vs. SPC -4 weeks post winter:8 weeks of winter	0.5009	-0.4651 to 1.467	No	ns	0.8830
83	8 weeks post winter:2 weeks of winter vs. SPC -8 weeks post winter:2 weeks of winter	0.6538	-0.3122 to 1.62	No	ns	0.5415
84	8 weeks post winter:2 weeks of winter vs. SPC -8 weeks post winter:4 weeks of winter	0.5009	-0.4651 to 1.467	No	ns	0.8830
85	8 weeks post winter:2 weeks of winter vs. SPC -8 weeks post winter:8 weeks of winter	0.2563	-0.7096 to 1.222	No	ns	0.9998
86	8 weeks post winter:4 weeks of winter vs. 8 weeks post winter:8 weeks of winter	-0.1854	-1.151 to 0.7806	No	ns	>0.9999
87	8 weeks post winter:4 weeks of winter vs. SPC -4 weeks post winter:2 weeks of winter	2.347	1.381 to 3.313	Yes	****	<0.0001
88	8 weeks post winter:4 weeks of winter vs. SPC -4 weeks post winter:4 weeks of winter	2.222	1.256 to 3.188	Yes	****	<0.0001
89	8 weeks post winter:4 weeks of winter vs. SPC -4 weeks post winter:8 weeks of winter	1.986	1.02 to 2.952	Yes	****	<0.0001
90	8 weeks post winter:4 weeks of winter vs. SPC -8 weeks post winter:2 weeks of winter	2.139	1.173 to 3.105	Yes	****	<0.0001

2way ANOVA
Multiple comparisons

91	8 weeks post winter:4 weeks of winter vs. SPC -8 weeks post winter:4 weeks of winter	1.986	1.02 to 2.952	Yes	****	<0.0001
92	8 weeks post winter:4 weeks of winter vs. SPC -8 weeks post winter:8 weeks of winter	1.741	0.7753 to 2.707	Yes	****	<0.0001
93	8 weeks post winter:8 weeks of winter vs. SPC -4 weeks post winter:2 weeks of winter	2.532	1.566 to 3.498	Yes	****	<0.0001
94	8 weeks post winter:8 weeks of winter vs. SPC -4 weeks post winter:4 weeks of winter	2.407	1.441 to 3.373	Yes	****	<0.0001
95	8 weeks post winter:8 weeks of winter vs. SPC -4 weeks post winter:8 weeks of winter	2.171	1.205 to 3.137	Yes	****	<0.0001
96	8 weeks post winter:8 weeks of winter vs. SPC -8 weeks post winter:2 weeks of winter	2.324	1.358 to 3.29	Yes	****	<0.0001
97	8 weeks post winter:8 weeks of winter vs. SPC -8 weeks post winter:4 weeks of winter	2.171	1.205 to 3.137	Yes	****	<0.0001
98	8 weeks post winter:8 weeks of winter vs. SPC -8 weeks post winter:8 weeks of winter	1.927	0.9607 to 2.893	Yes	****	<0.0001
99	SPC -4 weeks post winter:2 weeks of winter vs. SPC -4 weeks post winter:4 weeks of winter	-0.125	-1.091 to 0.841	No	ns	>0.9999
100	SPC -4 weeks post winter:2 weeks of winter vs. SPC -4 weeks post winter:8 weeks of winter	-0.3607	-1.327 to 0.6053	No	ns	0.9919
101	SPC -4 weeks post winter:2 weeks of winter vs. SPC -8 weeks post winter:2 weeks of winter	-0.2078	-1.174 to 0.7582	No	ns	>0.9999
102	SPC -4 weeks post winter:2 weeks of winter vs. SPC -8 weeks post winter:4 weeks of winter	-0.3607	-1.327 to 0.6053	No	ns	0.9919
103	SPC -4 weeks post winter:2 weeks of winter vs. SPC -8 weeks post winter:8 weeks of winter	-0.6053	-1.571 to 0.3607	No	ns	0.6656
104	SPC -4 weeks post winter:4 weeks of winter vs. SPC -4 weeks post winter:8 weeks of winter	-0.2357	-1.202 to 0.7303	No	ns	>0.9999
105	SPC -4 weeks post winter:4 weeks of winter vs. SPC -8 weeks post winter:2 weeks of winter	-0.08283	-1.049 to 0.8832	No	ns	>0.9999
106	SPC -4 weeks post winter:4 weeks of winter vs. SPC -8 weeks post winter:4 weeks of winter	-0.2357	-1.202 to 0.7303	No	ns	>0.9999
107	SPC -4 weeks post winter:4 weeks of winter vs. SPC -8 weeks post winter:8 weeks of winter	-0.4803	-1.446 to 0.4857	No	ns	0.9120
108	SPC -4 weeks post winter:8 weeks of winter vs. SPC -8 weeks post winter:2 weeks of winter	0.1529	-0.8131 to 1.119	No	ns	>0.9999
109	SPC -4 weeks post winter:8 weeks of winter vs. SPC -8 weeks post winter:4 weeks of winter	0	-0.966 to 0.966	No	ns	>0.9999
110	SPC -4 weeks post winter:8 weeks of winter vs. SPC -8 weeks post winter:8 weeks of winter	-0.2445	-1.211 to 0.7214	No	ns	0.9999
111	SPC -8 weeks post winter:2 weeks of winter vs. SPC -8 weeks post winter:4 weeks of winter	-0.1529	-1.119 to 0.8131	No	ns	>0.9999
112	SPC -8 weeks post winter:2 weeks of winter vs. SPC -8 weeks post winter:8 weeks of winter	-0.3974	-1.363 to 0.5686	No	ns	0.9803
113	SPC -8 weeks post winter:4 weeks of winter vs. SPC -8 weeks post winter:8 weeks of winter	-0.2445	-1.211 to 0.7214	No	ns	0.9999
114						
115						
116	Test details	Mean 1	Mean 2	Mean Diff.	SE of diff.	N1
117						
118	End of winter:2 weeks of winter vs. End of winter:4 weeks of winter	0.7202	0.8032	-0.08302	0.2754	6
119	End of winter:2 weeks of winter vs. End of winter:8 weeks of winter	0.7202	0.7511	-0.03097	0.2754	6
120	End of winter:2 weeks of winter vs. 4 weeks post winter:2 weeks of winter	0.7202	1.175	-0.4552	0.2754	6

2way ANOVA<br>Multiple comparisons						
121	End of winter:2 weeks of winter vs. 4 weeks post winter:4 weeks of winter	0.7202	1.386	-0.6661	0.2754	6
122	End of winter:2 weeks of winter vs. 4 weeks post winter:8 weeks of winter	0.7202	2.233	-1.513	0.2754	6
123	End of winter:2 weeks of winter vs. 8 weeks post winter:2 weeks of winter	0.7202	1.488	-0.7676	0.2754	6
124	End of winter:2 weeks of winter vs. 8 weeks post winter:4 weeks of winter	0.7202	2.973	-2.253	0.2754	6
125	End of winter:2 weeks of winter vs. 8 weeks post winter:8 weeks of winter	0.7202	3.158	-2.438	0.2754	6
126	End of winter:2 weeks of winter vs. SPC -4 weeks post winter:2 weeks of winter	0.7202	0.6261	0.09402	0.2754	6
127	End of winter:2 weeks of winter vs. SPC -4 weeks post winter:4 weeks of winter	0.7202	0.7511	-0.03097	0.2754	6
128	End of winter:2 weeks of winter vs. SPC -4 weeks post winter:8 weeks of winter	0.7202	0.9868	-0.2667	0.2754	6
129	End of winter:2 weeks of winter vs. SPC -8 weeks post winter:2 weeks of winter	0.7202	0.834	-0.1138	0.2754	6
130	End of winter:2 weeks of winter vs. SPC -8 weeks post winter:4 weeks of winter	0.7202	0.9868	-0.2667	0.2754	6
131	End of winter:2 weeks of winter vs. SPC -8 weeks post winter:8 weeks of winter	0.7202	1.231	-0.5112	0.2754	6
132	End of winter:4 weeks of winter vs. End of winter:8 weeks of winter	0.8032	0.7511	0.05205	0.2754	6
133	End of winter:4 weeks of winter vs. 4 weeks post winter:2 weeks of winter	0.8032	1.175	-0.3722	0.2754	6
134	End of winter:4 weeks of winter vs. 4 weeks post winter:4 weeks of winter	0.8032	1.386	-0.5831	0.2754	6
135	End of winter:4 weeks of winter vs. 4 weeks post winter:8 weeks of winter	0.8032	2.233	-1.43	0.2754	6
136	End of winter:4 weeks of winter vs. 8 weeks post winter:2 weeks of winter	0.8032	1.488	-0.6846	0.2754	6
137	End of winter:4 weeks of winter vs. 8 weeks post winter:4 weeks of winter	0.8032	2.973	-2.17	0.2754	6
138	End of winter:4 weeks of winter vs. 8 weeks post winter:8 weeks of winter	0.8032	3.158	-2.355	0.2754	6
139	End of winter:4 weeks of winter vs. SPC -4 weeks post winter:2 weeks of winter	0.8032	0.6261	0.177	0.2754	6
140	End of winter:4 weeks of winter vs. SPC -4 weeks post winter:4 weeks of winter	0.8032	0.7511	0.05205	0.2754	6
141	End of winter:4 weeks of winter vs. SPC -4 weeks post winter:8 weeks of winter	0.8032	0.9868	-0.1837	0.2754	6
142	End of winter:4 weeks of winter vs. SPC -8 weeks post winter:2 weeks of winter	0.8032	0.834	-0.03078	0.2754	6
143	End of winter:4 weeks of winter vs. SPC -8 weeks post winter:4 weeks of winter	0.8032	0.9868	-0.1837	0.2754	6
144	End of winter:4 weeks of winter vs. SPC -8 weeks post winter:8 weeks of winter	0.8032	1.231	-0.4282	0.2754	6
145	End of winter:8 weeks of winter vs. 4 weeks post winter:2 weeks of winter	0.7511	1.175	-0.4243	0.2754	6
146	End of winter:8 weeks of winter vs. 4 weeks post winter:4 weeks of winter	0.7511	1.386	-0.6352	0.2754	6
147	End of winter:8 weeks of winter vs. 4 weeks post winter:8 weeks of winter	0.7511	2.233	-1.482	0.2754	6
148	End of winter:8 weeks of winter vs. 8 weeks post winter:2 weeks of winter	0.7511	1.488	-0.7366	0.2754	6
149	End of winter:8 weeks of winter vs. 8 weeks post winter:4 weeks of winter	0.7511	2.973	-2.222	0.2754	6
150	End of winter:8 weeks of winter vs. 8 weeks post winter:8 weeks of winter	0.7511	3.158	-2.407	0.2754	6

2way ANOVA<br>Multiple comparisons						
151	End of winter:8 weeks of winter vs. SPC -4 weeks post winter:2 weeks of winter	0.7511	0.6261	0.125	0.2754	6
152	End of winter:8 weeks of winter vs. SPC -4 weeks post winter:4 weeks of winter	0.7511	0.7511	0	0.2754	6
153	End of winter:8 weeks of winter vs. SPC -4 weeks post winter:8 weeks of winter	0.7511	0.9868	-0.2357	0.2754	6
154	End of winter:8 weeks of winter vs. SPC -8 weeks post winter:2 weeks of winter	0.7511	0.834	-0.08283	0.2754	6
155	End of winter:8 weeks of winter vs. SPC -8 weeks post winter:4 weeks of winter	0.7511	0.9868	-0.2357	0.2754	6
156	End of winter:8 weeks of winter vs. SPC -8 weeks post winter:8 weeks of winter	0.7511	1.231	-0.4803	0.2754	6
157	4 weeks post winter:2 weeks of winter vs. 4 weeks post winter:4 weeks of winter	1.175	1.386	-0.2109	0.2754	6
158	4 weeks post winter:2 weeks of winter vs. 4 weeks post winter:8 weeks of winter	1.175	2.233	-1.058	0.2754	6
159	4 weeks post winter:2 weeks of winter vs. 8 weeks post winter:2 weeks of winter	1.175	1.488	-0.3124	0.2754	6
160	4 weeks post winter:2 weeks of winter vs. 8 weeks post winter:4 weeks of winter	1.175	2.973	-1.797	0.2754	6
161	4 weeks post winter:2 weeks of winter vs. 8 weeks post winter:8 weeks of winter	1.175	3.158	-1.983	0.2754	6
162	4 weeks post winter:2 weeks of winter vs. SPC -4 weeks post winter:2 weeks of winter	1.175	0.6261	0.5492	0.2754	6
163	4 weeks post winter:2 weeks of winter vs. SPC -4 weeks post winter:4 weeks of winter	1.175	0.7511	0.4243	0.2754	6
164	4 weeks post winter:2 weeks of winter vs. SPC -4 weeks post winter:8 weeks of winter	1.175	0.9868	0.1885	0.2754	6
165	4 weeks post winter:2 weeks of winter vs. SPC -8 weeks post winter:2 weeks of winter	1.175	0.834	0.3414	0.2754	6
166	4 weeks post winter:2 weeks of winter vs. SPC -8 weeks post winter:4 weeks of winter	1.175	0.9868	0.1885	0.2754	6
167	4 weeks post winter:2 weeks of winter vs. SPC -8 weeks post winter:8 weeks of winter	1.175	1.231	-0.05601	0.2754	6
168	4 weeks post winter:4 weeks of winter vs. 4 weeks post winter:8 weeks of winter	1.386	2.233	-0.847	0.2754	6
169	4 weeks post winter:4 weeks of winter vs. 8 weeks post winter:2 weeks of winter	1.386	1.488	-0.1014	0.2754	6
170	4 weeks post winter:4 weeks of winter vs. 8 weeks post winter:4 weeks of winter	1.386	2.973	-1.586	0.2754	6
171	4 weeks post winter:4 weeks of winter vs. 8 weeks post winter:8 weeks of winter	1.386	3.158	-1.772	0.2754	6
172	4 weeks post winter:4 weeks of winter vs. SPC -4 weeks post winter:2 weeks of winter	1.386	0.6261	0.7601	0.2754	6
173	4 weeks post winter:4 weeks of winter vs. SPC -4 weeks post winter:4 weeks of winter	1.386	0.7511	0.6352	0.2754	6
174	4 weeks post winter:4 weeks of winter vs. SPC -4 weeks post winter:8 weeks of winter	1.386	0.9868	0.3994	0.2754	6
175	4 weeks post winter:4 weeks of winter vs. SPC -8 weeks post winter:2 weeks of winter	1.386	0.834	0.5523	0.2754	6
176	4 weeks post winter:4 weeks of winter vs. SPC -8 weeks post winter:4 weeks of winter	1.386	0.9868	0.3994	0.2754	6
177	4 weeks post winter:4 weeks of winter vs. SPC -8 weeks post winter:8 weeks of winter	1.386	1.231	0.1549	0.2754	6
178	4 weeks post winter:8 weeks of winter vs. 8 weeks post winter:2 weeks of winter	2.233	1.488	0.7455	0.2754	6
179	4 weeks post winter:8 weeks of winter vs. 8 weeks post winter:4 weeks of winter	2.233	2.973	-0.7395	0.2754	6
180	4 weeks post winter:8 weeks of winter vs. 8 weeks post winter:8 weeks of winter	2.233	3.158	-0.9248	0.2754	6

2way ANOVA<br>Multiple comparisons						
181	4 weeks post winter:8 weeks of winter vs. SPC -4 weeks post winter:2 weeks of winter	2.233	0.6261	1.607	0.2754	6
182	4 weeks post winter:8 weeks of winter vs. SPC -4 weeks post winter:4 weeks of winter	2.233	0.7511	1.482	0.2754	6
183	4 weeks post winter:8 weeks of winter vs. SPC -4 weeks post winter:8 weeks of winter	2.233	0.9868	1.246	0.2754	6
184	4 weeks post winter:8 weeks of winter vs. SPC -8 weeks post winter:2 weeks of winter	2.233	0.834	1.399	0.2754	6
185	4 weeks post winter:8 weeks of winter vs. SPC -8 weeks post winter:4 weeks of winter	2.233	0.9868	1.246	0.2754	6
186	4 weeks post winter:8 weeks of winter vs. SPC -8 weeks post winter:8 weeks of winter	2.233	1.231	1.002	0.2754	6
187	8 weeks post winter:2 weeks of winter vs. 8 weeks post winter:4 weeks of winter	1.488	2.973	-1.485	0.2754	6
188	8 weeks post winter:2 weeks of winter vs. 8 weeks post winter:8 weeks of winter	1.488	3.158	-1.67	0.2754	6
189	8 weeks post winter:2 weeks of winter vs. SPC -4 weeks post winter:2 weeks of winter	1.488	0.6261	0.8616	0.2754	6
190	8 weeks post winter:2 weeks of winter vs. SPC -4 weeks post winter:4 weeks of winter	1.488	0.7511	0.7366	0.2754	6
191	8 weeks post winter:2 weeks of winter vs. SPC -4 weeks post winter:8 weeks of winter	1.488	0.9868	0.5009	0.2754	6
192	8 weeks post winter:2 weeks of winter vs. SPC -8 weeks post winter:2 weeks of winter	1.488	0.834	0.6538	0.2754	6
193	8 weeks post winter:2 weeks of winter vs. SPC -8 weeks post winter:4 weeks of winter	1.488	0.9868	0.5009	0.2754	6
194	8 weeks post winter:2 weeks of winter vs. SPC -8 weeks post winter:8 weeks of winter	1.488	1.231	0.2563	0.2754	6
195	8 weeks post winter:4 weeks of winter vs. 8 weeks post winter:8 weeks of winter	2.973	3.158	-0.1854	0.2754	6
196	8 weeks post winter:4 weeks of winter vs. SPC -4 weeks post winter:2 weeks of winter	2.973	0.6261	2.347	0.2754	6
197	8 weeks post winter:4 weeks of winter vs. SPC -4 weeks post winter:4 weeks of winter	2.973	0.7511	2.222	0.2754	6
198	8 weeks post winter:4 weeks of winter vs. SPC -4 weeks post winter:8 weeks of winter	2.973	0.9868	1.986	0.2754	6
199	8 weeks post winter:4 weeks of winter vs. SPC -8 weeks post winter:2 weeks of winter	2.973	0.834	2.139	0.2754	6
200	8 weeks post winter:4 weeks of winter vs. SPC -8 weeks post winter:4 weeks of winter	2.973	0.9868	1.986	0.2754	6
201	8 weeks post winter:4 weeks of winter vs. SPC -8 weeks post winter:8 weeks of winter	2.973	1.231	1.741	0.2754	6
202	8 weeks post winter:8 weeks of winter vs. SPC -4 weeks post winter:2 weeks of winter	3.158	0.6261	2.532	0.2754	6
203	8 weeks post winter:8 weeks of winter vs. SPC -4 weeks post winter:4 weeks of winter	3.158	0.7511	2.407	0.2754	6
204	8 weeks post winter:8 weeks of winter vs. SPC -4 weeks post winter:8 weeks of winter	3.158	0.9868	2.171	0.2754	6
205	8 weeks post winter:8 weeks of winter vs. SPC -8 weeks post winter:2 weeks of winter	3.158	0.834	2.324	0.2754	6
206	8 weeks post winter:8 weeks of winter vs. SPC -8 weeks post winter:4 weeks of winter	3.158	0.9868	2.171	0.2754	6
207	8 weeks post winter:8 weeks of winter vs. SPC -8 weeks post winter:8 weeks of winter	3.158	1.231	1.927	0.2754	6
208	SPC -4 weeks post winter:2 weeks of winter vs. SPC -4 weeks post winter:4 weeks of winter	0.6261	0.7511	-0.125	0.2754	6
209	SPC -4 weeks post winter:2 weeks of winter vs. SPC -4 weeks post winter:8 weeks of winter	0.6261	0.9868	-0.3607	0.2754	6
210	SPC -4 weeks post winter:2 weeks of winter vs. SPC -8 weeks post winter:2 weeks of winter	0.6261	0.834	-0.2078	0.2754	6

2way ANOVA<br>Multiple comparisons						
211	SPC -4 weeks post winter:2 weeks of winter vs. SPC -8 weeks post winter:4 weeks of winter	0.6261	0.9868	-0.3607	0.2754	6
212	SPC -4 weeks post winter:2 weeks of winter vs. SPC -8 weeks post winter:8 weeks of winter	0.6261	1.231	-0.6053	0.2754	6
213	SPC -4 weeks post winter:4 weeks of winter vs. SPC -4 weeks post winter:8 weeks of winter	0.7511	0.9868	-0.2357	0.2754	6
214	SPC -4 weeks post winter:4 weeks of winter vs. SPC -8 weeks post winter:2 weeks of winter	0.7511	0.834	-0.08283	0.2754	6
215	SPC -4 weeks post winter:4 weeks of winter vs. SPC -8 weeks post winter:4 weeks of winter	0.7511	0.9868	-0.2357	0.2754	6
216	SPC -4 weeks post winter:4 weeks of winter vs. SPC -8 weeks post winter:8 weeks of winter	0.7511	1.231	-0.4803	0.2754	6
217	SPC -4 weeks post winter:8 weeks of winter vs. SPC -8 weeks post winter:2 weeks of winter	0.9868	0.834	0.1529	0.2754	6
218	SPC -4 weeks post winter:8 weeks of winter vs. SPC -8 weeks post winter:4 weeks of winter	0.9868	0.9868	0	0.2754	6
219	SPC -4 weeks post winter:8 weeks of winter vs. SPC -8 weeks post winter:8 weeks of winter	0.9868	1.231	-0.2445	0.2754	6
220	SPC -8 weeks post winter:2 weeks of winter vs. SPC -8 weeks post winter:4 weeks of winter	0.834	0.9868	-0.1529	0.2754	6
221	SPC -8 weeks post winter:2 weeks of winter vs. SPC -8 weeks post winter:8 weeks of winter	0.834	1.231	-0.3974	0.2754	6
222	SPC -8 weeks post winter:4 weeks of winter vs. SPC -8 weeks post winter:8 weeks of winter	0.9868	1.231	-0.2445	0.2754	6

1			
2			
3			
4			
5			
6			
7			
8			
9			
10			
11			
12			
13			
14			
15			
16			
17			
18			
19			
20			
21			
22			
23			
24			
25			
26			
27			
28			
29			
30			

31			
32			
33			
34			
35			
36			
37			
38			
39			
40			
41			
42			
43			
44			
45			
46			
47			
48			
49			
50			
51			
52			
53			
54			
55			
56			
57			
58			
59			
60			

61			
62			
63			
64			
65			
66			
67			
68			
69			
70			
71			
72			
73			
74			
75			
76			
77			
78			
79			
80			
81			
82			
83			
84			
85			
86			
87			
88			
89			
90			

91			
92			
93			
94			
95			
96			
97			
98			
99			
100			
101			
102			
103			
104			
105			
106			
107			
108			
109			
110			
111			
112			
113			
114			
115			
116	N2	q	DF
117			
118	6	0.4262	75
119	6	0.159	75
120	6	2.337	75

**121**	6	3.42	75
**122**	6	7.769	75
**123**	6	3.941	75
**124**	6	11.57	75
**125**	6	12.52	75
**126**	6	0.4827	75
**127**	6	0.159	75
**128**	6	1.369	75
**129**	6	0.5843	75
**130**	6	1.369	75
**131**	6	2.625	75
**132**	6	0.2672	75
**133**	6	1.911	75
**134**	6	2.994	75
**135**	6	7.342	75
**136**	6	3.515	75
**137**	6	11.14	75
**138**	6	12.09	75
**139**	6	0.909	75
**140**	6	0.2672	75
**141**	6	0.943	75
**142**	6	0.158	75
**143**	6	0.943	75
**144**	6	2.199	75
**145**	6	2.178	75
**146**	6	3.261	75
**147**	6	7.61	75
**148**	6	3.782	75
**149**	6	11.41	75
**150**	6	12.36	75

**151**	6	0.6417	75
**152**	6	0	75
**153**	6	1.21	75
**154**	6	0.4253	75
**155**	6	1.21	75
**156**	6	2.466	75
**157**	6	1.083	75
**158**	6	5.431	75
**159**	6	1.604	75
**160**	6	9.228	75
**161**	6	10.18	75
**162**	6	2.82	75
**163**	6	2.178	75
**164**	6	0.968	75
**165**	6	1.753	75
**166**	6	0.968	75
**167**	6	0.2876	75
**168**	6	4.349	75
**169**	6	0.5209	75
**170**	6	8.145	75
**171**	6	9.097	75
**172**	6	3.903	75
**173**	6	3.261	75
**174**	6	2.051	75
**175**	6	2.836	75
**176**	6	2.051	75
**177**	6	0.7953	75
**178**	6	3.828	75
**179**	6	3.797	75
**180**	6	4.748	75

**181**	6	8.251	75
**182**	6	7.61	75
**183**	6	6.399	75
**184**	6	7.184	75
**185**	6	6.399	75
**186**	6	5.144	75
**187**	6	7.624	75
**188**	6	8.576	75
**189**	6	4.424	75
**190**	6	3.782	75
**191**	6	2.572	75
**192**	6	3.357	75
**193**	6	2.572	75
**194**	6	1.316	75
**195**	6	0.9517	75
**196**	6	12.05	75
**197**	6	11.41	75
**198**	6	10.2	75
**199**	6	10.98	75
**200**	6	10.2	75
**201**	6	8.94	75
**202**	6	13	75
**203**	6	12.36	75
**204**	6	11.15	75
**205**	6	11.93	75
**206**	6	11.15	75
**207**	6	9.892	75
**208**	6	0.6417	75
**209**	6	1.852	75
**210**	6	1.067	75

**211**	6	1.852	75
**212**	6	3.108	75
**213**	6	1.21	75
**214**	6	0.4253	75
**215**	6	1.21	75
**216**	6	2.466	75
**217**	6	0.785	75
**218**	6	0	75
**219**	6	1.256	75
**220**	6	0.785	75
**221**	6	2.041	75
**222**	6	1.256	75

2way ANOVA
Tabular results

1	Table Analyzed	TPH				
2						
3	Two-way ANOVA	Ordinary				
4	Alpha	0.05				
5						
6	Source of Variation	% of total variation	P value	P value summary	Significant?	
7	Interaction	30.55	<0.0001	****	Yes	
8	Time post-winter	21.16	<0.0001	****	Yes	
9	Treatment	11.8	<0.0001	****	Yes	
10						
11	ANOVA table	SS	DF	MS	F (DFn, DFd)	P value
12	Interaction	67.73	8	8.466	F (8, 75) = 7.849	P<0.0001
13	Time post-winter	46.91	4	11.73	F (4, 75) = 10.87	P<0.0001
14	Treatment	26.16	2	13.08	F (2, 75) = 12.13	P<0.0001
15	Residual	80.89	75	1.079		
16						
17	Number of missing values	0				

**2way ANOVA** Multiple comparisons						
1	Compare cell means regardless of rows and columns					
2						
3	Number of families	1				
4	Number of comparisons per family	105				
5	Alpha	0.05				
6						
7	Tukey's multiple comparisons test	Mean Diff.	95.00% CI of diff.	Significant?	Summary	Adjusted P Value
8						
9	End of winter:2 weeks of winter vs. End of winter:4 weeks of winter	-0.06621	-2.169 to 2.037	No	ns	>0.9999
10	End of winter:2 weeks of winter vs. End of winter:8 weeks of winter	-0.3664	-2.469 to 1.736	No	ns	>0.9999
11	End of winter:2 weeks of winter vs. 4 weeks post winter:2 weeks of winter	-0.6253	-2.728 to 1.477	No	ns	0.9992
12	End of winter:2 weeks of winter vs. 4 weeks post winter:4 weeks of winter	-0.5264	-2.629 to 1.576	No	ns	0.9999
13	End of winter:2 weeks of winter vs. 4 weeks post winter:8 weeks of winter	-0.495	-2.598 to 1.608	No	ns	>0.9999
14	End of winter:2 weeks of winter vs. 8 weeks post winter:2 weeks of winter	-0.2547	-2.358 to 1.848	No	ns	>0.9999
15	End of winter:2 weeks of winter vs. 8 weeks post winter:4 weeks of winter	-0.8466	-2.949 to 1.256	No	ns	0.9837
16	End of winter:2 weeks of winter vs. 8 weeks post winter:8 weeks of winter	-5.325	-7.427 to -3.222	Yes	****	<0.0001
17	End of winter:2 weeks of winter vs. SPC -4 weeks post winter:2 weeks of winter	-0.05656	-2.159 to 2.046	No	ns	>0.9999
18	End of winter:2 weeks of winter vs. SPC -4 weeks post winter:4 weeks of winter	-0.3664	-2.469 to 1.736	No	ns	>0.9999
19	End of winter:2 weeks of winter vs. SPC -4 weeks post winter:8 weeks of winter	-0.467	-2.57 to 1.636	No	ns	>0.9999
20	End of winter:2 weeks of winter vs. SPC -8 weeks post winter:2 weeks of winter	-0.307	-2.41 to 1.796	No	ns	>0.9999
21	End of winter:2 weeks of winter vs. SPC -8 weeks post winter:4 weeks of winter	-0.467	-2.57 to 1.636	No	ns	>0.9999
22	End of winter:2 weeks of winter vs. SPC -8 weeks post winter:8 weeks of winter	-0.7541	-2.857 to 1.349	No	ns	0.9945
23	End of winter:4 weeks of winter vs. End of winter:8 weeks of winter	-0.3002	-2.403 to 1.803	No	ns	>0.9999
24	End of winter:4 weeks of winter vs. 4 weeks post winter:2 weeks of winter	-0.5591	-2.662 to 1.544	No	ns	0.9998
25	End of winter:4 weeks of winter vs. 4 weeks post winter:4 weeks of winter	-0.4601	-2.563 to 1.643	No	ns	>0.9999
26	End of winter:4 weeks of winter vs. 4 weeks post winter:8 weeks of winter	-0.4288	-2.532 to 1.674	No	ns	>0.9999
27	End of winter:4 weeks of winter vs. 8 weeks post winter:2 weeks of winter	-0.1885	-2.291 to 1.914	No	ns	>0.9999
28	End of winter:4 weeks of winter vs. 8 weeks post winter:4 weeks of winter	-0.7804	-2.883 to 1.322	No	ns	0.9923
29	End of winter:4 weeks of winter vs. 8 weeks post winter:8 weeks of winter	-5.258	-7.361 to -3.156	Yes	****	<0.0001
30	End of winter:4 weeks of winter vs. SPC -4 weeks post winter:2 weeks of winter	0.00965	-2.093 to 2.112	No	ns	>0.9999

2way ANOVA<br>Multiple comparisons						
31	End of winter:4 weeks of winter vs. SPC -4 weeks post winter:4 weeks of winter	-0.3002	-2.403 to 1.803	No	ns	>0.9999
32	End of winter:4 weeks of winter vs. SPC -4 weeks post winter:8 weeks of winter	-0.4008	-2.504 to 1.702	No	ns	>0.9999
33	End of winter:4 weeks of winter vs. SPC -8 weeks post winter:2 weeks of winter	-0.2408	-2.344 to 1.862	No	ns	>0.9999
34	End of winter:4 weeks of winter vs. SPC -8 weeks post winter:4 weeks of winter	-0.4008	-2.504 to 1.702	No	ns	>0.9999
35	End of winter:4 weeks of winter vs. SPC -8 weeks post winter:8 weeks of winter	-0.6879	-2.791 to 1.415	No	ns	0.9978
36	End of winter:8 weeks of winter vs. 4 weeks post winter:2 weeks of winter	-0.2589	-2.362 to 1.844	No	ns	>0.9999
37	End of winter:8 weeks of winter vs. 4 weeks post winter:4 weeks of winter	-0.16	-2.263 to 1.943	No	ns	>0.9999
38	End of winter:8 weeks of winter vs. 4 weeks post winter:8 weeks of winter	-0.1286	-2.231 to 1.974	No	ns	>0.9999
39	End of winter:8 weeks of winter vs. 8 weeks post winter:2 weeks of winter	0.1117	-1.991 to 2.214	No	ns	>0.9999
40	End of winter:8 weeks of winter vs. 8 weeks post winter:4 weeks of winter	-0.4802	-2.583 to 1.623	No	ns	>0.9999
41	End of winter:8 weeks of winter vs. 8 weeks post winter:8 weeks of winter	-4.958	-7.061 to -2.855	Yes	****	<0.0001
42	End of winter:8 weeks of winter vs. SPC -4 weeks post winter:2 weeks of winter	0.3098	-1.793 to 2.413	No	ns	>0.9999
43	End of winter:8 weeks of winter vs. SPC -4 weeks post winter:4 weeks of winter	0	-2.103 to 2.103	No	ns	>0.9999
44	End of winter:8 weeks of winter vs. SPC -4 weeks post winter:8 weeks of winter	-0.1006	-2.203 to 2.002	No	ns	>0.9999
45	End of winter:8 weeks of winter vs. SPC -8 weeks post winter:2 weeks of winter	0.05941	-2.043 to 2.162	No	ns	>0.9999
46	End of winter:8 weeks of winter vs. SPC -8 weeks post winter:4 weeks of winter	-0.1006	-2.203 to 2.002	No	ns	>0.9999
47	End of winter:8 weeks of winter vs. SPC -8 weeks post winter:8 weeks of winter	-0.3877	-2.491 to 1.715	No	ns	>0.9999
48	4 weeks post winter:2 weeks of winter vs. 4 weeks post winter:4 weeks of winter	0.09897	-2.004 to 2.202	No	ns	>0.9999
49	4 weeks post winter:2 weeks of winter vs. 4 weeks post winter:8 weeks of winter	0.1303	-1.973 to 2.233	No	ns	>0.9999
50	4 weeks post winter:2 weeks of winter vs. 8 weeks post winter:2 weeks of winter	0.3706	-1.732 to 2.473	No	ns	>0.9999
51	4 weeks post winter:2 weeks of winter vs. 8 weeks post winter:4 weeks of winter	-0.2212	-2.324 to 1.882	No	ns	>0.9999
52	4 weeks post winter:2 weeks of winter vs. 8 weeks post winter:8 weeks of winter	-4.699	-6.802 to -2.596	Yes	****	<0.0001
53	4 weeks post winter:2 weeks of winter vs. SPC -4 weeks post winter:2 weeks of winter	0.5688	-1.534 to 2.672	No	ns	0.9997
54	4 weeks post winter:2 weeks of winter vs. SPC -4 weeks post winter:4 weeks of winter	0.2589	-1.844 to 2.362	No	ns	>0.9999
55	4 weeks post winter:2 weeks of winter vs. SPC -4 weeks post winter:8 weeks of winter	0.1583	-1.945 to 2.261	No	ns	>0.9999
56	4 weeks post winter:2 weeks of winter vs. SPC -8 weeks post winter:2 weeks of winter	0.3184	-1.784 to 2.421	No	ns	>0.9999
57	4 weeks post winter:2 weeks of winter vs. SPC -8 weeks post winter:4 weeks of winter	0.1583	-1.945 to 2.261	No	ns	>0.9999
58	4 weeks post winter:2 weeks of winter vs. SPC -8 weeks post winter:8 weeks of winter	-0.1287	-2.232 to 1.974	No	ns	>0.9999
59	4 weeks post winter:4 weeks of winter vs. 4 weeks post winter:8 weeks of winter	0.03135	-2.071 to 2.134	No	ns	>0.9999
60	4 weeks post winter:4 weeks of winter vs. 8 weeks post winter:2 weeks of winter	0.2716	-1.831 to 2.374	No	ns	>0.9999

2way ANOVA<br>Multiple comparisons						
61	4 weeks post winter:4 weeks of winter vs. 8 weeks post winter:4 weeks of winter	-0.3202	-2.423 to 1.783	No	ns	>0.9999
62	4 weeks post winter:4 weeks of winter vs. 8 weeks post winter:8 weeks of winter	-4.798	-6.901 to -2.695	Yes	****	<0.0001
63	4 weeks post winter:4 weeks of winter vs. SPC -4 weeks post winter:2 weeks of winter	0.4698	-1.633 to 2.573	No	ns	>0.9999
64	4 weeks post winter:4 weeks of winter vs. SPC -4 weeks post winter:4 weeks of winter	0.16	-1.943 to 2.263	No	ns	>0.9999
65	4 weeks post winter:4 weeks of winter vs. SPC -4 weeks post winter:8 weeks of winter	0.05934	-2.043 to 2.162	No	ns	>0.9999
66	4 weeks post winter:4 weeks of winter vs. SPC -8 weeks post winter:2 weeks of winter	0.2194	-1.883 to 2.322	No	ns	>0.9999
67	4 weeks post winter:4 weeks of winter vs. SPC -8 weeks post winter:4 weeks of winter	0.05934	-2.043 to 2.162	No	ns	>0.9999
68	4 weeks post winter:4 weeks of winter vs. SPC -8 weeks post winter:8 weeks of winter	-0.2277	-2.331 to 1.875	No	ns	>0.9999
69	4 weeks post winter:8 weeks of winter vs. 8 weeks post winter:2 weeks of winter	0.2403	-1.863 to 2.343	No	ns	>0.9999
70	4 weeks post winter:8 weeks of winter vs. 8 weeks post winter:4 weeks of winter	-0.3516	-2.454 to 1.751	No	ns	>0.9999
71	4 weeks post winter:8 weeks of winter vs. 8 weeks post winter:8 weeks of winter	-4.83	-6.932 to -2.727	Yes	****	<0.0001
72	4 weeks post winter:8 weeks of winter vs. SPC -4 weeks post winter:2 weeks of winter	0.4384	-1.664 to 2.541	No	ns	>0.9999
73	4 weeks post winter:8 weeks of winter vs. SPC -4 weeks post winter:4 weeks of winter	0.1286	-1.974 to 2.231	No	ns	>0.9999
74	4 weeks post winter:8 weeks of winter vs. SPC -4 weeks post winter:8 weeks of winter	0.02799	-2.075 to 2.131	No	ns	>0.9999
75	4 weeks post winter:8 weeks of winter vs. SPC -8 weeks post winter:2 weeks of winter	0.188	-1.915 to 2.291	No	ns	>0.9999
76	4 weeks post winter:8 weeks of winter vs. SPC -8 weeks post winter:4 weeks of winter	0.02799	-2.075 to 2.131	No	ns	>0.9999
77	4 weeks post winter:8 weeks of winter vs. SPC -8 weeks post winter:8 weeks of winter	-0.2591	-2.362 to 1.844	No	ns	>0.9999
78	8 weeks post winter:2 weeks of winter vs. 8 weeks post winter:4 weeks of winter	-0.5919	-2.695 to 1.511	No	ns	0.9996
79	8 weeks post winter:2 weeks of winter vs. 8 weeks post winter:8 weeks of winter	-5.07	-7.173 to -2.967	Yes	****	<0.0001
80	8 weeks post winter:2 weeks of winter vs. SPC -4 weeks post winter:2 weeks of winter	0.1982	-1.905 to 2.301	No	ns	>0.9999
81	8 weeks post winter:2 weeks of winter vs. SPC -4 weeks post winter:4 weeks of winter	-0.1117	-2.214 to 1.991	No	ns	>0.9999
82	8 weeks post winter:2 weeks of winter vs. SPC -4 weeks post winter:8 weeks of winter	-0.2123	-2.315 to 1.891	No	ns	>0.9999
83	8 weeks post winter:2 weeks of winter vs. SPC -8 weeks post winter:2 weeks of winter	-0.05225	-2.155 to 2.051	No	ns	>0.9999
84	8 weeks post winter:2 weeks of winter vs. SPC -8 weeks post winter:4 weeks of winter	-0.2123	-2.315 to 1.891	No	ns	>0.9999
85	8 weeks post winter:2 weeks of winter vs. SPC -8 weeks post winter:8 weeks of winter	-0.4993	-2.602 to 1.603	No	ns	>0.9999
86	8 weeks post winter:4 weeks of winter vs. 8 weeks post winter:8 weeks of winter	-4.478	-6.581 to -2.375	Yes	****	<0.0001
87	8 weeks post winter:4 weeks of winter vs. SPC -4 weeks post winter:2 weeks of winter	0.79	-1.313 to 2.893	No	ns	0.9914
88	8 weeks post winter:4 weeks of winter vs. SPC -4 weeks post winter:4 weeks of winter	0.4802	-1.623 to 2.583	No	ns	>0.9999
89	8 weeks post winter:4 weeks of winter vs. SPC -4 weeks post winter:8 weeks of winter	0.3796	-1.723 to 2.482	No	ns	>0.9999
90	8 weeks post winter:4 weeks of winter vs. SPC -8 weeks post winter:2 weeks of winter	0.5396	-1.563 to 2.642	No	ns	0.9999

	**2way ANOVA**<br>Multiple comparisons					
91	8 weeks post winter:4 weeks of winter vs. SPC -8 weeks post winter:4 weeks of winter	0.3796	-1.723 to 2.482	No	ns	>0.9999
92	8 weeks post winter:4 weeks of winter vs. SPC -8 weeks post winter:8 weeks of winter	0.09251	-2.01 to 2.195	No	ns	>0.9999
93	8 weeks post winter:8 weeks of winter vs. SPC -4 weeks post winter:2 weeks of winter	5.268	3.165 to 7.371	Yes	****	<0.0001
94	8 weeks post winter:8 weeks of winter vs. SPC -4 weeks post winter:4 weeks of winter	4.958	2.855 to 7.061	Yes	****	<0.0001
95	8 weeks post winter:8 weeks of winter vs. SPC -4 weeks post winter:8 weeks of winter	4.858	2.755 to 6.96	Yes	****	<0.0001
96	8 weeks post winter:8 weeks of winter vs. SPC -8 weeks post winter:2 weeks of winter	5.018	2.915 to 7.12	Yes	****	<0.0001
97	8 weeks post winter:8 weeks of winter vs. SPC -8 weeks post winter:4 weeks of winter	4.858	2.755 to 6.96	Yes	****	<0.0001
98	8 weeks post winter:8 weeks of winter vs. SPC -8 weeks post winter:8 weeks of winter	4.571	2.468 to 6.673	Yes	****	<0.0001
99	SPC -4 weeks post winter:2 weeks of winter vs. SPC -4 weeks post winter:4 weeks of winter	-0.3098	-2.413 to 1.793	No	ns	>0.9999
100	SPC -4 weeks post winter:2 weeks of winter vs. SPC -4 weeks post winter:8 weeks of winter	-0.4105	-2.513 to 1.692	No	ns	>0.9999
101	SPC -4 weeks post winter:2 weeks of winter vs. SPC -8 weeks post winter:2 weeks of winter	-0.2504	-2.353 to 1.852	No	ns	>0.9999
102	SPC -4 weeks post winter:2 weeks of winter vs. SPC -8 weeks post winter:4 weeks of winter	-0.4105	-2.513 to 1.692	No	ns	>0.9999
103	SPC -4 weeks post winter:2 weeks of winter vs. SPC -8 weeks post winter:8 weeks of winter	-0.6975	-2.8 to 1.405	No	ns	0.9975
104	SPC -4 weeks post winter:4 weeks of winter vs. SPC -4 weeks post winter:8 weeks of winter	-0.1006	-2.203 to 2.002	No	ns	>0.9999
105	SPC -4 weeks post winter:4 weeks of winter vs. SPC -8 weeks post winter:2 weeks of winter	0.05941	-2.043 to 2.162	No	ns	>0.9999
106	SPC -4 weeks post winter:4 weeks of winter vs. SPC -8 weeks post winter:4 weeks of winter	-0.1006	-2.203 to 2.002	No	ns	>0.9999
107	SPC -4 weeks post winter:4 weeks of winter vs. SPC -8 weeks post winter:8 weeks of winter	-0.3877	-2.491 to 1.715	No	ns	>0.9999
108	SPC -4 weeks post winter:8 weeks of winter vs. SPC -8 weeks post winter:2 weeks of winter	0.16	-1.943 to 2.263	No	ns	>0.9999
109	SPC -4 weeks post winter:8 weeks of winter vs. SPC -8 weeks post winter:4 weeks of winter	0	-2.103 to 2.103	No	ns	>0.9999
110	SPC -4 weeks post winter:8 weeks of winter vs. SPC -8 weeks post winter:8 weeks of winter	-0.287	-2.39 to 1.816	No	ns	>0.9999
111	SPC -8 weeks post winter:2 weeks of winter vs. SPC -8 weeks post winter:4 weeks of winter	-0.16	-2.263 to 1.943	No	ns	>0.9999
112	SPC -8 weeks post winter:2 weeks of winter vs. SPC -8 weeks post winter:8 weeks of winter	-0.4471	-2.55 to 1.656	No	ns	>0.9999
113	SPC -8 weeks post winter:4 weeks of winter vs. SPC -8 weeks post winter:8 weeks of winter	-0.287	-2.39 to 1.816	No	ns	>0.9999
114						
115						
116	Test details	Mean 1	Mean 2	Mean Diff.	SE of diff.	N1
117						
118	End of winter:2 weeks of winter vs. End of winter:4 weeks of winter	0.7905	0.8567	-0.06621	0.5996	6
119	End of winter:2 weeks of winter vs. End of winter:8 weeks of winter	0.7905	1.157	-0.3664	0.5996	6
120	End of winter:2 weeks of winter vs. 4 weeks post winter:2 weeks of winter	0.7905	1.416	-0.6253	0.5996	6

2way ANOVA<br>Multiple comparisons						
121	End of winter:2 weeks of winter vs. 4 weeks post winter:4 weeks of winter	0.7905	1.317	-0.5264	0.5996	6
122	End of winter:2 weeks of winter vs. 4 weeks post winter:8 weeks of winter	0.7905	1.286	-0.495	0.5996	6
123	End of winter:2 weeks of winter vs. 8 weeks post winter:2 weeks of winter	0.7905	1.045	-0.2547	0.5996	6
124	End of winter:2 weeks of winter vs. 8 weeks post winter:4 weeks of winter	0.7905	1.637	-0.8466	0.5996	6
125	End of winter:2 weeks of winter vs. 8 weeks post winter:8 weeks of winter	0.7905	6.115	-5.325	0.5996	6
126	End of winter:2 weeks of winter vs. SPC -4 weeks post winter:2 weeks of winter	0.7905	0.8471	-0.05656	0.5996	6
127	End of winter:2 weeks of winter vs. SPC -4 weeks post winter:4 weeks of winter	0.7905	1.157	-0.3664	0.5996	6
128	End of winter:2 weeks of winter vs. SPC -4 weeks post winter:8 weeks of winter	0.7905	1.258	-0.467	0.5996	6
129	End of winter:2 weeks of winter vs. SPC -8 weeks post winter:2 weeks of winter	0.7905	1.097	-0.307	0.5996	6
130	End of winter:2 weeks of winter vs. SPC -8 weeks post winter:4 weeks of winter	0.7905	1.258	-0.467	0.5996	6
131	End of winter:2 weeks of winter vs. SPC -8 weeks post winter:8 weeks of winter	0.7905	1.545	-0.7541	0.5996	6
132	End of winter:4 weeks of winter vs. End of winter:8 weeks of winter	0.8567	1.157	-0.3002	0.5996	6
133	End of winter:4 weeks of winter vs. 4 weeks post winter:2 weeks of winter	0.8567	1.416	-0.5591	0.5996	6
134	End of winter:4 weeks of winter vs. 4 weeks post winter:4 weeks of winter	0.8567	1.317	-0.4601	0.5996	6
135	End of winter:4 weeks of winter vs. 4 weeks post winter:8 weeks of winter	0.8567	1.286	-0.4288	0.5996	6
136	End of winter:4 weeks of winter vs. 8 weeks post winter:2 weeks of winter	0.8567	1.045	-0.1885	0.5996	6
137	End of winter:4 weeks of winter vs. 8 weeks post winter:4 weeks of winter	0.8567	1.637	-0.7804	0.5996	6
138	End of winter:4 weeks of winter vs. 8 weeks post winter:8 weeks of winter	0.8567	6.115	-5.258	0.5996	6
139	End of winter:4 weeks of winter vs. SPC -4 weeks post winter:2 weeks of winter	0.8567	0.8471	0.00965	0.5996	6
140	End of winter:4 weeks of winter vs. SPC -4 weeks post winter:4 weeks of winter	0.8567	1.157	-0.3002	0.5996	6
141	End of winter:4 weeks of winter vs. SPC -4 weeks post winter:8 weeks of winter	0.8567	1.258	-0.4008	0.5996	6
142	End of winter:4 weeks of winter vs. SPC -8 weeks post winter:2 weeks of winter	0.8567	1.097	-0.2408	0.5996	6
143	End of winter:4 weeks of winter vs. SPC -8 weeks post winter:4 weeks of winter	0.8567	1.258	-0.4008	0.5996	6
144	End of winter:4 weeks of winter vs. SPC -8 weeks post winter:8 weeks of winter	0.8567	1.545	-0.6879	0.5996	6
145	End of winter:8 weeks of winter vs. 4 weeks post winter:2 weeks of winter	1.157	1.416	-0.2589	0.5996	6
146	End of winter:8 weeks of winter vs. 4 weeks post winter:4 weeks of winter	1.157	1.317	-0.16	0.5996	6
147	End of winter:8 weeks of winter vs. 4 weeks post winter:8 weeks of winter	1.157	1.286	-0.1286	0.5996	6
148	End of winter:8 weeks of winter vs. 8 weeks post winter:2 weeks of winter	1.157	1.045	0.1117	0.5996	6
149	End of winter:8 weeks of winter vs. 8 weeks post winter:4 weeks of winter	1.157	1.637	-0.4802	0.5996	6
150	End of winter:8 weeks of winter vs. 8 weeks post winter:8 weeks of winter	1.157	6.115	-4.958	0.5996	6

2way ANOVA
Multiple comparisons

151	End of winter:8 weeks of winter vs. SPC -4 weeks post winter:2 weeks of winter	1.157	0.8471	0.3098	0.5996	6
152	End of winter:8 weeks of winter vs. SPC -4 weeks post winter:4 weeks of winter	1.157	1.157	0	0.5996	6
153	End of winter:8 weeks of winter vs. SPC -4 weeks post winter:8 weeks of winter	1.157	1.258	-0.1006	0.5996	6
154	End of winter:8 weeks of winter vs. SPC -8 weeks post winter:2 weeks of winter	1.157	1.097	0.05941	0.5996	6
155	End of winter:8 weeks of winter vs. SPC -8 weeks post winter:4 weeks of winter	1.157	1.258	-0.1006	0.5996	6
156	End of winter:8 weeks of winter vs. SPC -8 weeks post winter:8 weeks of winter	1.157	1.545	-0.3877	0.5996	6
157	4 weeks post winter:2 weeks of winter vs. 4 weeks post winter:4 weeks of winter	1.416	1.317	0.09897	0.5996	6
158	4 weeks post winter:2 weeks of winter vs. 4 weeks post winter:8 weeks of winter	1.416	1.286	0.1303	0.5996	6
159	4 weeks post winter:2 weeks of winter vs. 8 weeks post winter:2 weeks of winter	1.416	1.045	0.3706	0.5996	6
160	4 weeks post winter:2 weeks of winter vs. 8 weeks post winter:4 weeks of winter	1.416	1.637	-0.2212	0.5996	6
161	4 weeks post winter:2 weeks of winter vs. 8 weeks post winter:8 weeks of winter	1.416	6.115	-4.699	0.5996	6
162	4 weeks post winter:2 weeks of winter vs. SPC -4 weeks post winter:2 weeks of winter	1.416	0.8471	0.5688	0.5996	6
163	4 weeks post winter:2 weeks of winter vs. SPC -4 weeks post winter:4 weeks of winter	1.416	1.157	0.2589	0.5996	6
164	4 weeks post winter:2 weeks of winter vs. SPC -4 weeks post winter:8 weeks of winter	1.416	1.258	0.1583	0.5996	6
165	4 weeks post winter:2 weeks of winter vs. SPC -8 weeks post winter:2 weeks of winter	1.416	1.097	0.3184	0.5996	6
166	4 weeks post winter:2 weeks of winter vs. SPC -8 weeks post winter:4 weeks of winter	1.416	1.258	0.1583	0.5996	6
167	4 weeks post winter:2 weeks of winter vs. SPC -8 weeks post winter:8 weeks of winter	1.416	1.545	-0.1287	0.5996	6
168	4 weeks post winter:4 weeks of winter vs. 4 weeks post winter:8 weeks of winter	1.317	1.286	0.03135	0.5996	6
169	4 weeks post winter:4 weeks of winter vs. 8 weeks post winter:2 weeks of winter	1.317	1.045	0.2716	0.5996	6
170	4 weeks post winter:4 weeks of winter vs. 8 weeks post winter:4 weeks of winter	1.317	1.637	-0.3202	0.5996	6
171	4 weeks post winter:4 weeks of winter vs. 8 weeks post winter:8 weeks of winter	1.317	6.115	-4.798	0.5996	6
172	4 weeks post winter:4 weeks of winter vs. SPC -4 weeks post winter:2 weeks of winter	1.317	0.8471	0.4698	0.5996	6
173	4 weeks post winter:4 weeks of winter vs. SPC -4 weeks post winter:4 weeks of winter	1.317	1.157	0.16	0.5996	6
174	4 weeks post winter:4 weeks of winter vs. SPC -4 weeks post winter:8 weeks of winter	1.317	1.258	0.05934	0.5996	6
175	4 weeks post winter:4 weeks of winter vs. SPC -8 weeks post winter:2 weeks of winter	1.317	1.097	0.2194	0.5996	6
176	4 weeks post winter:4 weeks of winter vs. SPC -8 weeks post winter:4 weeks of winter	1.317	1.258	0.05934	0.5996	6
177	4 weeks post winter:4 weeks of winter vs. SPC -8 weeks post winter:8 weeks of winter	1.317	1.545	-0.2277	0.5996	6
178	4 weeks post winter:8 weeks of winter vs. 8 weeks post winter:2 weeks of winter	1.286	1.045	0.2403	0.5996	6
179	4 weeks post winter:8 weeks of winter vs. 8 weeks post winter:4 weeks of winter	1.286	1.637	-0.3516	0.5996	6
180	4 weeks post winter:8 weeks of winter vs. 8 weeks post winter:8 weeks of winter	1.286	6.115	-4.83	0.5996	6

2way ANOVA<br>Multiple comparisons						
181	4 weeks post winter:8 weeks of winter vs. SPC -4 weeks post winter:2 weeks of winter	1.286	0.8471	0.4384	0.5996	6
182	4 weeks post winter:8 weeks of winter vs. SPC -4 weeks post winter:4 weeks of winter	1.286	1.157	0.1286	0.5996	6
183	4 weeks post winter:8 weeks of winter vs. SPC -4 weeks post winter:8 weeks of winter	1.286	1.258	0.02799	0.5996	6
184	4 weeks post winter:8 weeks of winter vs. SPC -8 weeks post winter:2 weeks of winter	1.286	1.097	0.188	0.5996	6
185	4 weeks post winter:8 weeks of winter vs. SPC -8 weeks post winter:4 weeks of winter	1.286	1.258	0.02799	0.5996	6
186	4 weeks post winter:8 weeks of winter vs. SPC -8 weeks post winter:8 weeks of winter	1.286	1.545	-0.2591	0.5996	6
187	8 weeks post winter:2 weeks of winter vs. 8 weeks post winter:4 weeks of winter	1.045	1.637	-0.5919	0.5996	6
188	8 weeks post winter:2 weeks of winter vs. 8 weeks post winter:8 weeks of winter	1.045	6.115	-5.07	0.5996	6
189	8 weeks post winter:2 weeks of winter vs. SPC -4 weeks post winter:2 weeks of winter	1.045	0.8471	0.1982	0.5996	6
190	8 weeks post winter:2 weeks of winter vs. SPC -4 weeks post winter:4 weeks of winter	1.045	1.157	-0.1117	0.5996	6
191	8 weeks post winter:2 weeks of winter vs. SPC -4 weeks post winter:8 weeks of winter	1.045	1.258	-0.2123	0.5996	6
192	8 weeks post winter:2 weeks of winter vs. SPC -8 weeks post winter:2 weeks of winter	1.045	1.097	-0.05225	0.5996	6
193	8 weeks post winter:2 weeks of winter vs. SPC -8 weeks post winter:4 weeks of winter	1.045	1.258	-0.2123	0.5996	6
194	8 weeks post winter:2 weeks of winter vs. SPC -8 weeks post winter:8 weeks of winter	1.045	1.545	-0.4993	0.5996	6
195	8 weeks post winter:4 weeks of winter vs. 8 weeks post winter:8 weeks of winter	1.637	6.115	-4.478	0.5996	6
196	8 weeks post winter:4 weeks of winter vs. SPC -4 weeks post winter:2 weeks of winter	1.637	0.8471	0.79	0.5996	6
197	8 weeks post winter:4 weeks of winter vs. SPC -4 weeks post winter:4 weeks of winter	1.637	1.157	0.4802	0.5996	6
198	8 weeks post winter:4 weeks of winter vs. SPC -4 weeks post winter:8 weeks of winter	1.637	1.258	0.3796	0.5996	6
199	8 weeks post winter:4 weeks of winter vs. SPC -8 weeks post winter:2 weeks of winter	1.637	1.097	0.5396	0.5996	6
200	8 weeks post winter:4 weeks of winter vs. SPC -8 weeks post winter:4 weeks of winter	1.637	1.258	0.3796	0.5996	6
201	8 weeks post winter:4 weeks of winter vs. SPC -8 weeks post winter:8 weeks of winter	1.637	1.545	0.09251	0.5996	6
202	8 weeks post winter:8 weeks of winter vs. SPC -4 weeks post winter:2 weeks of winter	6.115	0.8471	5.268	0.5996	6
203	8 weeks post winter:8 weeks of winter vs. SPC -4 weeks post winter:4 weeks of winter	6.115	1.157	4.958	0.5996	6
204	8 weeks post winter:8 weeks of winter vs. SPC -4 weeks post winter:8 weeks of winter	6.115	1.258	4.858	0.5996	6
205	8 weeks post winter:8 weeks of winter vs. SPC -8 weeks post winter:2 weeks of winter	6.115	1.097	5.018	0.5996	6
206	8 weeks post winter:8 weeks of winter vs. SPC -8 weeks post winter:4 weeks of winter	6.115	1.258	4.858	0.5996	6
207	8 weeks post winter:8 weeks of winter vs. SPC -8 weeks post winter:8 weeks of winter	6.115	1.545	4.571	0.5996	6
208	SPC -4 weeks post winter:2 weeks of winter vs. SPC -4 weeks post winter:4 weeks of winter	0.8471	1.157	-0.3098	0.5996	6
209	SPC -4 weeks post winter:2 weeks of winter vs. SPC -4 weeks post winter:8 weeks of winter	0.8471	1.258	-0.4105	0.5996	6
210	SPC -4 weeks post winter:2 weeks of winter vs. SPC -8 weeks post winter:2 weeks of winter	0.8471	1.097	-0.2504	0.5996	6

2way ANOVA
Multiple comparisons

211	SPC -4 weeks post winter:2 weeks of winter vs. SPC -8 weeks post winter:4 weeks of winter	0.8471	1.258	-0.4105	0.5996	6
212	SPC -4 weeks post winter:2 weeks of winter vs. SPC -8 weeks post winter:8 weeks of winter	0.8471	1.545	-0.6975	0.5996	6
213	SPC -4 weeks post winter:4 weeks of winter vs. SPC -4 weeks post winter:8 weeks of winter	1.157	1.258	-0.1006	0.5996	6
214	SPC -4 weeks post winter:4 weeks of winter vs. SPC -8 weeks post winter:2 weeks of winter	1.157	1.097	0.05941	0.5996	6
215	SPC -4 weeks post winter:4 weeks of winter vs. SPC -8 weeks post winter:4 weeks of winter	1.157	1.258	-0.1006	0.5996	6
216	SPC -4 weeks post winter:4 weeks of winter vs. SPC -8 weeks post winter:8 weeks of winter	1.157	1.545	-0.3877	0.5996	6
217	SPC -4 weeks post winter:8 weeks of winter vs. SPC -8 weeks post winter:2 weeks of winter	1.258	1.097	0.16	0.5996	6
218	SPC -4 weeks post winter:8 weeks of winter vs. SPC -8 weeks post winter:4 weeks of winter	1.258	1.258	0	0.5996	6
219	SPC -4 weeks post winter:8 weeks of winter vs. SPC -8 weeks post winter:8 weeks of winter	1.258	1.545	-0.287	0.5996	6
220	SPC -8 weeks post winter:2 weeks of winter vs. SPC -8 weeks post winter:4 weeks of winter	1.097	1.258	-0.16	0.5996	6
221	SPC -8 weeks post winter:2 weeks of winter vs. SPC -8 weeks post winter:8 weeks of winter	1.097	1.545	-0.4471	0.5996	6
222	SPC -8 weeks post winter:4 weeks of winter vs. SPC -8 weeks post winter:8 weeks of winter	1.258	1.545	-0.287	0.5996	6

1			
2			
3			
4			
5			
6			
7			
8			
9			
10			
11			
12			
13			
14			
15			
16			
17			
18			
19			
20			
21			
22			
23			
24			
25			
26			
27			
28			
29			
30			

31			
32			
33			
34			
35			
36			
37			
38			
39			
40			
41			
42			
43			
44			
45			
46			
47			
48			
49			
50			
51			
52			
53			
54			
55			
56			
57			
58			
59			
60			

**61**			
**62**			
**63**			
**64**			
**65**			
**66**			
**67**			
**68**			
**69**			
**70**			
**71**			
**72**			
**73**			
**74**			
**75**			
**76**			
**77**			
**78**			
**79**			
**80**			
**81**			
**82**			
**83**			
**84**			
**85**			
**86**			
**87**			
**88**			
**89**			
**90**			

91			
92			
93			
94			
95			
96			
97			
98			
99			
100			
101			
102			
103			
104			
105			
106			
107			
108			
109			
110			
111			
112			
113			
114			
115			
116	N2	q	DF
117			
118	6	0.1562	75
119	6	0.8641	75
120	6	1.475	75

**121**	6	1.241	75
**122**	6	1.168	75
**123**	6	0.6008	75
**124**	6	1.997	75
**125**	6	12.56	75
**126**	6	0.1334	75
**127**	6	0.8641	75
**128**	6	1.101	75
**129**	6	0.724	75
**130**	6	1.101	75
**131**	6	1.779	75
**132**	6	0.708	75
**133**	6	1.319	75
**134**	6	1.085	75
**135**	6	1.011	75
**136**	6	0.4446	75
**137**	6	1.841	75
**138**	6	12.4	75
**139**	6	0.02276	75
**140**	6	0.708	75
**141**	6	0.9453	75
**142**	6	0.5679	75
**143**	6	0.9453	75
**144**	6	1.622	75
**145**	6	0.6107	75
**146**	6	0.3773	75
**147**	6	0.3034	75
**148**	6	0.2634	75
**149**	6	1.133	75
**150**	6	11.69	75

151	6	0.7307	75
152	6	0	75
153	6	0.2373	75
154	6	0.1401	75
155	6	0.2373	75
156	6	0.9144	75
157	6	0.2334	75
158	6	0.3074	75
159	6	0.8741	75
160	6	0.5218	75
161	6	11.08	75
162	6	1.341	75
163	6	0.6107	75
164	6	0.3734	75
165	6	0.7509	75
166	6	0.3734	75
167	6	0.3036	75
168	6	0.07394	75
169	6	0.6407	75
170	6	0.7553	75
171	6	11.32	75
172	6	1.108	75
173	6	0.3773	75
174	6	0.14	75
175	6	0.5174	75
176	6	0.14	75
177	6	0.5371	75
178	6	0.5667	75
179	6	0.8292	75
180	6	11.39	75

**181**	6	1.034	75
**182**	6	0.3034	75
**183**	6	0.06602	75
**184**	6	0.4435	75
**185**	6	0.06602	75
**186**	6	0.611	75
**187**	6	1.396	75
**188**	6	11.96	75
**189**	6	0.4674	75
**190**	6	0.2634	75
**191**	6	0.5007	75
**192**	6	0.1232	75
**193**	6	0.5007	75
**194**	6	1.178	75
**195**	6	10.56	75
**196**	6	1.863	75
**197**	6	1.133	75
**198**	6	0.8952	75
**199**	6	1.273	75
**200**	6	0.8952	75
**201**	6	0.2182	75
**202**	6	12.43	75
**203**	6	11.69	75
**204**	6	11.46	75
**205**	6	11.83	75
**206**	6	11.46	75
**207**	6	10.78	75
**208**	6	0.7307	75
**209**	6	0.9681	75
**210**	6	0.5906	75

211	6	0.9681	75
212	6	1.645	75
213	6	0.2373	75
214	6	0.1401	75
215	6	0.2373	75
216	6	0.9144	75
217	6	0.3775	75
218	6	0	75
219	6	0.677	75
220	6	0.3775	75
221	6	1.055	75
222	6	0.677	75

2way ANOVA
Tabular results

1	Table Analyzed	CAPN2				
2						
3	Two-way ANOVA	Ordinary				
4	Alpha	0.05				
5						
6	Source of Variation	% of total variation	P value	P value summary	Significant?	
7	Interaction	31.95	<0.0001	****	Yes	
8	Time post-winter	33.47	<0.0001	****	Yes	
9	Treatment	14.52	<0.0001	****	Yes	
10						
11	ANOVA table	SS	DF	MS	F (DFn, DFd)	P value
12	Interaction	16000	8	2000	F (8, 75) = 14.94	P<0.0001
13	Time post-winter	16760	4	4190	F (4, 75) = 31.3	P<0.0001
14	Treatment	7272	2	3636	F (2, 75) = 27.16	P<0.0001
15	Residual	10040	75	133.9		
16						
17	Number of missing values	0				

2way ANOVA
Multiple comparisons

1	Compare cell means regardless of rows and columns					
2						
3	Number of families	1				
4	Number of comparisons per family	105				
5	Alpha	0.05				
6						
7	Tukey's multiple comparisons test	Mean Diff.	95.00% CI of diff.	Significant?	Summary	Adjusted P Value
8						
9	End of winter:2 weeks of winter vs. End of winter:4 weeks of winter	0.1047	-23.32 to 23.53	No	ns	>0.9999
10	End of winter:2 weeks of winter vs. End of winter:8 weeks of winter	0.1421	-23.28 to 23.57	No	ns	>0.9999
11	End of winter:2 weeks of winter vs. 4 weeks post winter:2 weeks of winter	-0.7254	-24.15 to 22.7	No	ns	>0.9999
12	End of winter:2 weeks of winter vs. 4 weeks post winter:4 weeks of winter	-2.913	-26.34 to 20.51	No	ns	>0.9999
13	End of winter:2 weeks of winter vs. 4 weeks post winter:8 weeks of winter	-23.76	-47.19 to -0.3322	Yes	*	0.0435
14	End of winter:2 weeks of winter vs. 8 weeks post winter:2 weeks of winter	-2.268	-25.69 to 21.16	No	ns	>0.9999
15	End of winter:2 weeks of winter vs. 8 weeks post winter:4 weeks of winter	-20.7	-44.13 to 2.727	No	ns	0.1422
16	End of winter:2 weeks of winter vs. 8 weeks post winter:8 weeks of winter	-82.74	-106.2 to -59.32	Yes	****	<0.0001
17	End of winter:2 weeks of winter vs. SPC -4 weeks post winter:2 weeks of winter	0.1328	-23.29 to 23.56	No	ns	>0.9999
18	End of winter:2 weeks of winter vs. SPC -4 weeks post winter:4 weeks of winter	0.1421	-23.28 to 23.57	No	ns	>0.9999
19	End of winter:2 weeks of winter vs. SPC -4 weeks post winter:8 weeks of winter	-0.007331	-23.43 to 23.42	No	ns	>0.9999
20	End of winter:2 weeks of winter vs. SPC -8 weeks post winter:2 weeks of winter	-0.07921	-23.51 to 23.35	No	ns	>0.9999
21	End of winter:2 weeks of winter vs. SPC -8 weeks post winter:4 weeks of winter	-0.007331	-23.43 to 23.42	No	ns	>0.9999
22	End of winter:2 weeks of winter vs. SPC -8 weeks post winter:8 weeks of winter	-0.4728	-23.9 to 22.95	No	ns	>0.9999
23	End of winter:4 weeks of winter vs. End of winter:8 weeks of winter	0.0374	-23.39 to 23.46	No	ns	>0.9999
24	End of winter:4 weeks of winter vs. 4 weeks post winter:2 weeks of winter	-0.8301	-24.26 to 22.6	No	ns	>0.9999
25	End of winter:4 weeks of winter vs. 4 weeks post winter:4 weeks of winter	-3.018	-26.44 to 20.41	No	ns	>0.9999
26	End of winter:4 weeks of winter vs. 4 weeks post winter:8 weeks of winter	-23.86	-47.29 to -0.4369	Yes	*	0.0416
27	End of winter:4 weeks of winter vs. 8 weeks post winter:2 weeks of winter	-2.372	-25.8 to 21.05	No	ns	>0.9999
28	End of winter:4 weeks of winter vs. 8 weeks post winter:4 weeks of winter	-20.8	-44.23 to 2.623	No	ns	0.1371
29	End of winter:4 weeks of winter vs. 8 weeks post winter:8 weeks of winter	-82.85	-106.3 to -59.42	Yes	****	<0.0001
30	End of winter:4 weeks of winter vs. SPC -4 weeks post winter:2 weeks of winter	0.02807	-23.4 to 23.45	No	ns	>0.9999

**2way ANOVA**<br>Multiple comparisons						
31	End of winter:4 weeks of winter vs. SPC -4 weeks post winter:4 weeks of winter	0.0374	-23.39 to 23.46	No	ns	>0.9999
32	End of winter:4 weeks of winter vs. SPC -4 weeks post winter:8 weeks of winter	-0.112	-23.54 to 23.31	No	ns	>0.9999
33	End of winter:4 weeks of winter vs. SPC -8 weeks post winter:2 weeks of winter	-0.1839	-23.61 to 23.24	No	ns	>0.9999
34	End of winter:4 weeks of winter vs. SPC -8 weeks post winter:4 weeks of winter	-0.112	-23.54 to 23.31	No	ns	>0.9999
35	End of winter:4 weeks of winter vs. SPC -8 weeks post winter:8 weeks of winter	-0.5775	-24 to 22.85	No	ns	>0.9999
36	End of winter:8 weeks of winter vs. 4 weeks post winter:2 weeks of winter	-0.8675	-24.29 to 22.56	No	ns	>0.9999
37	End of winter:8 weeks of winter vs. 4 weeks post winter:4 weeks of winter	-3.055	-26.48 to 20.37	No	ns	>0.9999
38	End of winter:8 weeks of winter vs. 4 weeks post winter:8 weeks of winter	-23.9	-47.33 to -0.4743	Yes	*	0.0410
39	End of winter:8 weeks of winter vs. 8 weeks post winter:2 weeks of winter	-2.41	-25.84 to 21.02	No	ns	>0.9999
40	End of winter:8 weeks of winter vs. 8 weeks post winter:4 weeks of winter	-20.84	-44.27 to 2.585	No	ns	0.1353
41	End of winter:8 weeks of winter vs. 8 weeks post winter:8 weeks of winter	-82.89	-106.3 to -59.46	Yes	****	<0.0001
42	End of winter:8 weeks of winter vs. SPC -4 weeks post winter:2 weeks of winter	-0.009339	-23.44 to 23.42	No	ns	>0.9999
43	End of winter:8 weeks of winter vs. SPC -4 weeks post winter:4 weeks of winter	0	-23.43 to 23.43	No	ns	>0.9999
44	End of winter:8 weeks of winter vs. SPC -4 weeks post winter:8 weeks of winter	-0.1494	-23.58 to 23.28	No	ns	>0.9999
45	End of winter:8 weeks of winter vs. SPC -8 weeks post winter:2 weeks of winter	-0.2213	-23.65 to 23.21	No	ns	>0.9999
46	End of winter:8 weeks of winter vs. SPC -8 weeks post winter:4 weeks of winter	-0.1494	-23.58 to 23.28	No	ns	>0.9999
47	End of winter:8 weeks of winter vs. SPC -8 weeks post winter:8 weeks of winter	-0.6149	-24.04 to 22.81	No	ns	>0.9999
48	4 weeks post winter:2 weeks of winter vs. 4 weeks post winter:4 weeks of winter	-2.187	-25.61 to 21.24	No	ns	>0.9999
49	4 weeks post winter:2 weeks of winter vs. 4 weeks post winter:8 weeks of winter	-23.03	-46.46 to 0.3932	No	ns	0.0588
50	4 weeks post winter:2 weeks of winter vs. 8 weeks post winter:2 weeks of winter	-1.542	-24.97 to 21.88	No	ns	>0.9999
51	4 weeks post winter:2 weeks of winter vs. 8 weeks post winter:4 weeks of winter	-19.97	-43.4 to 3.453	No	ns	0.1819
52	4 weeks post winter:2 weeks of winter vs. 8 weeks post winter:8 weeks of winter	-82.02	-105.4 to -58.59	Yes	****	<0.0001
53	4 weeks post winter:2 weeks of winter vs. SPC -4 weeks post winter:2 weeks of winter	0.8582	-22.57 to 24.28	No	ns	>0.9999
54	4 weeks post winter:2 weeks of winter vs. SPC -4 weeks post winter:4 weeks of winter	0.8675	-22.56 to 24.29	No	ns	>0.9999
55	4 weeks post winter:2 weeks of winter vs. SPC -4 weeks post winter:8 weeks of winter	0.7181	-22.71 to 24.14	No	ns	>0.9999
56	4 weeks post winter:2 weeks of winter vs. SPC -8 weeks post winter:2 weeks of winter	0.6462	-22.78 to 24.07	No	ns	>0.9999
57	4 weeks post winter:2 weeks of winter vs. SPC -8 weeks post winter:4 weeks of winter	0.7181	-22.71 to 24.14	No	ns	>0.9999
58	4 weeks post winter:2 weeks of winter vs. SPC -8 weeks post winter:8 weeks of winter	0.2527	-23.17 to 23.68	No	ns	>0.9999
59	4 weeks post winter:4 weeks of winter vs. 4 weeks post winter:8 weeks of winter	-20.85	-44.27 to 2.581	No	ns	0.1351
60	4 weeks post winter:4 weeks of winter vs. 8 weeks post winter:2 weeks of winter	0.6452	-22.78 to 24.07	No	ns	>0.9999

2way ANOVA<br>Multiple comparisons						
61	4 weeks post winter:4 weeks of winter vs. 8 weeks post winter:4 weeks of winter	-17.79	-41.21 to 5.64	No	ns	0.3476
62	4 weeks post winter:4 weeks of winter vs. 8 weeks post winter:8 weeks of winter	-79.83	-103.3 to -56.4	Yes	****	<0.0001
63	4 weeks post winter:4 weeks of winter vs. SPC -4 weeks post winter:2 weeks of winter	3.046	-20.38 to 26.47	No	ns	>0.9999
64	4 weeks post winter:4 weeks of winter vs. SPC -4 weeks post winter:4 weeks of winter	3.055	-20.37 to 26.48	No	ns	>0.9999
65	4 weeks post winter:4 weeks of winter vs. SPC -4 weeks post winter:8 weeks of winter	2.906	-20.52 to 26.33	No	ns	>0.9999
66	4 weeks post winter:4 weeks of winter vs. SPC -8 weeks post winter:2 weeks of winter	2.834	-20.59 to 26.26	No	ns	>0.9999
67	4 weeks post winter:4 weeks of winter vs. SPC -8 weeks post winter:4 weeks of winter	2.906	-20.52 to 26.33	No	ns	>0.9999
68	4 weeks post winter:4 weeks of winter vs. SPC -8 weeks post winter:8 weeks of winter	2.44	-20.99 to 25.87	No	ns	>0.9999
69	4 weeks post winter:8 weeks of winter vs. 8 weeks post winter:2 weeks of winter	21.49	-1.935 to 44.92	No	ns	0.1070
70	4 weeks post winter:8 weeks of winter vs. 8 weeks post winter:4 weeks of winter	3.06	-20.37 to 26.49	No	ns	>0.9999
71	4 weeks post winter:8 weeks of winter vs. 8 weeks post winter:8 weeks of winter	-58.98	-82.41 to -35.56	Yes	****	<0.0001
72	4 weeks post winter:8 weeks of winter vs. SPC -4 weeks post winter:2 weeks of winter	23.89	0.465 to 47.32	Yes	*	0.0411
73	4 weeks post winter:8 weeks of winter vs. SPC -4 weeks post winter:4 weeks of winter	23.9	0.4743 to 47.33	Yes	*	0.0410
74	4 weeks post winter:8 weeks of winter vs. SPC -4 weeks post winter:8 weeks of winter	23.75	0.3249 to 47.18	Yes	*	0.0436
75	4 weeks post winter:8 weeks of winter vs. SPC -8 weeks post winter:2 weeks of winter	23.68	0.253 to 47.11	Yes	*	0.0450
76	4 weeks post winter:8 weeks of winter vs. SPC -8 weeks post winter:4 weeks of winter	23.75	0.3249 to 47.18	Yes	*	0.0436
77	4 weeks post winter:8 weeks of winter vs. SPC -8 weeks post winter:8 weeks of winter	23.29	-0.1405 to 46.71	No	ns	0.0530
78	8 weeks post winter:2 weeks of winter vs. 8 weeks post winter:4 weeks of winter	-18.43	-41.86 to 4.995	No	ns	0.2916
79	8 weeks post winter:2 weeks of winter vs. 8 weeks post winter:8 weeks of winter	-80.48	-103.9 to -57.05	Yes	****	<0.0001
80	8 weeks post winter:2 weeks of winter vs. SPC -4 weeks post winter:2 weeks of winter	2.4	-21.03 to 25.83	No	ns	>0.9999
81	8 weeks post winter:2 weeks of winter vs. SPC -4 weeks post winter:4 weeks of winter	2.41	-21.02 to 25.84	No	ns	>0.9999
82	8 weeks post winter:2 weeks of winter vs. SPC -4 weeks post winter:8 weeks of winter	2.26	-21.17 to 25.69	No	ns	>0.9999
83	8 weeks post winter:2 weeks of winter vs. SPC -8 weeks post winter:2 weeks of winter	2.189	-21.24 to 25.62	No	ns	>0.9999
84	8 weeks post winter:2 weeks of winter vs. SPC -8 weeks post winter:4 weeks of winter	2.26	-21.17 to 25.69	No	ns	>0.9999
85	8 weeks post winter:2 weeks of winter vs. SPC -8 weeks post winter:8 weeks of winter	1.795	-21.63 to 25.22	No	ns	>0.9999
86	8 weeks post winter:4 weeks of winter vs. 8 weeks post winter:8 weeks of winter	-62.04	-85.47 to -38.62	Yes	****	<0.0001
87	8 weeks post winter:4 weeks of winter vs. SPC -4 weeks post winter:2 weeks of winter	20.83	-2.595 to 44.26	No	ns	0.1357
88	8 weeks post winter:4 weeks of winter vs. SPC -4 weeks post winter:4 weeks of winter	20.84	-2.585 to 44.27	No	ns	0.1353
89	8 weeks post winter:4 weeks of winter vs. SPC -4 weeks post winter:8 weeks of winter	20.69	-2.735 to 44.12	No	ns	0.1426
90	8 weeks post winter:4 weeks of winter vs. SPC -8 weeks post winter:2 weeks of winter	20.62	-2.807 to 44.05	No	ns	0.1462

2way ANOVA<br>Multiple comparisons						
91	8 weeks post winter:4 weeks of winter vs. SPC -8 weeks post winter:4 weeks of winter	20.69	-2.735 to 44.12	No	ns	0.1426
92	8 weeks post winter:4 weeks of winter vs. SPC -8 weeks post winter:8 weeks of winter	20.23	-3.2 to 43.65	No	ns	0.1672
93	8 weeks post winter:8 weeks of winter vs. SPC -4 weeks post winter:2 weeks of winter	82.88	59.45 to 106.3	Yes	****	<0.0001
94	8 weeks post winter:8 weeks of winter vs. SPC -4 weeks post winter:4 weeks of winter	82.89	59.46 to 106.3	Yes	****	<0.0001
95	8 weeks post winter:8 weeks of winter vs. SPC -4 weeks post winter:8 weeks of winter	82.74	59.31 to 106.2	Yes	****	<0.0001
96	8 weeks post winter:8 weeks of winter vs. SPC -8 weeks post winter:2 weeks of winter	82.66	59.24 to 106.1	Yes	****	<0.0001
97	8 weeks post winter:8 weeks of winter vs. SPC -8 weeks post winter:4 weeks of winter	82.74	59.31 to 106.2	Yes	****	<0.0001
98	8 weeks post winter:8 weeks of winter vs. SPC -8 weeks post winter:8 weeks of winter	82.27	58.84 to 105.7	Yes	****	<0.0001
99	SPC -4 weeks post winter:2 weeks of winter vs. SPC -4 weeks post winter:4 weeks of winter	0.009339	-23.42 to 23.44	No	ns	>0.9999
100	SPC -4 weeks post winter:2 weeks of winter vs. SPC -4 weeks post winter:8 weeks of winter	-0.1401	-23.57 to 23.29	No	ns	>0.9999
101	SPC -4 weeks post winter:2 weeks of winter vs. SPC -8 weeks post winter:2 weeks of winter	-0.212	-23.64 to 23.21	No	ns	>0.9999
102	SPC -4 weeks post winter:2 weeks of winter vs. SPC -8 weeks post winter:4 weeks of winter	-0.1401	-23.57 to 23.29	No	ns	>0.9999
103	SPC -4 weeks post winter:2 weeks of winter vs. SPC -8 weeks post winter:8 weeks of winter	-0.6055	-24.03 to 22.82	No	ns	>0.9999
104	SPC -4 weeks post winter:4 weeks of winter vs. SPC -4 weeks post winter:8 weeks of winter	-0.1494	-23.58 to 23.28	No	ns	>0.9999
105	SPC -4 weeks post winter:4 weeks of winter vs. SPC -8 weeks post winter:2 weeks of winter	-0.2213	-23.65 to 23.21	No	ns	>0.9999
106	SPC -4 weeks post winter:4 weeks of winter vs. SPC -8 weeks post winter:4 weeks of winter	-0.1494	-23.58 to 23.28	No	ns	>0.9999
107	SPC -4 weeks post winter:4 weeks of winter vs. SPC -8 weeks post winter:8 weeks of winter	-0.6149	-24.04 to 22.81	No	ns	>0.9999
108	SPC -4 weeks post winter:8 weeks of winter vs. SPC -8 weeks post winter:2 weeks of winter	-0.07187	-23.5 to 23.35	No	ns	>0.9999
109	SPC -4 weeks post winter:8 weeks of winter vs. SPC -8 weeks post winter:4 weeks of winter	0	-23.43 to 23.43	No	ns	>0.9999
110	SPC -4 weeks post winter:8 weeks of winter vs. SPC -8 weeks post winter:8 weeks of winter	-0.4654	-23.89 to 22.96	No	ns	>0.9999
111	SPC -8 weeks post winter:2 weeks of winter vs. SPC -8 weeks post winter:4 weeks of winter	0.07187	-23.35 to 23.5	No	ns	>0.9999
112	SPC -8 weeks post winter:2 weeks of winter vs. SPC -8 weeks post winter:8 weeks of winter	-0.3936	-23.82 to 23.03	No	ns	>0.9999
113	SPC -8 weeks post winter:4 weeks of winter vs. SPC -8 weeks post winter:8 weeks of winter	-0.4654	-23.89 to 22.96	No	ns	>0.9999
114						
115						
116	Test details	Mean 1	Mean 2	Mean Diff.	SE of diff.	N1
117						
118	End of winter:2 weeks of winter vs. End of winter:4 weeks of winter	0.5799	0.4752	0.1047	6.68	6
119	End of winter:2 weeks of winter vs. End of winter:8 weeks of winter	0.5799	0.4378	0.1421	6.68	6
120	End of winter:2 weeks of winter vs. 4 weeks post winter:2 weeks of winter	0.5799	1.305	-0.7254	6.68	6

2way ANOVA
Multiple comparisons

121	End of winter:2 weeks of winter vs. 4 weeks post winter:4 weeks of winter	0.5799	3.493	-2.913	6.68	6
122	End of winter:2 weeks of winter vs. 4 weeks post winter:8 weeks of winter	0.5799	24.34	-23.76	6.68	6
123	End of winter:2 weeks of winter vs. 8 weeks post winter:2 weeks of winter	0.5799	2.848	-2.268	6.68	6
124	End of winter:2 weeks of winter vs. 8 weeks post winter:4 weeks of winter	0.5799	21.28	-20.7	6.68	6
125	End of winter:2 weeks of winter vs. 8 weeks post winter:8 weeks of winter	0.5799	83.32	-82.74	6.68	6
126	End of winter:2 weeks of winter vs. SPC -4 weeks post winter:2 weeks of winter	0.5799	0.4471	0.1328	6.68	6
127	End of winter:2 weeks of winter vs. SPC -4 weeks post winter:4 weeks of winter	0.5799	0.4378	0.1421	6.68	6
128	End of winter:2 weeks of winter vs. SPC -4 weeks post winter:8 weeks of winter	0.5799	0.5872	-0.007331	6.68	6
129	End of winter:2 weeks of winter vs. SPC -8 weeks post winter:2 weeks of winter	0.5799	0.6591	-0.07921	6.68	6
130	End of winter:2 weeks of winter vs. SPC -8 weeks post winter:4 weeks of winter	0.5799	0.5872	-0.007331	6.68	6
131	End of winter:2 weeks of winter vs. SPC -8 weeks post winter:8 weeks of winter	0.5799	1.053	-0.4728	6.68	6
132	End of winter:4 weeks of winter vs. End of winter:8 weeks of winter	0.4752	0.4378	0.0374	6.68	6
133	End of winter:4 weeks of winter vs. 4 weeks post winter:2 weeks of winter	0.4752	1.305	-0.8301	6.68	6
134	End of winter:4 weeks of winter vs. 4 weeks post winter:4 weeks of winter	0.4752	3.493	-3.018	6.68	6
135	End of winter:4 weeks of winter vs. 4 weeks post winter:8 weeks of winter	0.4752	24.34	-23.86	6.68	6
136	End of winter:4 weeks of winter vs. 8 weeks post winter:2 weeks of winter	0.4752	2.848	-2.372	6.68	6
137	End of winter:4 weeks of winter vs. 8 weeks post winter:4 weeks of winter	0.4752	21.28	-20.8	6.68	6
138	End of winter:4 weeks of winter vs. 8 weeks post winter:8 weeks of winter	0.4752	83.32	-82.85	6.68	6
139	End of winter:4 weeks of winter vs. SPC -4 weeks post winter:2 weeks of winter	0.4752	0.4471	0.02807	6.68	6
140	End of winter:4 weeks of winter vs. SPC -4 weeks post winter:4 weeks of winter	0.4752	0.4378	0.0374	6.68	6
141	End of winter:4 weeks of winter vs. SPC -4 weeks post winter:8 weeks of winter	0.4752	0.5872	-0.112	6.68	6
142	End of winter:4 weeks of winter vs. SPC -8 weeks post winter:2 weeks of winter	0.4752	0.6591	-0.1839	6.68	6
143	End of winter:4 weeks of winter vs. SPC -8 weeks post winter:4 weeks of winter	0.4752	0.5872	-0.112	6.68	6
144	End of winter:4 weeks of winter vs. SPC -8 weeks post winter:8 weeks of winter	0.4752	1.053	-0.5775	6.68	6
145	End of winter:8 weeks of winter vs. 4 weeks post winter:2 weeks of winter	0.4378	1.305	-0.8675	6.68	6
146	End of winter:8 weeks of winter vs. 4 weeks post winter:4 weeks of winter	0.4378	3.493	-3.055	6.68	6
147	End of winter:8 weeks of winter vs. 4 weeks post winter:8 weeks of winter	0.4378	24.34	-23.9	6.68	6
148	End of winter:8 weeks of winter vs. 8 weeks post winter:2 weeks of winter	0.4378	2.848	-2.41	6.68	6
149	End of winter:8 weeks of winter vs. 8 weeks post winter:4 weeks of winter	0.4378	21.28	-20.84	6.68	6
150	End of winter:8 weeks of winter vs. 8 weeks post winter:8 weeks of winter	0.4378	83.32	-82.89	6.68	6

2way ANOVA<br>Multiple comparisons						
151	End of winter:8 weeks of winter vs. SPC -4 weeks post winter:2 weeks of winter	0.4378	0.4471	-0.009339	6.68	6
152	End of winter:8 weeks of winter vs. SPC -4 weeks post winter:4 weeks of winter	0.4378	0.4378	0	6.68	6
153	End of winter:8 weeks of winter vs. SPC -4 weeks post winter:8 weeks of winter	0.4378	0.5872	-0.1494	6.68	6
154	End of winter:8 weeks of winter vs. SPC -8 weeks post winter:2 weeks of winter	0.4378	0.6591	-0.2213	6.68	6
155	End of winter:8 weeks of winter vs. SPC -8 weeks post winter:4 weeks of winter	0.4378	0.5872	-0.1494	6.68	6
156	End of winter:8 weeks of winter vs. SPC -8 weeks post winter:8 weeks of winter	0.4378	1.053	-0.6149	6.68	6
157	4 weeks post winter:2 weeks of winter vs. 4 weeks post winter:4 weeks of winter	1.305	3.493	-2.187	6.68	6
158	4 weeks post winter:2 weeks of winter vs. 4 weeks post winter:8 weeks of winter	1.305	24.34	-23.03	6.68	6
159	4 weeks post winter:2 weeks of winter vs. 8 weeks post winter:2 weeks of winter	1.305	2.848	-1.542	6.68	6
160	4 weeks post winter:2 weeks of winter vs. 8 weeks post winter:4 weeks of winter	1.305	21.28	-19.97	6.68	6
161	4 weeks post winter:2 weeks of winter vs. 8 weeks post winter:8 weeks of winter	1.305	83.32	-82.02	6.68	6
162	4 weeks post winter:2 weeks of winter vs. SPC -4 weeks post winter:2 weeks of winter	1.305	0.4471	0.8582	6.68	6
163	4 weeks post winter:2 weeks of winter vs. SPC -4 weeks post winter:4 weeks of winter	1.305	0.4378	0.8675	6.68	6
164	4 weeks post winter:2 weeks of winter vs. SPC -4 weeks post winter:8 weeks of winter	1.305	0.5872	0.7181	6.68	6
165	4 weeks post winter:2 weeks of winter vs. SPC -8 weeks post winter:2 weeks of winter	1.305	0.6591	0.6462	6.68	6
166	4 weeks post winter:2 weeks of winter vs. SPC -8 weeks post winter:4 weeks of winter	1.305	0.5872	0.7181	6.68	6
167	4 weeks post winter:2 weeks of winter vs. SPC -8 weeks post winter:8 weeks of winter	1.305	1.053	0.2527	6.68	6
168	4 weeks post winter:4 weeks of winter vs. 4 weeks post winter:8 weeks of winter	3.493	24.34	-20.85	6.68	6
169	4 weeks post winter:4 weeks of winter vs. 8 weeks post winter:2 weeks of winter	3.493	2.848	0.6452	6.68	6
170	4 weeks post winter:4 weeks of winter vs. 8 weeks post winter:4 weeks of winter	3.493	21.28	-17.79	6.68	6
171	4 weeks post winter:4 weeks of winter vs. 8 weeks post winter:8 weeks of winter	3.493	83.32	-79.83	6.68	6
172	4 weeks post winter:4 weeks of winter vs. SPC -4 weeks post winter:2 weeks of winter	3.493	0.4471	3.046	6.68	6
173	4 weeks post winter:4 weeks of winter vs. SPC -4 weeks post winter:4 weeks of winter	3.493	0.4378	3.055	6.68	6
174	4 weeks post winter:4 weeks of winter vs. SPC -4 weeks post winter:8 weeks of winter	3.493	0.5872	2.906	6.68	6
175	4 weeks post winter:4 weeks of winter vs. SPC -8 weeks post winter:2 weeks of winter	3.493	0.6591	2.834	6.68	6
176	4 weeks post winter:4 weeks of winter vs. SPC -8 weeks post winter:4 weeks of winter	3.493	0.5872	2.906	6.68	6
177	4 weeks post winter:4 weeks of winter vs. SPC -8 weeks post winter:8 weeks of winter	3.493	1.053	2.44	6.68	6
178	4 weeks post winter:8 weeks of winter vs. 8 weeks post winter:2 weeks of winter	24.34	2.848	21.49	6.68	6
179	4 weeks post winter:8 weeks of winter vs. 8 weeks post winter:4 weeks of winter	24.34	21.28	3.06	6.68	6
180	4 weeks post winter:8 weeks of winter vs. 8 weeks post winter:8 weeks of winter	24.34	83.32	-58.98	6.68	6

2way ANOVA<br>Multiple comparisons						
181	4 weeks post winter:8 weeks of winter vs. SPC -4 weeks post winter:2 weeks of winter	24.34	0.4471	23.89	6.68	6
182	4 weeks post winter:8 weeks of winter vs. SPC -4 weeks post winter:4 weeks of winter	24.34	0.4378	23.9	6.68	6
183	4 weeks post winter:8 weeks of winter vs. SPC -4 weeks post winter:8 weeks of winter	24.34	0.5872	23.75	6.68	6
184	4 weeks post winter:8 weeks of winter vs. SPC -8 weeks post winter:2 weeks of winter	24.34	0.6591	23.68	6.68	6
185	4 weeks post winter:8 weeks of winter vs. SPC -8 weeks post winter:4 weeks of winter	24.34	0.5872	23.75	6.68	6
186	4 weeks post winter:8 weeks of winter vs. SPC -8 weeks post winter:8 weeks of winter	24.34	1.053	23.29	6.68	6
187	8 weeks post winter:2 weeks of winter vs. 8 weeks post winter:4 weeks of winter	2.848	21.28	-18.43	6.68	6
188	8 weeks post winter:2 weeks of winter vs. 8 weeks post winter:8 weeks of winter	2.848	83.32	-80.48	6.68	6
189	8 weeks post winter:2 weeks of winter vs. SPC -4 weeks post winter:2 weeks of winter	2.848	0.4471	2.4	6.68	6
190	8 weeks post winter:2 weeks of winter vs. SPC -4 weeks post winter:4 weeks of winter	2.848	0.4378	2.41	6.68	6
191	8 weeks post winter:2 weeks of winter vs. SPC -4 weeks post winter:8 weeks of winter	2.848	0.5872	2.26	6.68	6
192	8 weeks post winter:2 weeks of winter vs. SPC -8 weeks post winter:2 weeks of winter	2.848	0.6591	2.189	6.68	6
193	8 weeks post winter:2 weeks of winter vs. SPC -8 weeks post winter:4 weeks of winter	2.848	0.5872	2.26	6.68	6
194	8 weeks post winter:2 weeks of winter vs. SPC -8 weeks post winter:8 weeks of winter	2.848	1.053	1.795	6.68	6
195	8 weeks post winter:4 weeks of winter vs. 8 weeks post winter:8 weeks of winter	21.28	83.32	-62.04	6.68	6
196	8 weeks post winter:4 weeks of winter vs. SPC -4 weeks post winter:2 weeks of winter	21.28	0.4471	20.83	6.68	6
197	8 weeks post winter:4 weeks of winter vs. SPC -4 weeks post winter:4 weeks of winter	21.28	0.4378	20.84	6.68	6
198	8 weeks post winter:4 weeks of winter vs. SPC -4 weeks post winter:8 weeks of winter	21.28	0.5872	20.69	6.68	6
199	8 weeks post winter:4 weeks of winter vs. SPC -8 weeks post winter:2 weeks of winter	21.28	0.6591	20.62	6.68	6
200	8 weeks post winter:4 weeks of winter vs. SPC -8 weeks post winter:4 weeks of winter	21.28	0.5872	20.69	6.68	6
201	8 weeks post winter:4 weeks of winter vs. SPC -8 weeks post winter:8 weeks of winter	21.28	1.053	20.23	6.68	6
202	8 weeks post winter:8 weeks of winter vs. SPC -4 weeks post winter:2 weeks of winter	83.32	0.4471	82.88	6.68	6
203	8 weeks post winter:8 weeks of winter vs. SPC -4 weeks post winter:4 weeks of winter	83.32	0.4378	82.89	6.68	6
204	8 weeks post winter:8 weeks of winter vs. SPC -4 weeks post winter:8 weeks of winter	83.32	0.5872	82.74	6.68	6
205	8 weeks post winter:8 weeks of winter vs. SPC -8 weeks post winter:2 weeks of winter	83.32	0.6591	82.66	6.68	6
206	8 weeks post winter:8 weeks of winter vs. SPC -8 weeks post winter:4 weeks of winter	83.32	0.5872	82.74	6.68	6
207	8 weeks post winter:8 weeks of winter vs. SPC -8 weeks post winter:8 weeks of winter	83.32	1.053	82.27	6.68	6
208	SPC -4 weeks post winter:2 weeks of winter vs. SPC -4 weeks post winter:4 weeks of winter	0.4471	0.4378	0.009339	6.68	6
209	SPC -4 weeks post winter:2 weeks of winter vs. SPC -4 weeks post winter:8 weeks of winter	0.4471	0.5872	-0.1401	6.68	6
210	SPC -4 weeks post winter:2 weeks of winter vs. SPC -8 weeks post winter:2 weeks of winter	0.4471	0.6591	-0.212	6.68	6

**2way ANOVA**<br>Multiple comparisons						
**211**	SPC -4 weeks post winter:2 weeks of winter vs. SPC -8 weeks post winter:4 weeks of winter	0.4471	0.5872	-0.1401	6.68	6
**212**	SPC -4 weeks post winter:2 weeks of winter vs. SPC -8 weeks post winter:8 weeks of winter	0.4471	1.053	-0.6055	6.68	6
**213**	SPC -4 weeks post winter:4 weeks of winter vs. SPC -4 weeks post winter:8 weeks of winter	0.4378	0.5872	-0.1494	6.68	6
**214**	SPC -4 weeks post winter:4 weeks of winter vs. SPC -8 weeks post winter:2 weeks of winter	0.4378	0.6591	-0.2213	6.68	6
**215**	SPC -4 weeks post winter:4 weeks of winter vs. SPC -8 weeks post winter:4 weeks of winter	0.4378	0.5872	-0.1494	6.68	6
**216**	SPC -4 weeks post winter:4 weeks of winter vs. SPC -8 weeks post winter:8 weeks of winter	0.4378	1.053	-0.6149	6.68	6
**217**	SPC -4 weeks post winter:8 weeks of winter vs. SPC -8 weeks post winter:2 weeks of winter	0.5872	0.6591	-0.07187	6.68	6
**218**	SPC -4 weeks post winter:8 weeks of winter vs. SPC -8 weeks post winter:4 weeks of winter	0.5872	0.5872	0	6.68	6
**219**	SPC -4 weeks post winter:8 weeks of winter vs. SPC -8 weeks post winter:8 weeks of winter	0.5872	1.053	-0.4654	6.68	6
**220**	SPC -8 weeks post winter:2 weeks of winter vs. SPC -8 weeks post winter:4 weeks of winter	0.6591	0.5872	0.07187	6.68	6
**221**	SPC -8 weeks post winter:2 weeks of winter vs. SPC -8 weeks post winter:8 weeks of winter	0.6591	1.053	-0.3936	6.68	6
**222**	SPC -8 weeks post winter:4 weeks of winter vs. SPC -8 weeks post winter:8 weeks of winter	0.5872	1.053	-0.4654	6.68	6

1			
2			
3			
4			
5			
6			
7			
8			
9			
10			
11			
12			
13			
14			
15			
16			
17			
18			
19			
20			
21			
22			
23			
24			
25			
26			
27			
28			
29			
30			

31			
32			
33			
34			
35			
36			
37			
38			
39			
40			
41			
42			
43			
44			
45			
46			
47			
48			
49			
50			
51			
52			
53			
54			
55			
56			
57			
58			
59			
60			

61			
62			
63			
64			
65			
66			
67			
68			
69			
70			
71			
72			
73			
74			
75			
76			
77			
78			
79			
80			
81			
82			
83			
84			
85			
86			
87			
88			
89			
90			

91			
92			
93			
94			
95			
96			
97			
98			
99			
100			
101			
102			
103			
104			
105			
106			
107			
108			
109			
110			
111			
112			
113			
114			
115			
116	N2	q	DF
117			
118	6	0.02217	75
119	6	0.03008	75
120	6	0.1536	75

**121**	6	0.6167	75
**122**	6	5.03	75
**123**	6	0.4801	75
**124**	6	4.382	75
**125**	6	17.52	75
**126**	6	0.02811	75
**127**	6	0.03008	75
**128**	6	0.001552	75
**129**	6	0.01677	75
**130**	6	0.001552	75
**131**	6	0.1001	75
**132**	6	0.007919	75
**133**	6	0.1757	75
**134**	6	0.6389	75
**135**	6	5.052	75
**136**	6	0.5023	75
**137**	6	4.404	75
**138**	6	17.54	75
**139**	6	0.005942	75
**140**	6	0.007919	75
**141**	6	0.02372	75
**142**	6	0.03893	75
**143**	6	0.02372	75
**144**	6	0.1223	75
**145**	6	0.1837	75
**146**	6	0.6468	75
**147**	6	5.06	75
**148**	6	0.5102	75
**149**	6	4.412	75
**150**	6	17.55	75

**151**	6	0.001977	75
**152**	6	0	75
**153**	6	0.03164	75
**154**	6	0.04685	75
**155**	6	0.03164	75
**156**	6	0.1302	75
**157**	6	0.4631	75
**158**	6	4.876	75
**159**	6	0.3265	75
**160**	6	4.229	75
**161**	6	17.36	75
**162**	6	0.1817	75
**163**	6	0.1837	75
**164**	6	0.152	75
**165**	6	0.1368	75
**166**	6	0.152	75
**167**	6	0.0535	75
**168**	6	4.413	75
**169**	6	0.1366	75
**170**	6	3.766	75
**171**	6	16.9	75
**172**	6	0.6448	75
**173**	6	0.6468	75
**174**	6	0.6151	75
**175**	6	0.5999	75
**176**	6	0.6151	75
**177**	6	0.5166	75
**178**	6	4.55	75
**179**	6	0.6478	75
**180**	6	12.49	75

**181**	6	5.058	75
**182**	6	5.06	75
**183**	6	5.028	75
**184**	6	5.013	75
**185**	6	5.028	75
**186**	6	4.93	75
**187**	6	3.902	75
**188**	6	17.04	75
**189**	6	0.5082	75
**190**	6	0.5102	75
**191**	6	0.4785	75
**192**	6	0.4633	75
**193**	6	0.4785	75
**194**	6	0.38	75
**195**	6	13.14	75
**196**	6	4.41	75
**197**	6	4.412	75
**198**	6	4.381	75
**199**	6	4.365	75
**200**	6	4.381	75
**201**	6	4.282	75
**202**	6	17.55	75
**203**	6	17.55	75
**204**	6	17.52	75
**205**	6	17.5	75
**206**	6	17.52	75
**207**	6	17.42	75
**208**	6	0.001977	75
**209**	6	0.02966	75
**210**	6	0.04488	75

**211**	6	0.02966	75
**212**	6	0.1282	75
**213**	6	0.03164	75
**214**	6	0.04685	75
**215**	6	0.03164	75
**216**	6	0.1302	75
**217**	6	0.01522	75
**218**	6	0	75
**219**	6	0.09853	75
**220**	6	0.01522	75
**221**	6	0.08332	75
**222**	6	0.09853	75

2way ANOVA
Tabular results

1	Table Analyzed	SLC5A7				
2						
3	Two-way ANOVA	Ordinary				
4	Alpha	0.05				
5						
6	Source of Variation	% of total variation	P value	P value summary	Significant?	
7	Interaction	16.21	<0.0001	****	Yes	
8	Time post-winter	46.38	<0.0001	****	Yes	
9	Treatment	15.81	<0.0001	****	Yes	
10						
11	ANOVA table	SS	DF	MS	F (DFn, DFd)	P value
12	Interaction	47.02	8	5.877	F (8, 75) = 7.037	P<0.0001
13	Time post-winter	134.5	4	33.63	F (4, 75) = 40.26	P<0.0001
14	Treatment	45.86	2	22.93	F (2, 75) = 27.45	P<0.0001
15	Residual	62.65	75	0.8353		
16						
17	Number of missing values	0				

2way ANOVA
Multiple comparisons

1	Compare cell means regardless of rows and columns					
2						
3	Number of families	1				
4	Number of comparisons per family	105				
5	Alpha	0.05				
6						
7	Tukey's multiple comparisons test	Mean Diff.	95.00% CI of diff.	Significant?	Summary	Adjusted P Value
8						
9	End of winter:2 weeks of winter vs. End of winter:4 weeks of winter	-0.04389	-1.894 to 1.807	No	ns	>0.9999
10	End of winter:2 weeks of winter vs. End of winter:8 weeks of winter	0.024	-1.826 to 1.875	No	ns	>0.9999
11	End of winter:2 weeks of winter vs. 4 weeks post winter:2 weeks of winter	-1.348	-3.198 to 0.5026	No	ns	0.4165
12	End of winter:2 weeks of winter vs. 4 weeks post winter:4 weeks of winter	-1.347	-3.198 to 0.5032	No	ns	0.4172
13	End of winter:2 weeks of winter vs. 4 weeks post winter:8 weeks of winter	-3.961	-5.812 to -2.111	Yes	****	<0.0001
14	End of winter:2 weeks of winter vs. 8 weeks post winter:2 weeks of winter	-1.214	-3.065 to 0.6361	No	ns	0.5926
15	End of winter:2 weeks of winter vs. 8 weeks post winter:4 weeks of winter	-2.278	-4.128 to -0.4273	Yes	**	0.0040
16	End of winter:2 weeks of winter vs. 8 weeks post winter:8 weeks of winter	-5.615	-7.466 to -3.765	Yes	****	<0.0001
17	End of winter:2 weeks of winter vs. SPC -4 weeks post winter:2 weeks of winter	0.1421	-1.708 to 1.993	No	ns	>0.9999
18	End of winter:2 weeks of winter vs. SPC -4 weeks post winter:4 weeks of winter	0.024	-1.826 to 1.875	No	ns	>0.9999
19	End of winter:2 weeks of winter vs. SPC -4 weeks post winter:8 weeks of winter	-0.4444	-2.295 to 1.406	No	ns	>0.9999
20	End of winter:2 weeks of winter vs. SPC -8 weeks post winter:2 weeks of winter	-0.3202	-2.171 to 1.53	No	ns	>0.9999
21	End of winter:2 weeks of winter vs. SPC -8 weeks post winter:4 weeks of winter	-0.4444	-2.295 to 1.406	No	ns	>0.9999
22	End of winter:2 weeks of winter vs. SPC -8 weeks post winter:8 weeks of winter	-0.8987	-2.749 to 0.9518	No	ns	0.9255
23	End of winter:4 weeks of winter vs. End of winter:8 weeks of winter	0.0679	-1.783 to 1.918	No	ns	>0.9999
24	End of winter:4 weeks of winter vs. 4 weeks post winter:2 weeks of winter	-1.304	-3.154 to 0.5465	No	ns	0.4728
25	End of winter:4 weeks of winter vs. 4 weeks post winter:4 weeks of winter	-1.303	-3.154 to 0.5471	No	ns	0.4736
26	End of winter:4 weeks of winter vs. 4 weeks post winter:8 weeks of winter	-3.917	-5.768 to -2.067	Yes	****	<0.0001
27	End of winter:4 weeks of winter vs. 8 weeks post winter:2 weeks of winter	-1.171	-3.021 to 0.68	No	ns	0.6511
28	End of winter:4 weeks of winter vs. 8 weeks post winter:4 weeks of winter	-2.234	-4.084 to -0.3834	Yes	**	0.0053
29	End of winter:4 weeks of winter vs. 8 weeks post winter:8 weeks of winter	-5.571	-7.422 to -3.721	Yes	****	<0.0001
30	End of winter:4 weeks of winter vs. SPC -4 weeks post winter:2 weeks of winter	0.186	-1.664 to 2.037	No	ns	>0.9999

2way ANOVA<br>Multiple comparisons						
31	End of winter:4 weeks of winter vs. SPC -4 weeks post winter:4 weeks of winter	0.0679	-1.783 to 1.918	No	ns	>0.9999
32	End of winter:4 weeks of winter vs. SPC -4 weeks post winter:8 weeks of winter	-0.4005	-2.251 to 1.45	No	ns	>0.9999
33	End of winter:4 weeks of winter vs. SPC -8 weeks post winter:2 weeks of winter	-0.2763	-2.127 to 1.574	No	ns	>0.9999
34	End of winter:4 weeks of winter vs. SPC -8 weeks post winter:4 weeks of winter	-0.4005	-2.251 to 1.45	No	ns	>0.9999
35	End of winter:4 weeks of winter vs. SPC -8 weeks post winter:8 weeks of winter	-0.8548	-2.705 to 0.9957	No	ns	0.9488
36	End of winter:8 weeks of winter vs. 4 weeks post winter:2 weeks of winter	-1.372	-3.222 to 0.4786	No	ns	0.3868
37	End of winter:8 weeks of winter vs. 4 weeks post winter:4 weeks of winter	-1.371	-3.222 to 0.4792	No	ns	0.3875
38	End of winter:8 weeks of winter vs. 4 weeks post winter:8 weeks of winter	-3.985	-5.836 to -2.135	Yes	****	<0.0001
39	End of winter:8 weeks of winter vs. 8 weeks post winter:2 weeks of winter	-1.238	-3.089 to 0.6121	No	ns	0.5603
40	End of winter:8 weeks of winter vs. 8 weeks post winter:4 weeks of winter	-2.302	-4.152 to -0.4513	Yes	**	0.0034
41	End of winter:8 weeks of winter vs. 8 weeks post winter:8 weeks of winter	-5.639	-7.49 to -3.789	Yes	****	<0.0001
42	End of winter:8 weeks of winter vs. SPC -4 weeks post winter:2 weeks of winter	0.1181	-1.732 to 1.969	No	ns	>0.9999
43	End of winter:8 weeks of winter vs. SPC -4 weeks post winter:4 weeks of winter	0	-1.85 to 1.85	No	ns	>0.9999
44	End of winter:8 weeks of winter vs. SPC -4 weeks post winter:8 weeks of winter	-0.4684	-2.319 to 1.382	No	ns	0.9999
45	End of winter:8 weeks of winter vs. SPC -8 weeks post winter:2 weeks of winter	-0.3442	-2.195 to 1.506	No	ns	>0.9999
46	End of winter:8 weeks of winter vs. SPC -8 weeks post winter:4 weeks of winter	-0.4684	-2.319 to 1.382	No	ns	0.9999
47	End of winter:8 weeks of winter vs. SPC -8 weeks post winter:8 weeks of winter	-0.9227	-2.773 to 0.9278	No	ns	0.9102
48	4 weeks post winter:2 weeks of winter vs. 4 weeks post winter:4 weeks of winter	0.0006112	-1.85 to 1.851	No	ns	>0.9999
49	4 weeks post winter:2 weeks of winter vs. 4 weeks post winter:8 weeks of winter	-2.613	-4.464 to -0.7628	Yes	***	0.0004
50	4 weeks post winter:2 weeks of winter vs. 8 weeks post winter:2 weeks of winter	0.1335	-1.717 to 1.984	No	ns	>0.9999
51	4 weeks post winter:2 weeks of winter vs. 8 weeks post winter:4 weeks of winter	-0.9299	-2.78 to 0.9206	No	ns	0.9052
52	4 weeks post winter:2 weeks of winter vs. 8 weeks post winter:8 weeks of winter	-4.267	-6.118 to -2.417	Yes	****	<0.0001
53	4 weeks post winter:2 weeks of winter vs. SPC -4 weeks post winter:2 weeks of winter	1.49	-0.3605 to 3.341	No	ns	0.2574
54	4 weeks post winter:2 weeks of winter vs. SPC -4 weeks post winter:4 weeks of winter	1.372	-0.4786 to 3.222	No	ns	0.3868
55	4 weeks post winter:2 weeks of winter vs. SPC -4 weeks post winter:8 weeks of winter	0.9034	-0.9471 to 2.754	No	ns	0.9226
56	4 weeks post winter:2 weeks of winter vs. SPC -8 weeks post winter:2 weeks of winter	1.028	-0.8228 to 2.878	No	ns	0.8210
57	4 weeks post winter:2 weeks of winter vs. SPC -8 weeks post winter:4 weeks of winter	0.9034	-0.9471 to 2.754	No	ns	0.9226
58	4 weeks post winter:2 weeks of winter vs. SPC -8 weeks post winter:8 weeks of winter	0.4492	-1.401 to 2.3	No	ns	>0.9999
59	4 weeks post winter:4 weeks of winter vs. 4 weeks post winter:8 weeks of winter	-2.614	-4.464 to -0.7634	Yes	***	0.0004
60	4 weeks post winter:4 weeks of winter vs. 8 weeks post winter:2 weeks of winter	0.1329	-1.718 to 1.983	No	ns	>0.9999

2way ANOVA<br>Multiple comparisons						
61	4 weeks post winter:4 weeks of winter vs. 8 weeks post winter:4 weeks of winter	-0.9305	-2.781 to 0.92	No	ns	0.9048
62	4 weeks post winter:4 weeks of winter vs. 8 weeks post winter:8 weeks of winter	-4.268	-6.118 to -2.417	Yes	****	<0.0001
63	4 weeks post winter:4 weeks of winter vs. SPC -4 weeks post winter:2 weeks of winter	1.489	-0.3611 to 3.34	No	ns	0.2580
64	4 weeks post winter:4 weeks of winter vs. SPC -4 weeks post winter:4 weeks of winter	1.371	-0.4792 to 3.222	No	ns	0.3875
65	4 weeks post winter:4 weeks of winter vs. SPC -4 weeks post winter:8 weeks of winter	0.9028	-0.9477 to 2.753	No	ns	0.9230
66	4 weeks post winter:4 weeks of winter vs. SPC -8 weeks post winter:2 weeks of winter	1.027	-0.8235 to 2.878	No	ns	0.8216
67	4 weeks post winter:4 weeks of winter vs. SPC -8 weeks post winter:4 weeks of winter	0.9028	-0.9477 to 2.753	No	ns	0.9230
68	4 weeks post winter:4 weeks of winter vs. SPC -8 weeks post winter:8 weeks of winter	0.4486	-1.402 to 2.299	No	ns	>0.9999
69	4 weeks post winter:8 weeks of winter vs. 8 weeks post winter:2 weeks of winter	2.747	0.8963 to 4.597	Yes	***	0.0002
70	4 weeks post winter:8 weeks of winter vs. 8 weeks post winter:4 weeks of winter	1.683	-0.1671 to 3.534	No	ns	0.1143
71	4 weeks post winter:8 weeks of winter vs. 8 weeks post winter:8 weeks of winter	-1.654	-3.504 to 0.1965	No	ns	0.1307
72	4 weeks post winter:8 weeks of winter vs. SPC -4 weeks post winter:2 weeks of winter	4.103	2.253 to 5.954	Yes	****	<0.0001
73	4 weeks post winter:8 weeks of winter vs. SPC -4 weeks post winter:4 weeks of winter	3.985	2.135 to 5.836	Yes	****	<0.0001
74	4 weeks post winter:8 weeks of winter vs. SPC -4 weeks post winter:8 weeks of winter	3.517	1.666 to 5.367	Yes	****	<0.0001
75	4 weeks post winter:8 weeks of winter vs. SPC -8 weeks post winter:2 weeks of winter	3.641	1.79 to 5.491	Yes	****	<0.0001
76	4 weeks post winter:8 weeks of winter vs. SPC -8 weeks post winter:4 weeks of winter	3.517	1.666 to 5.367	Yes	****	<0.0001
77	4 weeks post winter:8 weeks of winter vs. SPC -8 weeks post winter:8 weeks of winter	3.063	1.212 to 4.913	Yes	****	<0.0001
78	8 weeks post winter:2 weeks of winter vs. 8 weeks post winter:4 weeks of winter	-1.063	-2.914 to 0.7871	No	ns	0.7828
79	8 weeks post winter:2 weeks of winter vs. 8 weeks post winter:8 weeks of winter	-4.401	-6.251 to -2.55	Yes	****	<0.0001
80	8 weeks post winter:2 weeks of winter vs. SPC -4 weeks post winter:2 weeks of winter	1.357	-0.494 to 3.207	No	ns	0.4057
81	8 weeks post winter:2 weeks of winter vs. SPC -4 weeks post winter:4 weeks of winter	1.238	-0.6121 to 3.089	No	ns	0.5603
82	8 weeks post winter:2 weeks of winter vs. SPC -4 weeks post winter:8 weeks of winter	0.77	-1.081 to 2.62	No	ns	0.9782
83	8 weeks post winter:2 weeks of winter vs. SPC -8 weeks post winter:2 weeks of winter	0.8942	-0.9563 to 2.745	No	ns	0.9282
84	8 weeks post winter:2 weeks of winter vs. SPC -8 weeks post winter:4 weeks of winter	0.77	-1.081 to 2.62	No	ns	0.9782
85	8 weeks post winter:2 weeks of winter vs. SPC -8 weeks post winter:8 weeks of winter	0.3157	-1.535 to 2.166	No	ns	>0.9999
86	8 weeks post winter:4 weeks of winter vs. 8 weeks post winter:8 weeks of winter	-3.337	-5.188 to -1.487	Yes	****	<0.0001
87	8 weeks post winter:4 weeks of winter vs. SPC -4 weeks post winter:2 weeks of winter	2.42	0.5694 to 4.27	Yes	**	0.0016
88	8 weeks post winter:4 weeks of winter vs. SPC -4 weeks post winter:4 weeks of winter	2.302	0.4513 to 4.152	Yes	**	0.0034
89	8 weeks post winter:4 weeks of winter vs. SPC -4 weeks post winter:8 weeks of winter	1.833	-0.01714 to 3.684	No	ns	0.0547
90	8 weeks post winter:4 weeks of winter vs. SPC -8 weeks post winter:2 weeks of winter	1.958	0.1071 to 3.808	Yes	*	0.0279

2way ANOVA<br>Multiple comparisons						
91	8 weeks post winter:4 weeks of winter vs. SPC -8 weeks post winter:4 weeks of winter	1.833	-0.01714 to 3.684	No	ns	0.0547
92	8 weeks post winter:4 weeks of winter vs. SPC -8 weeks post winter:8 weeks of winter	1.379	-0.4714 to 3.23	No	ns	0.3781
93	8 weeks post winter:8 weeks of winter vs. SPC -4 weeks post winter:2 weeks of winter	5.757	3.907 to 7.608	Yes	****	<0.0001
94	8 weeks post winter:8 weeks of winter vs. SPC -4 weeks post winter:4 weeks of winter	5.639	3.789 to 7.49	Yes	****	<0.0001
95	8 weeks post winter:8 weeks of winter vs. SPC -4 weeks post winter:8 weeks of winter	5.171	3.32 to 7.021	Yes	****	<0.0001
96	8 weeks post winter:8 weeks of winter vs. SPC -8 weeks post winter:2 weeks of winter	5.295	3.444 to 7.145	Yes	****	<0.0001
97	8 weeks post winter:8 weeks of winter vs. SPC -8 weeks post winter:4 weeks of winter	5.171	3.32 to 7.021	Yes	****	<0.0001
98	8 weeks post winter:8 weeks of winter vs. SPC -8 weeks post winter:8 weeks of winter	4.717	2.866 to 6.567	Yes	****	<0.0001
99	SPC -4 weeks post winter:2 weeks of winter vs. SPC -4 weeks post winter:4 weeks of winter	-0.1181	-1.969 to 1.732	No	ns	>0.9999
100	SPC -4 weeks post winter:2 weeks of winter vs. SPC -4 weeks post winter:8 weeks of winter	-0.5866	-2.437 to 1.264	No	ns	0.9984
101	SPC -4 weeks post winter:2 weeks of winter vs. SPC -8 weeks post winter:2 weeks of winter	-0.4623	-2.313 to 1.388	No	ns	0.9999
102	SPC -4 weeks post winter:2 weeks of winter vs. SPC -8 weeks post winter:4 weeks of winter	-0.5866	-2.437 to 1.264	No	ns	0.9984
103	SPC -4 weeks post winter:2 weeks of winter vs. SPC -8 weeks post winter:8 weeks of winter	-1.041	-2.891 to 0.8097	No	ns	0.8074
104	SPC -4 weeks post winter:4 weeks of winter vs. SPC -4 weeks post winter:8 weeks of winter	-0.4684	-2.319 to 1.382	No	ns	0.9999
105	SPC -4 weeks post winter:4 weeks of winter vs. SPC -8 weeks post winter:2 weeks of winter	-0.3442	-2.195 to 1.506	No	ns	>0.9999
106	SPC -4 weeks post winter:4 weeks of winter vs. SPC -8 weeks post winter:4 weeks of winter	-0.4684	-2.319 to 1.382	No	ns	0.9999
107	SPC -4 weeks post winter:4 weeks of winter vs. SPC -8 weeks post winter:8 weeks of winter	-0.9227	-2.773 to 0.9278	No	ns	0.9102
108	SPC -4 weeks post winter:8 weeks of winter vs. SPC -8 weeks post winter:2 weeks of winter	0.1242	-1.726 to 1.975	No	ns	>0.9999
109	SPC -4 weeks post winter:8 weeks of winter vs. SPC -8 weeks post winter:4 weeks of winter	0	-1.85 to 1.85	No	ns	>0.9999
110	SPC -4 weeks post winter:8 weeks of winter vs. SPC -8 weeks post winter:8 weeks of winter	-0.4542	-2.305 to 1.396	No	ns	>0.9999
111	SPC -8 weeks post winter:2 weeks of winter vs. SPC -8 weeks post winter:4 weeks of winter	-0.1242	-1.975 to 1.726	No	ns	>0.9999
112	SPC -8 weeks post winter:2 weeks of winter vs. SPC -8 weeks post winter:8 weeks of winter	-0.5784	-2.429 to 1.272	No	ns	0.9987
113	SPC -8 weeks post winter:4 weeks of winter vs. SPC -8 weeks post winter:8 weeks of winter	-0.4542	-2.305 to 1.396	No	ns	>0.9999
114						
115						
116	Test details	Mean 1	Mean 2	Mean Diff.	SE of diff.	N1
117						
118	End of winter:2 weeks of winter vs. End of winter:4 weeks of winter	0.9046	0.9485	-0.04389	0.5277	6
119	End of winter:2 weeks of winter vs. End of winter:8 weeks of winter	0.9046	0.8806	0.024	0.5277	6
120	End of winter:2 weeks of winter vs. 4 weeks post winter:2 weeks of winter	0.9046	2.252	-1.348	0.5277	6

2way ANOVA<br>Multiple comparisons						
121	End of winter:2 weeks of winter vs. 4 weeks post winter:4 weeks of winter	0.9046	2.252	-1.347	0.5277	6
122	End of winter:2 weeks of winter vs. 4 weeks post winter:8 weeks of winter	0.9046	4.866	-3.961	0.5277	6
123	End of winter:2 weeks of winter vs. 8 weeks post winter:2 weeks of winter	0.9046	2.119	-1.214	0.5277	6
124	End of winter:2 weeks of winter vs. 8 weeks post winter:4 weeks of winter	0.9046	3.182	-2.278	0.5277	6
125	End of winter:2 weeks of winter vs. 8 weeks post winter:8 weeks of winter	0.9046	6.52	-5.615	0.5277	6
126	End of winter:2 weeks of winter vs. SPC -4 weeks post winter:2 weeks of winter	0.9046	0.7625	0.1421	0.5277	6
127	End of winter:2 weeks of winter vs. SPC -4 weeks post winter:4 weeks of winter	0.9046	0.8806	0.024	0.5277	6
128	End of winter:2 weeks of winter vs. SPC -4 weeks post winter:8 weeks of winter	0.9046	1.349	-0.4444	0.5277	6
129	End of winter:2 weeks of winter vs. SPC -8 weeks post winter:2 weeks of winter	0.9046	1.225	-0.3202	0.5277	6
130	End of winter:2 weeks of winter vs. SPC -8 weeks post winter:4 weeks of winter	0.9046	1.349	-0.4444	0.5277	6
131	End of winter:2 weeks of winter vs. SPC -8 weeks post winter:8 weeks of winter	0.9046	1.803	-0.8987	0.5277	6
132	End of winter:4 weeks of winter vs. End of winter:8 weeks of winter	0.9485	0.8806	0.0679	0.5277	6
133	End of winter:4 weeks of winter vs. 4 weeks post winter:2 weeks of winter	0.9485	2.252	-1.304	0.5277	6
134	End of winter:4 weeks of winter vs. 4 weeks post winter:4 weeks of winter	0.9485	2.252	-1.303	0.5277	6
135	End of winter:4 weeks of winter vs. 4 weeks post winter:8 weeks of winter	0.9485	4.866	-3.917	0.5277	6
136	End of winter:4 weeks of winter vs. 8 weeks post winter:2 weeks of winter	0.9485	2.119	-1.171	0.5277	6
137	End of winter:4 weeks of winter vs. 8 weeks post winter:4 weeks of winter	0.9485	3.182	-2.234	0.5277	6
138	End of winter:4 weeks of winter vs. 8 weeks post winter:8 weeks of winter	0.9485	6.52	-5.571	0.5277	6
139	End of winter:4 weeks of winter vs. SPC -4 weeks post winter:2 weeks of winter	0.9485	0.7625	0.186	0.5277	6
140	End of winter:4 weeks of winter vs. SPC -4 weeks post winter:4 weeks of winter	0.9485	0.8806	0.0679	0.5277	6
141	End of winter:4 weeks of winter vs. SPC -4 weeks post winter:8 weeks of winter	0.9485	1.349	-0.4005	0.5277	6
142	End of winter:4 weeks of winter vs. SPC -8 weeks post winter:2 weeks of winter	0.9485	1.225	-0.2763	0.5277	6
143	End of winter:4 weeks of winter vs. SPC -8 weeks post winter:4 weeks of winter	0.9485	1.349	-0.4005	0.5277	6
144	End of winter:4 weeks of winter vs. SPC -8 weeks post winter:8 weeks of winter	0.9485	1.803	-0.8548	0.5277	6
145	End of winter:8 weeks of winter vs. 4 weeks post winter:2 weeks of winter	0.8806	2.252	-1.372	0.5277	6
146	End of winter:8 weeks of winter vs. 4 weeks post winter:4 weeks of winter	0.8806	2.252	-1.371	0.5277	6
147	End of winter:8 weeks of winter vs. 4 weeks post winter:8 weeks of winter	0.8806	4.866	-3.985	0.5277	6
148	End of winter:8 weeks of winter vs. 8 weeks post winter:2 weeks of winter	0.8806	2.119	-1.238	0.5277	6
149	End of winter:8 weeks of winter vs. 8 weeks post winter:4 weeks of winter	0.8806	3.182	-2.302	0.5277	6
150	End of winter:8 weeks of winter vs. 8 weeks post winter:8 weeks of winter	0.8806	6.52	-5.639	0.5277	6

**2way ANOVA**<br>Multiple comparisons						
151	End of winter:8 weeks of winter vs. SPC -4 weeks post winter:2 weeks of winter	0.8806	0.7625	0.1181	0.5277	6
152	End of winter:8 weeks of winter vs. SPC -4 weeks post winter:4 weeks of winter	0.8806	0.8806	0	0.5277	6
153	End of winter:8 weeks of winter vs. SPC -4 weeks post winter:8 weeks of winter	0.8806	1.349	-0.4684	0.5277	6
154	End of winter:8 weeks of winter vs. SPC -8 weeks post winter:2 weeks of winter	0.8806	1.225	-0.3442	0.5277	6
155	End of winter:8 weeks of winter vs. SPC -8 weeks post winter:4 weeks of winter	0.8806	1.349	-0.4684	0.5277	6
156	End of winter:8 weeks of winter vs. SPC -8 weeks post winter:8 weeks of winter	0.8806	1.803	-0.9227	0.5277	6
157	4 weeks post winter:2 weeks of winter vs. 4 weeks post winter:4 weeks of winter	2.252	2.252	0.0006112	0.5277	6
158	4 weeks post winter:2 weeks of winter vs. 4 weeks post winter:8 weeks of winter	2.252	4.866	-2.613	0.5277	6
159	4 weeks post winter:2 weeks of winter vs. 8 weeks post winter:2 weeks of winter	2.252	2.119	0.1335	0.5277	6
160	4 weeks post winter:2 weeks of winter vs. 8 weeks post winter:4 weeks of winter	2.252	3.182	-0.9299	0.5277	6
161	4 weeks post winter:2 weeks of winter vs. 8 weeks post winter:8 weeks of winter	2.252	6.52	-4.267	0.5277	6
162	4 weeks post winter:2 weeks of winter vs. SPC -4 weeks post winter:2 weeks of winter	2.252	0.7625	1.49	0.5277	6
163	4 weeks post winter:2 weeks of winter vs. SPC -4 weeks post winter:4 weeks of winter	2.252	0.8806	1.372	0.5277	6
164	4 weeks post winter:2 weeks of winter vs. SPC -4 weeks post winter:8 weeks of winter	2.252	1.349	0.9034	0.5277	6
165	4 weeks post winter:2 weeks of winter vs. SPC -8 weeks post winter:2 weeks of winter	2.252	1.225	1.028	0.5277	6
166	4 weeks post winter:2 weeks of winter vs. SPC -8 weeks post winter:4 weeks of winter	2.252	1.349	0.9034	0.5277	6
167	4 weeks post winter:2 weeks of winter vs. SPC -8 weeks post winter:8 weeks of winter	2.252	1.803	0.4492	0.5277	6
168	4 weeks post winter:4 weeks of winter vs. 4 weeks post winter:8 weeks of winter	2.252	4.866	-2.614	0.5277	6
169	4 weeks post winter:4 weeks of winter vs. 8 weeks post winter:2 weeks of winter	2.252	2.119	0.1329	0.5277	6
170	4 weeks post winter:4 weeks of winter vs. 8 weeks post winter:4 weeks of winter	2.252	3.182	-0.9305	0.5277	6
171	4 weeks post winter:4 weeks of winter vs. 8 weeks post winter:8 weeks of winter	2.252	6.52	-4.268	0.5277	6
172	4 weeks post winter:4 weeks of winter vs. SPC -4 weeks post winter:2 weeks of winter	2.252	0.7625	1.489	0.5277	6
173	4 weeks post winter:4 weeks of winter vs. SPC -4 weeks post winter:4 weeks of winter	2.252	0.8806	1.371	0.5277	6
174	4 weeks post winter:4 weeks of winter vs. SPC -4 weeks post winter:8 weeks of winter	2.252	1.349	0.9028	0.5277	6
175	4 weeks post winter:4 weeks of winter vs. SPC -8 weeks post winter:2 weeks of winter	2.252	1.225	1.027	0.5277	6
176	4 weeks post winter:4 weeks of winter vs. SPC -8 weeks post winter:4 weeks of winter	2.252	1.349	0.9028	0.5277	6
177	4 weeks post winter:4 weeks of winter vs. SPC -8 weeks post winter:8 weeks of winter	2.252	1.803	0.4486	0.5277	6
178	4 weeks post winter:8 weeks of winter vs. 8 weeks post winter:2 weeks of winter	4.866	2.119	2.747	0.5277	6
179	4 weeks post winter:8 weeks of winter vs. 8 weeks post winter:4 weeks of winter	4.866	3.182	1.683	0.5277	6
180	4 weeks post winter:8 weeks of winter vs. 8 weeks post winter:8 weeks of winter	4.866	6.52	-1.654	0.5277	6

2way ANOVA<br>Multiple comparisons						
181	4 weeks post winter:8 weeks of winter vs. SPC -4 weeks post winter:2 weeks of winter	4.866	0.7625	4.103	0.5277	6
182	4 weeks post winter:8 weeks of winter vs. SPC -4 weeks post winter:4 weeks of winter	4.866	0.8806	3.985	0.5277	6
183	4 weeks post winter:8 weeks of winter vs. SPC -4 weeks post winter:8 weeks of winter	4.866	1.349	3.517	0.5277	6
184	4 weeks post winter:8 weeks of winter vs. SPC -8 weeks post winter:2 weeks of winter	4.866	1.225	3.641	0.5277	6
185	4 weeks post winter:8 weeks of winter vs. SPC -8 weeks post winter:4 weeks of winter	4.866	1.349	3.517	0.5277	6
186	4 weeks post winter:8 weeks of winter vs. SPC -8 weeks post winter:8 weeks of winter	4.866	1.803	3.063	0.5277	6
187	8 weeks post winter:2 weeks of winter vs. 8 weeks post winter:4 weeks of winter	2.119	3.182	-1.063	0.5277	6
188	8 weeks post winter:2 weeks of winter vs. 8 weeks post winter:8 weeks of winter	2.119	6.52	-4.401	0.5277	6
189	8 weeks post winter:2 weeks of winter vs. SPC -4 weeks post winter:2 weeks of winter	2.119	0.7625	1.357	0.5277	6
190	8 weeks post winter:2 weeks of winter vs. SPC -4 weeks post winter:4 weeks of winter	2.119	0.8806	1.238	0.5277	6
191	8 weeks post winter:2 weeks of winter vs. SPC -4 weeks post winter:8 weeks of winter	2.119	1.349	0.77	0.5277	6
192	8 weeks post winter:2 weeks of winter vs. SPC -8 weeks post winter:2 weeks of winter	2.119	1.225	0.8942	0.5277	6
193	8 weeks post winter:2 weeks of winter vs. SPC -8 weeks post winter:4 weeks of winter	2.119	1.349	0.77	0.5277	6
194	8 weeks post winter:2 weeks of winter vs. SPC -8 weeks post winter:8 weeks of winter	2.119	1.803	0.3157	0.5277	6
195	8 weeks post winter:4 weeks of winter vs. 8 weeks post winter:8 weeks of winter	3.182	6.52	-3.337	0.5277	6
196	8 weeks post winter:4 weeks of winter vs. SPC -4 weeks post winter:2 weeks of winter	3.182	0.7625	2.42	0.5277	6
197	8 weeks post winter:4 weeks of winter vs. SPC -4 weeks post winter:4 weeks of winter	3.182	0.8806	2.302	0.5277	6
198	8 weeks post winter:4 weeks of winter vs. SPC -4 weeks post winter:8 weeks of winter	3.182	1.349	1.833	0.5277	6
199	8 weeks post winter:4 weeks of winter vs. SPC -8 weeks post winter:2 weeks of winter	3.182	1.225	1.958	0.5277	6
200	8 weeks post winter:4 weeks of winter vs. SPC -8 weeks post winter:4 weeks of winter	3.182	1.349	1.833	0.5277	6
201	8 weeks post winter:4 weeks of winter vs. SPC -8 weeks post winter:8 weeks of winter	3.182	1.803	1.379	0.5277	6
202	8 weeks post winter:8 weeks of winter vs. SPC -4 weeks post winter:2 weeks of winter	6.52	0.7625	5.757	0.5277	6
203	8 weeks post winter:8 weeks of winter vs. SPC -4 weeks post winter:4 weeks of winter	6.52	0.8806	5.639	0.5277	6
204	8 weeks post winter:8 weeks of winter vs. SPC -4 weeks post winter:8 weeks of winter	6.52	1.349	5.171	0.5277	6
205	8 weeks post winter:8 weeks of winter vs. SPC -8 weeks post winter:2 weeks of winter	6.52	1.225	5.295	0.5277	6
206	8 weeks post winter:8 weeks of winter vs. SPC -8 weeks post winter:4 weeks of winter	6.52	1.349	5.171	0.5277	6
207	8 weeks post winter:8 weeks of winter vs. SPC -8 weeks post winter:8 weeks of winter	6.52	1.803	4.717	0.5277	6
208	SPC -4 weeks post winter:2 weeks of winter vs. SPC -4 weeks post winter:4 weeks of winter	0.7625	0.8806	-0.1181	0.5277	6
209	SPC -4 weeks post winter:2 weeks of winter vs. SPC -4 weeks post winter:8 weeks of winter	0.7625	1.349	-0.5866	0.5277	6
210	SPC -4 weeks post winter:2 weeks of winter vs. SPC -8 weeks post winter:2 weeks of winter	0.7625	1.225	-0.4623	0.5277	6

2way ANOVA<br>Multiple comparisons						
211	SPC -4 weeks post winter:2 weeks of winter vs. SPC -8 weeks post winter:4 weeks of winter	0.7625	1.349	-0.5866	0.5277	6
212	SPC -4 weeks post winter:2 weeks of winter vs. SPC -8 weeks post winter:8 weeks of winter	0.7625	1.803	-1.041	0.5277	6
213	SPC -4 weeks post winter:4 weeks of winter vs. SPC -4 weeks post winter:8 weeks of winter	0.8806	1.349	-0.4684	0.5277	6
214	SPC -4 weeks post winter:4 weeks of winter vs. SPC -8 weeks post winter:2 weeks of winter	0.8806	1.225	-0.3442	0.5277	6
215	SPC -4 weeks post winter:4 weeks of winter vs. SPC -8 weeks post winter:4 weeks of winter	0.8806	1.349	-0.4684	0.5277	6
216	SPC -4 weeks post winter:4 weeks of winter vs. SPC -8 weeks post winter:8 weeks of winter	0.8806	1.803	-0.9227	0.5277	6
217	SPC -4 weeks post winter:8 weeks of winter vs. SPC -8 weeks post winter:2 weeks of winter	1.349	1.225	0.1242	0.5277	6
218	SPC -4 weeks post winter:8 weeks of winter vs. SPC -8 weeks post winter:4 weeks of winter	1.349	1.349	0	0.5277	6
219	SPC -4 weeks post winter:8 weeks of winter vs. SPC -8 weeks post winter:8 weeks of winter	1.349	1.803	-0.4542	0.5277	6
220	SPC -8 weeks post winter:2 weeks of winter vs. SPC -8 weeks post winter:4 weeks of winter	1.225	1.349	-0.1242	0.5277	6
221	SPC -8 weeks post winter:2 weeks of winter vs. SPC -8 weeks post winter:8 weeks of winter	1.225	1.803	-0.5784	0.5277	6
222	SPC -8 weeks post winter:4 weeks of winter vs. SPC -8 weeks post winter:8 weeks of winter	1.349	1.803	-0.4542	0.5277	6

1			
2			
3			
4			
5			
6			
7			
8			
9			
10			
11			
12			
13			
14			
15			
16			
17			
18			
19			
20			
21			
22			
23			
24			
25			
26			
27			
28			
29			
30			

31			
32			
33			
34			
35			
36			
37			
38			
39			
40			
41			
42			
43			
44			
45			
46			
47			
48			
49			
50			
51			
52			
53			
54			
55			
56			
57			
58			
59			
60			

61			
62			
63			
64			
65			
66			
67			
68			
69			
70			
71			
72			
73			
74			
75			
76			
77			
78			
79			
80			
81			
82			
83			
84			
85			
86			
87			
88			
89			
90			

91			
92			
93			
94			
95			
96			
97			
98			
99			
100			
101			
102			
103			
104			
105			
106			
107			
108			
109			
110			
111			
112			
113			
114			
115			
116	N2	q	DF
117			
118	6	0.1176	75
119	6	0.06434	75
120	6	3.613	75

**121**	6	3.611	75
**122**	6	10.62	75
**123**	6	3.255	75
**124**	6	6.105	75
**125**	6	15.05	75
**126**	6	0.3809	75
**127**	6	0.06434	75
**128**	6	1.191	75
**129**	6	0.8583	75
**130**	6	1.191	75
**131**	6	2.409	75
**132**	6	0.182	75
**133**	6	3.495	75
**134**	6	3.493	75
**135**	6	10.5	75
**136**	6	3.137	75
**137**	6	5.987	75
**138**	6	14.93	75
**139**	6	0.4985	75
**140**	6	0.182	75
**141**	6	1.074	75
**142**	6	0.7407	75
**143**	6	1.074	75
**144**	6	2.291	75
**145**	6	3.677	75
**146**	6	3.675	75
**147**	6	10.68	75
**148**	6	3.319	75
**149**	6	6.169	75
**150**	6	15.11	75

**151**	6	0.3165	75
**152**	6	0	75
**153**	6	1.256	75
**154**	6	0.9226	75
**155**	6	1.256	75
**156**	6	2.473	75
**157**	6	0.001638	75
**158**	6	7.004	75
**159**	6	0.3578	75
**160**	6	2.492	75
**161**	6	11.44	75
**162**	6	3.993	75
**163**	6	3.677	75
**164**	6	2.421	75
**165**	6	2.754	75
**166**	6	2.421	75
**167**	6	1.204	75
**168**	6	7.006	75
**169**	6	0.3561	75
**170**	6	2.494	75
**171**	6	11.44	75
**172**	6	3.992	75
**173**	6	3.675	75
**174**	6	2.42	75
**175**	6	2.753	75
**176**	6	2.42	75
**177**	6	1.202	75
**178**	6	7.362	75
**179**	6	4.512	75
**180**	6	4.433	75

**181**	6	11	75
**182**	6	10.68	75
**183**	6	9.426	75
**184**	6	9.758	75
**185**	6	9.426	75
**186**	6	8.208	75
**187**	6	2.85	75
**188**	6	11.79	75
**189**	6	3.636	75
**190**	6	3.319	75
**191**	6	2.064	75
**192**	6	2.397	75
**193**	6	2.064	75
**194**	6	0.8462	75
**195**	6	8.945	75
**196**	6	6.486	75
**197**	6	6.169	75
**198**	6	4.914	75
**199**	6	5.247	75
**200**	6	4.914	75
**201**	6	3.696	75
**202**	6	15.43	75
**203**	6	15.11	75
**204**	6	13.86	75
**205**	6	14.19	75
**206**	6	13.86	75
**207**	6	12.64	75
**208**	6	0.3165	75
**209**	6	1.572	75
**210**	6	1.239	75

**211**	6	1.572	75
**212**	6	2.789	75
**213**	6	1.256	75
**214**	6	0.9226	75
**215**	6	1.256	75
**216**	6	2.473	75
**217**	6	0.3329	75
**218**	6	0	75
**219**	6	1.217	75
**220**	6	0.3329	75
**221**	6	1.55	75
**222**	6	1.217	75

2way ANOVA
Tabular results

1	Table Analyzed	FKBP5				
2						
3	Two-way ANOVA	Ordinary				
4	Alpha	0.05				
5						
6	Source of Variation	% of total variation	P value	P value summary	Significant?	
7	Interaction	19.42	0.0004	***	Yes	
8	Time post-winter	15.81	0.0001	***	Yes	
9	Treatment	20.52	<0.0001	****	Yes	
10						
11	ANOVA table	SS	DF	MS	F (DFn, DFd)	P value
12	Interaction	13.33	8	1.667	F (8, 75) = 4.114	P=0.0004
13	Time post-winter	10.86	4	2.714	F (4, 75) = 6.699	P=0.0001
14	Treatment	14.09	2	7.045	F (2, 75) = 17.39	P<0.0001
15	Residual	30.38	75	0.4051		
16						
17	Number of missing values	0				

2way ANOVA
Multiple comparisons

1	Compare cell means regardless of rows and columns					
2						
3	Number of families	1				
4	Number of comparisons per family	105				
5	Alpha	0.05				
6						
7	Tukey's multiple comparisons test	Mean Diff.	95.00% CI of diff.	Significant?	Summary	Adjusted P Value
8						
9	End of winter:2 weeks of winter vs. End of winter:4 weeks of winter	0.2969	-0.9919 to 1.586	No	ns	>0.9999
10	End of winter:2 weeks of winter vs. End of winter:8 weeks of winter	-0.3618	-1.651 to 0.927	No	ns	0.9996
11	End of winter:2 weeks of winter vs. 4 weeks post winter:2 weeks of winter	0.1339	-1.155 to 1.423	No	ns	>0.9999
12	End of winter:2 weeks of winter vs. 4 weeks post winter:4 weeks of winter	-0.6927	-1.981 to 0.5961	No	ns	0.8530
13	End of winter:2 weeks of winter vs. 4 weeks post winter:8 weeks of winter	-2.14	-3.429 to -0.8512	Yes	****	<0.0001
14	End of winter:2 weeks of winter vs. 8 weeks post winter:2 weeks of winter	0.162	-1.127 to 1.451	No	ns	>0.9999
15	End of winter:2 weeks of winter vs. 8 weeks post winter:4 weeks of winter	-0.914	-2.203 to 0.3747	No	ns	0.4618
16	End of winter:2 weeks of winter vs. 8 weeks post winter:8 weeks of winter	-1.605	-2.893 to -0.3159	Yes	**	0.0034
17	End of winter:2 weeks of winter vs. SPC -4 weeks post winter:2 weeks of winter	0.07709	-1.212 to 1.366	No	ns	>0.9999
18	End of winter:2 weeks of winter vs. SPC -4 weeks post winter:4 weeks of winter	-0.3618	-1.651 to 0.927	No	ns	0.9996
19	End of winter:2 weeks of winter vs. SPC -4 weeks post winter:8 weeks of winter	-0.21	-1.499 to 1.079	No	ns	>0.9999
20	End of winter:2 weeks of winter vs. SPC -8 weeks post winter:2 weeks of winter	-0.2662	-1.555 to 1.023	No	ns	>0.9999
21	End of winter:2 weeks of winter vs. SPC -8 weeks post winter:4 weeks of winter	-0.21	-1.499 to 1.079	No	ns	>0.9999
22	End of winter:2 weeks of winter vs. SPC -8 weeks post winter:8 weeks of winter	-0.398	-1.687 to 0.8907	No	ns	0.9988
23	End of winter:4 weeks of winter vs. End of winter:8 weeks of winter	-0.6587	-1.947 to 0.6301	No	ns	0.8937
24	End of winter:4 weeks of winter vs. 4 weeks post winter:2 weeks of winter	-0.163	-1.452 to 1.126	No	ns	>0.9999
25	End of winter:4 weeks of winter vs. 4 weeks post winter:4 weeks of winter	-0.9896	-2.278 to 0.2992	No	ns	0.3295
26	End of winter:4 weeks of winter vs. 4 weeks post winter:8 weeks of winter	-2.437	-3.726 to -1.148	Yes	****	<0.0001
27	End of winter:4 weeks of winter vs. 8 weeks post winter:2 weeks of winter	-0.135	-1.424 to 1.154	No	ns	>0.9999
28	End of winter:4 weeks of winter vs. 8 weeks post winter:4 weeks of winter	-1.211	-2.5 to 0.07783	No	ns	0.0880
29	End of winter:4 weeks of winter vs. 8 weeks post winter:8 weeks of winter	-1.902	-3.19 to -0.6129	Yes	***	0.0002
30	End of winter:4 weeks of winter vs. SPC -4 weeks post winter:2 weeks of winter	-0.2198	-1.509 to 1.069	No	ns	>0.9999

2way ANOVA<br>Multiple comparisons						
31	End of winter:4 weeks of winter vs. SPC -4 weeks post winter:4 weeks of winter	-0.6587	-1.947 to 0.6301	No	ns	0.8937
32	End of winter:4 weeks of winter vs. SPC -4 weeks post winter:8 weeks of winter	-0.5069	-1.796 to 0.7819	No	ns	0.9868
33	End of winter:4 weeks of winter vs. SPC -8 weeks post winter:2 weeks of winter	-0.5631	-1.852 to 0.7256	No	ns	0.9671
34	End of winter:4 weeks of winter vs. SPC -8 weeks post winter:4 weeks of winter	-0.5069	-1.796 to 0.7819	No	ns	0.9868
35	End of winter:4 weeks of winter vs. SPC -8 weeks post winter:8 weeks of winter	-0.6949	-1.984 to 0.5938	No	ns	0.8499
36	End of winter:8 weeks of winter vs. 4 weeks post winter:2 weeks of winter	0.4957	-0.793 to 1.784	No	ns	0.9892
37	End of winter:8 weeks of winter vs. 4 weeks post winter:4 weeks of winter	-0.3309	-1.62 to 0.9579	No	ns	0.9999
38	End of winter:8 weeks of winter vs. 4 weeks post winter:8 weeks of winter	-1.778	-3.067 to -0.4894	Yes	***	0.0006
39	End of winter:8 weeks of winter vs. 8 weeks post winter:2 weeks of winter	0.5237	-0.765 to 1.812	No	ns	0.9823
40	End of winter:8 weeks of winter vs. 8 weeks post winter:4 weeks of winter	-0.5522	-1.841 to 0.7365	No	ns	0.9720
41	End of winter:8 weeks of winter vs. 8 weeks post winter:8 weeks of winter	-1.243	-2.532 to 0.04583	No	ns	0.0701
42	End of winter:8 weeks of winter vs. SPC -4 weeks post winter:2 weeks of winter	0.4389	-0.8499 to 1.728	No	ns	0.9967
43	End of winter:8 weeks of winter vs. SPC -4 weeks post winter:4 weeks of winter	0	-1.289 to 1.289	No	ns	>0.9999
44	End of winter:8 weeks of winter vs. SPC -4 weeks post winter:8 weeks of winter	0.1518	-1.137 to 1.441	No	ns	>0.9999
45	End of winter:8 weeks of winter vs. SPC -8 weeks post winter:2 weeks of winter	0.09557	-1.193 to 1.384	No	ns	>0.9999
46	End of winter:8 weeks of winter vs. SPC -8 weeks post winter:4 weeks of winter	0.1518	-1.137 to 1.441	No	ns	>0.9999
47	End of winter:8 weeks of winter vs. SPC -8 weeks post winter:8 weeks of winter	-0.03626	-1.325 to 1.253	No	ns	>0.9999
48	4 weeks post winter:2 weeks of winter vs. 4 weeks post winter:4 weeks of winter	-0.8266	-2.115 to 0.4622	No	ns	0.6295
49	4 weeks post winter:2 weeks of winter vs. 4 weeks post winter:8 weeks of winter	-2.274	-3.563 to -0.9851	Yes	****	<0.0001
50	4 weeks post winter:2 weeks of winter vs. 8 weeks post winter:2 weeks of winter	0.02801	-1.261 to 1.317	No	ns	>0.9999
51	4 weeks post winter:2 weeks of winter vs. 8 weeks post winter:4 weeks of winter	-1.048	-2.337 to 0.2408	No	ns	0.2434
52	4 weeks post winter:2 weeks of winter vs. 8 weeks post winter:8 weeks of winter	-1.739	-3.027 to -0.4499	Yes	***	0.0009
53	4 weeks post winter:2 weeks of winter vs. SPC -4 weeks post winter:2 weeks of winter	-0.05685	-1.346 to 1.232	No	ns	>0.9999
54	4 weeks post winter:2 weeks of winter vs. SPC -4 weeks post winter:4 weeks of winter	-0.4957	-1.784 to 0.793	No	ns	0.9892
55	4 weeks post winter:2 weeks of winter vs. SPC -4 weeks post winter:8 weeks of winter	-0.3439	-1.633 to 0.9448	No	ns	0.9998
56	4 weeks post winter:2 weeks of winter vs. SPC -8 weeks post winter:2 weeks of winter	-0.4001	-1.689 to 0.8886	No	ns	0.9987
57	4 weeks post winter:2 weeks of winter vs. SPC -8 weeks post winter:4 weeks of winter	-0.3439	-1.633 to 0.9448	No	ns	0.9998
58	4 weeks post winter:2 weeks of winter vs. SPC -8 weeks post winter:8 weeks of winter	-0.532	-1.821 to 0.7568	No	ns	0.9797
59	4 weeks post winter:4 weeks of winter vs. 4 weeks post winter:8 weeks of winter	-1.447	-2.736 to -0.1585	Yes	*	0.0139
60	4 weeks post winter:4 weeks of winter vs. 8 weeks post winter:2 weeks of winter	0.8546	-0.4342 to 2.143	No	ns	0.5755

**2way ANOVA** Multiple comparisons						
61	4 weeks post winter:4 weeks of winter vs. 8 weeks post winter:4 weeks of winter	-0.2214	-1.51 to 1.067	No	ns	>0.9999
62	4 weeks post winter:4 weeks of winter vs. 8 weeks post winter:8 weeks of winter	-0.9121	-2.201 to 0.3767	No	ns	0.4655
63	4 weeks post winter:4 weeks of winter vs. SPC -4 weeks post winter:2 weeks of winter	0.7697	-0.519 to 2.059	No	ns	0.7340
64	4 weeks post winter:4 weeks of winter vs. SPC -4 weeks post winter:4 weeks of winter	0.3309	-0.9579 to 1.62	No	ns	0.9999
65	4 weeks post winter:4 weeks of winter vs. SPC -4 weeks post winter:8 weeks of winter	0.4827	-0.8061 to 1.771	No	ns	0.9916
66	4 weeks post winter:4 weeks of winter vs. SPC -8 weeks post winter:2 weeks of winter	0.4264	-0.8623 to 1.715	No	ns	0.9975
67	4 weeks post winter:4 weeks of winter vs. SPC -8 weeks post winter:4 weeks of winter	0.4827	-0.8061 to 1.771	No	ns	0.9916
68	4 weeks post winter:4 weeks of winter vs. SPC -8 weeks post winter:8 weeks of winter	0.2946	-0.9941 to 1.583	No	ns	>0.9999
69	4 weeks post winter:8 weeks of winter vs. 8 weeks post winter:2 weeks of winter	2.302	1.013 to 3.591	Yes	****	<0.0001
70	4 weeks post winter:8 weeks of winter vs. 8 weeks post winter:4 weeks of winter	1.226	-0.06285 to 2.515	No	ns	0.0792
71	4 weeks post winter:8 weeks of winter vs. 8 weeks post winter:8 weeks of winter	0.5352	-0.7535 to 1.824	No	ns	0.9786
72	4 weeks post winter:8 weeks of winter vs. SPC -4 weeks post winter:2 weeks of winter	2.217	0.9283 to 3.506	Yes	****	<0.0001
73	4 weeks post winter:8 weeks of winter vs. SPC -4 weeks post winter:4 weeks of winter	1.778	0.4894 to 3.067	Yes	***	0.0006
74	4 weeks post winter:8 weeks of winter vs. SPC -4 weeks post winter:8 weeks of winter	1.93	0.6412 to 3.219	Yes	***	0.0001
75	4 weeks post winter:8 weeks of winter vs. SPC -8 weeks post winter:2 weeks of winter	1.874	0.585 to 3.162	Yes	***	0.0002
76	4 weeks post winter:8 weeks of winter vs. SPC -8 weeks post winter:4 weeks of winter	1.93	0.6412 to 3.219	Yes	***	0.0001
77	4 weeks post winter:8 weeks of winter vs. SPC -8 weeks post winter:8 weeks of winter	1.742	0.4531 to 3.031	Yes	***	0.0009
78	8 weeks post winter:2 weeks of winter vs. 8 weeks post winter:4 weeks of winter	-1.076	-2.365 to 0.2128	No	ns	0.2079
79	8 weeks post winter:2 weeks of winter vs. 8 weeks post winter:8 weeks of winter	-1.767	-3.055 to -0.4779	Yes	***	0.0007
80	8 weeks post winter:2 weeks of winter vs. SPC -4 weeks post winter:2 weeks of winter	-0.08486	-1.374 to 1.204	No	ns	>0.9999
81	8 weeks post winter:2 weeks of winter vs. SPC -4 weeks post winter:4 weeks of winter	-0.5237	-1.812 to 0.765	No	ns	0.9823
82	8 weeks post winter:2 weeks of winter vs. SPC -4 weeks post winter:8 weeks of winter	-0.372	-1.661 to 0.9168	No	ns	0.9994
83	8 weeks post winter:2 weeks of winter vs. SPC -8 weeks post winter:2 weeks of winter	-0.4282	-1.717 to 0.8606	No	ns	0.9974
84	8 weeks post winter:2 weeks of winter vs. SPC -8 weeks post winter:4 weeks of winter	-0.372	-1.661 to 0.9168	No	ns	0.9994
85	8 weeks post winter:2 weeks of winter vs. SPC -8 weeks post winter:8 weeks of winter	-0.56	-1.849 to 0.7288	No	ns	0.9686
86	8 weeks post winter:4 weeks of winter vs. 8 weeks post winter:8 weeks of winter	-0.6907	-1.979 to 0.5981	No	ns	0.8555
87	8 weeks post winter:4 weeks of winter vs. SPC -4 weeks post winter:2 weeks of winter	0.9911	-0.2976 to 2.28	No	ns	0.3270
88	8 weeks post winter:4 weeks of winter vs. SPC -4 weeks post winter:4 weeks of winter	0.5522	-0.7365 to 1.841	No	ns	0.9720
89	8 weeks post winter:4 weeks of winter vs. SPC -4 weeks post winter:8 weeks of winter	0.704	-0.5847 to 1.993	No	ns	0.8376
90	8 weeks post winter:4 weeks of winter vs. SPC -8 weeks post winter:2 weeks of winter	0.6478	-0.6409 to 1.937	No	ns	0.9050

2way ANOVA
Multiple comparisons

91	8 weeks post winter:4 weeks of winter vs. SPC -8 weeks post winter:4 weeks of winter	0.704	-0.5847 to 1.993	No	ns	0.8376
92	8 weeks post winter:4 weeks of winter vs. SPC -8 weeks post winter:8 weeks of winter	0.516	-0.7728 to 1.805	No	ns	0.9845
93	8 weeks post winter:8 weeks of winter vs. SPC -4 weeks post winter:2 weeks of winter	1.682	0.393 to 2.971	Yes	**	0.0016
94	8 weeks post winter:8 weeks of winter vs. SPC -4 weeks post winter:4 weeks of winter	1.243	-0.04583 to 2.532	No	ns	0.0701
95	8 weeks post winter:8 weeks of winter vs. SPC -4 weeks post winter:8 weeks of winter	1.395	0.1059 to 2.683	Yes	*	0.0216
96	8 weeks post winter:8 weeks of winter vs. SPC -8 weeks post winter:2 weeks of winter	1.339	0.04974 to 2.627	Yes	*	0.0341
97	8 weeks post winter:8 weeks of winter vs. SPC -8 weeks post winter:4 weeks of winter	1.395	0.1059 to 2.683	Yes	*	0.0216
98	8 weeks post winter:8 weeks of winter vs. SPC -8 weeks post winter:8 weeks of winter	1.207	-0.08209 to 2.495	No	ns	0.0906
99	SPC -4 weeks post winter:2 weeks of winter vs. SPC -4 weeks post winter:4 weeks of winter	-0.4389	-1.728 to 0.8499	No	ns	0.9967
100	SPC -4 weeks post winter:2 weeks of winter vs. SPC -4 weeks post winter:8 weeks of winter	-0.2871	-1.576 to 1.002	No	ns	>0.9999
101	SPC -4 weeks post winter:2 weeks of winter vs. SPC -8 weeks post winter:2 weeks of winter	-0.3433	-1.632 to 0.9455	No	ns	0.9998
102	SPC -4 weeks post winter:2 weeks of winter vs. SPC -8 weeks post winter:4 weeks of winter	-0.2871	-1.576 to 1.002	No	ns	>0.9999
103	SPC -4 weeks post winter:2 weeks of winter vs. SPC -8 weeks post winter:8 weeks of winter	-0.4751	-1.764 to 0.8136	No	ns	0.9928
104	SPC -4 weeks post winter:4 weeks of winter vs. SPC -4 weeks post winter:8 weeks of winter	0.1518	-1.137 to 1.441	No	ns	>0.9999
105	SPC -4 weeks post winter:4 weeks of winter vs. SPC -8 weeks post winter:2 weeks of winter	0.09557	-1.193 to 1.384	No	ns	>0.9999
106	SPC -4 weeks post winter:4 weeks of winter vs. SPC -8 weeks post winter:4 weeks of winter	0.1518	-1.137 to 1.441	No	ns	>0.9999
107	SPC -4 weeks post winter:4 weeks of winter vs. SPC -8 weeks post winter:8 weeks of winter	-0.03626	-1.325 to 1.253	No	ns	>0.9999
108	SPC -4 weeks post winter:8 weeks of winter vs. SPC -8 weeks post winter:2 weeks of winter	-0.05621	-1.345 to 1.233	No	ns	>0.9999
109	SPC -4 weeks post winter:8 weeks of winter vs. SPC -8 weeks post winter:4 weeks of winter	0	-1.289 to 1.289	No	ns	>0.9999
110	SPC -4 weeks post winter:8 weeks of winter vs. SPC -8 weeks post winter:8 weeks of winter	-0.188	-1.477 to 1.101	No	ns	>0.9999
111	SPC -8 weeks post winter:2 weeks of winter vs. SPC -8 weeks post winter:4 weeks of winter	0.05621	-1.233 to 1.345	No	ns	>0.9999
112	SPC -8 weeks post winter:2 weeks of winter vs. SPC -8 weeks post winter:8 weeks of winter	-0.1318	-1.421 to 1.157	No	ns	>0.9999
113	SPC -8 weeks post winter:4 weeks of winter vs. SPC -8 weeks post winter:8 weeks of winter	-0.188	-1.477 to 1.101	No	ns	>0.9999
114						
115						
116	Test details	Mean 1	Mean 2	Mean Diff.	SE of diff.	N1
117						
118	End of winter:2 weeks of winter vs. End of winter:4 weeks of winter	1.041	0.7437	0.2969	0.3675	6
119	End of winter:2 weeks of winter vs. End of winter:8 weeks of winter	1.041	1.402	-0.3618	0.3675	6
120	End of winter:2 weeks of winter vs. 4 weeks post winter:2 weeks of winter	1.041	0.9066	0.1339	0.3675	6

2way ANOVA<br>Multiple comparisons						
121	End of winter:2 weeks of winter vs. 4 weeks post winter:4 weeks of winter	1.041	1.733	-0.6927	0.3675	6
122	End of winter:2 weeks of winter vs. 4 weeks post winter:8 weeks of winter	1.041	3.181	-2.14	0.3675	6
123	End of winter:2 weeks of winter vs. 8 weeks post winter:2 weeks of winter	1.041	0.8786	0.162	0.3675	6
124	End of winter:2 weeks of winter vs. 8 weeks post winter:4 weeks of winter	1.041	1.955	-0.914	0.3675	6
125	End of winter:2 weeks of winter vs. 8 weeks post winter:8 weeks of winter	1.041	2.645	-1.605	0.3675	6
126	End of winter:2 weeks of winter vs. SPC -4 weeks post winter:2 weeks of winter	1.041	0.9635	0.07709	0.3675	6
127	End of winter:2 weeks of winter vs. SPC -4 weeks post winter:4 weeks of winter	1.041	1.402	-0.3618	0.3675	6
128	End of winter:2 weeks of winter vs. SPC -4 weeks post winter:8 weeks of winter	1.041	1.251	-0.21	0.3675	6
129	End of winter:2 weeks of winter vs. SPC -8 weeks post winter:2 weeks of winter	1.041	1.307	-0.2662	0.3675	6
130	End of winter:2 weeks of winter vs. SPC -8 weeks post winter:4 weeks of winter	1.041	1.251	-0.21	0.3675	6
131	End of winter:2 weeks of winter vs. SPC -8 weeks post winter:8 weeks of winter	1.041	1.439	-0.398	0.3675	6
132	End of winter:4 weeks of winter vs. End of winter:8 weeks of winter	0.7437	1.402	-0.6587	0.3675	6
133	End of winter:4 weeks of winter vs. 4 weeks post winter:2 weeks of winter	0.7437	0.9066	-0.163	0.3675	6
134	End of winter:4 weeks of winter vs. 4 weeks post winter:4 weeks of winter	0.7437	1.733	-0.9896	0.3675	6
135	End of winter:4 weeks of winter vs. 4 weeks post winter:8 weeks of winter	0.7437	3.181	-2.437	0.3675	6
136	End of winter:4 weeks of winter vs. 8 weeks post winter:2 weeks of winter	0.7437	0.8786	-0.135	0.3675	6
137	End of winter:4 weeks of winter vs. 8 weeks post winter:4 weeks of winter	0.7437	1.955	-1.211	0.3675	6
138	End of winter:4 weeks of winter vs. 8 weeks post winter:8 weeks of winter	0.7437	2.645	-1.902	0.3675	6
139	End of winter:4 weeks of winter vs. SPC -4 weeks post winter:2 weeks of winter	0.7437	0.9635	-0.2198	0.3675	6
140	End of winter:4 weeks of winter vs. SPC -4 weeks post winter:4 weeks of winter	0.7437	1.402	-0.6587	0.3675	6
141	End of winter:4 weeks of winter vs. SPC -4 weeks post winter:8 weeks of winter	0.7437	1.251	-0.5069	0.3675	6
142	End of winter:4 weeks of winter vs. SPC -8 weeks post winter:2 weeks of winter	0.7437	1.307	-0.5631	0.3675	6
143	End of winter:4 weeks of winter vs. SPC -8 weeks post winter:4 weeks of winter	0.7437	1.251	-0.5069	0.3675	6
144	End of winter:4 weeks of winter vs. SPC -8 weeks post winter:8 weeks of winter	0.7437	1.439	-0.6949	0.3675	6
145	End of winter:8 weeks of winter vs. 4 weeks post winter:2 weeks of winter	1.402	0.9066	0.4957	0.3675	6
146	End of winter:8 weeks of winter vs. 4 weeks post winter:4 weeks of winter	1.402	1.733	-0.3309	0.3675	6
147	End of winter:8 weeks of winter vs. 4 weeks post winter:8 weeks of winter	1.402	3.181	-1.778	0.3675	6
148	End of winter:8 weeks of winter vs. 8 weeks post winter:2 weeks of winter	1.402	0.8786	0.5237	0.3675	6
149	End of winter:8 weeks of winter vs. 8 weeks post winter:4 weeks of winter	1.402	1.955	-0.5522	0.3675	6
150	End of winter:8 weeks of winter vs. 8 weeks post winter:8 weeks of winter	1.402	2.645	-1.243	0.3675	6

2way ANOVA<br>Multiple comparisons						
151	End of winter:8 weeks of winter vs. SPC -4 weeks post winter:2 weeks of winter	1.402	0.9635	0.4389	0.3675	6
152	End of winter:8 weeks of winter vs. SPC -4 weeks post winter:4 weeks of winter	1.402	1.402	0	0.3675	6
153	End of winter:8 weeks of winter vs. SPC -4 weeks post winter:8 weeks of winter	1.402	1.251	0.1518	0.3675	6
154	End of winter:8 weeks of winter vs. SPC -8 weeks post winter:2 weeks of winter	1.402	1.307	0.09557	0.3675	6
155	End of winter:8 weeks of winter vs. SPC -8 weeks post winter:4 weeks of winter	1.402	1.251	0.1518	0.3675	6
156	End of winter:8 weeks of winter vs. SPC -8 weeks post winter:8 weeks of winter	1.402	1.439	-0.03626	0.3675	6
157	4 weeks post winter:2 weeks of winter vs. 4 weeks post winter:4 weeks of winter	0.9066	1.733	-0.8266	0.3675	6
158	4 weeks post winter:2 weeks of winter vs. 4 weeks post winter:8 weeks of winter	0.9066	3.181	-2.274	0.3675	6
159	4 weeks post winter:2 weeks of winter vs. 8 weeks post winter:2 weeks of winter	0.9066	0.8786	0.02801	0.3675	6
160	4 weeks post winter:2 weeks of winter vs. 8 weeks post winter:4 weeks of winter	0.9066	1.955	-1.048	0.3675	6
161	4 weeks post winter:2 weeks of winter vs. 8 weeks post winter:8 weeks of winter	0.9066	2.645	-1.739	0.3675	6
162	4 weeks post winter:2 weeks of winter vs. SPC -4 weeks post winter:2 weeks of winter	0.9066	0.9635	-0.05685	0.3675	6
163	4 weeks post winter:2 weeks of winter vs. SPC -4 weeks post winter:4 weeks of winter	0.9066	1.402	-0.4957	0.3675	6
164	4 weeks post winter:2 weeks of winter vs. SPC -4 weeks post winter:8 weeks of winter	0.9066	1.251	-0.3439	0.3675	6
165	4 weeks post winter:2 weeks of winter vs. SPC -8 weeks post winter:2 weeks of winter	0.9066	1.307	-0.4001	0.3675	6
166	4 weeks post winter:2 weeks of winter vs. SPC -8 weeks post winter:4 weeks of winter	0.9066	1.251	-0.3439	0.3675	6
167	4 weeks post winter:2 weeks of winter vs. SPC -8 weeks post winter:8 weeks of winter	0.9066	1.439	-0.532	0.3675	6
168	4 weeks post winter:4 weeks of winter vs. 4 weeks post winter:8 weeks of winter	1.733	3.181	-1.447	0.3675	6
169	4 weeks post winter:4 weeks of winter vs. 8 weeks post winter:2 weeks of winter	1.733	0.8786	0.8546	0.3675	6
170	4 weeks post winter:4 weeks of winter vs. 8 weeks post winter:4 weeks of winter	1.733	1.955	-0.2214	0.3675	6
171	4 weeks post winter:4 weeks of winter vs. 8 weeks post winter:8 weeks of winter	1.733	2.645	-0.9121	0.3675	6
172	4 weeks post winter:4 weeks of winter vs. SPC -4 weeks post winter:2 weeks of winter	1.733	0.9635	0.7697	0.3675	6
173	4 weeks post winter:4 weeks of winter vs. SPC -4 weeks post winter:4 weeks of winter	1.733	1.402	0.3309	0.3675	6
174	4 weeks post winter:4 weeks of winter vs. SPC -4 weeks post winter:8 weeks of winter	1.733	1.251	0.4827	0.3675	6
175	4 weeks post winter:4 weeks of winter vs. SPC -8 weeks post winter:2 weeks of winter	1.733	1.307	0.4264	0.3675	6
176	4 weeks post winter:4 weeks of winter vs. SPC -8 weeks post winter:4 weeks of winter	1.733	1.251	0.4827	0.3675	6
177	4 weeks post winter:4 weeks of winter vs. SPC -8 weeks post winter:8 weeks of winter	1.733	1.439	0.2946	0.3675	6
178	4 weeks post winter:8 weeks of winter vs. 8 weeks post winter:2 weeks of winter	3.181	0.8786	2.302	0.3675	6
179	4 weeks post winter:8 weeks of winter vs. 8 weeks post winter:4 weeks of winter	3.181	1.955	1.226	0.3675	6
180	4 weeks post winter:8 weeks of winter vs. 8 weeks post winter:8 weeks of winter	3.181	2.645	0.5352	0.3675	6

2way ANOVA<br>Multiple comparisons						
181	4 weeks post winter:8 weeks of winter vs. SPC -4 weeks post winter:2 weeks of winter	3.181	0.9635	2.217	0.3675	6
182	4 weeks post winter:8 weeks of winter vs. SPC -4 weeks post winter:4 weeks of winter	3.181	1.402	1.778	0.3675	6
183	4 weeks post winter:8 weeks of winter vs. SPC -4 weeks post winter:8 weeks of winter	3.181	1.251	1.93	0.3675	6
184	4 weeks post winter:8 weeks of winter vs. SPC -8 weeks post winter:2 weeks of winter	3.181	1.307	1.874	0.3675	6
185	4 weeks post winter:8 weeks of winter vs. SPC -8 weeks post winter:4 weeks of winter	3.181	1.251	1.93	0.3675	6
186	4 weeks post winter:8 weeks of winter vs. SPC -8 weeks post winter:8 weeks of winter	3.181	1.439	1.742	0.3675	6
187	8 weeks post winter:2 weeks of winter vs. 8 weeks post winter:4 weeks of winter	0.8786	1.955	-1.076	0.3675	6
188	8 weeks post winter:2 weeks of winter vs. 8 weeks post winter:8 weeks of winter	0.8786	2.645	-1.767	0.3675	6
189	8 weeks post winter:2 weeks of winter vs. SPC -4 weeks post winter:2 weeks of winter	0.8786	0.9635	-0.08486	0.3675	6
190	8 weeks post winter:2 weeks of winter vs. SPC -4 weeks post winter:4 weeks of winter	0.8786	1.402	-0.5237	0.3675	6
191	8 weeks post winter:2 weeks of winter vs. SPC -4 weeks post winter:8 weeks of winter	0.8786	1.251	-0.372	0.3675	6
192	8 weeks post winter:2 weeks of winter vs. SPC -8 weeks post winter:2 weeks of winter	0.8786	1.307	-0.4282	0.3675	6
193	8 weeks post winter:2 weeks of winter vs. SPC -8 weeks post winter:4 weeks of winter	0.8786	1.251	-0.372	0.3675	6
194	8 weeks post winter:2 weeks of winter vs. SPC -8 weeks post winter:8 weeks of winter	0.8786	1.439	-0.56	0.3675	6
195	8 weeks post winter:4 weeks of winter vs. 8 weeks post winter:8 weeks of winter	1.955	2.645	-0.6907	0.3675	6
196	8 weeks post winter:4 weeks of winter vs. SPC -4 weeks post winter:2 weeks of winter	1.955	0.9635	0.9911	0.3675	6
197	8 weeks post winter:4 weeks of winter vs. SPC -4 weeks post winter:4 weeks of winter	1.955	1.402	0.5522	0.3675	6
198	8 weeks post winter:4 weeks of winter vs. SPC -4 weeks post winter:8 weeks of winter	1.955	1.251	0.704	0.3675	6
199	8 weeks post winter:4 weeks of winter vs. SPC -8 weeks post winter:2 weeks of winter	1.955	1.307	0.6478	0.3675	6
200	8 weeks post winter:4 weeks of winter vs. SPC -8 weeks post winter:4 weeks of winter	1.955	1.251	0.704	0.3675	6
201	8 weeks post winter:4 weeks of winter vs. SPC -8 weeks post winter:8 weeks of winter	1.955	1.439	0.516	0.3675	6
202	8 weeks post winter:8 weeks of winter vs. SPC -4 weeks post winter:2 weeks of winter	2.645	0.9635	1.682	0.3675	6
203	8 weeks post winter:8 weeks of winter vs. SPC -4 weeks post winter:4 weeks of winter	2.645	1.402	1.243	0.3675	6
204	8 weeks post winter:8 weeks of winter vs. SPC -4 weeks post winter:8 weeks of winter	2.645	1.251	1.395	0.3675	6
205	8 weeks post winter:8 weeks of winter vs. SPC -8 weeks post winter:2 weeks of winter	2.645	1.307	1.339	0.3675	6
206	8 weeks post winter:8 weeks of winter vs. SPC -8 weeks post winter:4 weeks of winter	2.645	1.251	1.395	0.3675	6
207	8 weeks post winter:8 weeks of winter vs. SPC -8 weeks post winter:8 weeks of winter	2.645	1.439	1.207	0.3675	6
208	SPC -4 weeks post winter:2 weeks of winter vs. SPC -4 weeks post winter:4 weeks of winter	0.9635	1.402	-0.4389	0.3675	6
209	SPC -4 weeks post winter:2 weeks of winter vs. SPC -4 weeks post winter:8 weeks of winter	0.9635	1.251	-0.2871	0.3675	6
210	SPC -4 weeks post winter:2 weeks of winter vs. SPC -8 weeks post winter:2 weeks of winter	0.9635	1.307	-0.3433	0.3675	6

2way ANOVA
Multiple comparisons

211	SPC -4 weeks post winter:2 weeks of winter vs. SPC -8 weeks post winter:4 weeks of winter	0.9635	1.251	-0.2871	0.3675	6
212	SPC -4 weeks post winter:2 weeks of winter vs. SPC -8 weeks post winter:8 weeks of winter	0.9635	1.439	-0.4751	0.3675	6
213	SPC -4 weeks post winter:4 weeks of winter vs. SPC -4 weeks post winter:8 weeks of winter	1.402	1.251	0.1518	0.3675	6
214	SPC -4 weeks post winter:4 weeks of winter vs. SPC -8 weeks post winter:2 weeks of winter	1.402	1.307	0.09557	0.3675	6
215	SPC -4 weeks post winter:4 weeks of winter vs. SPC -8 weeks post winter:4 weeks of winter	1.402	1.251	0.1518	0.3675	6
216	SPC -4 weeks post winter:4 weeks of winter vs. SPC -8 weeks post winter:8 weeks of winter	1.402	1.439	-0.03626	0.3675	6
217	SPC -4 weeks post winter:8 weeks of winter vs. SPC -8 weeks post winter:2 weeks of winter	1.251	1.307	-0.05621	0.3675	6
218	SPC -4 weeks post winter:8 weeks of winter vs. SPC -8 weeks post winter:4 weeks of winter	1.251	1.251	0	0.3675	6
219	SPC -4 weeks post winter:8 weeks of winter vs. SPC -8 weeks post winter:8 weeks of winter	1.251	1.439	-0.188	0.3675	6
220	SPC -8 weeks post winter:2 weeks of winter vs. SPC -8 weeks post winter:4 weeks of winter	1.307	1.251	0.05621	0.3675	6
221	SPC -8 weeks post winter:2 weeks of winter vs. SPC -8 weeks post winter:8 weeks of winter	1.307	1.439	-0.1318	0.3675	6
222	SPC -8 weeks post winter:4 weeks of winter vs. SPC -8 weeks post winter:8 weeks of winter	1.251	1.439	-0.188	0.3675	6

1			
2			
3			
4			
5			
6			
7			
8			
9			
10			
11			
12			
13			
14			
15			
16			
17			
18			
19			
20			
21			
22			
23			
24			
25			
26			
27			
28			
29			
30			

31			
32			
33			
34			
35			
36			
37			
38			
39			
40			
41			
42			
43			
44			
45			
46			
47			
48			
49			
50			
51			
52			
53			
54			
55			
56			
57			
58			
59			
60			

61			
62			
63			
64			
65			
66			
67			
68			
69			
70			
71			
72			
73			
74			
75			
76			
77			
78			
79			
80			
81			
82			
83			
84			
85			
86			
87			
88			
89			
90			

91			
92			
93			
94			
95			
96			
97			
98			
99			
100			
101			
102			
103			
104			
105			
106			
107			
108			
109			
110			
111			
112			
113			
114			
115			
116	N2	q	DF
117			
118	6	1.143	75
119	6	1.392	75
120	6	0.5155	75

**121**	6	2.666	75
**122**	6	8.235	75
**123**	6	0.6233	75
**124**	6	3.518	75
**125**	6	6.176	75
**126**	6	0.2967	75
**127**	6	1.392	75
**128**	6	0.8081	75
**129**	6	1.024	75
**130**	6	0.8081	75
**131**	6	1.532	75
**132**	6	2.535	75
**133**	6	0.6271	75
**134**	6	3.808	75
**135**	6	9.378	75
**136**	6	0.5194	75
**137**	6	4.66	75
**138**	6	7.318	75
**139**	6	0.8459	75
**140**	6	2.535	75
**141**	6	1.951	75
**142**	6	2.167	75
**143**	6	1.951	75
**144**	6	2.674	75
**145**	6	1.908	75
**146**	6	1.273	75
**147**	6	6.843	75
**148**	6	2.016	75
**149**	6	2.125	75
**150**	6	4.783	75

**151**	6	1.689	75
**152**	6	0	75
**153**	6	0.5841	75
**154**	6	0.3678	75
**155**	6	0.5841	75
**156**	6	0.1395	75
**157**	6	3.181	75
**158**	6	8.751	75
**159**	6	0.1078	75
**160**	6	4.033	75
**161**	6	6.691	75
**162**	6	0.2188	75
**163**	6	1.908	75
**164**	6	1.324	75
**165**	6	1.54	75
**166**	6	1.324	75
**167**	6	2.047	75
**168**	6	5.57	75
**169**	6	3.289	75
**170**	6	0.8519	75
**171**	6	3.51	75
**172**	6	2.962	75
**173**	6	1.273	75
**174**	6	1.857	75
**175**	6	1.641	75
**176**	6	1.857	75
**177**	6	1.134	75
**178**	6	8.859	75
**179**	6	4.718	75
**180**	6	2.06	75

**181**	6	8.532	75
**182**	6	6.843	75
**183**	6	7.427	75
**184**	6	7.211	75
**185**	6	7.427	75
**186**	6	6.703	75
**187**	6	4.141	75
**188**	6	6.799	75
**189**	6	0.3266	75
**190**	6	2.016	75
**191**	6	1.431	75
**192**	6	1.648	75
**193**	6	1.431	75
**194**	6	2.155	75
**195**	6	2.658	75
**196**	6	3.814	75
**197**	6	2.125	75
**198**	6	2.709	75
**199**	6	2.493	75
**200**	6	2.709	75
**201**	6	1.986	75
**202**	6	6.472	75
**203**	6	4.783	75
**204**	6	5.367	75
**205**	6	5.151	75
**206**	6	5.367	75
**207**	6	4.644	75
**208**	6	1.689	75
**209**	6	1.105	75
**210**	6	1.321	75

**211**	6	1.105	75
**212**	6	1.828	75
**213**	6	0.5841	75
**214**	6	0.3678	75
**215**	6	0.5841	75
**216**	6	0.1395	75
**217**	6	0.2163	75
**218**	6	0	75
**219**	6	0.7237	75
**220**	6	0.2163	75
**221**	6	0.5073	75
**222**	6	0.7237	75

2way ANOVA
Tabular results

1	Table Analyzed	S100A				
2						
3	Two-way ANOVA	Ordinary				
4	Alpha	0.05				
5						
6	Source of Variation	% of total variation	P value	P value summary	Significant?	
7	Interaction	16.69	<0.0001	****	Yes	
8	Time post-winter	44.31	<0.0001	****	Yes	
9	Treatment	16.22	<0.0001	****	Yes	
10						
11	ANOVA table	SS	DF	MS	F (DFn, DFd)	P value
12	Interaction	139.8	8	17.47	F (8, 75) = 6.864	P<0.0001
13	Time post-winter	371.1	4	92.77	F (4, 75) = 36.45	P<0.0001
14	Treatment	135.8	2	67.9	F (2, 75) = 26.68	P<0.0001
15	Residual	190.9	75	2.545		
16						
17	Number of missing values	0				

2way ANOVA
Multiple comparisons

1	Compare cell means regardless of rows and columns					
2						
3	Number of families	1				
4	Number of comparisons per family	105				
5	Alpha	0.05				
6						
7	Tukey's multiple comparisons test	Mean Diff.	95.00% CI of diff.	Significant?	Summary	Adjusted P Value
8						
9	End of winter:2 weeks of winter vs. End of winter:4 weeks of winter	-0.2441	-3.474 to 2.986	No	ns	>0.9999
10	End of winter:2 weeks of winter vs. End of winter:8 weeks of winter	-0.04865	-3.279 to 3.182	No	ns	>0.9999
11	End of winter:2 weeks of winter vs. 4 weeks post winter:2 weeks of winter	-0.7398	-3.97 to 2.49	No	ns	>0.9999
12	End of winter:2 weeks of winter vs. 4 weeks post winter:4 weeks of winter	-1.701	-4.931 to 1.529	No	ns	0.8708
13	End of winter:2 weeks of winter vs. 4 weeks post winter:8 weeks of winter	-6.062	-9.292 to -2.832	Yes	****	<0.0001
14	End of winter:2 weeks of winter vs. 8 weeks post winter:2 weeks of winter	-1.674	-4.904 to 1.556	No	ns	0.8835
15	End of winter:2 weeks of winter vs. 8 weeks post winter:4 weeks of winter	-4.879	-8.109 to -1.649	Yes	***	0.0001
16	End of winter:2 weeks of winter vs. 8 weeks post winter:8 weeks of winter	-9.18	-12.41 to -5.95	Yes	****	<0.0001
17	End of winter:2 weeks of winter vs. SPC -4 weeks post winter:2 weeks of winter	0.1999	-3.03 to 3.43	No	ns	>0.9999
18	End of winter:2 weeks of winter vs. SPC -4 weeks post winter:4 weeks of winter	-0.04865	-3.279 to 3.182	No	ns	>0.9999
19	End of winter:2 weeks of winter vs. SPC -4 weeks post winter:8 weeks of winter	-0.1656	-3.396 to 3.065	No	ns	>0.9999
20	End of winter:2 weeks of winter vs. SPC -8 weeks post winter:2 weeks of winter	0.02359	-3.207 to 3.254	No	ns	>0.9999
21	End of winter:2 weeks of winter vs. SPC -8 weeks post winter:4 weeks of winter	-0.1656	-3.396 to 3.065	No	ns	>0.9999
22	End of winter:2 weeks of winter vs. SPC -8 weeks post winter:8 weeks of winter	-1.492	-4.723 to 1.738	No	ns	0.9487
23	End of winter:4 weeks of winter vs. End of winter:8 weeks of winter	0.1954	-3.035 to 3.426	No	ns	>0.9999
24	End of winter:4 weeks of winter vs. 4 weeks post winter:2 weeks of winter	-0.4957	-3.726 to 2.734	No	ns	>0.9999
25	End of winter:4 weeks of winter vs. 4 weeks post winter:4 weeks of winter	-1.457	-4.687 to 1.774	No	ns	0.9576
26	End of winter:4 weeks of winter vs. 4 weeks post winter:8 weeks of winter	-5.818	-9.048 to -2.587	Yes	****	<0.0001
27	End of winter:4 weeks of winter vs. 8 weeks post winter:2 weeks of winter	-1.43	-4.66 to 1.8	No	ns	0.9634
28	End of winter:4 weeks of winter vs. 8 weeks post winter:4 weeks of winter	-4.635	-7.865 to -1.405	Yes	***	0.0003
29	End of winter:4 weeks of winter vs. 8 weeks post winter:8 weeks of winter	-8.936	-12.17 to -5.706	Yes	****	<0.0001
30	End of winter:4 weeks of winter vs. SPC -4 weeks post winter:2 weeks of winter	0.444	-2.786 to 3.674	No	ns	>0.9999

2way ANOVA<br>Multiple comparisons						
31	End of winter:4 weeks of winter vs. SPC -4 weeks post winter:4 weeks of winter	0.1954	-3.035 to 3.426	No	ns	>0.9999
32	End of winter:4 weeks of winter vs. SPC -4 weeks post winter:8 weeks of winter	0.07851	-3.152 to 3.309	No	ns	>0.9999
33	End of winter:4 weeks of winter vs. SPC -8 weeks post winter:2 weeks of winter	0.2677	-2.962 to 3.498	No	ns	>0.9999
34	End of winter:4 weeks of winter vs. SPC -8 weeks post winter:4 weeks of winter	0.07851	-3.152 to 3.309	No	ns	>0.9999
35	End of winter:4 weeks of winter vs. SPC -8 weeks post winter:8 weeks of winter	-1.248	-4.478 to 1.982	No	ns	0.9888
36	End of winter:8 weeks of winter vs. 4 weeks post winter:2 weeks of winter	-0.6912	-3.921 to 2.539	No	ns	>0.9999
37	End of winter:8 weeks of winter vs. 4 weeks post winter:4 weeks of winter	-1.652	-4.882 to 1.578	No	ns	0.8932
38	End of winter:8 weeks of winter vs. 4 weeks post winter:8 weeks of winter	-6.013	-9.243 to -2.783	Yes	****	<0.0001
39	End of winter:8 weeks of winter vs. 8 weeks post winter:2 weeks of winter	-1.625	-4.855 to 1.605	No	ns	0.9044
40	End of winter:8 weeks of winter vs. 8 weeks post winter:4 weeks of winter	-4.831	-8.061 to -1.6	Yes	***	0.0001
41	End of winter:8 weeks of winter vs. 8 weeks post winter:8 weeks of winter	-9.131	-12.36 to -5.901	Yes	****	<0.0001
42	End of winter:8 weeks of winter vs. SPC -4 weeks post winter:2 weeks of winter	0.2486	-2.982 to 3.479	No	ns	>0.9999
43	End of winter:8 weeks of winter vs. SPC -4 weeks post winter:4 weeks of winter	0	-3.23 to 3.23	No	ns	>0.9999
44	End of winter:8 weeks of winter vs. SPC -4 weeks post winter:8 weeks of winter	-0.1169	-3.347 to 3.113	No	ns	>0.9999
45	End of winter:8 weeks of winter vs. SPC -8 weeks post winter:2 weeks of winter	0.07224	-3.158 to 3.302	No	ns	>0.9999
46	End of winter:8 weeks of winter vs. SPC -8 weeks post winter:4 weeks of winter	-0.1169	-3.347 to 3.113	No	ns	>0.9999
47	End of winter:8 weeks of winter vs. SPC -8 weeks post winter:8 weeks of winter	-1.444	-4.674 to 1.786	No	ns	0.9605
48	4 weeks post winter:2 weeks of winter vs. 4 weeks post winter:4 weeks of winter	-0.9609	-4.191 to 2.269	No	ns	0.9992
49	4 weeks post winter:2 weeks of winter vs. 4 weeks post winter:8 weeks of winter	-5.322	-8.552 to -2.092	Yes	****	<0.0001
50	4 weeks post winter:2 weeks of winter vs. 8 weeks post winter:2 weeks of winter	-0.934	-4.164 to 2.296	No	ns	0.9994
51	4 weeks post winter:2 weeks of winter vs. 8 weeks post winter:4 weeks of winter	-4.139	-7.37 to -0.9093	Yes	**	0.0022
52	4 weeks post winter:2 weeks of winter vs. 8 weeks post winter:8 weeks of winter	-8.44	-11.67 to -5.21	Yes	****	<0.0001
53	4 weeks post winter:2 weeks of winter vs. SPC -4 weeks post winter:2 weeks of winter	0.9397	-2.29 to 4.17	No	ns	0.9994
54	4 weeks post winter:2 weeks of winter vs. SPC -4 weeks post winter:4 weeks of winter	0.6912	-2.539 to 3.921	No	ns	>0.9999
55	4 weeks post winter:2 weeks of winter vs. SPC -4 weeks post winter:8 weeks of winter	0.5742	-2.656 to 3.804	No	ns	>0.9999
56	4 weeks post winter:2 weeks of winter vs. SPC -8 weeks post winter:2 weeks of winter	0.7634	-2.467 to 3.994	No	ns	>0.9999
57	4 weeks post winter:2 weeks of winter vs. SPC -8 weeks post winter:4 weeks of winter	0.5742	-2.656 to 3.804	No	ns	>0.9999
58	4 weeks post winter:2 weeks of winter vs. SPC -8 weeks post winter:8 weeks of winter	-0.7526	-3.983 to 2.478	No	ns	>0.9999
59	4 weeks post winter:4 weeks of winter vs. 4 weeks post winter:8 weeks of winter	-4.361	-7.591 to -1.131	Yes	***	0.0009
60	4 weeks post winter:4 weeks of winter vs. 8 weeks post winter:2 weeks of winter	0.02688	-3.203 to 3.257	No	ns	>0.9999

2way ANOVA<br>Multiple comparisons						
61	4 weeks post winter:4 weeks of winter vs. 8 weeks post winter:4 weeks of winter	-3.179	-6.409 to 0.05163	No	ns	0.0583
62	4 weeks post winter:4 weeks of winter vs. 8 weeks post winter:8 weeks of winter	-7.479	-10.71 to -4.249	Yes	****	<0.0001
63	4 weeks post winter:4 weeks of winter vs. SPC -4 weeks post winter:2 weeks of winter	1.901	-1.33 to 5.131	No	ns	0.7536
64	4 weeks post winter:4 weeks of winter vs. SPC -4 weeks post winter:4 weeks of winter	1.652	-1.578 to 4.882	No	ns	0.8932
65	4 weeks post winter:4 weeks of winter vs. SPC -4 weeks post winter:8 weeks of winter	1.535	-1.695 to 4.765	No	ns	0.9365
66	4 weeks post winter:4 weeks of winter vs. SPC -8 weeks post winter:2 weeks of winter	1.724	-1.506 to 4.954	No	ns	0.8591
67	4 weeks post winter:4 weeks of winter vs. SPC -8 weeks post winter:4 weeks of winter	1.535	-1.695 to 4.765	No	ns	0.9365
68	4 weeks post winter:4 weeks of winter vs. SPC -8 weeks post winter:8 weeks of winter	0.2083	-3.022 to 3.438	No	ns	>0.9999
69	4 weeks post winter:8 weeks of winter vs. 8 weeks post winter:2 weeks of winter	4.388	1.158 to 7.618	Yes	***	0.0008
70	4 weeks post winter:8 weeks of winter vs. 8 weeks post winter:4 weeks of winter	1.182	-2.048 to 4.413	No	ns	0.9933
71	4 weeks post winter:8 weeks of winter vs. 8 weeks post winter:8 weeks of winter	-3.118	-6.348 to 0.1119	No	ns	0.0695
72	4 weeks post winter:8 weeks of winter vs. SPC -4 weeks post winter:2 weeks of winter	6.262	3.031 to 9.492	Yes	****	<0.0001
73	4 weeks post winter:8 weeks of winter vs. SPC -4 weeks post winter:4 weeks of winter	6.013	2.783 to 9.243	Yes	****	<0.0001
74	4 weeks post winter:8 weeks of winter vs. SPC -4 weeks post winter:8 weeks of winter	5.896	2.666 to 9.126	Yes	****	<0.0001
75	4 weeks post winter:8 weeks of winter vs. SPC -8 weeks post winter:2 weeks of winter	6.085	2.855 to 9.315	Yes	****	<0.0001
76	4 weeks post winter:8 weeks of winter vs. SPC -8 weeks post winter:4 weeks of winter	5.896	2.666 to 9.126	Yes	****	<0.0001
77	4 weeks post winter:8 weeks of winter vs. SPC -8 weeks post winter:8 weeks of winter	4.569	1.339 to 7.799	Yes	***	0.0004
78	8 weeks post winter:2 weeks of winter vs. 8 weeks post winter:4 weeks of winter	-3.205	-6.436 to 0.02475	No	ns	0.0539
79	8 weeks post winter:2 weeks of winter vs. 8 weeks post winter:8 weeks of winter	-7.506	-10.74 to -4.276	Yes	****	<0.0001
80	8 weeks post winter:2 weeks of winter vs. SPC -4 weeks post winter:2 weeks of winter	1.874	-1.356 to 5.104	No	ns	0.7715
81	8 weeks post winter:2 weeks of winter vs. SPC -4 weeks post winter:4 weeks of winter	1.625	-1.605 to 4.855	No	ns	0.9044
82	8 weeks post winter:2 weeks of winter vs. SPC -4 weeks post winter:8 weeks of winter	1.508	-1.722 to 4.738	No	ns	0.9444
83	8 weeks post winter:2 weeks of winter vs. SPC -8 weeks post winter:2 weeks of winter	1.697	-1.533 to 4.928	No	ns	0.8724
84	8 weeks post winter:2 weeks of winter vs. SPC -8 weeks post winter:4 weeks of winter	1.508	-1.722 to 4.738	No	ns	0.9444
85	8 weeks post winter:2 weeks of winter vs. SPC -8 weeks post winter:8 weeks of winter	0.1814	-3.049 to 3.412	No	ns	>0.9999
86	8 weeks post winter:4 weeks of winter vs. 8 weeks post winter:8 weeks of winter	-4.301	-7.531 to -1.071	Yes	**	0.0012
87	8 weeks post winter:4 weeks of winter vs. SPC -4 weeks post winter:2 weeks of winter	5.079	1.849 to 8.309	Yes	****	<0.0001
88	8 weeks post winter:4 weeks of winter vs. SPC -4 weeks post winter:4 weeks of winter	4.831	1.6 to 8.061	Yes	***	0.0001
89	8 weeks post winter:4 weeks of winter vs. SPC -4 weeks post winter:8 weeks of winter	4.714	1.483 to 7.944	Yes	***	0.0002
90	8 weeks post winter:4 weeks of winter vs. SPC -8 weeks post winter:2 weeks of winter	4.903	1.673 to 8.133	Yes	****	<0.0001

2way ANOVA<br>Multiple comparisons						
91	8 weeks post winter:4 weeks of winter vs. SPC -8 weeks post winter:4 weeks of winter	4.714	1.483 to 7.944	Yes	***	0.0002
92	8 weeks post winter:4 weeks of winter vs. SPC -8 weeks post winter:8 weeks of winter	3.387	0.1567 to 6.617	Yes	*	0.0308
93	8 weeks post winter:8 weeks of winter vs. SPC -4 weeks post winter:2 weeks of winter	9.38	6.15 to 12.61	Yes	****	<0.0001
94	8 weeks post winter:8 weeks of winter vs. SPC -4 weeks post winter:4 weeks of winter	9.131	5.901 to 12.36	Yes	****	<0.0001
95	8 weeks post winter:8 weeks of winter vs. SPC -4 weeks post winter:8 weeks of winter	9.014	5.784 to 12.24	Yes	****	<0.0001
96	8 weeks post winter:8 weeks of winter vs. SPC -8 weeks post winter:2 weeks of winter	9.204	5.973 to 12.43	Yes	****	<0.0001
97	8 weeks post winter:8 weeks of winter vs. SPC -8 weeks post winter:4 weeks of winter	9.014	5.784 to 12.24	Yes	****	<0.0001
98	8 weeks post winter:8 weeks of winter vs. SPC -8 weeks post winter:8 weeks of winter	7.688	4.457 to 10.92	Yes	****	<0.0001
99	SPC -4 weeks post winter:2 weeks of winter vs. SPC -4 weeks post winter:4 weeks of winter	-0.2486	-3.479 to 2.982	No	ns	>0.9999
100	SPC -4 weeks post winter:2 weeks of winter vs. SPC -4 weeks post winter:8 weeks of winter	-0.3655	-3.596 to 2.865	No	ns	>0.9999
101	SPC -4 weeks post winter:2 weeks of winter vs. SPC -8 weeks post winter:2 weeks of winter	-0.1763	-3.407 to 3.054	No	ns	>0.9999
102	SPC -4 weeks post winter:2 weeks of winter vs. SPC -8 weeks post winter:4 weeks of winter	-0.3655	-3.596 to 2.865	No	ns	>0.9999
103	SPC -4 weeks post winter:2 weeks of winter vs. SPC -8 weeks post winter:8 weeks of winter	-1.692	-4.923 to 1.538	No	ns	0.8749
104	SPC -4 weeks post winter:4 weeks of winter vs. SPC -4 weeks post winter:8 weeks of winter	-0.1169	-3.347 to 3.113	No	ns	>0.9999
105	SPC -4 weeks post winter:4 weeks of winter vs. SPC -8 weeks post winter:2 weeks of winter	0.07224	-3.158 to 3.302	No	ns	>0.9999
106	SPC -4 weeks post winter:4 weeks of winter vs. SPC -8 weeks post winter:4 weeks of winter	-0.1169	-3.347 to 3.113	No	ns	>0.9999
107	SPC -4 weeks post winter:4 weeks of winter vs. SPC -8 weeks post winter:8 weeks of winter	-1.444	-4.674 to 1.786	No	ns	0.9605
108	SPC -4 weeks post winter:8 weeks of winter vs. SPC -8 weeks post winter:2 weeks of winter	0.1892	-3.041 to 3.419	No	ns	>0.9999
109	SPC -4 weeks post winter:8 weeks of winter vs. SPC -8 weeks post winter:4 weeks of winter	0	-3.23 to 3.23	No	ns	>0.9999
110	SPC -4 weeks post winter:8 weeks of winter vs. SPC -8 weeks post winter:8 weeks of winter	-1.327	-4.557 to 1.903	No	ns	0.9805
111	SPC -8 weeks post winter:2 weeks of winter vs. SPC -8 weeks post winter:4 weeks of winter	-0.1892	-3.419 to 3.041	No	ns	>0.9999
112	SPC -8 weeks post winter:2 weeks of winter vs. SPC -8 weeks post winter:8 weeks of winter	-1.516	-4.746 to 1.714	No	ns	0.9422
113	SPC -8 weeks post winter:4 weeks of winter vs. SPC -8 weeks post winter:8 weeks of winter	-1.327	-4.557 to 1.903	No	ns	0.9805
114						
115						
116	Test details	Mean 1	Mean 2	Mean Diff.	SE of diff.	N1
117						
118	End of winter:2 weeks of winter vs. End of winter:4 weeks of winter	0.6577	0.9018	-0.2441	0.9211	6
119	End of winter:2 weeks of winter vs. End of winter:8 weeks of winter	0.6577	0.7064	-0.04865	0.9211	6
120	End of winter:2 weeks of winter vs. 4 weeks post winter:2 weeks of winter	0.6577	1.398	-0.7398	0.9211	6

2way ANOVA<br>Multiple comparisons						
121	End of winter:2 weeks of winter vs. 4 weeks post winter:4 weeks of winter	0.6577	2.358	-1.701	0.9211	6
122	End of winter:2 weeks of winter vs. 4 weeks post winter:8 weeks of winter	0.6577	6.719	-6.062	0.9211	6
123	End of winter:2 weeks of winter vs. 8 weeks post winter:2 weeks of winter	0.6577	2.332	-1.674	0.9211	6
124	End of winter:2 weeks of winter vs. 8 weeks post winter:4 weeks of winter	0.6577	5.537	-4.879	0.9211	6
125	End of winter:2 weeks of winter vs. 8 weeks post winter:8 weeks of winter	0.6577	9.838	-9.18	0.9211	6
126	End of winter:2 weeks of winter vs. SPC -4 weeks post winter:2 weeks of winter	0.6577	0.4578	0.1999	0.9211	6
127	End of winter:2 weeks of winter vs. SPC -4 weeks post winter:4 weeks of winter	0.6577	0.7064	-0.04865	0.9211	6
128	End of winter:2 weeks of winter vs. SPC -4 weeks post winter:8 weeks of winter	0.6577	0.8233	-0.1656	0.9211	6
129	End of winter:2 weeks of winter vs. SPC -8 weeks post winter:2 weeks of winter	0.6577	0.6341	0.02359	0.9211	6
130	End of winter:2 weeks of winter vs. SPC -8 weeks post winter:4 weeks of winter	0.6577	0.8233	-0.1656	0.9211	6
131	End of winter:2 weeks of winter vs. SPC -8 weeks post winter:8 weeks of winter	0.6577	2.15	-1.492	0.9211	6
132	End of winter:4 weeks of winter vs. End of winter:8 weeks of winter	0.9018	0.7064	0.1954	0.9211	6
133	End of winter:4 weeks of winter vs. 4 weeks post winter:2 weeks of winter	0.9018	1.398	-0.4957	0.9211	6
134	End of winter:4 weeks of winter vs. 4 weeks post winter:4 weeks of winter	0.9018	2.358	-1.457	0.9211	6
135	End of winter:4 weeks of winter vs. 4 weeks post winter:8 weeks of winter	0.9018	6.719	-5.818	0.9211	6
136	End of winter:4 weeks of winter vs. 8 weeks post winter:2 weeks of winter	0.9018	2.332	-1.43	0.9211	6
137	End of winter:4 weeks of winter vs. 8 weeks post winter:4 weeks of winter	0.9018	5.537	-4.635	0.9211	6
138	End of winter:4 weeks of winter vs. 8 weeks post winter:8 weeks of winter	0.9018	9.838	-8.936	0.9211	6
139	End of winter:4 weeks of winter vs. SPC -4 weeks post winter:2 weeks of winter	0.9018	0.4578	0.444	0.9211	6
140	End of winter:4 weeks of winter vs. SPC -4 weeks post winter:4 weeks of winter	0.9018	0.7064	0.1954	0.9211	6
141	End of winter:4 weeks of winter vs. SPC -4 weeks post winter:8 weeks of winter	0.9018	0.8233	0.07851	0.9211	6
142	End of winter:4 weeks of winter vs. SPC -8 weeks post winter:2 weeks of winter	0.9018	0.6341	0.2677	0.9211	6
143	End of winter:4 weeks of winter vs. SPC -8 weeks post winter:4 weeks of winter	0.9018	0.8233	0.07851	0.9211	6
144	End of winter:4 weeks of winter vs. SPC -8 weeks post winter:8 weeks of winter	0.9018	2.15	-1.248	0.9211	6
145	End of winter:8 weeks of winter vs. 4 weeks post winter:2 weeks of winter	0.7064	1.398	-0.6912	0.9211	6
146	End of winter:8 weeks of winter vs. 4 weeks post winter:4 weeks of winter	0.7064	2.358	-1.652	0.9211	6
147	End of winter:8 weeks of winter vs. 4 weeks post winter:8 weeks of winter	0.7064	6.719	-6.013	0.9211	6
148	End of winter:8 weeks of winter vs. 8 weeks post winter:2 weeks of winter	0.7064	2.332	-1.625	0.9211	6
149	End of winter:8 weeks of winter vs. 8 weeks post winter:4 weeks of winter	0.7064	5.537	-4.831	0.9211	6
150	End of winter:8 weeks of winter vs. 8 weeks post winter:8 weeks of winter	0.7064	9.838	-9.131	0.9211	6

**2way ANOVA** Multiple comparisons						
151	End of winter:8 weeks of winter vs. SPC -4 weeks post winter:2 weeks of winter	0.7064	0.4578	0.2486	0.9211	6
152	End of winter:8 weeks of winter vs. SPC -4 weeks post winter:4 weeks of winter	0.7064	0.7064	0	0.9211	6
153	End of winter:8 weeks of winter vs. SPC -4 weeks post winter:8 weeks of winter	0.7064	0.8233	-0.1169	0.9211	6
154	End of winter:8 weeks of winter vs. SPC -8 weeks post winter:2 weeks of winter	0.7064	0.6341	0.07224	0.9211	6
155	End of winter:8 weeks of winter vs. SPC -8 weeks post winter:4 weeks of winter	0.7064	0.8233	-0.1169	0.9211	6
156	End of winter:8 weeks of winter vs. SPC -8 weeks post winter:8 weeks of winter	0.7064	2.15	-1.444	0.9211	6
157	4 weeks post winter:2 weeks of winter vs. 4 weeks post winter:4 weeks of winter	1.398	2.358	-0.9609	0.9211	6
158	4 weeks post winter:2 weeks of winter vs. 4 weeks post winter:8 weeks of winter	1.398	6.719	-5.322	0.9211	6
159	4 weeks post winter:2 weeks of winter vs. 8 weeks post winter:2 weeks of winter	1.398	2.332	-0.934	0.9211	6
160	4 weeks post winter:2 weeks of winter vs. 8 weeks post winter:4 weeks of winter	1.398	5.537	-4.139	0.9211	6
161	4 weeks post winter:2 weeks of winter vs. 8 weeks post winter:8 weeks of winter	1.398	9.838	-8.44	0.9211	6
162	4 weeks post winter:2 weeks of winter vs. SPC -4 weeks post winter:2 weeks of winter	1.398	0.4578	0.9397	0.9211	6
163	4 weeks post winter:2 weeks of winter vs. SPC -4 weeks post winter:4 weeks of winter	1.398	0.7064	0.6912	0.9211	6
164	4 weeks post winter:2 weeks of winter vs. SPC -4 weeks post winter:8 weeks of winter	1.398	0.8233	0.5742	0.9211	6
165	4 weeks post winter:2 weeks of winter vs. SPC -8 weeks post winter:2 weeks of winter	1.398	0.6341	0.7634	0.9211	6
166	4 weeks post winter:2 weeks of winter vs. SPC -8 weeks post winter:4 weeks of winter	1.398	0.8233	0.5742	0.9211	6
167	4 weeks post winter:2 weeks of winter vs. SPC -8 weeks post winter:8 weeks of winter	1.398	2.15	-0.7526	0.9211	6
168	4 weeks post winter:4 weeks of winter vs. 4 weeks post winter:8 weeks of winter	2.358	6.719	-4.361	0.9211	6
169	4 weeks post winter:4 weeks of winter vs. 8 weeks post winter:2 weeks of winter	2.358	2.332	0.02688	0.9211	6
170	4 weeks post winter:4 weeks of winter vs. 8 weeks post winter:4 weeks of winter	2.358	5.537	-3.179	0.9211	6
171	4 weeks post winter:4 weeks of winter vs. 8 weeks post winter:8 weeks of winter	2.358	9.838	-7.479	0.9211	6
172	4 weeks post winter:4 weeks of winter vs. SPC -4 weeks post winter:2 weeks of winter	2.358	0.4578	1.901	0.9211	6
173	4 weeks post winter:4 weeks of winter vs. SPC -4 weeks post winter:4 weeks of winter	2.358	0.7064	1.652	0.9211	6
174	4 weeks post winter:4 weeks of winter vs. SPC -4 weeks post winter:8 weeks of winter	2.358	0.8233	1.535	0.9211	6
175	4 weeks post winter:4 weeks of winter vs. SPC -8 weeks post winter:2 weeks of winter	2.358	0.6341	1.724	0.9211	6
176	4 weeks post winter:4 weeks of winter vs. SPC -8 weeks post winter:4 weeks of winter	2.358	0.8233	1.535	0.9211	6
177	4 weeks post winter:4 weeks of winter vs. SPC -8 weeks post winter:8 weeks of winter	2.358	2.15	0.2083	0.9211	6
178	4 weeks post winter:8 weeks of winter vs. 8 weeks post winter:2 weeks of winter	6.719	2.332	4.388	0.9211	6
179	4 weeks post winter:8 weeks of winter vs. 8 weeks post winter:4 weeks of winter	6.719	5.537	1.182	0.9211	6
180	4 weeks post winter:8 weeks of winter vs. 8 weeks post winter:8 weeks of winter	6.719	9.838	-3.118	0.9211	6

**2way ANOVA**<br>Multiple comparisons						
**181**	4 weeks post winter:8 weeks of winter vs. SPC -4 weeks post winter:2 weeks of winter	6.719	0.4578	6.262	0.9211	6
**182**	4 weeks post winter:8 weeks of winter vs. SPC -4 weeks post winter:4 weeks of winter	6.719	0.7064	6.013	0.9211	6
**183**	4 weeks post winter:8 weeks of winter vs. SPC -4 weeks post winter:8 weeks of winter	6.719	0.8233	5.896	0.9211	6
**184**	4 weeks post winter:8 weeks of winter vs. SPC -8 weeks post winter:2 weeks of winter	6.719	0.6341	6.085	0.9211	6
**185**	4 weeks post winter:8 weeks of winter vs. SPC -8 weeks post winter:4 weeks of winter	6.719	0.8233	5.896	0.9211	6
**186**	4 weeks post winter:8 weeks of winter vs. SPC -8 weeks post winter:8 weeks of winter	6.719	2.15	4.569	0.9211	6
**187**	8 weeks post winter:2 weeks of winter vs. 8 weeks post winter:4 weeks of winter	2.332	5.537	-3.205	0.9211	6
**188**	8 weeks post winter:2 weeks of winter vs. 8 weeks post winter:8 weeks of winter	2.332	9.838	-7.506	0.9211	6
**189**	8 weeks post winter:2 weeks of winter vs. SPC -4 weeks post winter:2 weeks of winter	2.332	0.4578	1.874	0.9211	6
**190**	8 weeks post winter:2 weeks of winter vs. SPC -4 weeks post winter:4 weeks of winter	2.332	0.7064	1.625	0.9211	6
**191**	8 weeks post winter:2 weeks of winter vs. SPC -4 weeks post winter:8 weeks of winter	2.332	0.8233	1.508	0.9211	6
**192**	8 weeks post winter:2 weeks of winter vs. SPC -8 weeks post winter:2 weeks of winter	2.332	0.6341	1.697	0.9211	6
**193**	8 weeks post winter:2 weeks of winter vs. SPC -8 weeks post winter:4 weeks of winter	2.332	0.8233	1.508	0.9211	6
**194**	8 weeks post winter:2 weeks of winter vs. SPC -8 weeks post winter:8 weeks of winter	2.332	2.15	0.1814	0.9211	6
**195**	8 weeks post winter:4 weeks of winter vs. 8 weeks post winter:8 weeks of winter	5.537	9.838	-4.301	0.9211	6
**196**	8 weeks post winter:4 weeks of winter vs. SPC -4 weeks post winter:2 weeks of winter	5.537	0.4578	5.079	0.9211	6
**197**	8 weeks post winter:4 weeks of winter vs. SPC -4 weeks post winter:4 weeks of winter	5.537	0.7064	4.831	0.9211	6
**198**	8 weeks post winter:4 weeks of winter vs. SPC -4 weeks post winter:8 weeks of winter	5.537	0.8233	4.714	0.9211	6
**199**	8 weeks post winter:4 weeks of winter vs. SPC -8 weeks post winter:2 weeks of winter	5.537	0.6341	4.903	0.9211	6
**200**	8 weeks post winter:4 weeks of winter vs. SPC -8 weeks post winter:4 weeks of winter	5.537	0.8233	4.714	0.9211	6
**201**	8 weeks post winter:4 weeks of winter vs. SPC -8 weeks post winter:8 weeks of winter	5.537	2.15	3.387	0.9211	6
**202**	8 weeks post winter:8 weeks of winter vs. SPC -4 weeks post winter:2 weeks of winter	9.838	0.4578	9.38	0.9211	6
**203**	8 weeks post winter:8 weeks of winter vs. SPC -4 weeks post winter:4 weeks of winter	9.838	0.7064	9.131	0.9211	6
**204**	8 weeks post winter:8 weeks of winter vs. SPC -4 weeks post winter:8 weeks of winter	9.838	0.8233	9.014	0.9211	6
**205**	8 weeks post winter:8 weeks of winter vs. SPC -8 weeks post winter:2 weeks of winter	9.838	0.6341	9.204	0.9211	6
**206**	8 weeks post winter:8 weeks of winter vs. SPC -8 weeks post winter:4 weeks of winter	9.838	0.8233	9.014	0.9211	6
**207**	8 weeks post winter:8 weeks of winter vs. SPC -8 weeks post winter:8 weeks of winter	9.838	2.15	7.688	0.9211	6
**208**	SPC -4 weeks post winter:2 weeks of winter vs. SPC -4 weeks post winter:4 weeks of winter	0.4578	0.7064	-0.2486	0.9211	6
**209**	SPC -4 weeks post winter:2 weeks of winter vs. SPC -4 weeks post winter:8 weeks of winter	0.4578	0.8233	-0.3655	0.9211	6
**210**	SPC -4 weeks post winter:2 weeks of winter vs. SPC -8 weeks post winter:2 weeks of winter	0.4578	0.6341	-0.1763	0.9211	6

2way ANOVA<br>Multiple comparisons						
211	SPC -4 weeks post winter:2 weeks of winter vs. SPC -8 weeks post winter:4 weeks of winter	0.4578	0.8233	-0.3655	0.9211	6
212	SPC -4 weeks post winter:2 weeks of winter vs. SPC -8 weeks post winter:8 weeks of winter	0.4578	2.15	-1.692	0.9211	6
213	SPC -4 weeks post winter:4 weeks of winter vs. SPC -4 weeks post winter:8 weeks of winter	0.7064	0.8233	-0.1169	0.9211	6
214	SPC -4 weeks post winter:4 weeks of winter vs. SPC -8 weeks post winter:2 weeks of winter	0.7064	0.6341	0.07224	0.9211	6
215	SPC -4 weeks post winter:4 weeks of winter vs. SPC -8 weeks post winter:4 weeks of winter	0.7064	0.8233	-0.1169	0.9211	6
216	SPC -4 weeks post winter:4 weeks of winter vs. SPC -8 weeks post winter:8 weeks of winter	0.7064	2.15	-1.444	0.9211	6
217	SPC -4 weeks post winter:8 weeks of winter vs. SPC -8 weeks post winter:2 weeks of winter	0.8233	0.6341	0.1892	0.9211	6
218	SPC -4 weeks post winter:8 weeks of winter vs. SPC -8 weeks post winter:4 weeks of winter	0.8233	0.8233	0	0.9211	6
219	SPC -4 weeks post winter:8 weeks of winter vs. SPC -8 weeks post winter:8 weeks of winter	0.8233	2.15	-1.327	0.9211	6
220	SPC -8 weeks post winter:2 weeks of winter vs. SPC -8 weeks post winter:4 weeks of winter	0.6341	0.8233	-0.1892	0.9211	6
221	SPC -8 weeks post winter:2 weeks of winter vs. SPC -8 weeks post winter:8 weeks of winter	0.6341	2.15	-1.516	0.9211	6
222	SPC -8 weeks post winter:4 weeks of winter vs. SPC -8 weeks post winter:8 weeks of winter	0.8233	2.15	-1.327	0.9211	6

1			
2			
3			
4			
5			
6			
7			
8			
9			
10			
11			
12			
13			
14			
15			
16			
17			
18			
19			
20			
21			
22			
23			
24			
25			
26			
27			
28			
29			
30			

31			
32			
33			
34			
35			
36			
37			
38			
39			
40			
41			
42			
43			
44			
45			
46			
47			
48			
49			
50			
51			
52			
53			
54			
55			
56			
57			
58			
59			
60			

61			
62			
63			
64			
65			
66			
67			
68			
69			
70			
71			
72			
73			
74			
75			
76			
77			
78			
79			
80			
81			
82			
83			
84			
85			
86			
87			
88			
89			
90			

91			
92			
93			
94			
95			
96			
97			
98			
99			
100			
101			
102			
103			
104			
105			
106			
107			
108			
109			
110			
111			
112			
113			
114			
115			
116	N2	q	DF
117			
118	6	0.3748	75
119	6	0.0747	75
120	6	1.136	75

**121**	6	2.611	75
**122**	6	9.307	75
**123**	6	2.57	75
**124**	6	7.492	75
**125**	6	14.1	75
**126**	6	0.307	75
**127**	6	0.0747	75
**128**	6	0.2543	75
**129**	6	0.03622	75
**130**	6	0.2543	75
**131**	6	2.291	75
**132**	6	0.3001	75
**133**	6	0.7611	75
**134**	6	2.236	75
**135**	6	8.932	75
**136**	6	2.195	75
**137**	6	7.117	75
**138**	6	13.72	75
**139**	6	0.6818	75
**140**	6	0.3001	75
**141**	6	0.1205	75
**142**	6	0.411	75
**143**	6	0.1205	75
**144**	6	1.917	75
**145**	6	1.061	75
**146**	6	2.537	75
**147**	6	9.233	75
**148**	6	2.495	75
**149**	6	7.417	75
**150**	6	14.02	75

**151**	6	0.3817	75
**152**	6	0	75
**153**	6	0.1796	75
**154**	6	0.1109	75
**155**	6	0.1796	75
**156**	6	2.217	75
**157**	6	1.475	75
**158**	6	8.171	75
**159**	6	1.434	75
**160**	6	6.356	75
**161**	6	12.96	75
**162**	6	1.443	75
**163**	6	1.061	75
**164**	6	0.8817	75
**165**	6	1.172	75
**166**	6	0.8817	75
**167**	6	1.156	75
**168**	6	6.696	75
**169**	6	0.04126	75
**170**	6	4.88	75
**171**	6	11.48	75
**172**	6	2.918	75
**173**	6	2.537	75
**174**	6	2.357	75
**175**	6	2.648	75
**176**	6	2.357	75
**177**	6	0.3198	75
**178**	6	6.737	75
**179**	6	1.816	75
**180**	6	4.788	75

**181**	6	9.614	75
**182**	6	9.233	75
**183**	6	9.053	75
**184**	6	9.343	75
**185**	6	9.053	75
**186**	6	7.016	75
**187**	6	4.922	75
**188**	6	11.53	75
**189**	6	2.877	75
**190**	6	2.495	75
**191**	6	2.316	75
**192**	6	2.606	75
**193**	6	2.316	75
**194**	6	0.2786	75
**195**	6	6.603	75
**196**	6	7.799	75
**197**	6	7.417	75
**198**	6	7.237	75
**199**	6	7.528	75
**200**	6	7.237	75
**201**	6	5.2	75
**202**	6	14.4	75
**203**	6	14.02	75
**204**	6	13.84	75
**205**	6	14.13	75
**206**	6	13.84	75
**207**	6	11.8	75
**208**	6	0.3817	75
**209**	6	0.5612	75
**210**	6	0.2708	75

**211**	6	0.5612	75
**212**	6	2.598	75
**213**	6	0.1796	75
**214**	6	0.1109	75
**215**	6	0.1796	75
**216**	6	2.217	75
**217**	6	0.2905	75
**218**	6	0	75
**219**	6	2.037	75
**220**	6	0.2905	75
**221**	6	2.328	75
**222**	6	2.037	75
