# Supplementary figures and images for: RNA profiling identifies novel, photoperiod-history dependent markers associated with enhanced saltwater performance in juvenile Atlantic salmon
Source: PLoS One. 2020 Apr 8;15(4):e0227496. doi: 10.1371/journal.pone.0227496 (PMC7141700; doi:10.1371/journal.pone.0227496)

Supplemental Information 4

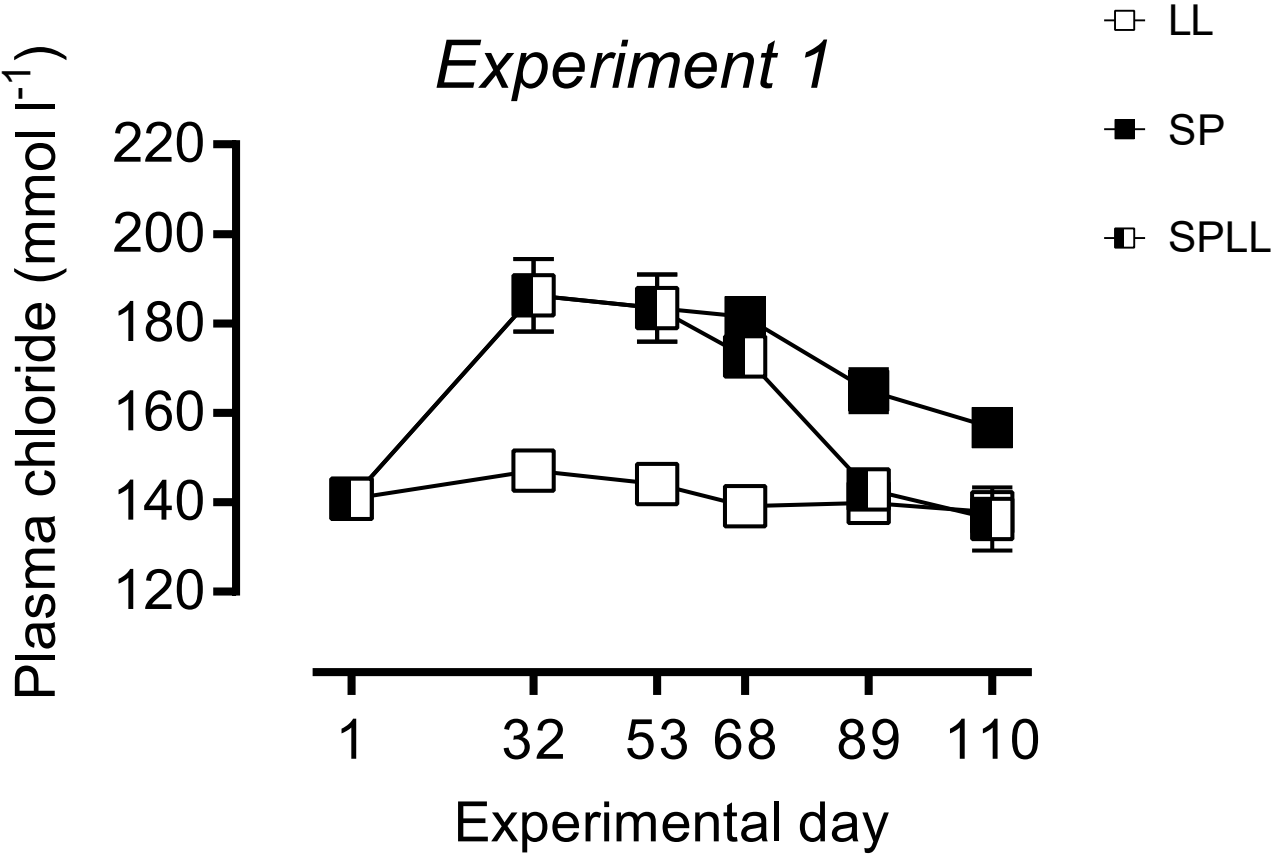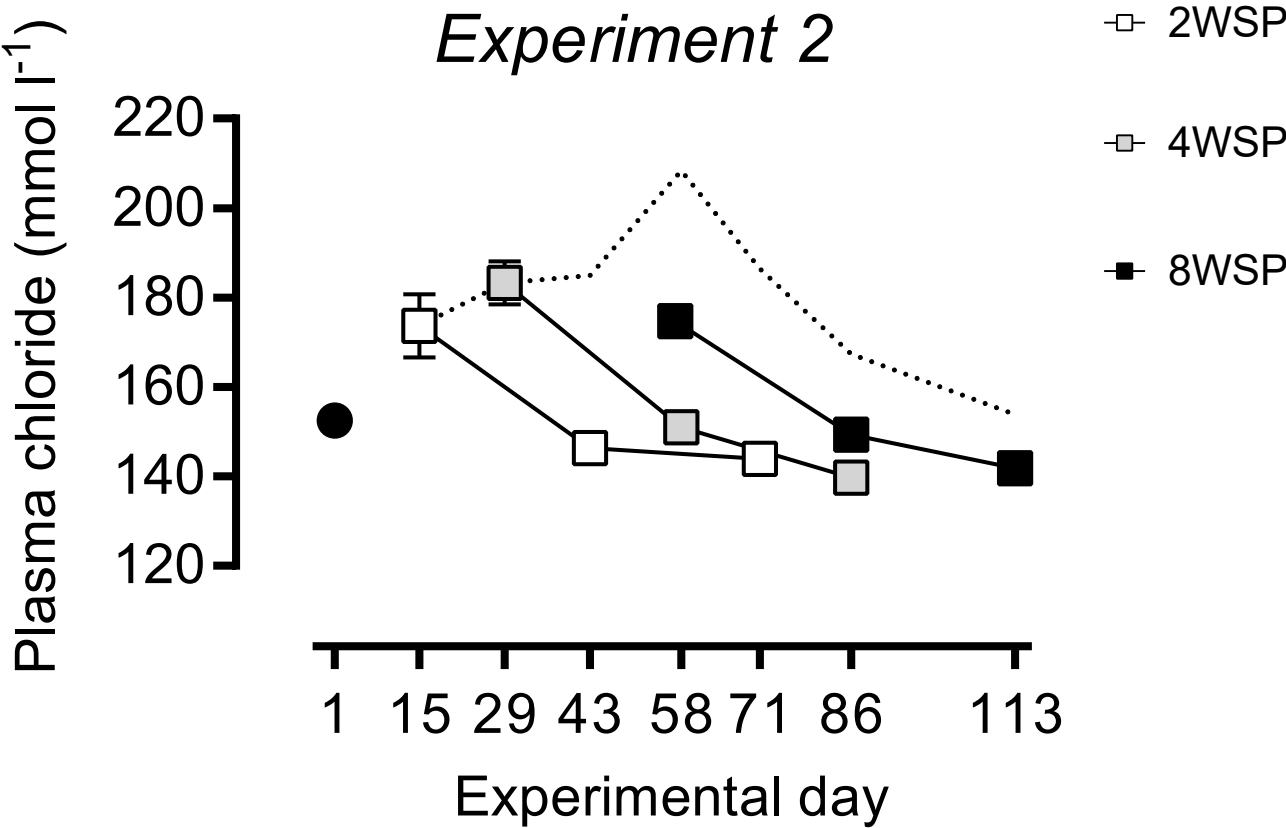

Supplement: S1 Fig — Graphs showing the averaged levels (±SEM) of plasma chloride (mmol l-1) in the treatments groups of experiment 1 (top, n = 6) and experiment 2 (bottom, n = 10, dotted line represents the SPC group), measured after 24h SWCs. (PDF) [file pone.0227496.s010.pdf]
